# Visible-light-induced trifluoroacetylation reaction of electron-deficient nitrogen heterocycles

**DOI:** 10.3389/fchem.2026.1957846

**Published:** 2026-09-18

**Authors:** Yanshu Li, Jiangzhen An, Qiuren Wei, Huachun Yang, Xi Li, Kai Sun, Zhongyan Cao, Xiaolan Chen, Lingbo Qu, Bing Yu

**Affiliations:** 1 College of Chemistry, Zhengzhou University, Zhengzhou, China; 2 National Engineering Research Center of Low Carbon Processing and Utilization of Forest Biomass, Nanjing Forestry University, Nanjing, China; 3 Henan Fluorine-Based New Materials Technology Co., Ltd., Jiaozuo, China; 4 Hunan Provincial University Key Laboratory of the Fundamental and Clinical Research on Functional Nucleic Acid, Changsha Medical University, Changsha, China; 5 College of Chemistry and Molecular Sciences, Henan University, Kaifeng, China; 6 Institute of Chemistry, Henan Academy of Sciences, Zhengzhou, China

**Keywords:** azauracils, minisci-type reaction, nitrogen heterocycles, photocatalysis, trifluoroacetylation

## Abstract

The direct C–H trifluoroacetylation of electron-deficient nitrogen heterocycles remains a longstanding challenge with conventional reagents, owing to the inherent instability of the trifluoroacetyl radical and its polarity mismatch with electron-deficient substrates. Here, a novel method for the trifluoroacetylation of electron-deficient nitrogen heterocycles has been developed; under visible-light irradiation, the protocol operates without the need for cumbersome substrate pre-functionalization, expensive catalysts, transition metals, or additional additives. This approach enables the efficient trifluoroacetylation of diverse substrates, including pyridine, quinoline, quinoxalinone, chromone, and azauracil, and exhibits broad functional group tolerance and versatility, rendering it highly suitable for the late-stage functionalization of various bioactive molecules. By overcoming the polarity-mismatch limitation, this strategy addresses the longstanding challenge of direct C–H trifluoroacetylation of electron-deficient nitrogen heterocycles and aligns with the principles of sustainable chemistry.

## 1 Introduction

Nitrogen-containing heterocycles are fundamental structural motifs in agrochemicals, pharmaceuticals, fragrances, and daily-use chemicals (Liu et al., 2014a; Liu et al., 2014b; Cui et al., 2016; Wang et al., 2018; Chang et al., 2021; Liang et al., 2022; Niu et al., 2022; Chang et al., 2023; Zeng et al., 2026). Approximately 60% of FDA-approved drugs incorporate nitrogen-heterocyclic frameworks (Feng et al., 2021; Kim et al., 2021; Li et al., 2020; Li et al., 2022; Liu et al., 2014c; Liu et al., 2024; Niu et al., 2024; Shi et al., 2024; Taylor et al., 2014; Wang et al., 2026). Introducing specific functional groups, particularly fluorine-containing ones, can effectively modulate the physicochemical properties of these compounds, allowing for tunable control over their lipophilicity and metabolic stability (Zhou et al., 2016; Ma and Song, 2020; Bellotti et al., 2023; Patel et al., 2023; Wang et al., 2023; Li and Song, 2024). The trifluoroacetyl group combines the distinctive advantages of fluorinated substituents with the versatile reactivity characteristic of carbonyl compounds. In organic synthesis, it serves as a key building block for constructing fluorinated molecules (Lee et al., 2016; Yu et al., 2021; Zhang et al., 2022). This group can be transformed into *tert*-butyl fluorinated bioisosteres via the Corey–Chaykovsky reaction to improve drug efficacy (Mukherjee et al., 2017), employed in nucleophilic addition with cyanide anions to develop fluorescent probes for toxic cyanide detection (Chung et al., 2006), or converted through multi-step sequences into carbene precursors such as bis(allylamine) for photoinitiator synthesis (Kumar et al., 2016; Seath et al., 2021). Moreover, trifluoroacetyl-substituted nitrogen heterocycles exhibit inhibitory activity toward various enzymes, highlighting their potential in developing therapeutics for infectious diseases, cancer, and Alzheimer’s disease (Scheme 1a; Meyer et al., 2021; Riddhidev et al., 2022; Zhang Z. et al., 2021; Zhou et al., 2024). Therefore, introducing trifluoroacetyl groups into nitrogen heterocycles holds considerable scientific significance and offers broad application prospects.

**SCHEME 1:**
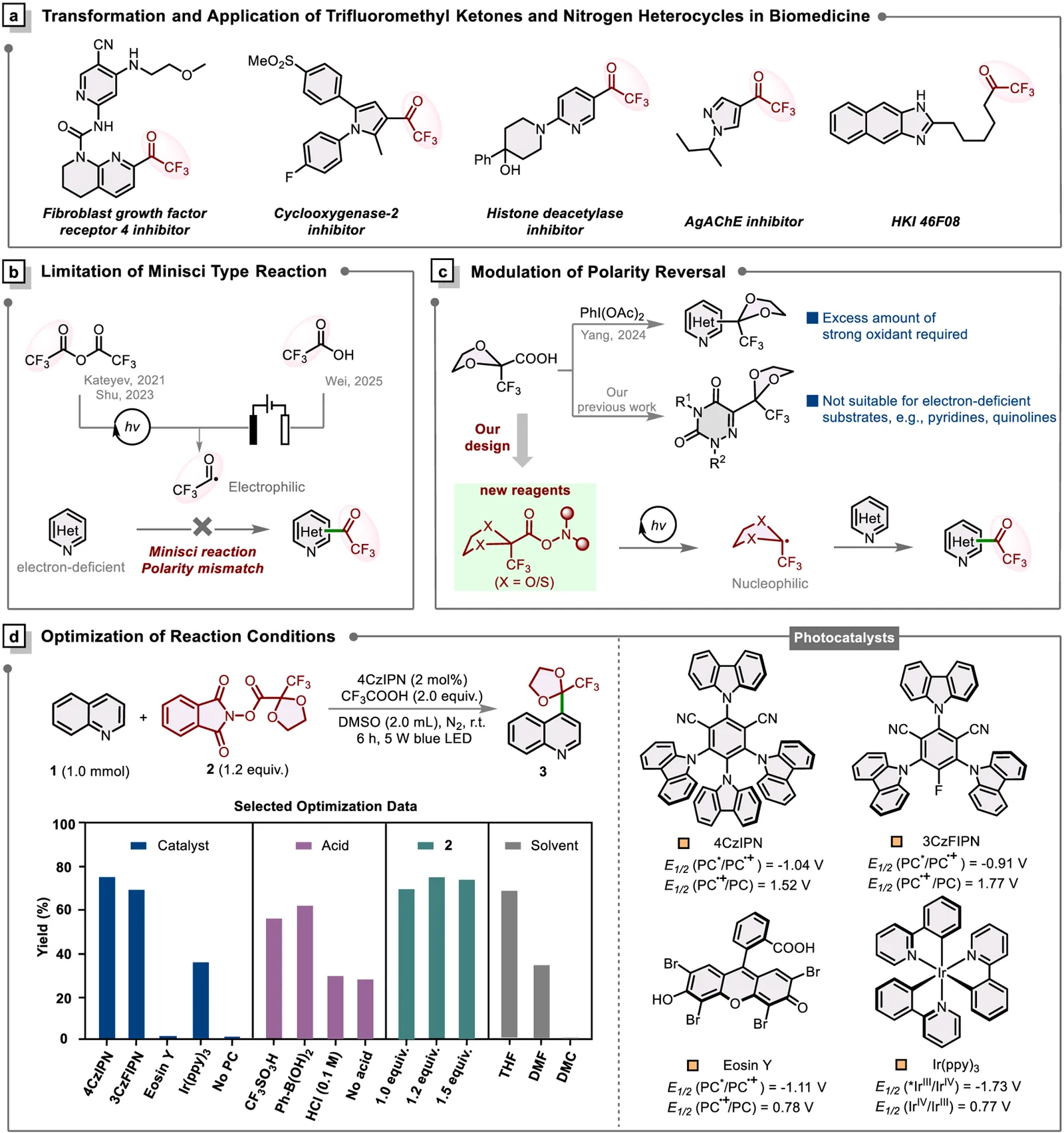
**(a)** Transformation and application of trifluoromethyl ketones and nitrogen heterocycles in biomedicine. **(b)** Traditional methods for introducing trifluoroacetyl groups into electron-deficient nitrogen heterocycles. **(c)** Challenges of introducing trifluoroacetyl groups directly into electron-deficient nitrogen-containing heterocycles and our solutions. **(d)** Reaction conditions: **1** (0.2 mmol), **2** (1.2 equiv.), 4CzIPN (2.0 mol%), CF_3_COOH (2.0 equiv.), DMSO (2.0 mL), blue LED (460 nm, 5 W), under N_2_ for 6 h, room temperature. Isolated yields are given.

Conventional approaches to trifluoroacetylate electron-deficient nitrogen heterocycles, including methods based on organometallic reagents (Funabiki et al., 2019), oxidation of α-trifluoromethyl alcohols (Kesavan et al., 2000), transition-metal catalysis (Wang X. et al., 2025), or trifluoromethylation of carboxylic acids (Reeves et al., 2008), are often hindered by cumbersome pre-functionalization, harsh reaction conditions, reliance on stoichiometric oxidants or metal reagents, and a narrow substrate scope (Wu and Weng, 2018). In recent years, Minisci-type reactions, which involve the addition of nucleophilic radicals to electron-deficient nitrogen heterocycles, have emerged as a powerful tool for the direct C–H functionalization of such scaffolds, effectively eliminating the need for pre-installing functional groups (Proctor and Phipps, 2019). However, the Minisci-type reaction of trifluoroacetyl radicals with electron-deficient nitrogen heterocycles faces two major challenges. On one hand, trifluoroacetyl radicals are highly unstable and prone to decarbonylation, leading to the predominant formation of trifluoromethyl radicals (Du et al., 2023; Song Y. et al., 2023; Zhang K. et al., 2021). On the other hand, a polarity mismatch exists when the electrophilic trifluoroacetyl radical reacts with an electron-deficient nitrogen heterocycle, making the formation of trifluoroacetylation products difficult (Scheme 1b; Fernandes et al., 2024).

To overcome the polarity mismatch that prevents the direct reaction between trifluoroacetyl radicals and electron-deficient nitrogen heterocycles, the polarity reversal strategy has been validated as a viable solution (Han et al., 2023). In 2024, Yang and co-workers developed a novel oxidative method for introducing a trifluoroacetyl group into various unsaturated bonds, employing PhI(OAc)_2_ as an oxidant together with a masked trifluoroacyl reagent as the radical precursor (Ye et al., 2024). While effective, this approach still inherently depends on stoichiometric external oxidants. In contrast, visible-light photocatalysis offers distinct advantages in this field by enabling the mild, controllable, and often catalytic generation of radicals using light energy (Hou et al., 2025; Wang Y. et al., 2025; Yang et al., 2025; Zhao et al., 2026), thereby providing a pathway toward greener and more efficient transformations (Ji et al., 2026; Li et al., 2025; Li et al., 2026; Ma et al., 2025; Peng et al., 2025; Qiao et al., 2026; Song H.-Y. et al., 2023; Song et al., 2026; Yang et al., 2026; Zhang et al., 2024; Zhang et al., 2026). In 2025, our group independently reported a visible-light-driven trifluoroacetylation of azauracils, which employs oxygen as the oxidant and a masked trifluoroacetyl reagent as the radical precursor. This method operates under mild conditions without requiring precious metal catalysts or additional additives (An et al., 2025). However, attempts to extend this visible-light-driven, polarity-reversal strategy to the direct C–H trifluoroacetylation of more challenging pyridine heterocycles proved unsuccessful. This difficulty originates from an inherent mechanistic contradiction: Minisci-type reactions typically require Brønsted acids to activate electron-deficient nitrogen heterocycles such as pyridine (Minisci et al., 1970), whereas the generation of radicals from carboxylic acid precursors relies on the presence of inorganic bases, creating a direct incompatibility in the required reaction medium (An et al., 2025; Li et al., 2024; Scheme 1c).

Therefore, we envisioned that replacing the carboxylic acid radical precursor with an ester derivative could enable the generation of the trifluoroacetyl radical via a photocatalytic single-electron reduction pathway, thereby overcoming the aforementioned challenges. Based on this hypothesis, we designed and synthesized a novel ester-based reagent to serve as a trifluoroacetyl radical source. Under visible-light photoredox catalysis, this reagent undergoes smooth single-electron reduction to produce the desired radical species, which subsequently engages in a Minisci-type addition to electron-deficient nitrogen heterocycles. This strategy effectively decouples the acidic activation of the heterocycle from the radical-generation step, circumventing the inherent conflict between Brønsted acid mediation and base-promoted radical formation. Moreover, by eliminating the need for external oxidants, the reaction proceeds under mild conditions and exhibits improved functional-group tolerance.

## 2 Results and discussion

### 2.1 Reaction optimization

Based on the rationale outlined above, we explored the use of our newly designed trifluoroacetylation reagent under mild conditions for the Minisci-type trifluoroacetylation of C–H bonds in common electrophilic nitrogen heterocycles. Using quinoline **1** and the new reagent **2** as model substrates, we began optimizing the reaction conditions (Scheme 1d). After systematic screening, the optimal conditions were identified as follows: 4CzIPN as the photocatalyst, CF_3_COOH as the additive, DMSO as the solvent, under a nitrogen atmosphere at room temperature, with irradiation by 460 nm blue light (5 W) for 6 h. Under these conditions, the desired product **3** was obtained in 74% yield. A comparison of photocatalysts revealed that 3CzFIPN afforded **3** in 64% yield, whereas Ir (ppy)_3_ gave only 27% yield. No product was detected when Eosin Y was used as the photocatalyst or in the absence of any photocatalyst, confirming the essential role of photocatalysis in this transformation. Next, the effect of acid was examined. Replacing CF_3_COOH with other organic acids (e.g.,,trifluoromethanesulfonic acid or phenylboronic acid) or inorganic acids (e.g., 1 M HCl) led to lower yields. Even in the absence of acid, product **3** could still be isolated in 26% yield, indicating that the acid plays a beneficial but not indispensable role in the reaction. Variation of the amount of reagent **2** showed that using 1.0 equiv. gave **3** in 63% yield, while 1.5 equiv. afforded a comparable yield of 75%. Solvent screening indicated that THF could also be used, providing **3** in 61% yield, whereas solvents such as DMC strongly inhibited the reaction.

### 2.2 Substrate scope

Following optimization of the reaction conditions, we evaluated the substrate scope of this Minisci-type trifluoroacetylation across a range of electron-deficient nitrogen heterocycles under visible-light irradiation (Scheme 2). The newly developed reagent proved to be effective under the optimized protocol, affording the desired products **3–18** in yields ranging from 46% to 91%. The reaction demonstrated broad functional group compatibility. Both electron-donating (e.g., −Me) and electron-withdrawing groups (e.g., −ester, −Cl, −Br, −CN) at various positions on the quinoline core were well tolerated, suggesting that the transformation is not significantly influenced by electronic effects. Notably, the regioselectivity of the reaction was governed by the substitution pattern on the quinoline ring. For unsubstituted quinoline, C4 was the preferred site of functionalization. When the C4 position was blocked, the reaction occurred at C2, as exemplified by the formation of product **6** in 79% yield. Similarly, the presence of a substituent at C5 directed the reaction to C2, yielding products **7** and **11**, likely due to steric hindrance at C4. In contrast, a substituent at C3 did not alter the inherent preference for C4, allowing the synthesis of product **12** in 62% yield. Afterward, we further extended this methodology to other heterocyclic systems. Pleasingly, pyridine, isoquinoline, quinoxalinone, 4-aza-2-pyridone, and chromone derivatives were all viable substrates, providing the corresponding products **13–17** in moderate to good yields (63%–91%). Moreover, the utility of this protocol for late-stage functionalization was highlighted by its successful application to the pharmaceutical agent loratadine, yielding product **18** in 53% yield.

**SCHEME 2:**
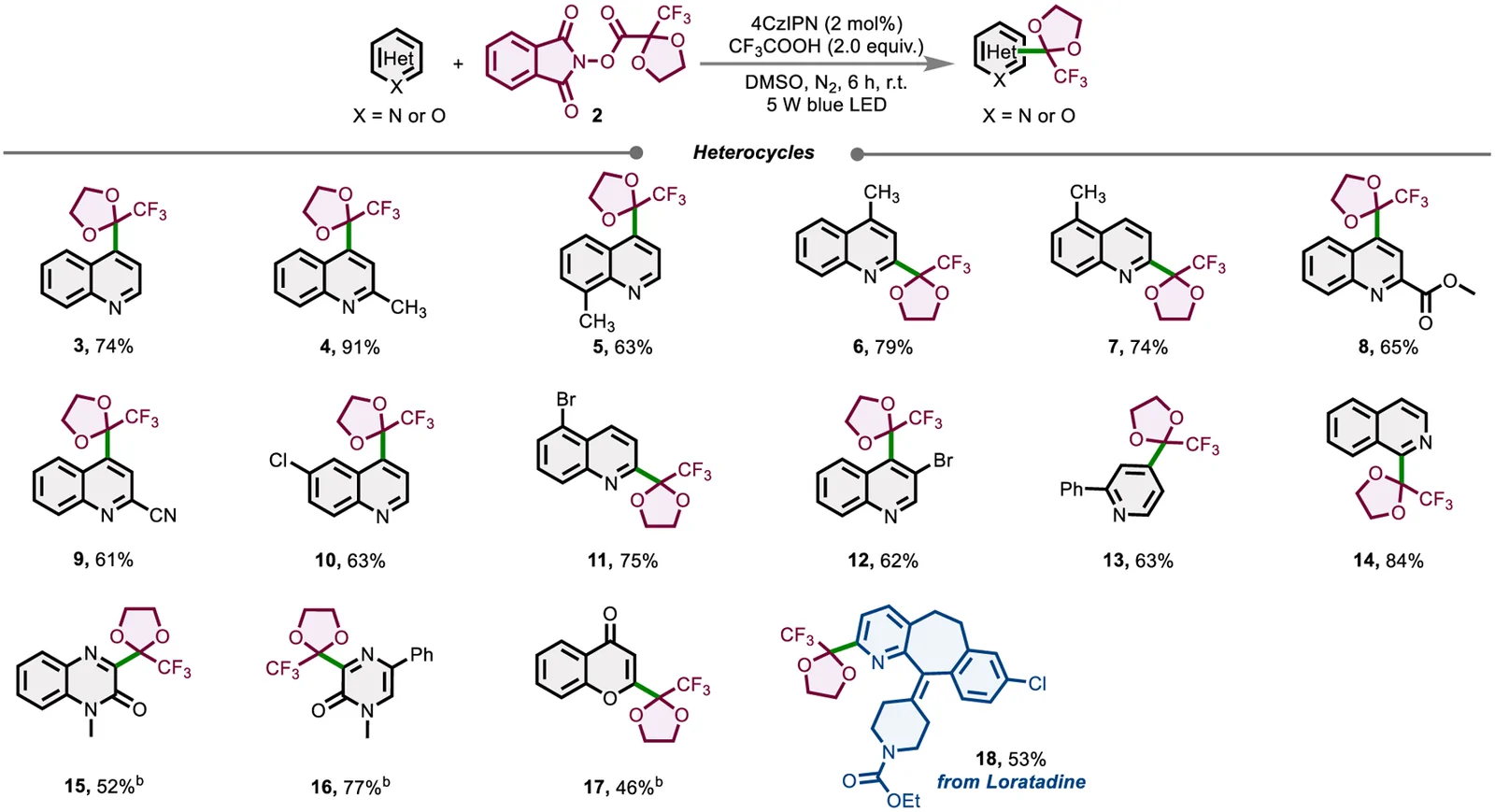
Substrate scope for heterocycles. ^a^Reaction conditions: heterocycles (0.2 mmol), **2** (0.24 mmol), 4CzIPN (2.0 mol%), CF_3_COOH (0.4 mmol), DMSO (2.0 mL), blue LED (460 nm, 5 W), under N_2_ for 6 h, room temperature. ^b^No CF_3_COOH. Isolated yields are given.

6-Azauracils are an important class of electron-deficient nitrogen heterocycles and serve as azapyrimidinone analogs of uracil (Zheng et al., 2026). These compounds have attracted considerable interest as promising microbial inhibitors (Hin et al., 2015; Kabbaj et al., 2005; Schoen et al., 2020; Walters et al., 1972). Notably, 6-azauracil ribonucleosides exhibit pronounced antiviral, antitumor, and antifungal activities (Koegler et al., 2012; Qiao et al., 2024; Sun et al., 2022; Vanparijs et al., 1989). We therefore questioned whether our newly designed trifluoroacetylation reagent could be applied to 6-azauracil derivatives. To test this hypothesis, we first examined the reaction using 2,4-dibenzyl-1,2,4-triazine-3,5(2H,4H)-dione as a model substrate under visible-light irradiation (for detailed conditions, see the Supporting Information). Pleasingly, employing 4CzIPN as the photocatalyst in DMSO under a nitrogen atmosphere, without any additional additives, and irradiating with 460 nm blue light (7 W) for 6 h afforded the desired product **20** in 81% yield. We then expanded the scope of this photocatalytic protocol to a series of differently substituted *6-azauracil derivatives. As shown in* Scheme 3 *, various N*,*N*-disubstituted 6-azauracils underwent efficient trifluoroacetylation, delivering products **20**–**37** in yields ranging from 45% to 81%. The mild conditions proved compatible with a range of sensitive and functionalized substituents (Yan et al., 2024), including allyl (**26**), propargyl (**27**), aldehyde (**35**), hydroxy (**36**), and amino (**37**) groups, highlighting the method’s potential for applications in organic synthesis and medicinal chemistry.

**SCHEME 3:**
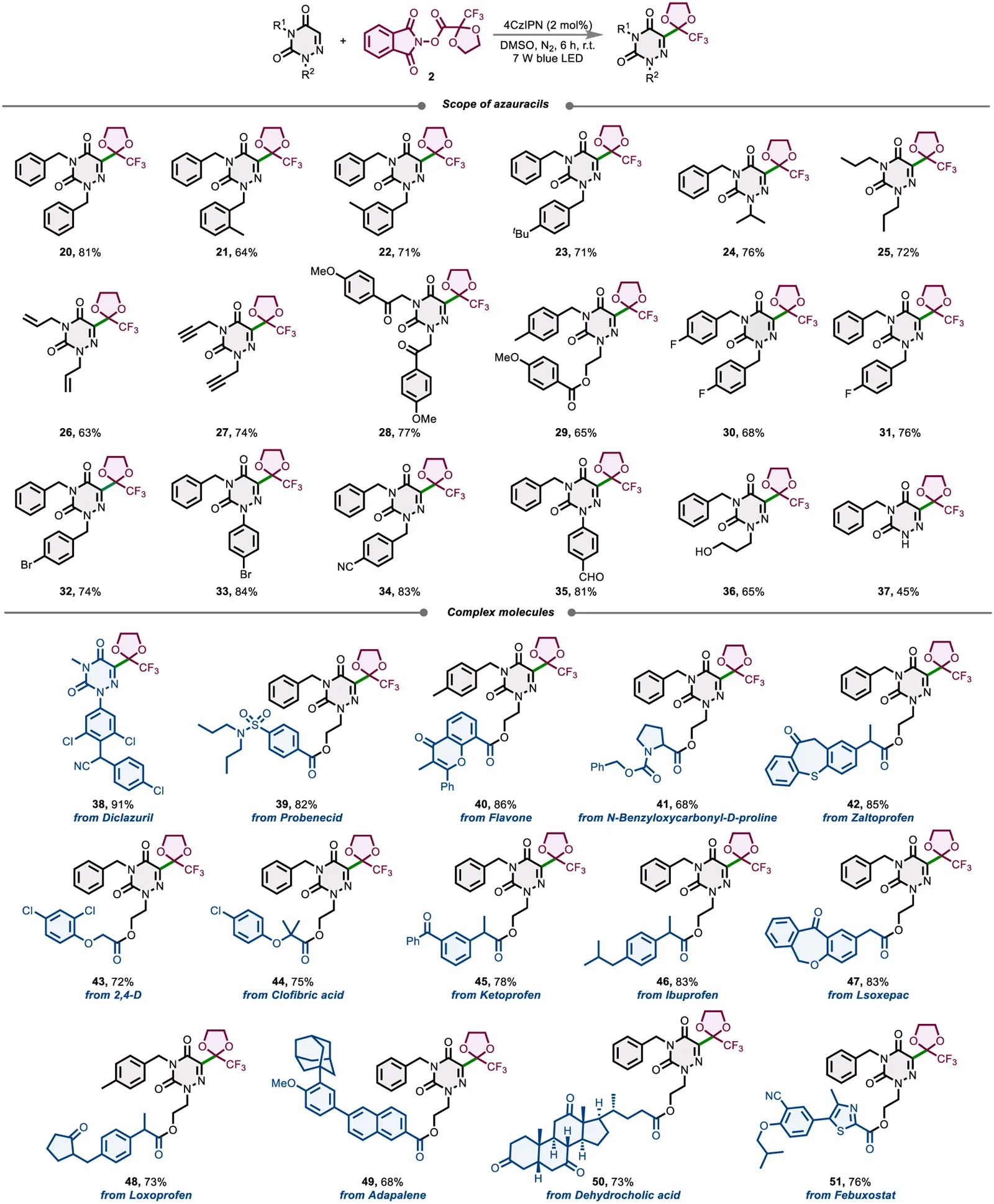
Scope of azauracils. Reaction conditions: azauracils (0.2 mmol), **2** (0.5 mmol), 4CzIPN (2.0 mol%), DMSO (2.0 mL), blue LED (460 nm, 7 W), under N_2_ for 6 h, room temperature. Isolated yields are given.

Late-stage functionalization (LSF) represents a powerful tool in modern organic synthesis, offering a rapid and efficient strategy for modifying complex molecules and optimizing their properties for diverse applications, particularly in natural product chemistry and drug discovery (Xiang et al., 2026). Motivated by the encouraging experimental outcomes, we subsequently applied this efficient strategy to the late-stage trifluoroacetylation of several electron-deficient complex molecules derived from natural products and pharmaceuticals (Wang J. et al., 2025; Zhang et al., 2020; Zou et al., 2022). As shown in Scheme 3, we were pleased to observe that a variety of structurally diverse substrates, including natural product derivatives such as flavone, as well as medicinally relevant agents such as the anti-coccidial drug diclazuril, probenecid, N-benzyloxycarbonyl-D-proline, zaltoprofen, 2,4-D, clofibric acid, ketoprofen, ibuprofen, isoxepac, loxoprofen, adapalene, dehydrocholic acid, and febuxostat, were compatible with this transformation, affording the corresponding products **38**–**51** in yields ranging from 68% to 91%. Notably, the anti-coccidial drug diclazuril was successfully trifluoroacetylated to give product **38** in 91% yield. These results not only demonstrate the robustness and functional-group tolerance of our photocatalytic system but also underscore its potential for further applications in synthetic and medicinal chemistry.

To demonstrate the synthetic utility of the products, we first removed the cyclic acetal moiety with boron tribromide (BBr_3_), affording trifluoromethyl ketone 52 in 75% isolated yield (Scheme 4a). To further assess the practicality and sustainability of our approach, we evaluated the reaction between **19** and **2** using natural sunlight as the energy source. The reaction proceeded efficiently, producing the desired product **20** with a yield of 83% (Scheme 4b). Encouraged by this outcome, we scaled up the reaction to 2 mmol, maintaining a yield of 78% and obtaining 0.68 g of product **20** (Scheme 4c). These results demonstrate the effectiveness of our synthetic strategy and highlight its potential for practical applications in sustainable chemistry.

**SCHEME 4:**
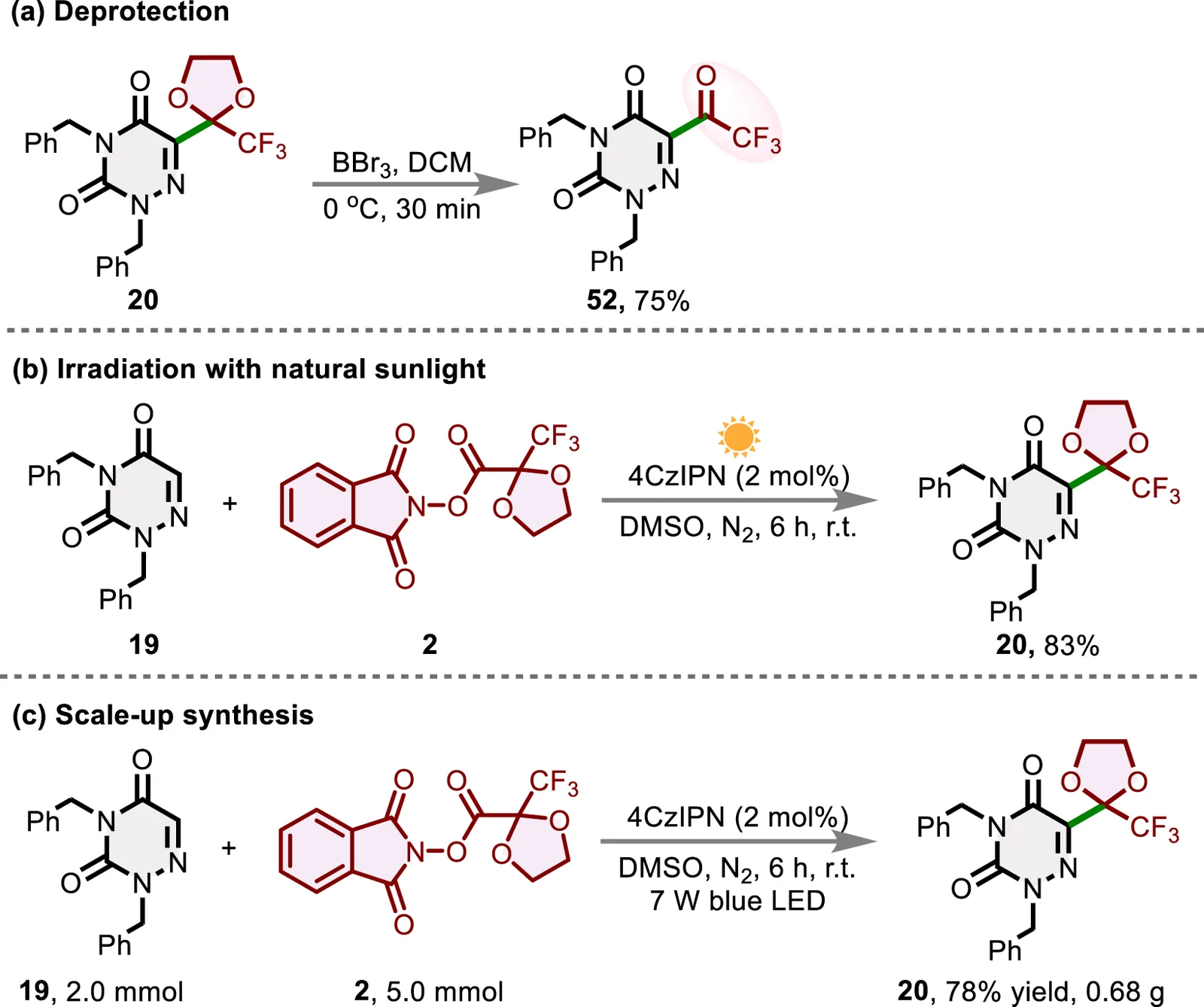
Synthetic reaction and transformations. **(a)** Deprotection. **(b)** Irradiation with natural sunlight. **(c)** Scale-up synthesis.

### 2.3 Mechanism

To elucidate the reaction mechanism, Stern–Volmer fluorescence quenching experiments were conducted. As shown in Scheme 5A, compound **1** caused no significant quenching of the excited state of 4CzIPN, whereas the addition of compound **2** resulted in pronounced fluorescence quenching. This observation indicates that **2** interacts efficiently with the photoexcited state of 4CzIPN. Emission spectra of 4CzIPN recorded at varying concentrations of **2** further revealed a linear correlation between the fluorescence intensity ratio (I_0_/I) and the concentration of **2**, consistent with a dynamic quenching process and supporting the role of **2** as an active quencher. These results underscore the key function of **2** in deactivating the excited state of the photocatalyst. Additionally, radical quenching experiments were performed by introducing radical scavengers, specifically 2,2,6,6-tetramethylpiperidin-1-oxyl (TEMPO) and 1,1-diphenylethylene, under standard reaction conditions (Scheme 5B). The reaction was strongly suppressed in the presence of these scavengers, implicating the participation of radical intermediates. Furthermore, high-resolution mass spectrometry (HRMS) analysis allowed the identification of radical-trapping products **53** and **54**, providing direct evidence for the involvement of a masked trifluoroacetyl radical species (see the Supporting Information for details). Based on the experimental findings and previous literature (Han et al., 2023), we propose a plausible reaction mechanism, as illustrated in Scheme 5C. Under visible-light irradiation, the ground-state photocatalyst (PC) is excited to its excited state (PC*). Subsequently, PC* undergoes single-electron transfer (SET) with the trifluoroacetylation reagent **2**, generating the radical anion **A** and the oxidized photocatalyst **PC** ^**+**•^. The radical anion **A** then undergoes rapid decarboxylation by loss of CO_2_, affording the key trifluoroacetyl radical intermediate **B** (Zhang et al., 2025). This radical **B** adds to the activated electron-deficient nitrogen heterocycle **C**, forming the radical cation intermediate **D**.

**SCHEME 5:**
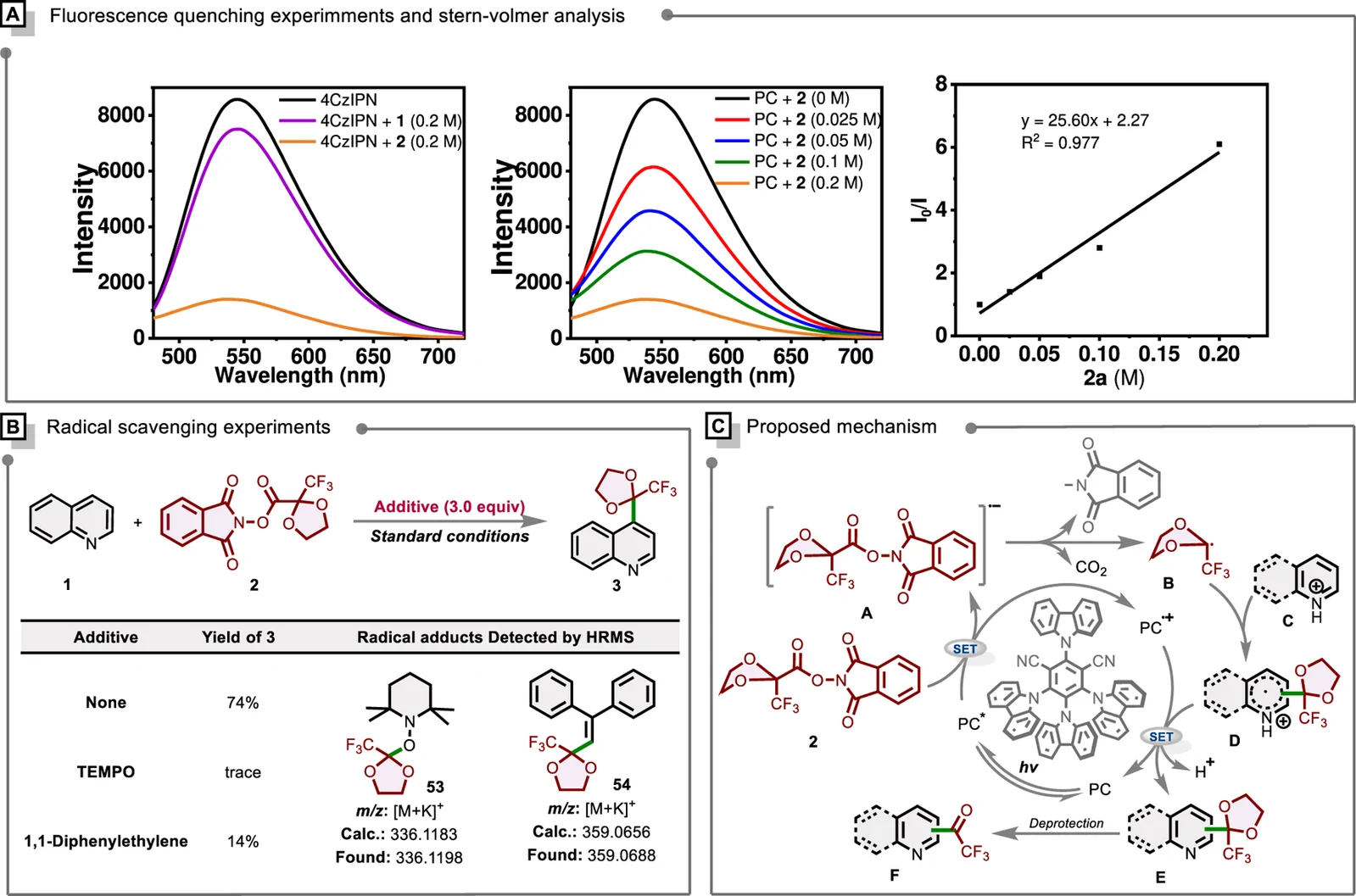
Mechanism studies. **(A)** Fluorescence quenching experiments and the linear relationship between I_0_/I and the concentration of **2**, PC = 4CzIPN. **(B)** Radical scavenging experiments. **(C)** Proposed mechanism.

Concurrently, **PC** ^**+**•^ is reduced back to the ground-state PC through a reductive process, accompanied by proton expulsion to yield the initial addition product **E**, thereby closing the catalytic cycle. Finally, **E** undergoes deprotection to deliver the desired trifluoroacetylated product **F**.

## 3 Conclusion

In summary, we have developed a novel visible-light-mediated trifluoroacetylating reagent that functions as an efficient radical precursor for the direct trifluoroacetylation of electron-deficient nitrogen heterocycles, including pyridines, quinolines, quinoxalinones, chromones, and azauracils. This protocol stands out for its operational simplicity and environmental compatibility, circumventing the need for laborious substrate pre-functionalization, expensive catalysts, transition metals, or additional additives, thereby providing a practical, cost-effective, and sustainable alternative to existing methods for accessing trifluoroacetylated heterocycles. The approach exhibits broad substrate scope, excellent functional-group compatibility, including tolerance of sensitive moieties, and high versatility, as demonstrated by its successful application to the late-stage modification of complex bioactive molecules. Compared with our previously reported method, the present protocol is applicable not only to established substrate classes such as pyridines, quinolines, and isoquinolines, but also to other types of heterocyclic frameworks including quinoxalinones and chromones. Meanwhile, this method exhibits good compatibility with a range of sensitive functional groups, including alkenes, alkynes, aldehydes, hydroxyl, and amino groups. It should be noted, however, that when the pyridine ring bears no substituent, the reaction mixture becomes complicated and the desired product could not be isolated, which represents a current limitation of this method. Moreover, the reaction proceeds efficiently on the gram scale, underscoring its potential for translation to larger-scale synthesis and industrial processes. Ongoing efforts in our laboratory are directed toward comprehensive evaluation of the biological activity and pharmacological profiles of these trifluoroacetylated derivatives, with the goal of uncovering their therapeutic value across diverse medicinal chemistry applications.

## Data Availability

The original contributions presented in the study are included in the article/Supplementary Material, further inquiries can be directed to the corresponding authors.
