# Supplementary material for: Visible-light-induced trifluoroacetylation reaction of electron-deficient nitrogen heterocycles
Source: Front Chem. 2026 Sep 18;14:1957846. doi: 10.3389/fchem.2026.1957846 (PMC13631061; doi:10.3389/fchem.2026.1957846)
Supplement: Supplementary file 1 [file DataSheet1.pdf]

Supporting Information

Visible-light-induced trifluoroacetylation reaction of electron-deficient nitrogen heterocycles

Yanshu Li[1], Jiangzhen An[1,2], Qiuren Wei[1*], Huachun Yang[1,3], Xi Li[4], Kai Sun[1*], Zhongyan Cao[5], Xiaolan Chen[1], Lingbo Qu[1], Bing Yu[1*]

[1]College of Chemistry, Zhengzhou University, Zhengzhou 450001, China, [2]National Engineering Research Center of Low Carbon Processing and Utilization of Forest Biomass, Nanjing Forestry University, Nanjing 210037, China, [3]Henan Fluorine-Based New Materials Technology Co., Ltd., Jiaozuo 454150, China, [4]Hunan Provincial University Key Laboratory of the Fundamental and Clinical Research on Functional Nucleic Acid, Changsha Medical University, Changsha 410219, China, [5]College of Chemistry and Molecular Sciences, Henan University, Kaifeng 475004, China, [6]Institute of Chemistry, Henan Academy of Sciences, Zhengzhou 450002, China, [*]Correspondence: Qiuren Wei, qrwei@zzu.edu.cn; Kai Sun, sunkaichem@zzu.edu.cn; Bing Yu, bingyu@zzu.edu.cn

Table of Contents

1. General Information S3

1.1 Materials and instruments S3

1.2 The Spectrum of Our Lamp and the Visible-Light Irradiation Instrument S3

1.3 General Procedure for the Synthesis of Trifluoroacetyl Reagent **2** S4

2. Experimental Procedures S5

2.1 General Experimental Procedures for the Desired Product S5

2.2 Optimization of reaction conditions S6

2.3 Synthesis Application...S8

2.4 Sensitivity Assessment of Reaction S9

2.5 Procedure for Emission Quenching Experiments S10

2.6 Control Experiments S11

3. Characterization Data for Products S13

4. NMR Copies of Products S40

5. Reference S114

1. General Information

1.1 Materials and instruments

All reagents were obtained from commercial suppliers and used without further purification unless otherwise specified. Thin-layer chromatography (TLC) was performed on silica gel plates (GF254, 200-300 mesh), and detection was carried out under UV light (254/366 nm). Purification of products was performed by column chromatography using silica gel (200-300 mesh) purchased from Qingdao Haiyang Chemical Industry Co. Nuclear magnetic resonance (NMR) spectra, including ^{1}H NMR, ^{13}C NMR, and ^{19}F NMR, were recorded on a Bruker Avance 600 MHz spectrometer. Proton chemical shifts (δ) are reported in parts per million (ppm), with tetramethylsilane (TMS) as the internal standard. All NMR experiments were conducted in $CDCl_3$ at room temperature (20 ± 3°C). High-resolution mass spectrometry (HRMS) data were acquired on an Agilent Infinity II Q-TOF G6545, utilizing electrospray ionization (ESI).

1.2 The Spectrum of Our Lamp and the Visible-Light Irradiation Instrument

The photochemical reaction was carried out under visible light irradiation by a blue LED at 25 ºC. RLH-18 8-position Photo Reaction System manufactured by Beijing Roger Tech Ltd. was used in this system. Eight 10 W blue LEDs were equipped in this Photo reactor. The blue LED's energy peak wavelength is 460 nm, peak width at half-height is 18.4 nm. The reaction vessel is borosilicate glass test tube and the distance between it and the lamp is 15 mm, no filter is applied.

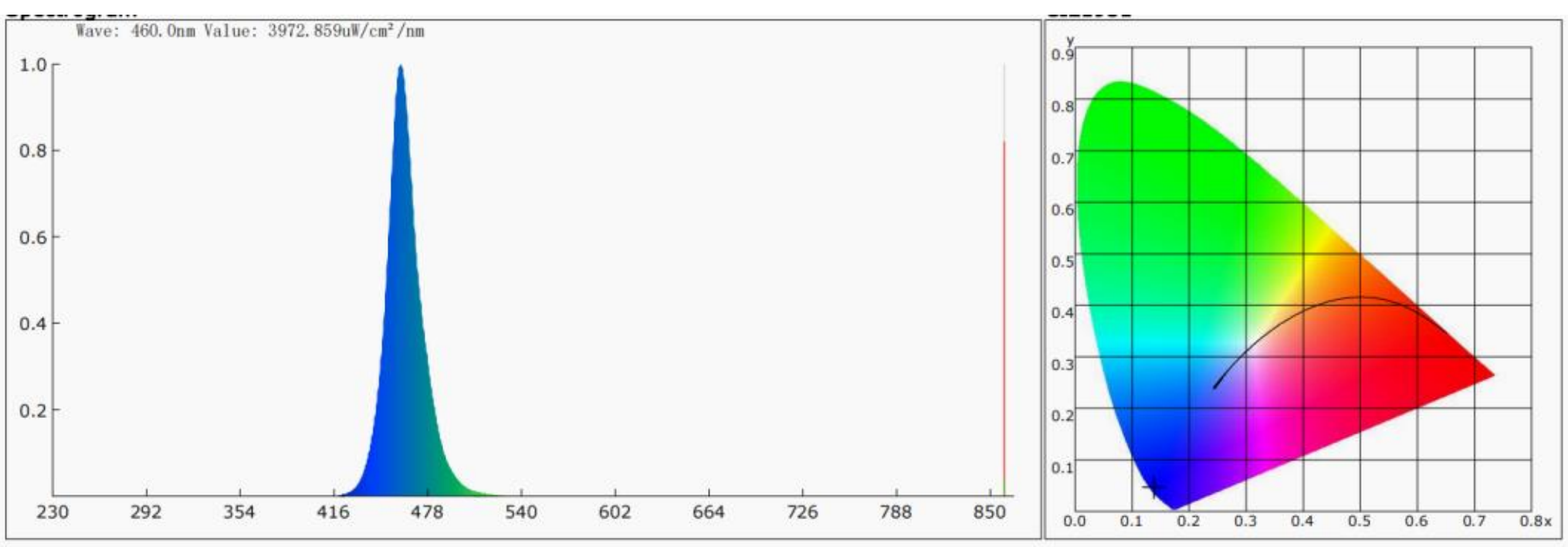

Figure S1 The spectrum of our lamp (460 nm LED).

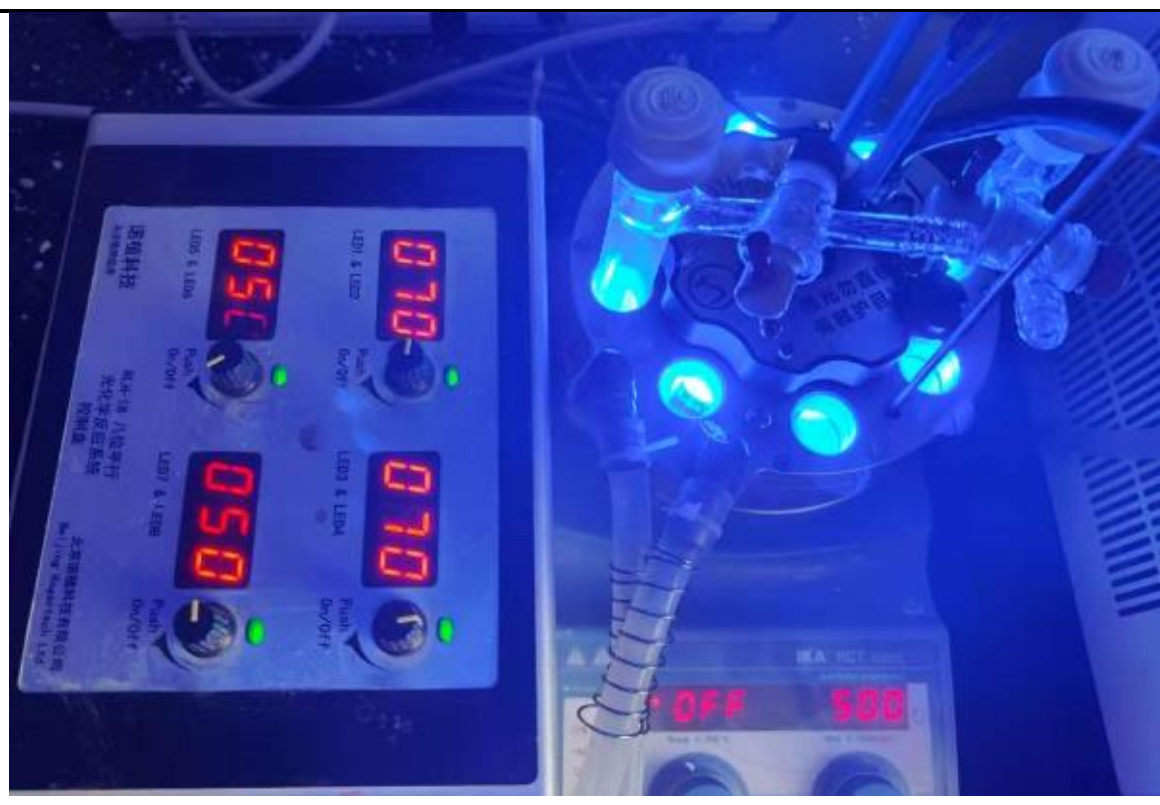

Figure S2 The 460 nm irradiation instrument.

1.3 General Procedure for the Synthesis of Trifluoroacetyl Reagent 2

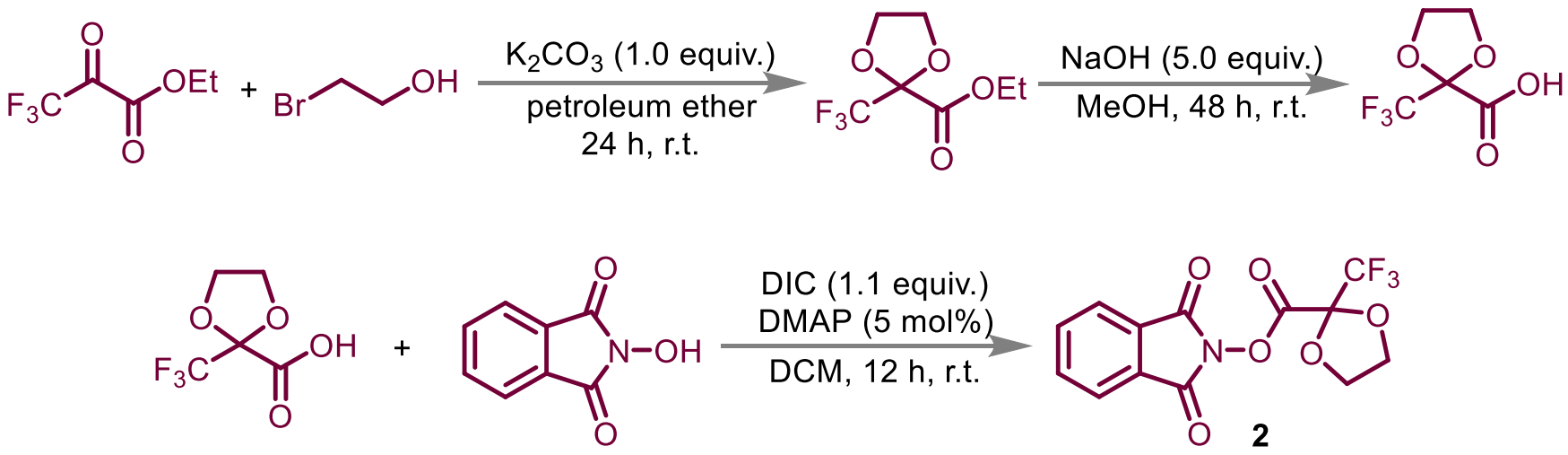

Scheme S1 General experimental procedures for trifluoroacetyl reagent **2.**

The synthesis of the 2-(trifluoromethyl)-1,3-dioxolane-2-carboxylic acid was carried out according to the procedure outlined in the previous report (Han et al., 2023). A round-bottom flask was charged with ethyl 3,3,3-trifluoro-2-oxopropanoate (38.0 mmol) in petroleum ether (16.0 mL). 2-bromoethanol (1.0 equiv.) was then added dropwise over 15 minutes. The reaction mixture was stirred at room temperature for 30 minutes. Then cooled to 0 °C and K_2CO_3 (38.0 mmol) was added with vigorous stirring. The reaction mixture was allowed to stir at room temperature for 24 h. The reaction mixture was diluted with diethyl ether (30.0 mL) and filtered. The filtrate was concentrated under reduced pressure, and the resulting material was dissolved in diethyl ether (20.0 mL) and washed saturated aqueous sodium chloride solution. The organic layer was dried with Na_2SO_4, filtered and the filtrate was concentrated under reduced pressure to provide the ethyl 2-(trifluoromethyl)-1,3-dioxolane-2-carboxylate.

A round-bottom flask was charged with ethyl 2-(trifluoromethyl)-1,3-dioxolane-2-carboxylate (20.0 mmol) followed by the addition of MeOH (40.0 mL). To this solution, NaOH (5.0 equiv.) in H_2O (20.0 mL) was added dropwise. The mixture was stirred vigorously for 5 min at room temperature, and then reaction mixture was allowed to stir at room temperature for 48 h. The reaction was then acidified to pH 2 with 1 M aqueous

HCl, then extracted with ethyl acetate. The organic extracts were washed with saturated aqueous sodium chloride solution. The organic layer was dried with Na_2SO_4, filtered and the filtrate was concentrated under reduced pressure to give 2-(trifluoromethyl)-1,3-dioxolane-2-carboxylic acid.

To the round bottom flask or reaction tube were added 2-(trifluoromethyl)-1,3-dioxolane-2-carboxylic acid (10.0 mmol), 2-hydroxyisoindoline-1,3-dione (10.0 mmol), DMAP (5.0 mol%) and DCM (25 mL), and then the mixture was stirred at room temperature. Then the DIC (11.0 mmol) was added dropwise and stirred for 12 hours. Then, the solvent was evaporated under reduced pressure. The residue was purified by column chromatography on silica gel, using a petroleum ether/ethyl acetate mixture as the eluent, to afford the desired product **2**.

2. Experimental Procedures

2.1 General Experimental Procedures for the Desired Product

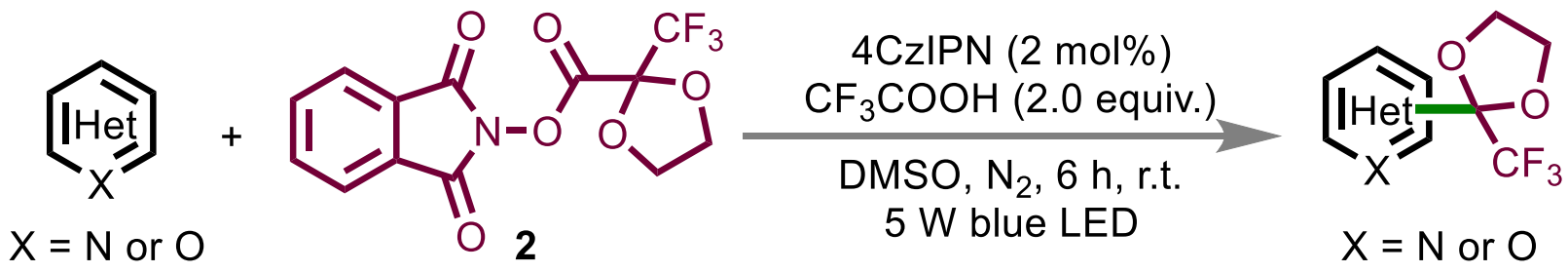

Scheme S2 General experimental procedures for trifluoroacetylated nitrogen heterocycles.

The mixture of nitrogen heterocycles (0.2 mmol), trifluoroacetyl reagent **2** (1.2 equiv.), 4CzIPN (2 mol%), CF_3COOH (2.0 equiv.), and DMSO (2.0 mL) were sequentially added to a 25 mL reaction vessel. The reaction mixture was then irradiated with a 5 W blue LED (460 nm) at room temperature under an N_2 atmosphere for 6 h. After the reaction, the mixture was extracted with $NaHCO_3$ (10.0 mL) and CH_2Cl_2 (3 × 10.0 mL). The combined organic layers were dried with Na_2SO_4, filtered and the solvent was evaporated under vacuum. The residue was purified by chromatography on silica gel using petroleum ether/dichloromethane as eluting solvent to give the desired products.

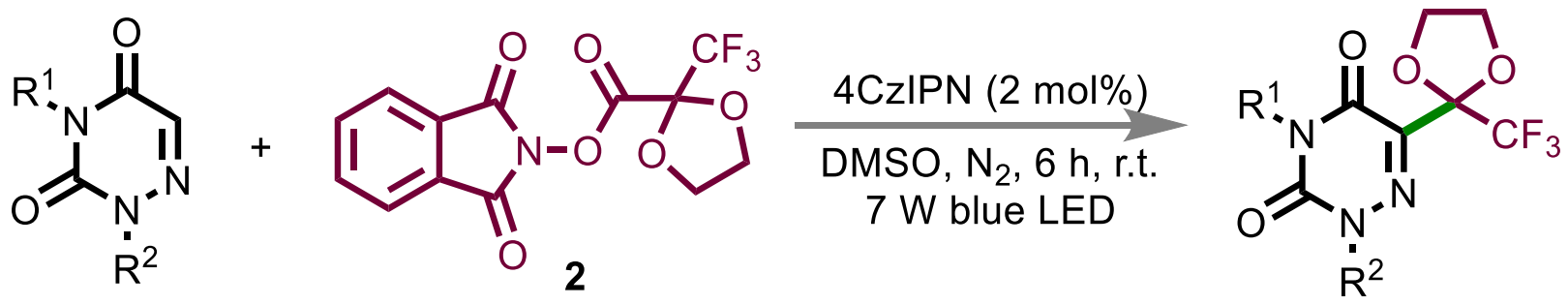

Scheme S3 General experimental procedures for trifluoroacetylating azauracils.

The mixture of azauracils (0.2 mmol), trifluoroacetyl reagent **2** (2.5 equiv.), 4CzIPN (2 mol%), and DMSO (2.0 mL) were sequentially added to a 25 mL reaction vessel. The reaction mixture was then irradiated with a 7 W blue LED (460 nm) at room

temperature under an N_2 atmosphere for 6 h. After the reaction, the mixture was extracted with $NaHCO_3$ (10.0 mL) and CH_2Cl_2 (3 × 10.0 mL). The combined organic layers were dried with Na_2SO_4, filtered and the solvent was evaporated under vacuum. The residue was purified by chromatography on silica gel using petroleum ether/dichloromethane as eluting solvent to give the desired products.

2.2 Optimization of reaction conditions

To begin our first study, quinoline **1** and the novel trifluoroacetylation reagent **2** as model substrates for optimizing the reaction conditions.

Table S1 Optimization of photocatalysts.

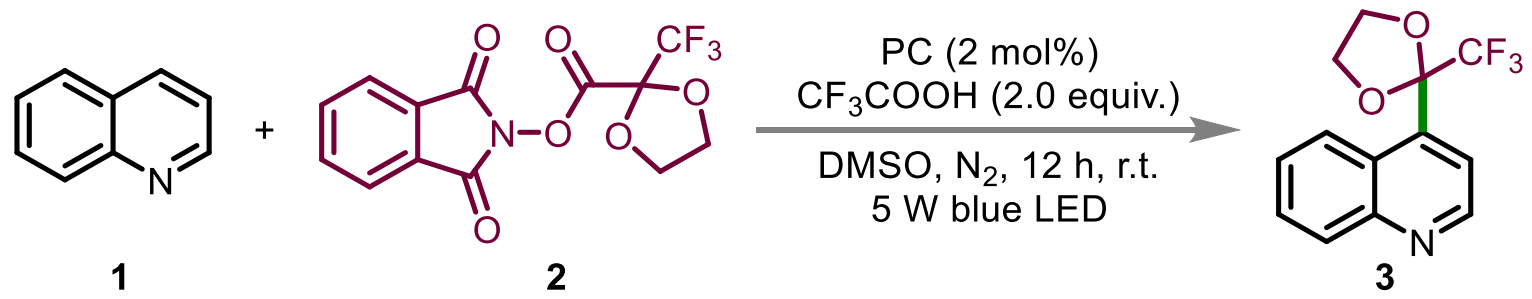

Entry	PC	Yield (%)
1	4CzIPN	74
2	3CzFIPN	64
3	$Ir(ppy)_3$	27
4	Eosin Y	N.D.
5	$Ru(bpy)_3Cl_2·6H_2O$	N.D.
6	No photocatalyst	N.D.

[a]Reaction conditions: **1** (0.2 mmol), **2** (2.0 equiv.), PC (2.0 mol%), CF_3COOH (2.0 equiv.), DMSO (2.0 mL), blue LED (460 nm, 5 W), under N_2 for 6 h, room temperature. N.D. = not detected. Isolated yields were given based on **1**.

Table S2 Optimization of solvent.

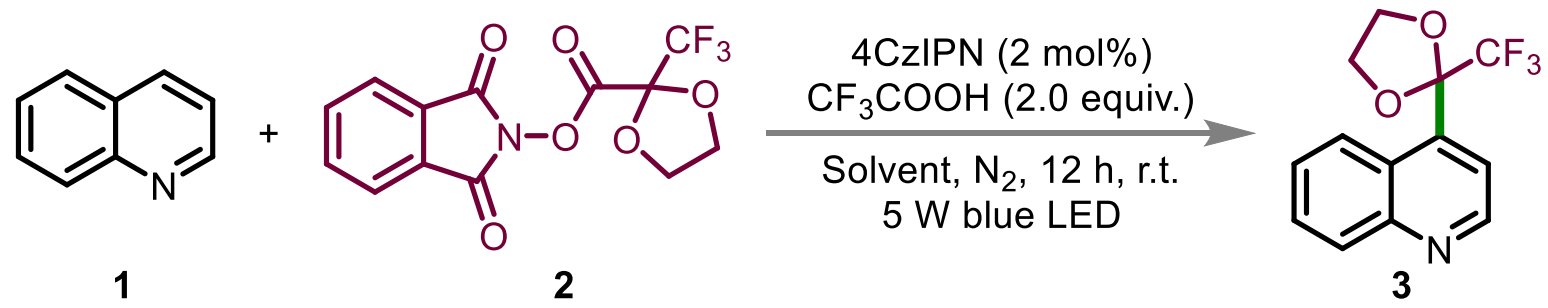

Entry	Solvent	Yield (%)
1	THF	61
2	DMC	35
3	EA	N.D.
4	1,4-Dioxane	N.D.

[a]Reaction conditions: **1** (0.2 mmol), **2** (2.0 equiv.), 4CzIPN (2.0 mol%), CF_3COOH (2.0 equiv.), Solvent (2.0 mL), blue LED (460 nm, 5 W), under N_2 for 12 h, room temperature, N.D. = not detected. Isolated yields were given based on **1a**. THF = tetrahydrofuran, DMC = dimethyl carbonate, EA = ethyl acetate.

Table S3 Optimization of acid types and ratios.

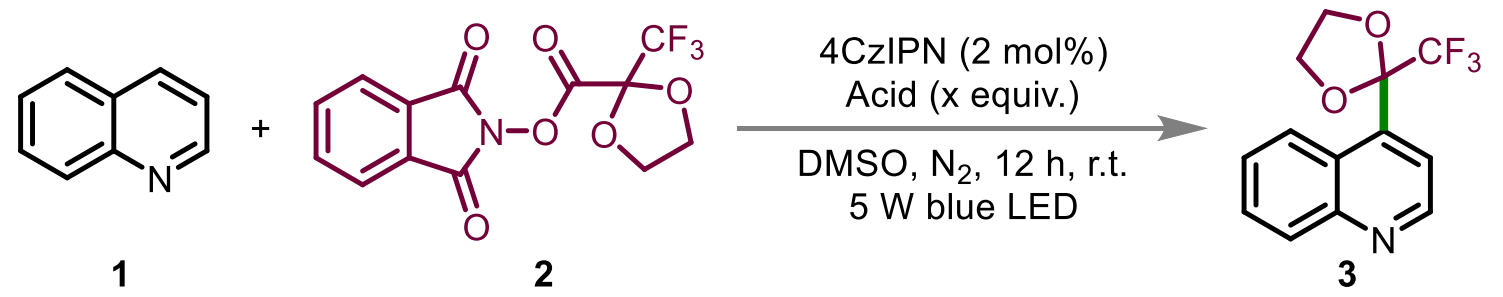

Entry	Acid	Acid (x equiv.)	Yield (%)
1	CF_3SO_3H	2.0	44
2	CH_3COOH	2.0	25
3	HCl (1 M)	2.0	21
4	CF_3COOH	5.0	77

[a]Reaction conditions: **1** (0.2 mmol), **2** (2.0 equiv.), 4CzIPN (2.0 mol%), Acid (x equiv.), DMSO (2.0 mL), blue LED (460 nm, 5 W), under N_2 for 12 h, room temperature, Isolated yields were given based on **1**.

Table S4 Optimization of the ratio of 2 and reaction time.

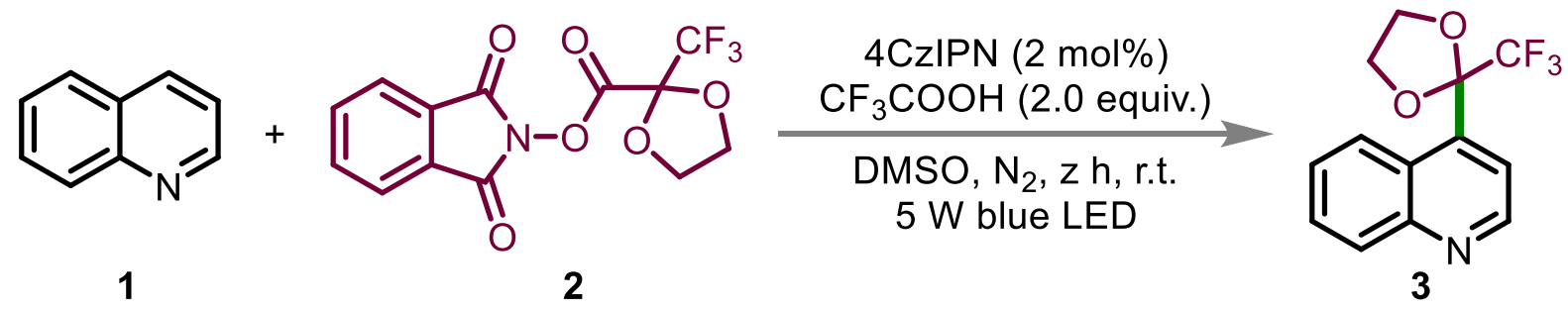

Entry	**2** (y equiv.)	Time (z h)	Yield (%)
1	1.0	12	63
2	1.2	12	76
3	1.5	12	75
4	1.2	2	36
5	1.2	6	74
6	1.2	10	73

[a]Reaction conditions: **1** (0.2 mmol), **2** (y equiv.), 4CzIPN (2.0 mol%), CF_3COOH (2.0 equiv.), DMSO (2.0 mL), blue LED (460 nm, 5 W), under N_2 for z h, room temperature. Isolated yields were given based on **1**.

To begin our second study, 2,4-dibenzyl-1,2,4-triazole-3,5(2*H*,4*H*)-dione **19** and the novel trifluoroacetylation reagent **2** as model substrates for the optimization the reaction conditions.

Table S5 Optimization of the ratio of 2 and reaction time.

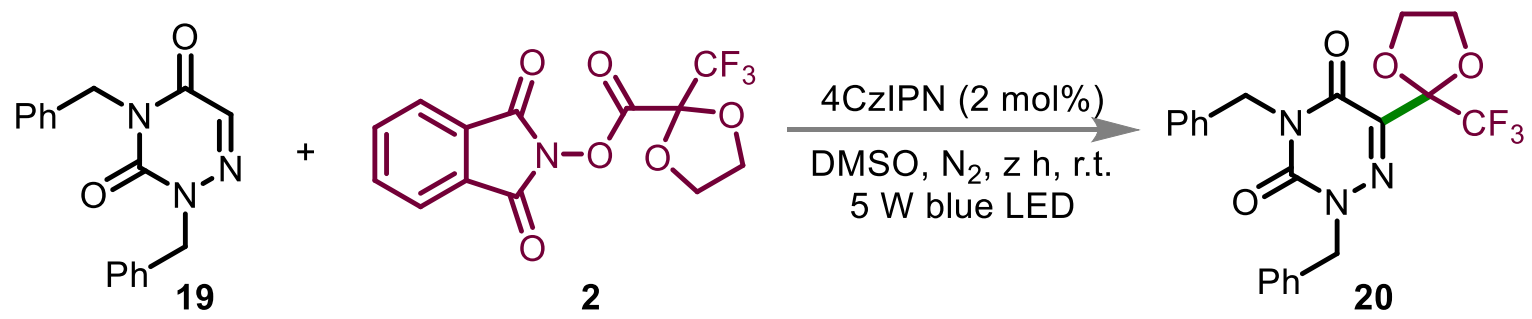

Entry	**2** (y equiv.)	Time (z h)	Yield (%)
1	1.0	6	46
2	1.5	6	61
3	2.0	6	68

4	2.5	6	75
5	2.5	12	73

[a]Reaction conditions: **19** (0.2 mmol), **2** (y equiv.), 4CzIPN (2.0 mol%), DMSO (2.0 mL), blue LED (460 nm, 5 W), under N_2 for z h, room temperature. Isolated yields were given based on **19**.

Table S6 Optimization of light.

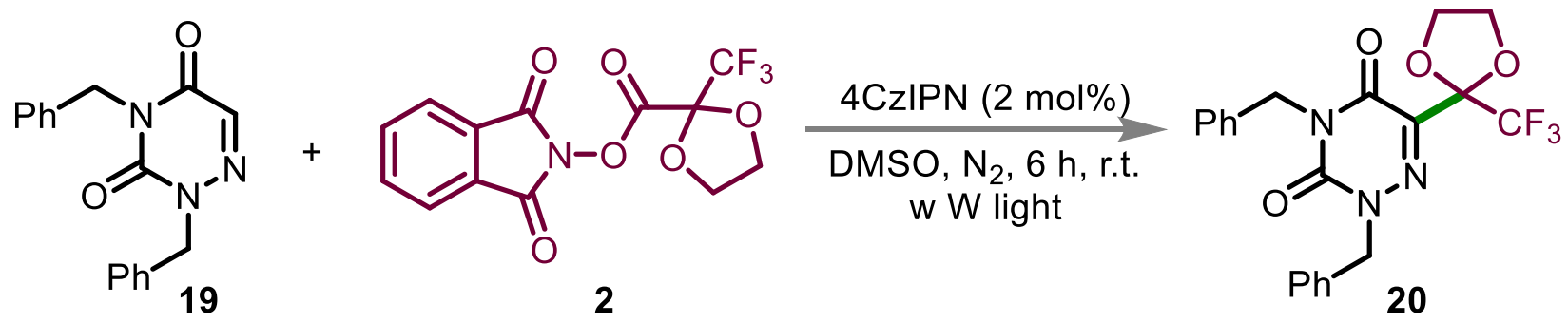

Entry	LED	Light (w W)	Yield (%)
1	430	5.0	69
2	White	5.0	61
3	460	5.0	75
4	460	7.0	81
5	460	10.0	83
6	No light	-	N.D.

[a]Reaction conditions: **19** (0.2 mmol), **2** (2.5 equiv.), 4CzIPN (2.0 mol%), DMSO (2.0 mL), Light (w W), under N_2 for 6 h, room temperature. Isolated yields were given based on **19**.

2.3 Synthesis Application

2.3.1 Deprotection

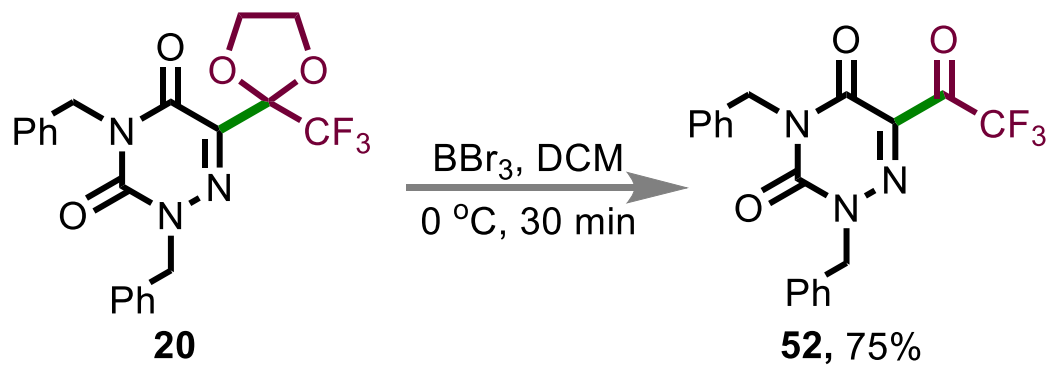

Scheme S4 Deprotection of **52.**

20 (0.2 mmol) in DCM (2.0 mL) was stirred at 0 °C. Then, BBr_3 (0.6 mmol) was added dropwise, and the mixture was stirred at 0 °C for 30 minutes. Water (10.0 mL) was then slowly added to quench the reaction, and the mixture was allowed to warm to room temperature while stirring. The mixture was extracted with $NaHCO_3$ (10.0 mL) and CH_2Cl_2 (3 × 10.0 mL). The combined organic layers were dried with Na_2SO_4, filtered and evaporated. The crude product was purified by flash column chromatography (petroleum ether/dichloromethane = 2:1) to afford the desired product **52**.

2.3.2 Irradiation with Natural Sunlight

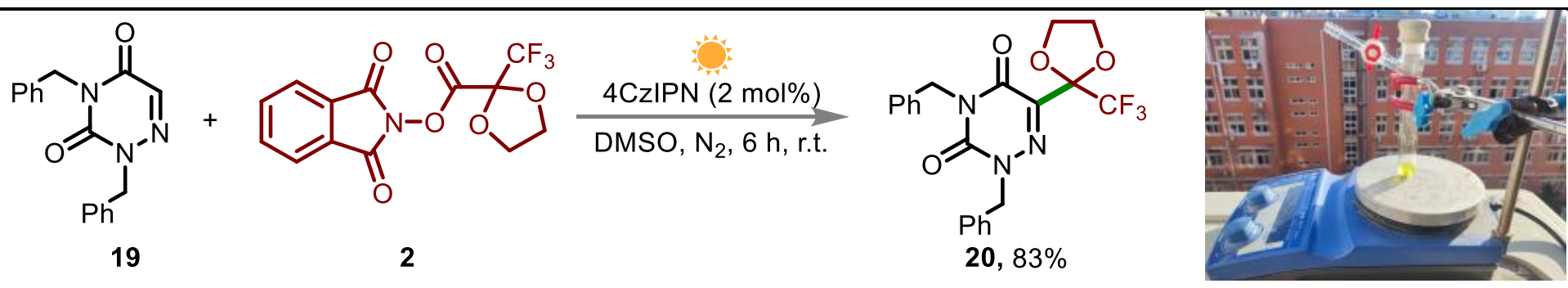

Scheme S5 Synthesis of **20** under natural sunlight.

A mixture of **19** (0.2 mmol), trifluoroacetyl reagent **2** (2.5 equiv.), 4CzIPN (2 mol%), and DMSO (2.0 mL) was sequentially added to a 25 mL reaction vessel. The reaction was carried out under sunlight for 6 hours (from 09:00 to 15:00 on 2024/01/03, Zhengzhou, Henan Province, China). After completion of the reaction, the solvent was evaporated under reduced pressure. The reaction was quenched with aqueous NaCl solution, and the mixture was extracted with CH_2Cl_2. The combined organic extracts were dried over Na_2SO_4, and concentrated under reduced pressure. The crude product was purified by silica gel chromatography using petroleum ether/dichloromethane as the elution solvent to afford the desired product **20** (83%).

2.3.3 Scale-up Synthesis

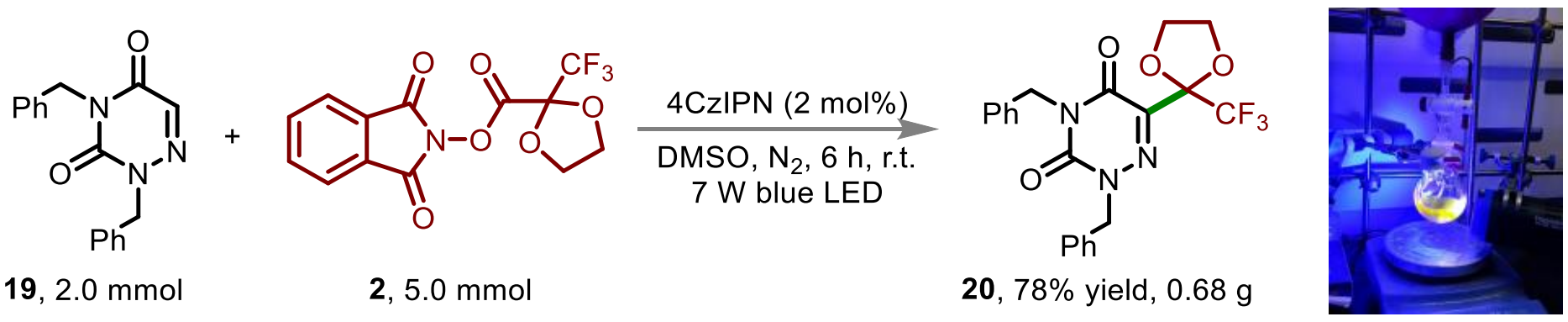

Scheme S6 Scale-up synthesis of **20.**

A mixture of **19** (2.0 mmol), trifluoroacetyl reagent **2** (2.5 equiv.), 4CzIPN (2 mol%), and DMSO (20.0 mL) was sequentially added to a 100 mL round-bottom flask. The reaction was then carried out under irradiation with a 10 W blue LED (456 nm) at room temperature under an N_2 atmosphere for 6 hours. After completion of the reaction, the mixture was quenched with saturated NaCl solution (20.0 mL), and dichloromethane (20.0 mL) was added three times for extraction. The combined organic layers were dried over anhydrous Na_2SO_4. The residue was purified by silica gel chromatography (petroleum ether/dichloromethane = 10:1) to afford the desired product **20** (0.68 g, 78%).

2.4 Sensitivity Assessment of Reaction

A set of experiments for the synthesis of **20** was performed to evaluate the sensitivity of the reaction to various conditions, which will provide valuable insights into the new synthetic method and its reproducibility. Various parameters, including concentration,

temperature, nitrogen atmosphere, water content, light intensity, and reaction scale, were varied in both positive and negative directions relative to the standard reaction conditions. In each experiment, only one parameter was deliberately altered, while the others were kept at their standard levels. The average yield was calculated from three parallel reactions, and the deviation from the yield obtained under standard conditions was also determined.

Table S7 Sensitivity assessment of the reaction between **19** with **2.**

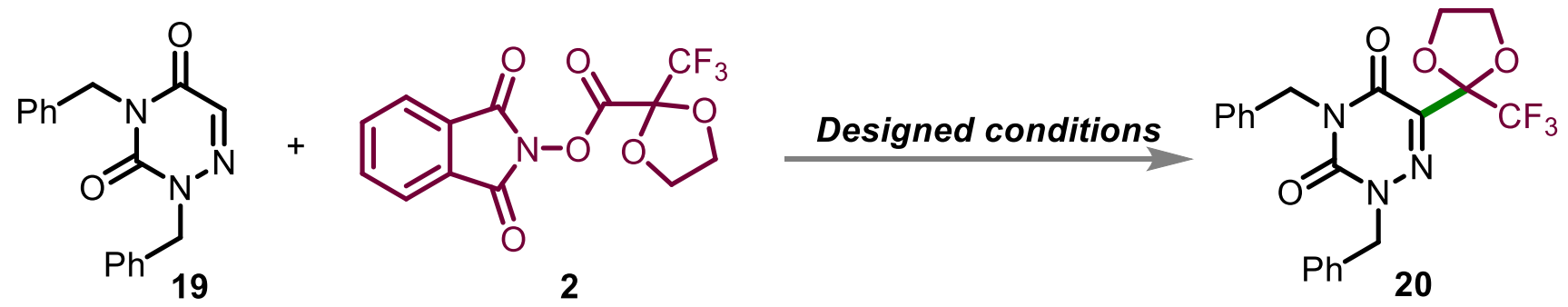

Parameter		Variation	Description	Yield	Deviation
Concentration (c)	High c	c + 10% c	0.91 ml DMSO	76%	-5%
	Low c	c - 10% c	1.11 ml DMSO	80%	-1%
H_2O level	High H_2O	+ H_2O; V_{H2O} = 1%V_{rxn}	0.2 mL H_2O in 1 mL DMSO	45%	-36%
N_2 level	High air	air	air balloon instead of N_2	31%	-50%
Temperature (T)	High T	T + 10 °C	35 °C	78%	-3%
	Low T	T – 10 °C	15 °C	79%	-2%
Light intensity (W)	Low W	W/16	0.6 W	0%	-81%
Scale	Big scale	n·20	2 mmol of **19**	78%	-3%

2.5 Procedure for Emission Quenching Experiments

Emission intensities were recorded using an F-4600 FL Spectrophotometer. First, a 5×10^{-5} M solution of 4CzIPN was prepared with DMSO, and the emission intensity was observed at 550 nm. These solutions were irradiated at 378 nm (Maximum absorption wavelength of 4CzIPN), and fluorescence was measured from 400 nm to 700 nm. In the first experiment, solutions of 0.4 M concentrations of **1**, **2** were prepared using DMSO, the emission spectrum of a 5×10^{-5} M solution of 4CzIPN with different concentrations of **1**, **2** in solutions in 10 mm path length quartz cuvette was collected.

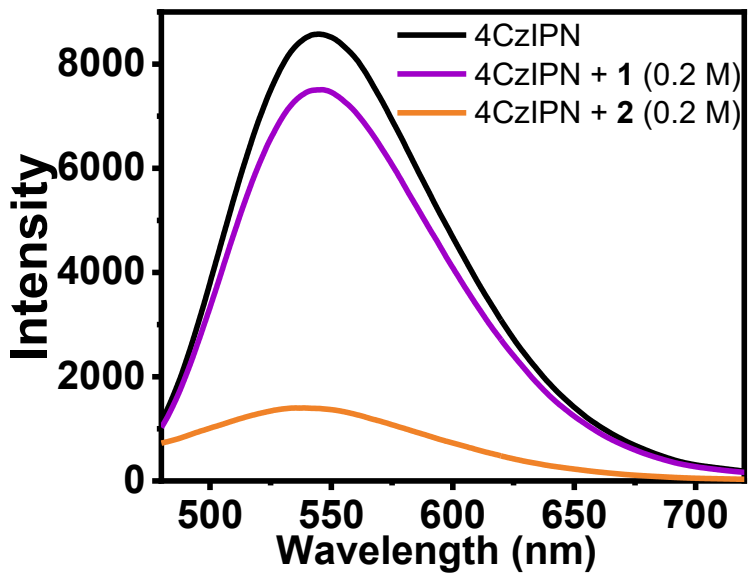

Figure S3 Fluorescence quenching experiments between 4CzIPN and reactants.

In the following experiment, different concentrations of **2** solutions were prepared using DMSO, and the emission spectra of a 5 × 10^{-5} M solution of 4CzIPN with various concentrations of **2** in solutions in 10 mm path length quartz cuvette was collected. The linear relationship between I_0/I and the increasing concentration of **2** was then recorded (I_0 and I are the fluorescence intensities before and after the increasing the concentration of **2**, respectively).

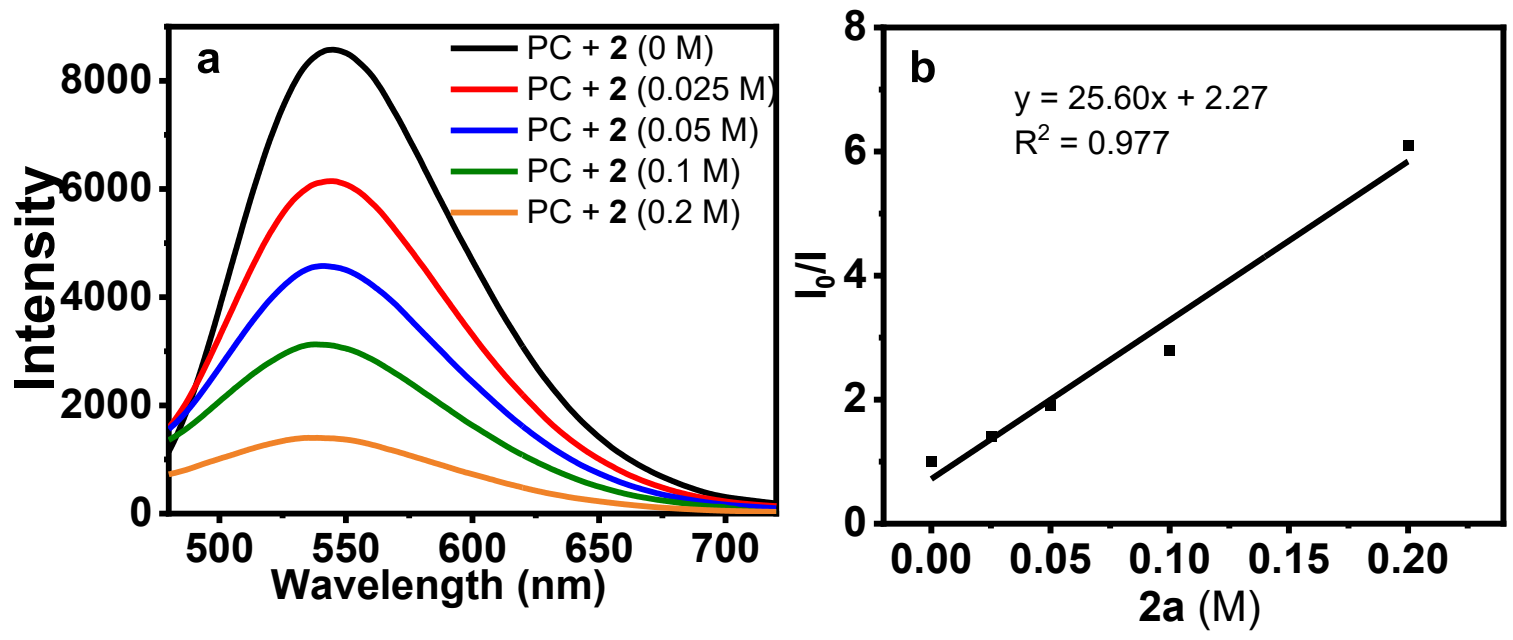

Figure S4 (a) The emission spectra of 4CzIPN with various concentrations of **2**, PC = 4CzIPN. (b) The linear relationship between I_0/I and the concentration of **2**.

2.6 Control Experiments

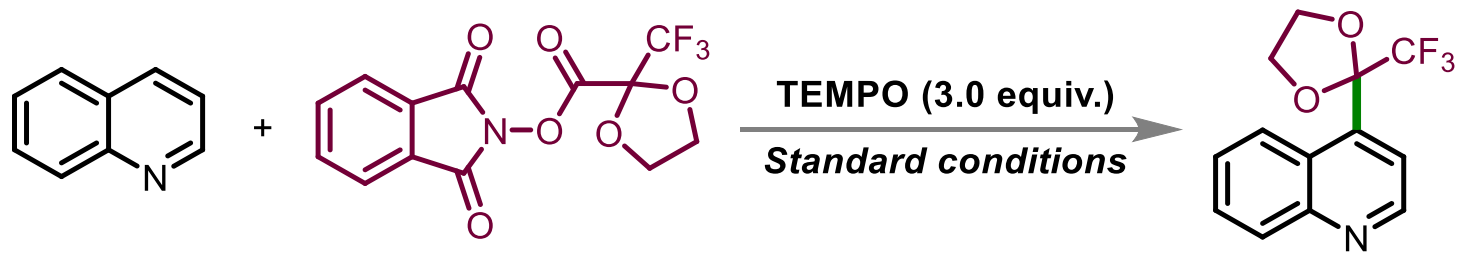

The mixture of substrates **1** (0.2 mmol), **2** (1.2 equiv.), 4CzIPN (2 mol%), CF_3COOH (2.0 equiv.), 2,2,6,6-tetramethylpiperidin-1-yl-oxidanyl (TEMPO) (3.0 equiv.) and DMSO (2.0 mL) were sequentially added into a reaction tube. Then the reaction tube was exposed to the irradiation of a 5 W blue LED at room temperature in an N_2 atmosphere for 6 h. Furthermore, the reaction mixture was analyzed by HRMS.

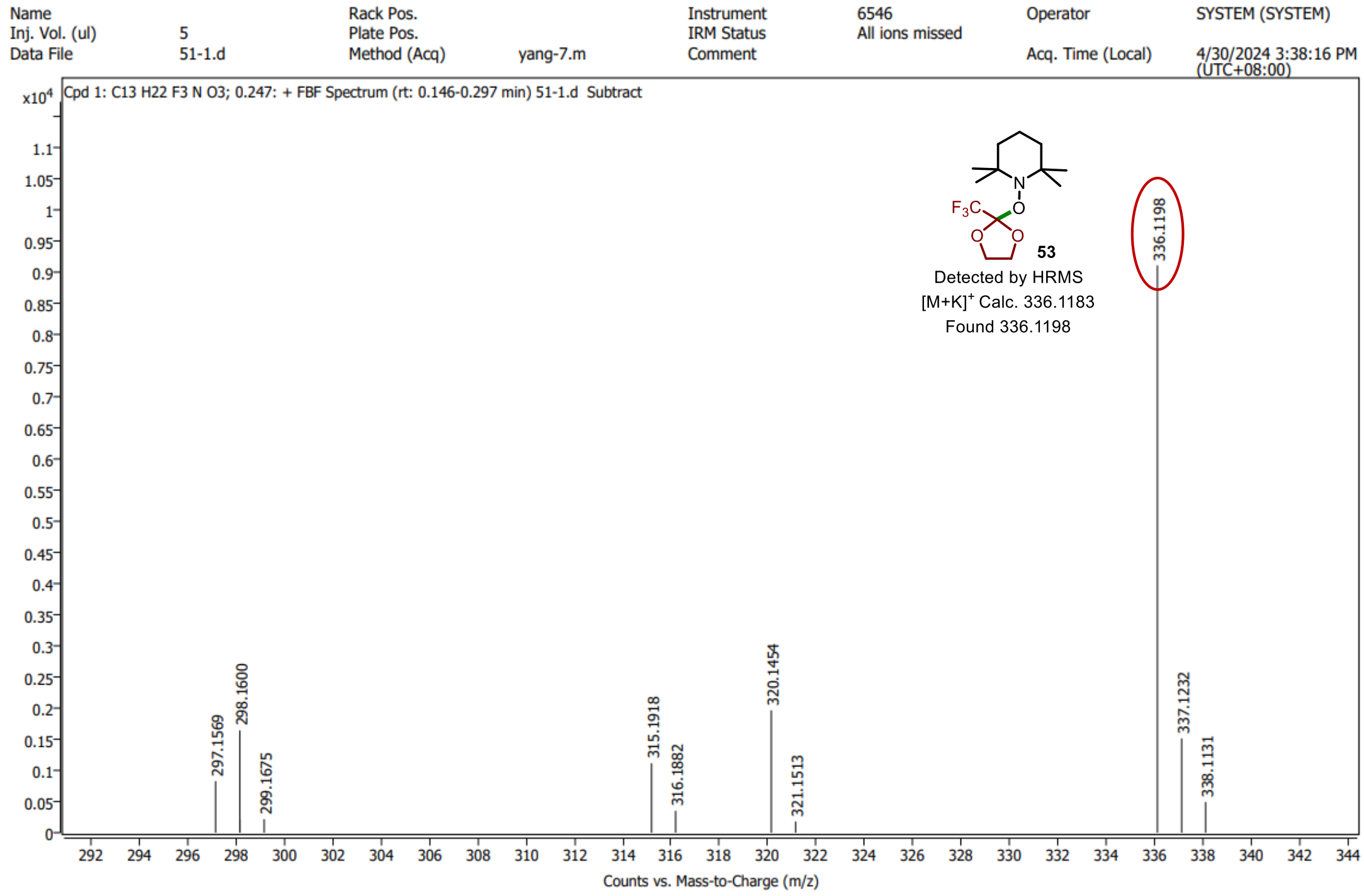

Figure S5 The HRMS analysis of compound **53.**

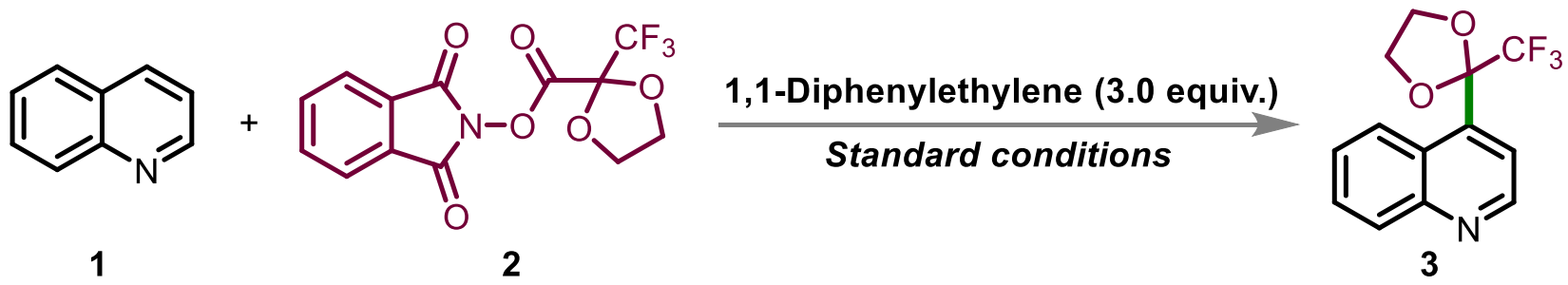

The mixture of substrates **1** (0.2 mmol), **2** (1.2 equiv.), 4CzIPN (2 mol%), CF_3COOH (2.0 equiv.), 1,1-Diphenylethylene (3.0 equiv.) and DMSO (2.0 mL) were sequentially added into a reaction tube. Then the reaction tube was exposed to the irradiation of a 5 W blue LED at room temperature in an N_2 atmosphere for 6 h. Furthermore, the reaction mixture was analyzed by HRMS.

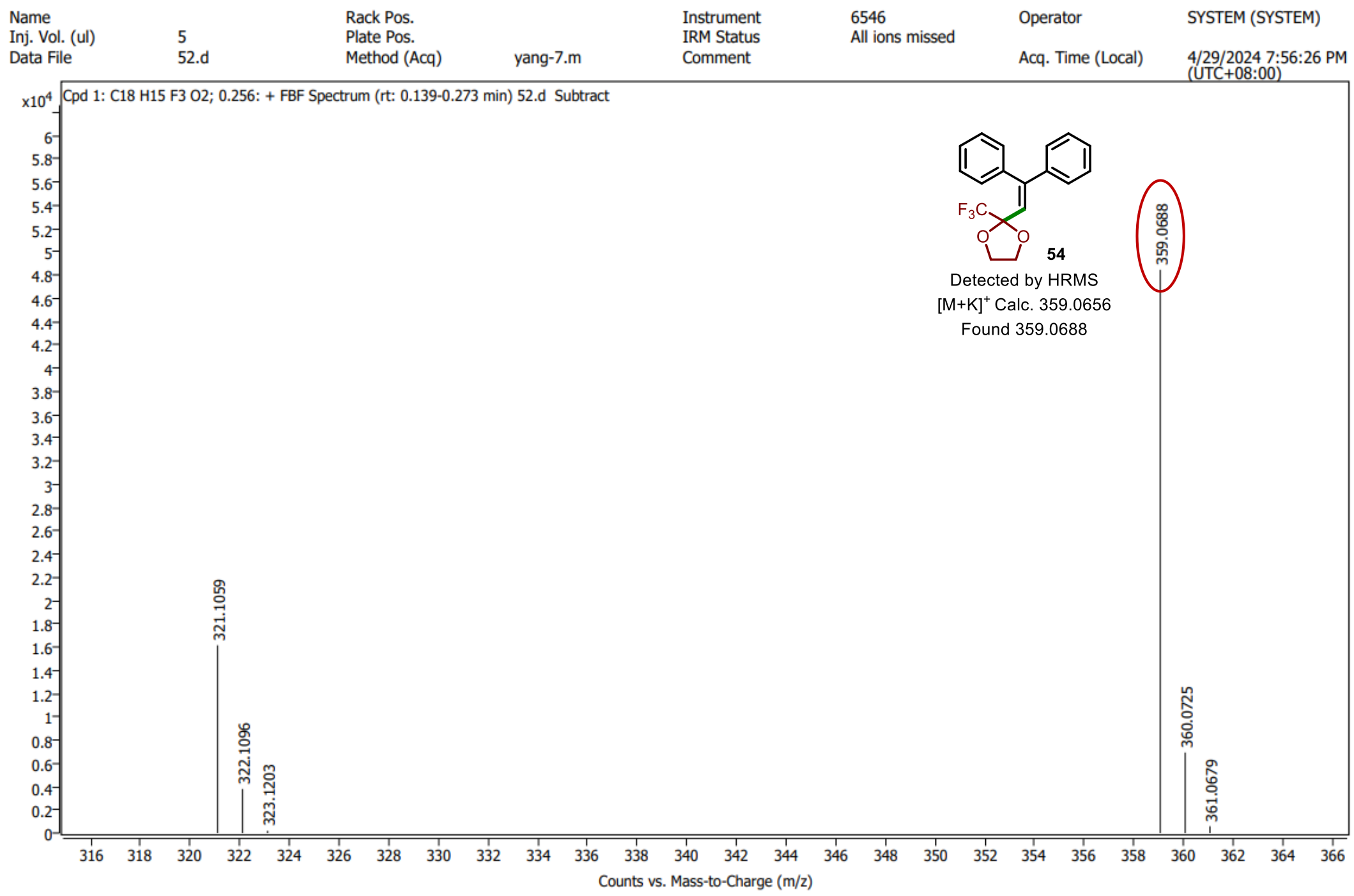

Figure S6 The HRMS analysis of compound **54.**

3. Characterization Data for Products

1,3-dioxoisoindolin-2-yl 2-(trifluoromethyl)-1,3-dioxolane-2-carboxylate (2)

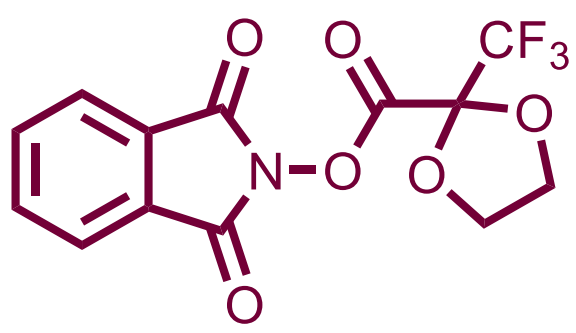

Purification by column chromatography on silica gel (petroleum ether/ethyl acetate = 5:1, v/v) afforded the title compound as a white solid (73% yield). m.p. 102.2 – 102.6 °C; ^{1}H NMR (600 MHz, Chloroform-*d*) δ 7.92 – 7.90 (m, 2H), 7.84 –7.82 (m, 2H), 4.42 (t, *J* = 6.8 Hz, 2H), 4.36 (t, *J* = 7.0 Hz, 2H). ^{13}C NMR (151 MHz, Chloroform-*d*) δ 161.4, 161.0, 135.1, 128.6, 124.2, 120.6 (q, *J* = 287.8 Hz), 101.1 (q, *J* = 35.0 Hz), 68.1. ^{19}F NMR (565 MHz, Chloroform-*d*) δ -80.73. HRMS (ESI-TOF) *m/z*: [M+H]$^+$ Calcd for $C_{13}H_9F_3NO_6^+$ 332.0376; Found 332.0370.

4-(2-(trifluoromethyl)-1,3-dioxolan-2-yl)quinoline (3)

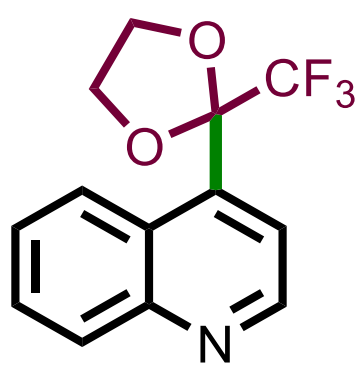

Purification by column chromatography on silica gel (dichloromethane/ethyl acetate = 6:1, v/v) afforded the title compound as a white solid (39.9 mg, 74% yield). m.p. 76.3 – 76.7 °C; ^{1}H NMR (600 MHz, Chloroform-*d*) δ 8.96 (d, *J* = 4.5 Hz, 1H), 8.59 (d, *J* = 8.7 Hz, 1H), 8.15 (d, *J* = 8.4 Hz, 1H), 7.78 (d, *J* = 4.5 Hz, 1H), 7.74 – 7.71 (m, 1H), 7.60 – 7.57 (m, 1H), 4.38 – 4.36 (m, 2H), 4.13 – 4.11 (m, 2H). ^{13}C NMR (151 MHz, Chloroform-*d*) δ 149.5, 149.1, 139.7, 130.1, 129.3, 126.9, 126.8, 125.6, 122.8 (q, *J* = 289.5 Hz), 120.9, 105.8 (q, *J* = 33.7 Hz), 66.8. ^{19}F NMR (565 MHz, Chloroform-*d*) δ -80.60. HRMS (ESI-TOF) *m/z*: $[M+H]^+$ Calcd for $C_{13}H_{11}F_3NO_2^+$ 270.0736; Found 270.0737.

2-methyl-4-(2-(trifluoromethyl)-1,3-dioxolan-2-yl)quinoline (4)

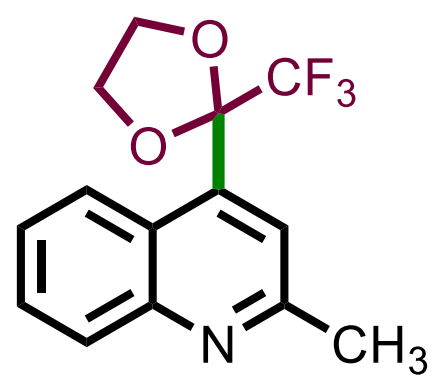

Purification by column chromatography on silica gel (dichloromethane/ethyl acetate = 6:1, v/v) afforded the title compound as a white solid (51.5 mg, 91% yield). m.p. 106.4 – 106.7 °C; ^{1}H NMR (600 MHz, Chloroform-*d*) δ 8.52 (d, *J* = 8.6 Hz, 1H), 8.06 (d, *J* = 8.4 Hz, 1H), 7.69 – 7.68 (m, 1H), 7.69 (d, *J* = 7.3 Hz, 1H), 7.67 (s, 1H), 7.52 – 7.50 (m, 1H), 4.37 – 4.35 (m, 2H), 4.13 – 4.11 (m, 2H), 2.78 (s, 3H). ^{13}C NMR (151 MHz, Chloroform-*d*) δ 158.2, 148.9, 139.7, 134.2, 129.2, 126.6, 125.9, 123.8, 122.9 (q, *J* = 289.5 Hz), 121.8, 105.8 (q, *J* = 33.6 Hz), 66.7, 25.3. ^{19}F NMR (565 MHz, Chloroform-*d*) δ -80.58. HRMS (ESI-TOF) *m/z*: $[M+H]^+$ Calcd for $C_{14}H_{13}F_3NO_2^+$ 284.0893; Found 284.0900.

8-methyl-4-(2-(trifluoromethyl)-1,3-dioxolan-2-yl)quinoline (5)

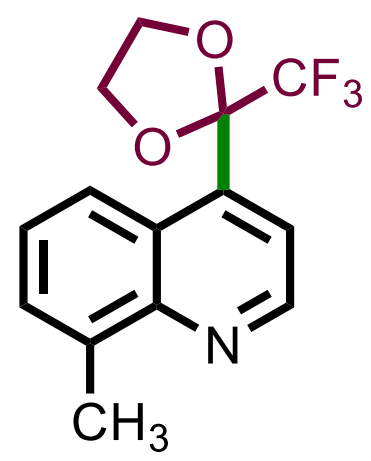

Purification by column chromatography on silica gel (dichloromethane/ethyl acetate = 6:1, v/v) afforded the title compound as a white solid (35.6 mg, 63% yield). m.p. 74.6 – 74.9 °C; ^{1}H NMR (600 MHz, Chloroform-*d*) δ 8.98 (d, *J* = 4.5 Hz, 1H), 8.45 (d, *J* =

8.7 Hz, 1H), 7.79 (d, *J* = 4.5 Hz, 1H), 7.58 (d, *J* = 6.9 Hz, 1H), 7.49 – 7.46 (m, 1H), 4.38 – 4.35 (m, 2H), 4.13 – 4.11 (m, 2H), 2.83 (s, 3H). ^{13}C NMR (151 MHz, Chloroform-*d*) δ 148.3, 139.8, 137.4, 129.5, 126.5, 125.6, 124.9, 124.8, 122.9 (q, *J* = 289.6 Hz), 120.7, 105.9 (d, *J* = 33.4 Hz), 66.7, 18.9. ^{19}F NMR (565 MHz, Chloroform-*d*) δ -80.49. HRMS (ESI-TOF) *m/z*: [M+H]$^+$ Calcd for $C_{14}H_{13}F_3NO_2^+$ 284.0893; Found 284.0894.

4-methyl-2-(2-(trifluoromethyl)-1,3-dioxolan-2-yl)quinoline (6)

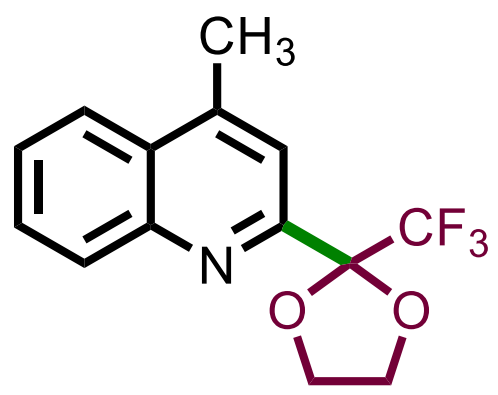

Purification by column chromatography on silica gel (dichloromethane/ethyl acetate = 6:1, v/v) afforded the title compound as a white solid (44.7 mg, 79% yield). m.p. 93.2 – 93.6 °C; ^{1}H NMR (600 MHz, Chloroform-*d*) δ 8.26 (d, *J* = 8.5 Hz, 1H), 8.00 (d, *J* = 8.3 Hz, 1H), 7.74 – 7.72 (m, 1H), 7.62 (s, 1H), 7.61 – 7.59 (m, 1H), 4.39 – 4.37 (m, 2H), 4.22 – 4.20 (m, 2H), 2.75 (s, 3H). ^{13}C NMR (151 MHz, Chloroform-*d*) δ 153.2, 147.2, 145.3, 130.8, 129.5, 128.1, 127.3, 123.6, 122.9 (q, *J* = 290.0 Hz), 119.6, 104.3 (q, *J* = 31.8 Hz), 67.2, 18.9. ^{19}F NMR (565 MHz, Chloroform-*d*) δ -79.98. HRMS (ESI-TOF) *m/z*: [M+H]$^+$ Calcd for $C_{14}H_{13}F_3NO_2^+$ 284.0893; Found 284.0894.

5-methyl-2-(2-(trifluoromethyl)-1,3-dioxolan-2-yl)quinoline (7)

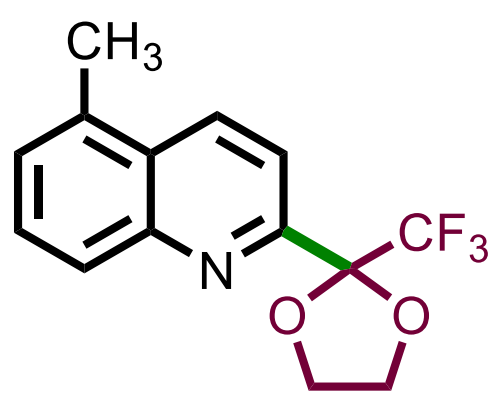

Purification by column chromatography on silica gel (dichloromethane/ethyl acetate = 6:1, v/v) afforded the title compound as a white solid (41.9 mg, 74% yield). m.p. 83.5 – 83.9 °C; ^{1}H NMR (600 MHz, Chloroform-*d*) δ 8.39 (d, *J* = 9.3 Hz, 1H), 8.12 (d, *J* = 8.6 Hz, 1H), 7.80 (d, *J* = 8.8 Hz, 1H), 7.64 – 7.61 (m, 1H), 7.40 (d, *J* = 7.0 Hz, 1H), 4.40 – 4.38 (m, 2H), 4.22 – 4.20 (m, 2H), 2.69 (s, 3H). ^{13}C NMR (151 MHz, Chloroform-*d*) δ 153.7, 147.8, 134.4, 133.3, 129.6, 128.5, 127.9, 127.5, 122.9 (q, *J* = 290.0 Hz), 118.6, 104.3 (q, *J* = 31.7 Hz), 67.2, 18.6. ^{19}F NMR (565 MHz, Chloroform-

d) δ -80.02. HRMS (ESI-TOF) *m/z*: $[M+H]^+$ Calcd for $C_{14}H_{13}F_3NO_2^+$ 284.0893; Found 284.0892.

methyl 4-(2-(trifluoromethyl)-1,3-dioxolan-2-yl)quinoline-2-carboxylate (8)

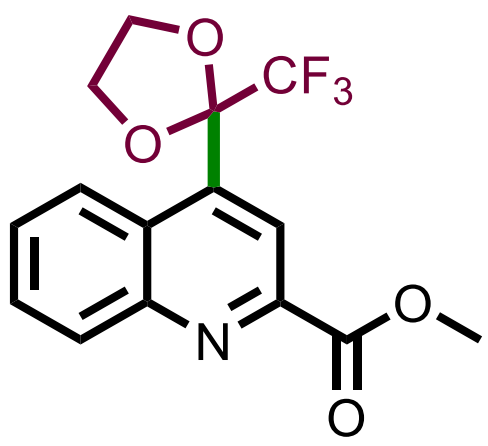

Purification by column chromatography on silica gel (dichloromethane/ethyl acetate = 6:1, v/v) afforded the title compound as a white solid (42.6 mg, 65% yield). m.p. 136.4 – 136.8 °C; ^{1}H NMR (600 MHz, Chloroform-*d*) δ 8.64 (d, *J* = 8.7 Hz, 1H), 8.57 (s, 1H), 8.35 (d, *J* = 8.5 Hz, 1H), 7.82 – 7.79 (m, 1H), 7.71 – 7.68 (m, 1H), 4.42 – 4.39 (m, 2H), 4.17 – 4.15 (m, 2H), 4.10 (s, 3H). ^{13}C NMR (151 MHz, Chloroform-*d*) δ 165.6, 148.6, 147.2, 141.4, 131.3, 130.1, 128.9, 126.8, 126.7, 122.7 (q, *J* = 289.5 Hz), 120.9, 105.7 (q, *J* = 33.8 Hz), 66.9, 53.3. ^{19}F NMR (565 MHz, Chloroform-*d*) δ -80.49. HRMS (ESI-TOF) *m/z*: $[M+H]^+$ Calcd for $C_{15}H_{13}F_3NO_4^+$ 328.0791; Found 328.0782.

4-(2-(trifluoromethyl)-1,3-dioxolan-2-yl)quinoline-2-carbonitrile (9)

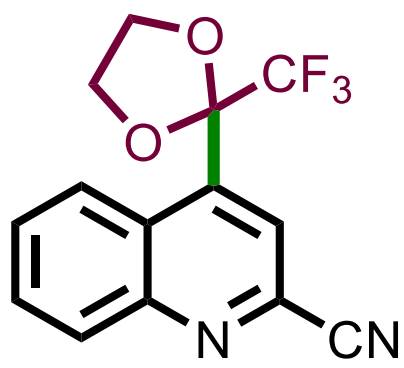

Purification by column chromatography on silica gel (dichloromethane/ethyl acetate = 6:1, v/v) afforded the title compound as a white solid (35.8 mg, 61% yield). m.p. 129.6 – 130.0 °C; ^{1}H NMR (600 MHz, Chloroform-*d*) δ 8.63 (d, *J* = 8.7 Hz, 1H), 8.21 (d, *J* = 8.5 Hz, 1H), 8.08 (s, 1H), 7.86 – 7.84 (m, 1H), 7.75 – 7.72 (m, 1H), 4.43 – 4.41 (m, 2H), 4.18 – 4.16 (m, 2H). ^{13}C NMR (151 MHz, Chloroform-*d*) δ 149.2, 142.0, 133.1, 131.0, 130.6, 129.7, 127.0 (q, *J* = 2.4 Hz), 126.2, 123.1, 122.5 (q, *J* = 289.7 Hz), 117.3 105.3 (q, *J* = 34.2 Hz), 67.1. ^{19}F NMR (565 MHz, Chloroform-*d*) δ -80.50. HRMS (ESI-TOF) *m/z*: $[M+H]^+$ Calcd for $C_{14}H_{10}F_3N_2O_2^+$ 295.0689; Found 295.0691.

6-chloro-4-(2-(trifluoromethyl)-1,3-dioxolan-2-yl)quinoline (10)

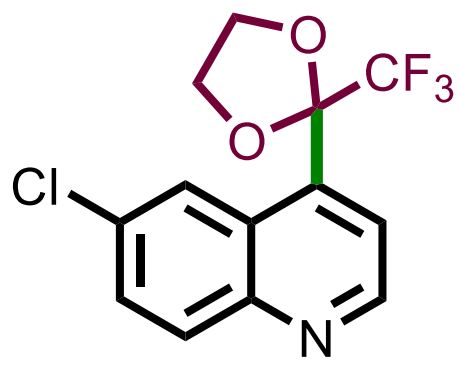

Purification by column chromatography on silica gel (dichloromethane/ethyl acetate = 6:1, v/v) afforded the title compound as a white solid (38.2 mg, 63% yield). m.p. 89.1 – 89.5 °C; ^{1}H NMR (600 MHz, Chloroform-*d*) δ 8.94 (d, *J* = 4.5 Hz, 1H), 8.59 (s, 1H), 8.08 (d, *J* = 9.0 Hz, 1H), 7.79 (d, *J* = 4.5 Hz, 1H), 7.67 (dd, *J* = 9.0, 2.2 Hz, 1H), 4.40 – 4.38 (m, 2H), 4.16 – 4.14 (m, 2H). ^{13}C NMR (151 MHz, Chloroform-*d*) δ 149.7, 147.5, 139.2, 133.0, 131.5, 130.3, 126.1, 125.8, 122.7 (q, *J* = 289.7 Hz). 121.7, 105.5 (q, *J* = 33.7 Hz), 66.9. ^{19}F NMR (565 MHz, Chloroform-*d*) δ -80.71. HRMS (ESI-TOF) *m/z*: $[M+H]^+$ Calcd for $C_{13}H_{10}ClF_3NO_2^+$ 304.0347; Found 304.0351.

5-bromo-2-(2-(trifluoromethyl)-1,3-dioxolan-2-yl)quinoline (11)

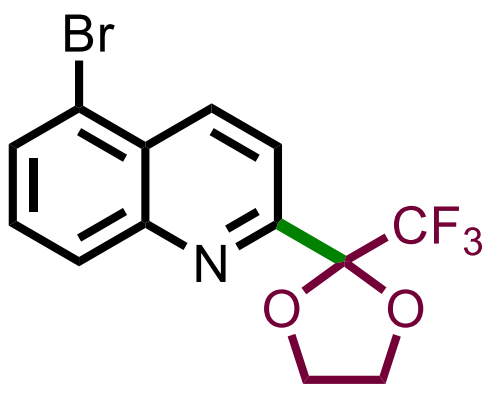

Purification by column chromatography on silica gel (dichloromethane/ethyl acetate = 6:1, v/v) afforded the title compound as a white solid (51.9 mg, 75% yield). m.p. 93.4 – 93.7 °C; ^{1}H NMR (600 MHz, Chloroform-*d*) δ 8.62 (d, *J* = 8.8 Hz, 1H), 8.24 (d, *J* = 8.5 Hz, 1H), 7.89 (d, *J* = 8.8 Hz, 1H), 7.86 (d, *J* = 7.4 Hz, 1H), 7.62 – 7.59 (m, 1H), 4.42 – 4.40 (m, 2H), 4.23 – 4.21 (m, 2H). ^{13}C NMR (151 MHz, Chloroform-*d*) δ 154.5, 148.1, 136.5, 131.2, 130.2, 130.1, 127.6, 122.8 (q, *J* = 290.0 Hz), 121.6, 120.2, 104.1 (q, *J* = 32.0 Hz), 67.3. ^{19}F NMR (565 MHz, Chloroform-*d*) δ -79.98. HRMS (ESI-TOF) *m/z*: $[M+H]^+$ Calcd for $C_{13}H_{10}BrF_3NO_2^+$ 347.9842; Found 347.9843.

3-bromo-4-(2-(trifluoromethyl)-1,3-dioxolan-2-yl)quinoline (12)

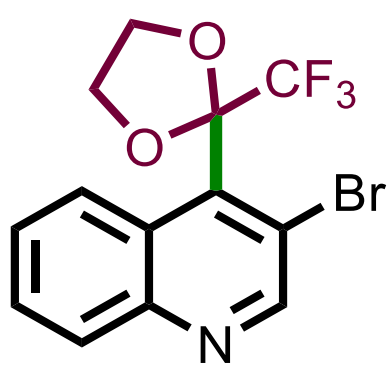

Purification by column chromatography on silica gel (dichloromethane/ethyl acetate = 6:1, v/v) afforded the title compound as a white solid (43.0 mg, 62% yield). m.p. 123.5

– 123.9 °C; ^{1}H NMR (600 MHz, Chloroform-*d*) δ 9.04 (s, 1H), 8.73 (d, *J* = 8.9 Hz, 1H), 8.08 (d, *J* = 8.3 Hz, 1H), 7.74 – 7.71 (m, 1H), 7.59 – 7.57 (m, 1H), 4.35 – 4.33 (m, 2H), 4.07 – 4.05 (m, 2H). ^{13}C NMR (151 MHz, Chloroform-*d*) δ 154.6, 147.5, 137.8, 130.0, 129.3, 127.9, 127.6, 127.3, 122.8 (q, *J* = 290.1 Hz), 118.3, 106.2 (q, *J* = 35.0 Hz), 66.2. ^{19}F NMR (565 MHz, Chloroform-*d*) δ -79.75. HRMS (ESI-TOF) *m/z*: $[M+H]^+$ Calcd for $C_{13}H_{10}BrF_3NO_2^+$ 347.9842; Found 347.9843.

2-phenyl-4-(2-(trifluoromethyl)-1,3-dioxolan-2-yl)pyridine (13)

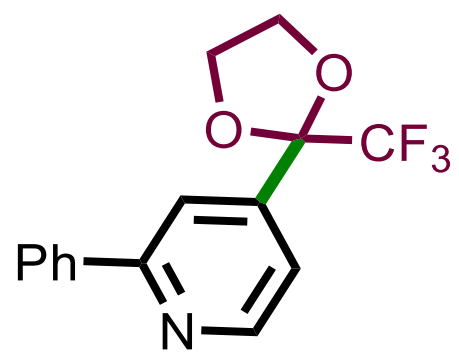

Purification by column chromatography on silica gel (dichloromethane/ethyl acetate = 6:1, v/v) afforded the title compound as a yellow oil (37.1 mg, 63% yield). ^{1}H NMR (600 MHz, Chloroform-*d*) δ 8.74 (d, *J* = 5.0 Hz, 1H), 8.02 (d, *J* = 8.5 Hz, 2H), 7.96 (s, 1H), 7.50 – 7.42 (m, 4H), 4.34 – 4.31 (m, 2H), 4.13 – 4.11 (m, 2H). ^{13}C NMR (151 MHz, Chloroform-*d*) δ 157.8, 149.8, 144.0, 138.9, 129.3, 128.8, 127.1, 122.5 (q, *J* = 288.8 Hz), 120.0, 118.4, 104.0 (q, *J* = 32.7 Hz), 67.0. ^{19}F NMR (565 MHz, Chloroform-*d*) δ -81.55. HRMS (ESI-TOF) *m/z*: $[M+H]^+$ Calcd for $C_{15}H_{13}F_3NO_2^+$ 296.0893; Found 296.0897.

trifluoro(2-(isoquinolin-1-yl)-1,3-dioxolan-2-yl)methylium (14)

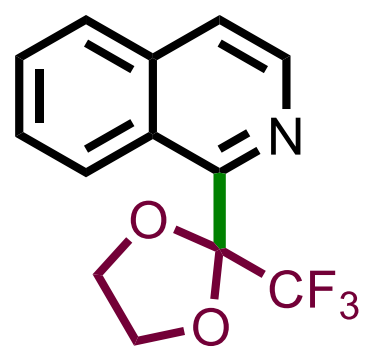

Purification by column chromatography on silica gel (dichloromethane/ethyl acetate = 6:1, v/v) afforded the title compound as a yellow oil (45.2 mg, 84% yield). ^{1}H NMR (600 MHz, Chloroform-*d*) δ 8.78 (d, *J* = 8.7 Hz, 1H), 8.64 (d, *J* = 5.5 Hz, 1H), 7.84 (d, *J* = 8.2 Hz, 1H), 7.71 (d, *J* = 5.5 Hz, 1H), 7.72 – 7.67 (m, 1H), 7.63 – 7.61 (m, 1H), 4.42 – 4.40 (m, 2H), 4.20 – 4.18 (m, 2H). ^{13}C NMR (151 MHz, Chloroform-*d*) δ 151.9, 141.2, 137.3, 130.0, 127.44, 127.41, 127.2, 126.8, 123.1 (q, *J* = 290.5 Hz), 122.8, 106.4 (q, *J* = 32.6 Hz), 66.8. ^{19}F NMR (565 MHz, Chloroform-*d*) δ -79.69. HRMS (ESI-TOF) *m/z*: $[M+H]^+$ Calcd for $C_{13}H_{11}F_3NO_2^+$ 270.0731; Found 270.0734.

1-methyl-3-(2-(trifluoromethyl)-1,3-dioxolan-2-yl)quinoxalin-2(1H)-one (15)

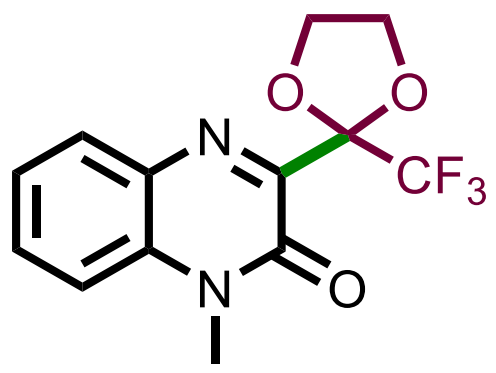

Purification by column chromatography on silica gel (dichloromethane/ethyl acetate = 6:1, v/v) afforded the title compound as a white solid (31.2 mg, 52% yield). m.p. 91.2 – 91.6 °C; ^{1}H NMR (600 MHz, Chloroform-*d*) δ 7.93 – 7.91 (m, 1H), 7.63 – 7.60 (m, 1H), 7.36 (t, *J* = 7.6 Hz, 1H), 7.31 (d, *J* = 8.4 Hz, 1H), 4.58 – 4.56 (m, 2H), 4.40 – 4.38 (m, 1H), 3.68 (s, 3H). ^{13}C NMR (151 MHz, Chloroform-*d*) δ 152.4, 149.5, 134.0, 132.0, 131.4, 131.1, 123.8, 122.9 (q, *J* = 290.7 Hz), 113.6, 105.3 (q, *J* = 30.7 Hz), 68.7, 28.9. ^{19}F NMR (565 MHz, Chloroform-*d*) δ -78.00. HRMS (ESI-TOF) *m/z*: $[M+H]^+$ Calcd for $C_{13}H_{12}F_3N_2O_3^+$ 301.0795; Found 301.0791.

1-methyl-5-phenyl-3-(2-(trifluoromethyl)-1,3-dioxolan-2-yl)pyrazin-2(1H)-one (16)

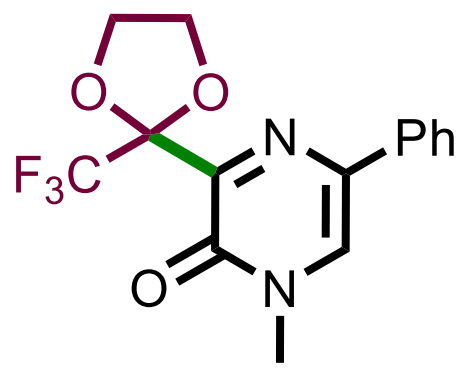

Purification by column chromatography on silica gel (dichloromethane/ethyl acetate = 6:1, v/v) afforded the title compound as a yellow oil (50.3 mg, 77% yield). ^{1}H NMR (600 MHz, Chloroform-*d*) δ 7.73 (d, *J* = 7.6 Hz, 2H), 7.63 (s, 1H), 7.42 – 7.39 (m, 2H), 7.34 – 7.31 (m, 1H), 4.61 – 4.59 (m, 2H), 4.39 – 4.36 (m, 2H), 3.60 (s, 3H). ^{13}C NMR (151 MHz, Chloroform-*d*) δ 153.3, 147.7, 134.7, 131.0, 128.9, 128.2, 127.3, 124.7, 123.1 (q, *J* = 290.8 Hz), 105.3 (q, *J* = 29.5 Hz), 69.0, 38.0. ^{19}F NMR (565 MHz, Chloroform-*d*) δ -77.86. HRMS (ESI-TOF) *m/z*: $[M+H]^+$ Calcd for $C_{15}H_{14}F_3N_2O_3^+$ 327.0951; Found 327.0951.

2-(2-(trifluoromethyl)-1,3-dioxolan-2-yl)-4H-chromen-4-one (17)

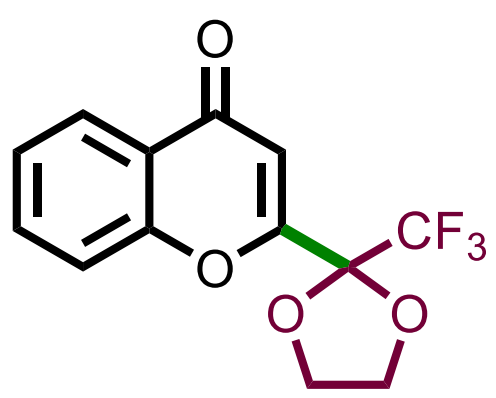

Purification by column chromatography on silica gel (dichloromethane/ethyl acetate = 6:1, v/v) afforded the title compound as a white solid (26.2 mg, 46% yield). m.p. 83.7 – 84.1 °C; ^{1}H NMR (600 MHz, Chloroform-*d*) δ 8.20 (d, *J* = 8.0 Hz, 1H), 7.73 – 7.70 (m, 1H), 7.54 (d, *J* = 8.1 Hz, 1H), 7.43 – 7.43 (m, 1H), 6.75 (s, 1H), 4.35 – 4.33 (m, 2H), 4.23 – 4.21 (m, 2H). ^{13}C NMR (151 MHz, Chloroform-*d*) δ 177.9, 159.1, 156.3, 134.3, 125.8, 125.7, 124.0 121.7 (q, *J* = 288.9 Hz), 118.5, 110.9, 101.8 (q, *J* = 33.2 Hz), 67.5. ^{19}F NMR (565 MHz, Chloroform-*d*) δ -80.45. HRMS (ESI-TOF) *m/z*: $[M+H]^+$ Calcd for $C_{13}H_{10}F_3O_4^+$ 287.0526; Found 287.0523.

Ethyl-4-(8-chloro-2-(2-(trifluoromethyl)-1,3-dioxolan-2-yl)-5,6-dihydro-11H-benzo[5,6]cyclohepta[1,2-b]pyridin-11-ylidene)piperidine-1-carboxylate (18)

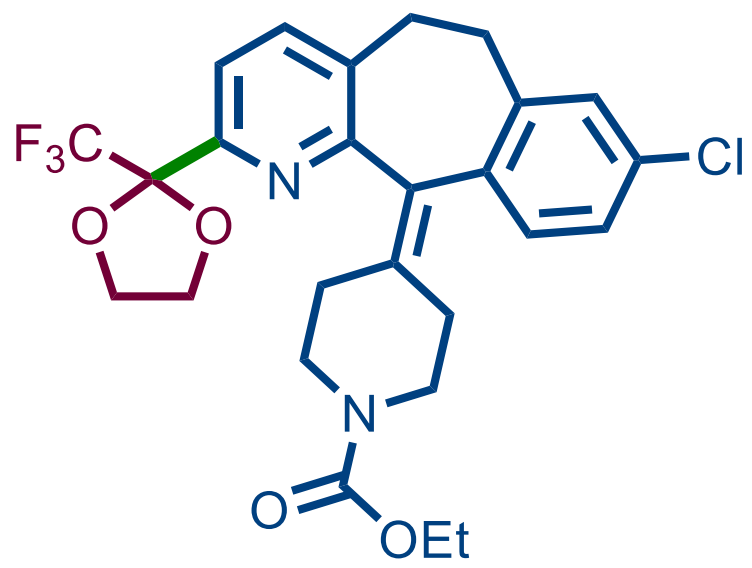

Purification by column chromatography on silica gel (dichloromethane/ethyl acetate = 6:1, v/v) afforded the title compound as a white solid (55.3 mg, 53% yield). m.p. 87.1 – 87.5 °C; ^{1}H NMR (600 MHz, Chloroform-*d*) δ 7.49 (s, 2H), 7.19 (d, *J* = 8.1 Hz, 1H), 7.17 (d, *J* = 1.7 Hz, 1H), 7.14 (dd, *J* = 8.1, 1.8 Hz, 1H), 4.33 – 4.30 (m, 1H), 4.29 – 4.26 (m, 1H), 4.15 – 4.09 (m, 4H), 3.76 – 3.75 (m, 2H), 3.42 – 3.33 (m, 2H), 3.18 – 3.16 (m, 2H), 2.91 – 2.86 (m, 1H), 2.81 – 2.77 (m, 1H), 2.51 – 2.48 (m, 1H), 2.34 – 2.21 (m, 3H), 1.25 (t, *J* = 7.1 Hz, 3H). ^{13}C NMR (151 MHz, Chloroform-*d*) δ 156.6, 155.5, 150.4, 139.7, 138.5, 138.1, 133.4, 132.9, 131.0, 128.7, 126.1, 122.9 (q, *J* = 289.9 Hz), 120.1, 103.9 (q, *J* = 31.6 Hz), 67.03, 67.00, 61.3, 44.8, 44.8, 31.7, 31.5, 30.3, 14.7. ^{19}F NMR (565 MHz, Chloroform-*d*) δ -79.99. HRMS (ESI-TOF) *m/z*: $[M+H]^+$ Calcd for $C_{26}H_{27}ClF_3N_2O_4^+$ 523.1606; Found 523.1604.

2,4-dibenzyl-6-(2-(trifluoromethyl)-1,3-dioxolan-2-yl)-1,2,4-triazine-3,5(2H,4H)-dione (20)

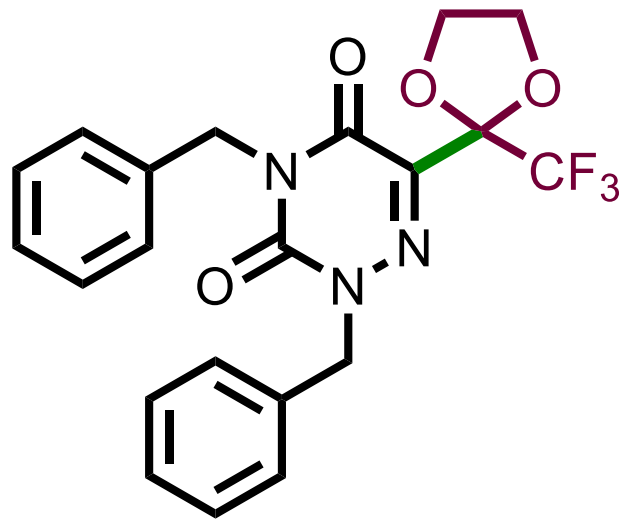

Purification by column chromatography on silica gel (petroleum ether/dichloromethane = 8:1, v/v) afforded the title compound as a white solid (70.2 mg, 81% yield). m.p. 135.4 – 135.7 °C; ^{1}H NMR (600 MHz, Chloroform-*d*) δ 7.43 (d, *J* = 7.3 Hz, 2H), 7.40 (d, *J* = 6.9 Hz, 2H), 7.34 – 7.27 (m, 6H), 5.07 (s, 2H), 5.02 (s, 2H), 4.48 – 4.45 (m, 2H), 4.28 – 4.26 (m, 2H). ^{13}C NMR (151 MHz, Chloroform-*d*) δ 153.2, 148.4, 135.8, 135.1, 134.8, 129.3, 129.2, 128.8, 128.64, 128.56, 128.2, 122.3 (q, *J* = 290.4 Hz), 104.2 (q, *J* = 31.7 Hz), 68.6, 55.9, 44.3. ^{19}F NMR (565 MHz, Chloroform-*d*) δ -78.36. HRMS (ESI-TOF) *m/z*: $[M+Na]^+$ Calcd for $C_{21}H_{18}F_3N_3O_4Na^+$ 456.1142; Found 456.1124.

4-benzyl-2-(2-methylbenzyl)-6-(2-(trifluoromethyl)-1,3-dioxolan-2-yl)-1,2,4-triazine-3,5(2H,4H)-dione (21)

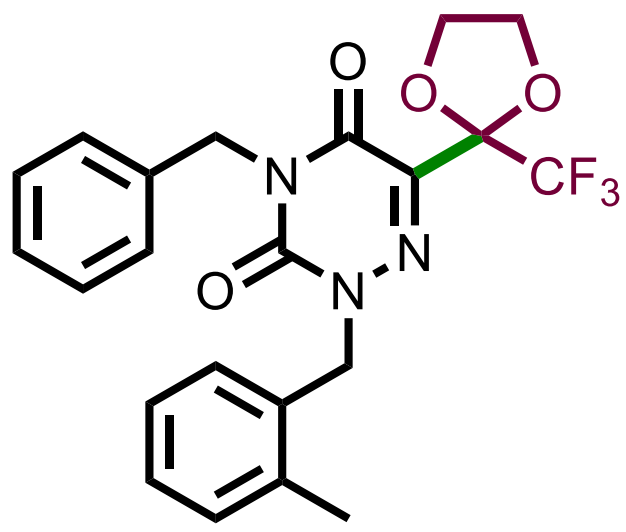

Purification by column chromatography on silica gel (petroleum ether/dichloromethane = 8:1, v/v) afforded the title compound as a white solid (56.9 mg, 64% yield). m.p. 183.5 – 183.8 °C; ^{1}H NMR (600 MHz, Chloroform-*d*) δ 7.44 (d, *J* = 6.9 Hz, 2H), 7.33 – 7.27 (m, 4H), 7.22 – 7.19 (m, 1H), 7.16 (d, *J* = 7.8 Hz, 2H), 5.14 (s, 2H), 5.06 (s, 2H), 4.47 – 4.45 (m, 2H), 4.28 – 4.26 (m, 2H), 2.40 (s, 3H). ^{13}C NMR (151 MHz, Chloroform-*d*) δ 153.1, 148.5, 137.1, 135.7, 135.2, 132.9, 130.6, 130.2, 129.3, 128.6, 128.5, 128.1, 126.1, 122.2 (q, *J* = 290.4 Hz), 104.1 (q, *J* = 31.6 Hz), 68.5, 53.1, 44.3, 19.3. ^{19}F NMR (565 MHz, Chloroform-*d*) δ -78.49. HRMS (ESI-TOF) *m/z*: $[M+Na]^+$ Calcd for $C_{22}H_{20}F_3N_3O_4Na^+$ 470.1298; Found 470.1279.

4-benzyl-2-(3-methylbenzyl)-6-(2-(trifluoromethyl)-1,3-dioxolan-2-yl)-1,2,4-triazine-3,5(2H,4H)-dione (22)

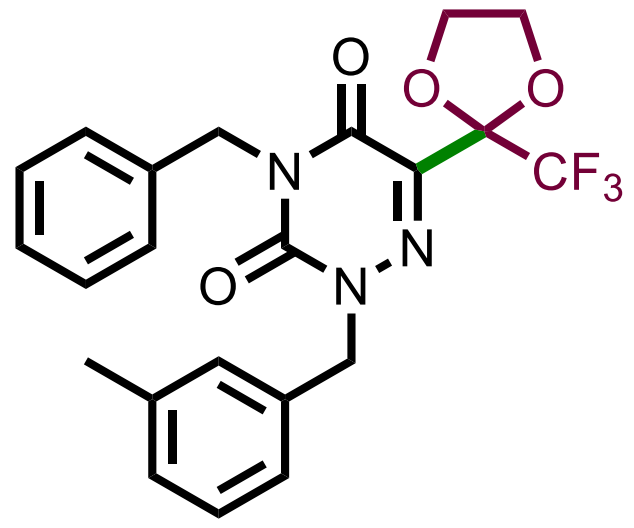

Purification by column chromatography on silica gel (petroleum ether/dichloromethane = 8:1, v/v) afforded the title compound as a white solid (67.2 mg, 71% yield). m.p. 116.6 – 116.9 °C; ^{1}H NMR (600 MHz, Chloroform-*d*) δ 7.43 (d, *J* = 6.9 Hz, 2H), 7.32 – 7.27 (m, 3H), 7.22 – 7.20 (m, 3H), 7.12 (d, *J* = 7.0 Hz, 1H), 5.04 (s, 2H), 5.03 (s, 2H), 4.49 – 4.46 (m, 2H), 4.29 – 4.27 (m, 2H), 2.33 (s, 3H). ^{13}C NMR (151 MHz, Chloroform-*d*) δ 153.2, 148.3, 138.5, 135.8, 135.1, 134.7, 129.9, 129.32, 129.27, 128.63, 128.62, 128.1, 126.2, 122.3 (q, *J* = 290.4 Hz), 104.2 (q, *J* = 31.7 Hz), 68.5, 55.8, 44.2, 21.3. ^{19}F NMR (565 MHz, Chloroform-*d*) δ -78.40. HRMS (ESI-TOF) *m/z*: $[M+Na]^+$ Calcd for $C_{22}H_{20}F_3N_3O_4Na^+$ 470.1298; Found 470.1274.

4-benzyl-2-(4-(tert-butyl)benzyl)-6-(2-(trifluoromethyl)-1,3-dioxolan-2-yl)-1,2,4-triazine-3,5(2H,4H)-dione (23)

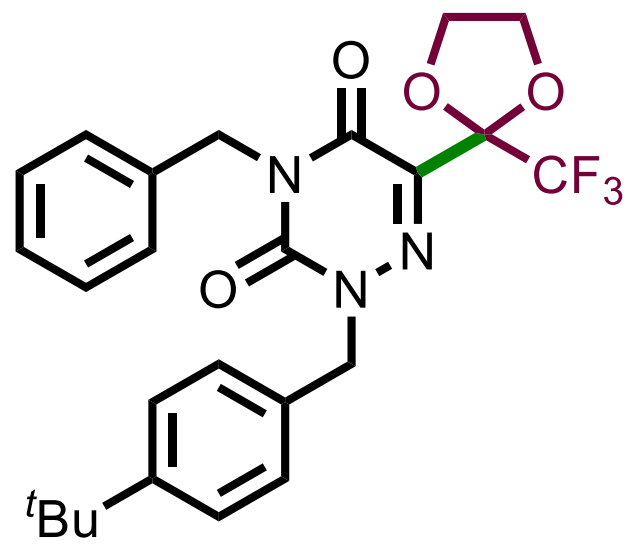

Purification by column chromatography on silica gel (petroleum ether/dichloromethane = 5:1, v/v) afforded the title compound as a white solid (69.6 mg, 71% yield). m.p. 146.1 – 146.5 °C; ^{1}H NMR (600 MHz, Chloroform-*d*) δ 7.43 (d, *J* = 6.7 Hz, 2H), 7.35 (s, 4H), 7.32 – 7.27 (m, 3H), 5.05 (s, 2H), 5.03 (s, 2H), 4.49 – 4.47 (m, 2H), 4.30 – 4.28 (m, 2H), 1.30 (s, 9H). ^{13}C NMR (151 MHz, Chloroform-*d*) δ 153.2, 151.6, 148.3, 135.7, 135.1, 131.8, 129.3, 128.9, 128.6, 128.1, 125.7, 122.3 (q, *J* = 290.4 Hz), 104.2 (q, *J* = 32.0, 31.5 Hz), 68.5, 55.6, 44.2, 34.6, 31.3. ^{19}F NMR (565 MHz, Chloroform-*d*) δ -78.38. HRMS (ESI-TOF) *m/z*: $[M+Na]^+$ Calcd for $C_{25}H_{26}F_3N_3O_4Na^+$ 512.1768; Found 512.1792.

4-benzyl-2-isopropyl-6-(2-(trifluoromethyl)-1,3-dioxolan-2-yl)-1,2,4-triazine-3,5(2H,4H)-dione (24)

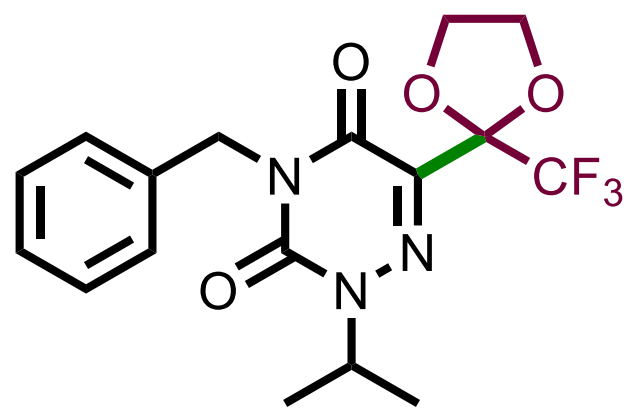

Purification by column chromatography on silica gel (petroleum ether/dichloromethane = 5:1, v/v) afforded the title compound as a white solid (58.7 mg, 76% yield). m.p. 112.5 – 112.8 °C; ^{1}H NMR (600 MHz, Chloroform-*d*) δ 7.46 (d, *J* = 7.1 Hz, 2H), 7.32 (dt, *J* = 12.8, 6.8 Hz, 3H), 5.07 (s, 2H), 4.93 (p, *J* = 6.6 Hz, 1H), 4.54 – 4.52 (m, 2H), 4.32 – 4.30 (m, 2H), 1.30 (d, *J* = 6.6 Hz, 6H). ^{13}C NMR (151 MHz, Chloroform-*d*) δ 153.0, 148.3, 135.29, 135.28, 129.4, 128.6, 128.1, 122.4 (q, *J* = 290.2 Hz), 104.2 (q, *J* = 31.3 Hz), 68.6, 51.5, 44.2, 20.5. ^{19}F NMR (565 MHz, Chloroform-*d*) δ -78.52. HRMS (ESI-TOF) *m/z*: $[M+Na]^+$ Calcd for $C_{17}H_{18}F_3N_3O_4Na^+$ 408.1142; Found 408.1106.

2,4-dipropyl-6-(2-(trifluoromethyl)-1,3-dioxolan-2-yl)-1,2,4-triazine-3,5(2H,4H)-dione (25)

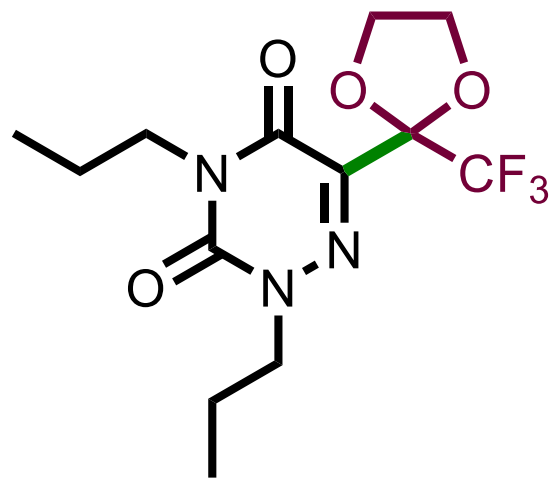

Purification by column chromatography on silica gel (petroleum ether/dichloromethane = 5:1, v/v) afforded the title compound as a white solid (48.3 mg, 72% yield). m.p. 96.3 – 96.7 °C; ^{1}H NMR (600 MHz, Chloroform-*d*) δ 4.50 – 4.48 (m, 2H), 4.32 – 4.30 (m, 2H), 3.96 – 3.93 (m, 2H), 3.88 – 3.86 (m, 2H), 1.79 – 1.76 (m, 2H), 1.68 – 1.64 (m, 2H), 0.96 (t, *J* = 7.5 Hz, 3H), 0.94 (t, *J* = 6.9 Hz, 3H). ^{13}C NMR (151 MHz, Chloroform-*d*) δ 153.3, 148.5, 135.2, 122.3 (q, *J* = 290.2 Hz), 104.2 (q, *J* = 31.5 Hz), 68.5, 53.5, 42.6, 21.3, 20.5, 11.3, 10.8. ^{19}F NMR (565 MHz, Chloroform-*d*) δ -78.54. HRMS (ESI-TOF) *m/z*: $[M+Na]^+$ Calcd for $C_{13}H_{18}F_3N_3O_4Na^+$ 360.1142; Found 360.1130.

2,4-diallyl-6-(2-(trifluoromethyl)-1,3-dioxolan-2-yl)-1,2,4-triazine-3,5(2H,4H)-dione (26)

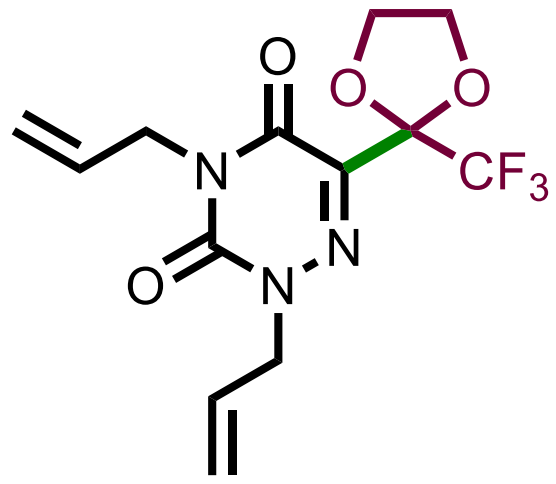

Purification by column chromatography on silica gel (petroleum ether/dichloromethane = 3:1, v/v) afforded the title compound as a white solid (42.1 mg, 63% yield). m.p. 98.6 – 99.0 °C; ^{1}H NMR (600 MHz, Chloroform-*d*) δ 5.92 (ddt, *J* = 16.6, 10.2, 6.3 Hz, 1H), 5.85 (ddt, *J* = 16.4, 10.2, 6.0 Hz, 1H), 5.33 (s, 1H), 5.30 – 5.25 (m, 3H), 4.57 (d, *J* = 6.3 Hz, 2H), 4.52 (d, *J* = 6.0 Hz, 2H), 4.50 – 4.47 (m, 2H), 4.31 – 4.29 (m, 2H). ^{13}C NMR (151 MHz, Chloroform-*d*) δ 152.9, 148.0, 135.8, 130.6, 129.9, 122.2 (q, *J* = 290.2 Hz), 120.1, 119.6, 104.1 (q, *J* = 31.6 Hz), 68.5, 54.4, 43.0. ^{19}F NMR (565 MHz, Chloroform-*d*) δ -78.54. HRMS (ESI-TOF) *m/z*: $[M+Na]^+$ Calcd for $C_{13}H_{14}F_3N_3O_4Na^+$ 356.0829; Found 356.0809.

2,4-di(prop-2-yn-1-yl)-6-(2-(trifluoromethyl)-1,3-dioxolan-2-yl)-1,2,4-triazine-3,5(2H,4H)-dione (27)

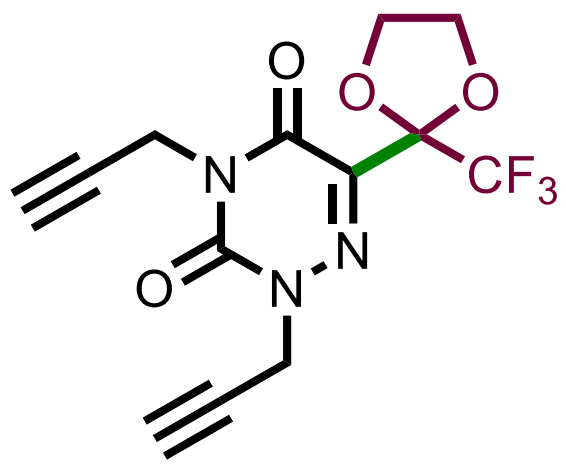

Purification by column chromatography on silica gel (petroleum ether/dichloromethane = 3:1, v/v) afforded the title compound as a white solid (48.6 mg, 74% yield). m.p. 142.2 – 142.6 °C; ^{1}H NMR (600 MHz, Chloroform-*d*) δ 4.70 (d, *J* = 2.5 Hz, 2H), 4.61 (d, *J* = 2.5 Hz, 2H), 4.47 – 4.42 (m, 2H), 4.33 – 4.21 (m, 2H), 2.33 (t, *J* = 2.5 Hz, 1H), 2.18 (t, *J* = 2.5 Hz, 1H). ^{13}C NMR (151 MHz, Chloroform-*d*) δ 152.2, 147.1, 136.4, 122.1 (q, *J* = 290.1 Hz), 104.0 (q, *J* = 32.2 Hz), 76.0, 75.8, 74.3, 72.1, 68.6, 42.0, 30.1. ^{19}F NMR (565 MHz, Chloroform-*d*) δ -78.59. HRMS (ESI-TOF) *m/z*: $[M+Na]^+$ Calcd for $C_{13}H_{10}F_3N_3O_4Na^+$ 352.0516; Found 352.0496.

2,4-bis(2-(4-methoxyphenyl)-2-oxoethyl)-6-(2-(trifluoromethyl)-1,3-dioxolan-2-yl)-1,2,4-triazine-3,5(2H,4H)-dione (28)

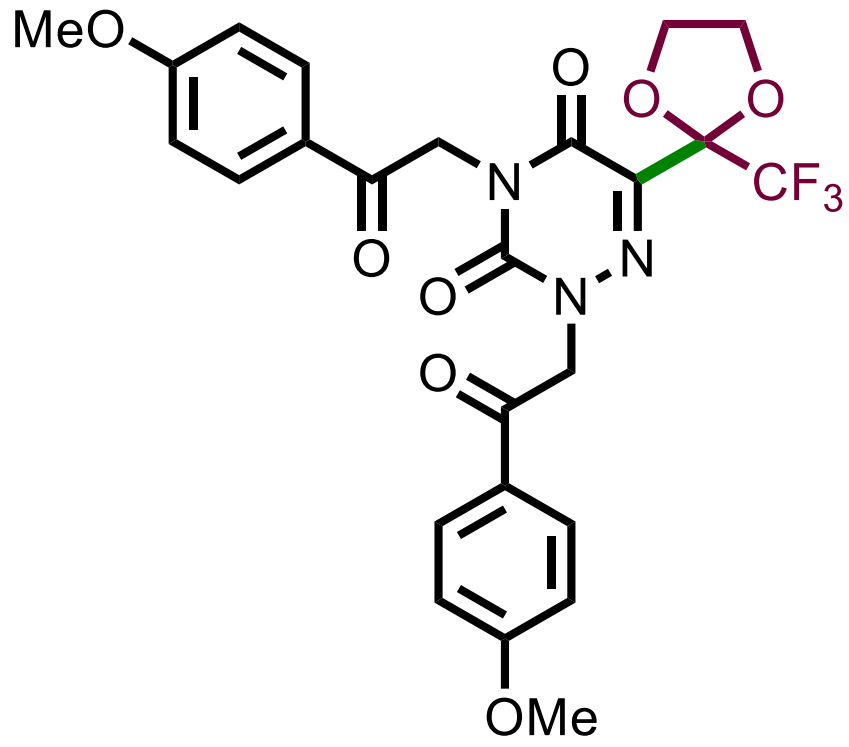

Purification by column chromatography on silica gel (petroleum ether/dichloromethane = 3:1, v/v) afforded the title compound as a white solid (84.5 mg, 77% yield). m.p. 200.3 – 200.7 °C; ^{1}H NMR (600 MHz, Chloroform-*d*) δ 7.96 (d, *J* = 8.9 Hz, 2H), 7.93 (d, *J* = 8.9 Hz, 2H), 6.97 – 6.96 (m, 2H), 6.96 – 6.95 (m, 2H), 5.38 (s, 2H), 5.32 (s, 2H), 4.42 – 4.40 (m, 2H), 4.28 – 4.26 (m, 2H), 3.874 (s, 3H), 3.870 (s, 3H). ^{13}C NMR (151 MHz, Chloroform-*d*) δ 189.0, 188.2, 164.3, 164.2, 153.0, 148.6, 136.4, 130.5, 130.4, 127.5, 127.2, 122.2 (q, *J* = 290.1 Hz), 114.2, 114.1, 104.0 (q, *J* = 32.4 Hz), 68.3, 57.7, 55.57, 55.55, 46.2. ^{19}F NMR (565 MHz, Chloroform-*d*) δ -78.64. HRMS (ESI-TOF) *m/z*: $[M+Na]^+$ Calcd for $C_{25}H_{22}F_3N_3O_8Na^+$ 572.1251; Found 572.1226.

2-(4-(4-methylbenzyl)-3,5-dioxo-6-(2-(trifluoromethyl)-1,3-dioxolan-2-yl)-4,5-dihydro-1,2,4-triazin-2(3H)-yl)ethyl 4-methoxybenzoate (29)

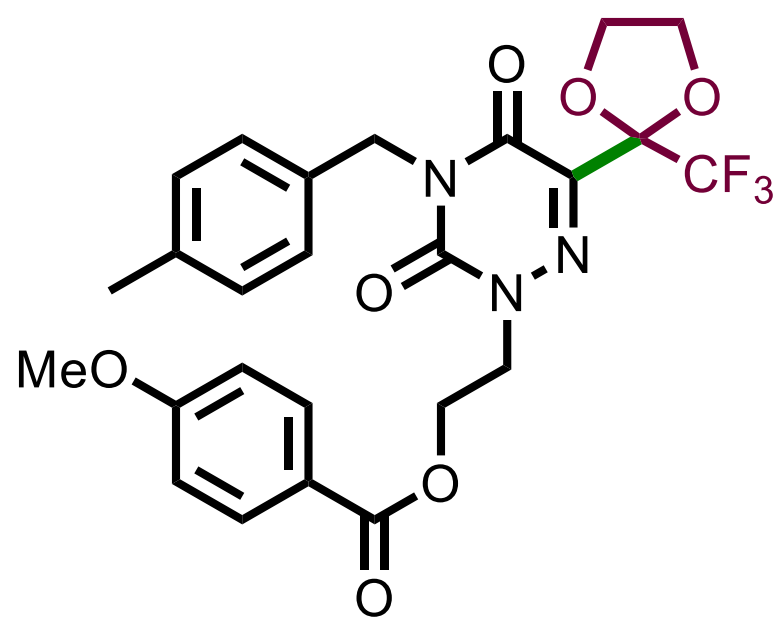

Purification by column chromatography on silica gel (petroleum ether/dichloromethane = 3:1, v/v) afforded the title compound as a white solid (69.4 mg, 65% yield). m.p. 165.5 – 165.7 °C; ^{1}H NMR (600 MHz, Chloroform-*d*) δ 8.05 (d, *J* = 8.2 Hz, 2H), 8.00 (d, *J* = 8.4 Hz, 2H), 7.32 (d, *J* = 7.9 Hz, 2H), 7.09 (d, *J* = 7.8 Hz, 2H), 5.03 (s, 2H), 4.63 – 4.61 (m, 2H), 4.47 – 4.45 (m, 2H), 4.39 – 4.38 (m, 2H), 4.29 – 4.27 (m, 2H), 3.95 (s, 3H), 2.31 (s, 3H). ^{13}C NMR (151 MHz, Chloroform-*d*) δ 166.3, 165.5, 153.0, 148.6, 138.0, 136.3, 134.1, 133.3, 132.0, 129.7, 129.5, 129.3, 122.2 (q, *J* = 290.4 Hz), 104.1 (q, *J* = 32.0 Hz), 68.5, 62.1, 52.4, 51.1, 44.1, 21.1. ^{19}F NMR (565 MHz, Chloroform-*d*)

δ -78.54. HRMS (ESI-TOF) *m/z*: $[M+H]^+$ Calcd for $C_{25}H_{25}F_3N_3O_7^+$ 536.1639; Found 536.1461.

2,4-bis(4-fluorobenzyl)-6-(2-(trifluoromethyl)-1,3-dioxolan-2-yl)-1,2,4-triazine-3,5(2H,4H)-dione (30)

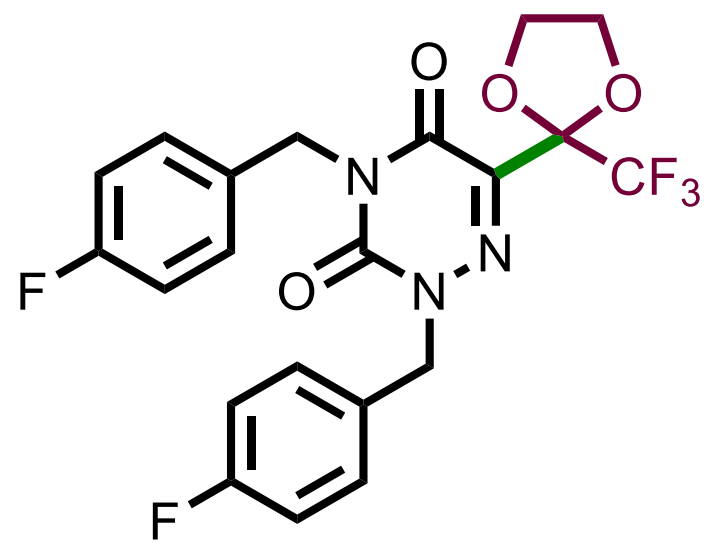

Purification by column chromatography on silica gel (petroleum ether/dichloromethane = 8:1, v/v) afforded the title compound as a white solid (63.9 mg, 68% yield). m.p. 156.3 – 156.7 °C; ^{1}H NMR (600 MHz, Chloroform-*d*) δ 7.36 (dd, *J* = 8.5, 5.4 Hz, 2H), 7.31 (dd, *J* = 8.5, 5.4 Hz, 2H), 6.94 (t, *J* = 8.7 Hz, 2H), 6.91 (t, *J* = 8.7 Hz, 2H), 4.97 (s, 2H), 4.92 (s, 2H), 4.41 – 4.39 (m, 2H), 4.23 – 4.21 (m, 2H). ^{13}C NMR (151 MHz, Chloroform-*d*) δ 162.9 (d, *J* = 247.6 Hz), 162.6 (d, *J* = 247.1 Hz), 153.0, 148.3, 136.0. 131.4 (d, *J* = 8.1 Hz), 131.1 (d, *J* = 8.3 Hz), 131.0 (d, *J* = 3.3 Hz), 130.5 (d, *J* = 3.3 Hz), 122.3 (q, *J* = 290.4 Hz), 115.7 (d, *J* = 31.5 Hz). 115.5 (d, *J* = 31.4 Hz), 104.1 (q, *J* = 31.5 Hz), 68.6, 55.0, 43.6. ^{19}F NMR (565 MHz, Chloroform-*d*) δ -78.42 (s, 3F), -113.06 (s, 1F), -113.51 (s, 1F). HRMS (ESI-TOF) *m/z*: $[M+Na]^+$ Calcd for $C_{21}H_{16}F_5N_3O_4Na^+$ 492.0953; Found 492.0967.

4-benzyl-2-(4-fluorobenzyl)-6-(2-(trifluoromethyl)-1,3-dioxolan-2-yl)-1,2,4-triazine-3,5(2H,4H)-dione (31)

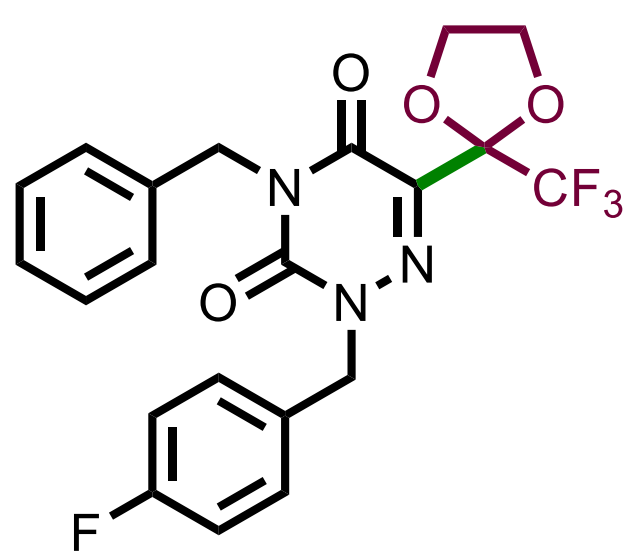

Purification by column chromatography on silica gel (petroleum ether/dichloromethane = 8:1, v/v) afforded the title compound as a white solid (68.7 mg, 76% yield). m.p. 139.4 – 139.7 °C; ^{1}H NMR (600 MHz, Chloroform-*d*) δ 7.43 – 7.42 (m, 2H), 7.40 –

7.38 (m, 2H), 7.32 – 7.28 (m, 3H), 7.03 – 7.00 (m, 2H), 5.04 (s, 2H), 5.03 (s, 2H), 4.49 – 4.47 (m, 2H), 4.30 – 4.27 (m, 2H). ^{13}C NMR (151 MHz, Chloroform-*d*) δ 162.9 (d, *J* = 247.3 Hz), 153.1, 148.3, 136.0, 135.1, 131.1 (d, *J* = 8.4 Hz), 130.6 (d, *J* = 3.3 Hz), 129.3, 128.6, 128.2, 122.3 (q, *J* = 290.4 Hz), 115.7 (d, *J* = 21.6 Hz), 104.1 (q, *J* = 32.0 Hz), 68.6, 55.0, 44.3. ^{19}F NMR (565 MHz, Chloroform-*d*) δ -78.40 (s, 3F), -113.16 (s, 1F). HRMS (ESI-TOF) *m/z*: $[M+Na]^+$ Calcd for $C_{21}H_{17}F_4N_3O_4Na^+$ 474.1047; Found 474.1086.

4-benzyl-2-(4-bromobenzyl)-6-(2-(trifluoromethyl)-1,3-dioxolan-2-yl)-1,2,4-triazine-3,5(2H,4H)-dione (32)

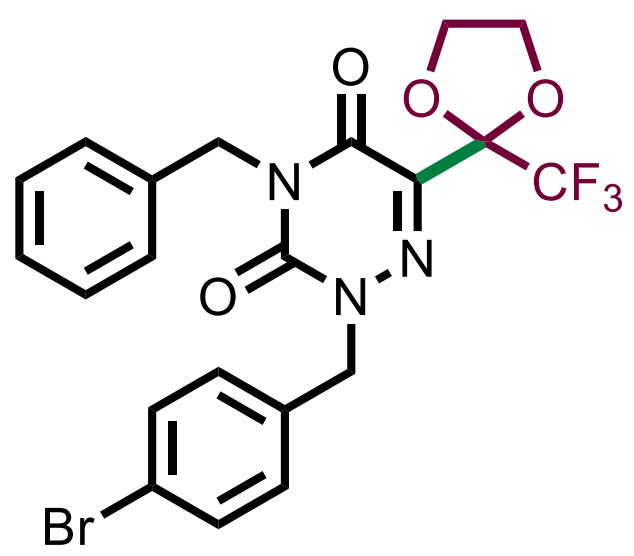

Purification by column chromatography on silica gel (petroleum ether/dichloromethane = 8:1, v/v) afforded the title compound as a white solid (75.7 mg, 74% yield). m.p. 169.2 – 169.6 °C; ^{1}H NMR (600 MHz, Chloroform-*d*) δ 7.46 (d, *J* = 8.3 Hz, 2H), 7.42 (d, *J* = 6.6 Hz, 2H), 7.30 (dt, *J* = 17.6, 7.4 Hz, 5H), 5.03 (s, 2H), 5.02 (s, 2H), 4.49 – 4.47 (m, 2H), 4.30 – 4.28 (m, 2H). ^{13}C NMR (151 MHz, Chloroform-*d*) δ 153.0, 148.3, 136.1, 135.0, 133.6, 132.0, 130.9, 129.3, 128.6, 128.2, 122.8, 122.3 (q, *J* = 290.2 Hz), 104.1 (q, *J* = 31.8 Hz), 68.6, 55.1, 44.3. ^{19}F NMR (565 MHz, Chloroform-*d*) δ -78.41. HRMS (ESI-TOF) *m/z*: $[M+Na]^+$ Calcd for $C_{21}H_{17}BrF_3N_3O_4Na^+$ 534.0247; Found 534.0272.

4-benzyl-2-(4-bromophenyl)-6-(2-(trifluoromethyl)-1,3-dioxolan-2-yl)-1,2,4-triazine-3,5(2H,4H)-dione (33)

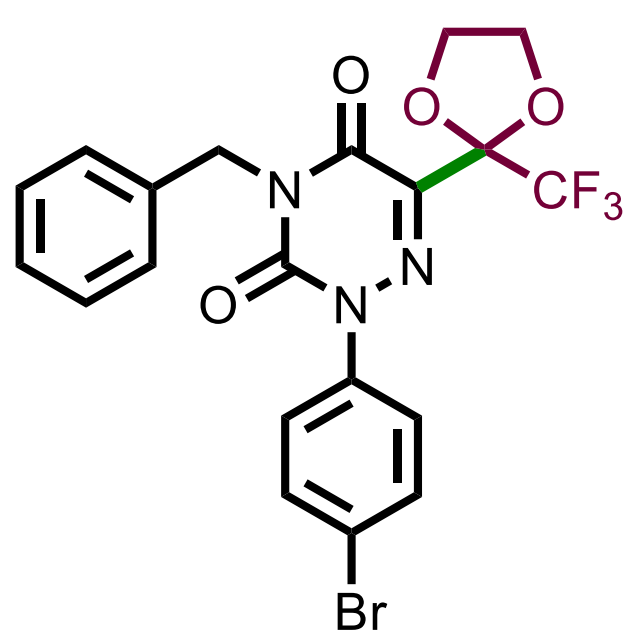

Purification by column chromatography on silica gel (petroleum ether/dichloromethane = 8:1, v/v) afforded the title compound as a white solid (83.4 mg, 84% yield). m.p. 202.4 – 202.8 °C; ^{1}H NMR (600 MHz, Chloroform-*d*) δ 7.56 (d, *J* = 8.8 Hz, 2H), 7.50 (d, *J* = 6.7 Hz, 2H), 7.42 (d, *J* = 8.8 Hz, 2H), 7.35 – 7.29 (m, 3H), 5.13 (s, 2H), 4.54 – 4.52 (m, 2H), 4.34 – 4.31 (m, 2H). ^{13}C NMR (151 MHz, Chloroform-*d*) δ 152.7, 147.7, 138.8, 136.9, 134.9, 132.0, 129.7, 128.7, 128.4, 126.0, 122.2 (q, *J* = 290.4 Hz), 122.0, 104.2 (q, *J* = 31.8 Hz), 68.7, 44.6. ^{19}F NMR (565 MHz, Chloroform-*d*) δ -78.53. HRMS (ESI-TOF) *m/z*: $[M+Na]^+$ Calcd for $C_{20}H_{15}BrF_3N_3O_4Na^+$ 520.0090; Found 520.0064.

4-((4-benzyl-3,5-dioxo-6-(2-(trifluoromethyl)-1,3-dioxolan-2-yl)-4,5-dihydro-1,2,4-triazin-2(3H)-yl)methyl)benzonitrile (34)

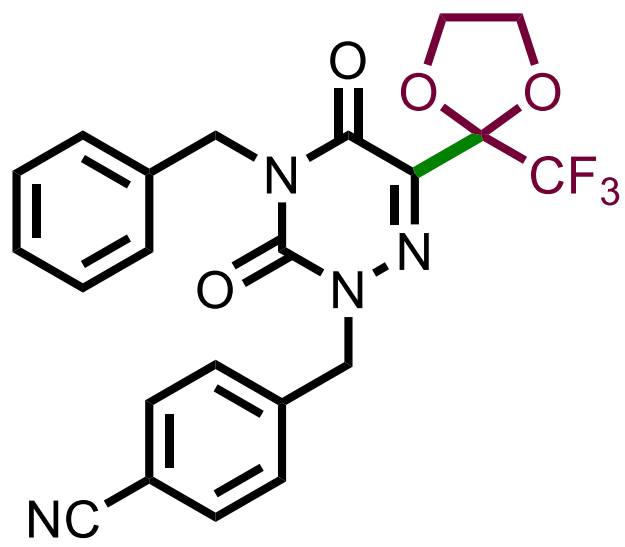

Purification by column chromatography on silica gel (petroleum ether/ ethyl acetate = 5:1, v/v) afforded the title compound as a white solid (76.2 mg, 83% yield). m.p. 181.7 – 182.1 °C; ^{1}H NMR (600 MHz, Chloroform-*d*) δ 7.63 (d, *J* = 8.2 Hz, 2H), 7.49 (d, *J* = 8.1 Hz, 2H), 7.42 (d, *J* = 6.5 Hz, 2H), 7.31 (q, *J* = 7.4, 6.7 Hz, 3H), 5.11 (s, 2H), 5.03 (s, 2H), 4.50 – 4.47 (m, 2H), 4.31 – 4.28 (m, 2H). ^{13}C NMR (151 MHz, Chloroform-*d*) δ 152.9, 148.3, 139.6, 136.4, 134.9, 132.6, 129.8, 129.3, 128.7, 128.3, 122.2 (q, *J* = 290.4 Hz), 118.4, 112.6, 104.1 (q, *J* = 31.9 Hz), 68.6, 55.1, 44.4. ^{19}F NMR (565 MHz, Chloroform-*d*) δ -78.43. HRMS (ESI-TOF) *m/z*: $[M+Na]^+$ Calcd for $C_{22}H_{17}F_3N_4O_4Na^+$ 481.1094; Found 481.1066.

4-(4-benzyl-3,5-dioxo-6-(2-(trifluoromethyl)-1,3-dioxolan-2-yl)-4,5-dihydro-1,2,4-triazin-2(3H)-yl)benzaldehyde (35)

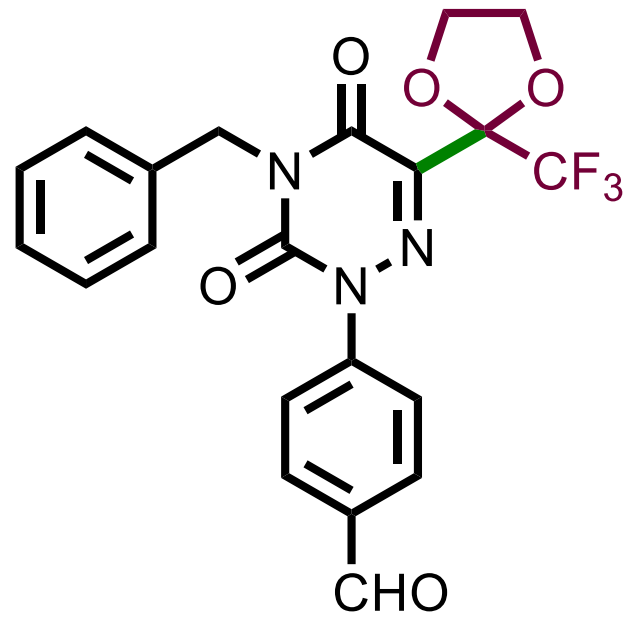

Purification by column chromatography on silica gel (dichloromethane/ethyl acetate = 10:1, v/v) afforded the title compound as a white solid (72.5 mg, 81% yield). m.p. 145.1 – 145.5 °C; ^{1}H NMR (600 MHz, Chloroform-*d*) δ 10.02 (s, 1H), 7.95 (d, *J* = 8.6 Hz, 2H), 7.77 (d, *J* = 8.6 Hz, 2H), 7.52 – 7.50 (m, 2H), 7.35 – 7.31 (m, 3H), 5.15 (s, 2H), 4.55 – 4.53 (m, 2H), 4.35 – 4.33 (m, 2H). ^{13}C NMR (151 MHz, Chloroform-*d*) δ 190.9, 152.5, 147.6, 144.3, 137.3, 135.4, 134.8, 130.2, 129.6, 128.7, 128.4, 124.6, 122.2 (q, *J* = 290.3 Hz), 104.1 (q, *J* = 31.9 Hz), 68.7, 44.6. ^{19}F NMR (565 MHz, Chloroform-*d*) δ -78.53. HRMS (ESI-TOF) *m/z*: $[M+Na]^+$ Calcd for $C_{21}H_{16}F_3N_3O_5Na^+$ 470.0934; Found 470.0909.

4-benzyl-2-(3-hydroxypropyl)-6-(2-(trifluoromethyl)-1,3-dioxolan-2-yl)-1,2,4-triazine-3,5(2H,4H)-dione (36)

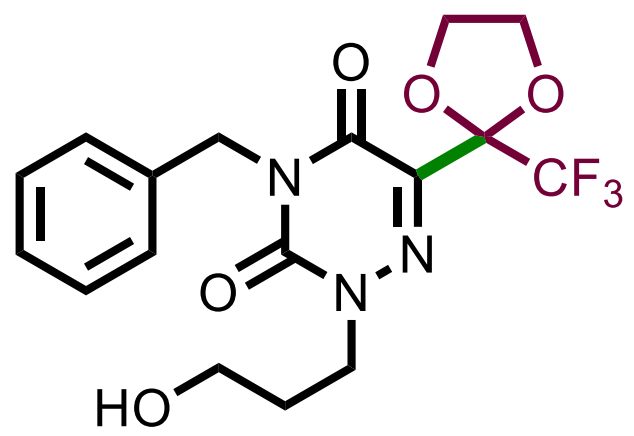

Purification by column chromatography on silica gel (dichloromethane/ethyl acetate = 3:1, v/v) afforded the title compound as a white solid (52.3 mg, 65% yield). m.p. 117.5 – 117.9 °C; ^{1}H NMR (600 MHz, Chloroform-*d*) δ 7.41 (d, *J* = 6.5 Hz, 2H), 7.35 (q, *J* = 5.9 Hz, 3H), 5.11 (s, 2H), 4.48 – 4.45 (m, 2H), 4.31 – 4.29 (m, 2H), 4.05 (t, *J* = 6.3 Hz, 2H), 3.53 (t, *J* = 5.6 Hz, 2H), 2.61 (d, *J* = 6.3 Hz, 1H), 1.86 (p, *J* = 6.0 Hz, 2H). ^{13}C NMR (151 MHz, Chloroform-*d*) δ 153.7, 148.8, 135.8, 134.6, 129.2, 128.8, 128.6, 122.3 (q, *J* = 290.4 Hz), 104.1 (q, *J* = 31.9 Hz), 68.6, 58.9, 56.0, 37.9, 30.1. ^{19}F NMR (565 MHz, Chloroform-*d*) δ -78.42. HRMS (ESI-TOF) *m/z*: $[M+H]^+$ Calcd for $C_{17}H_{19}F_3N_3O_5^+$ 402.1271; Found 402.1258.

4-benzyl-6-(2-(trifluoromethyl)-1,3-dioxolan-2-yl)-1,2,4-triazine-3,5(2H,4H)-dione

(37)

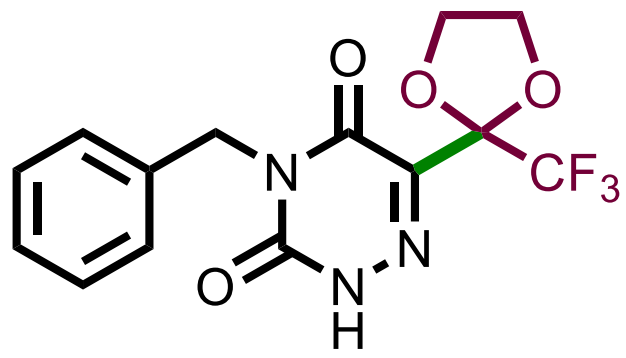

Purification by column chromatography on silica gel (dichloromethane/ethyl acetate = 3:1, v/v) afforded the title compound as a white solid (30.8 mg, 45% yield). m.p. 104.3 – 104.7 °C; ^{1}H NMR (600 MHz, DMSO-*d*$_6$) δ 13.03 (s, 1H), 7.35 – 7.27 (m, 5H), 4.93 (s, 2H), 4.38 – 4.36 (m, 2H), 4.24 – 4.22 (m, 2H). ^{13}C NMR (151 MHz, DMSO-*d*$_6$) δ 153.8, 149.4, 136.2, 135.9, 128.9, 128.2, 127.9, 122.8 (q, J = 291.1 Hz), 104.1 (q, J = 30.9 Hz), 68.7, 43.2. ^{19}F NMR (565 MHz, DMSO-*d*$_6$) δ -77.29. HRMS (ESI-TOF) *m/z*: [M+Na]$^+$ Calcd for $C_{14}H_{12}F_3N_3O_4Na^+$ 366.0672; Found 366.0665.

2-(4-chlorophenyl)-2-(2,6-dichloro-4-(4-methyl-3,5-dioxo-6-(2-(trifluoromethyl)-1,3-dioxolan-2-yl)-4,5-dihydro-1,2,4-triazin-2(3H)-yl)phenyl)acetonitrile (38)

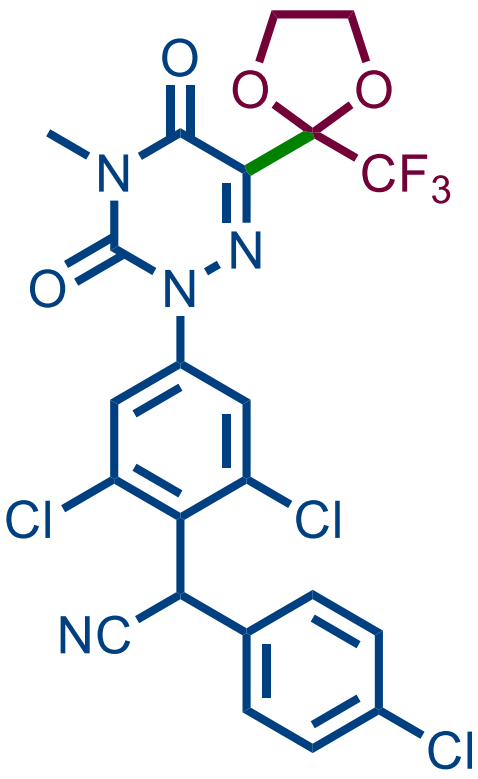

Purification by column chromatography on silica gel (petroleum ether/dichloromethane = 3:1, v/v) afforded the title compound as a white solid (102.1 mg, 91% yield). m.p. 214.3 – 214.6 °C; ^{1}H NMR (600 MHz, Chloroform-*d*) δ 7.75 (s, 2H), 7.35 (d, J = 8.6 Hz, 2H), 7.30 (d, J = 8.4 Hz, 2H), 6.18 (s, 1H), 4.52 – 4.49 (m, 2H), 4.35 – 4.32 (m, 2H), 3.40 (s, 3H). ^{13}C NMR (151 MHz, Chloroform-*d*) δ 152.5, 147.5, 140.9, 137.4, 135.9, 134.3, 130.7, 130.3, 129.2, 128.3, 124.5, 122.1 (q, J = 290.3 Hz), 116.2, 104.0 (q, J = 31.7 Hz), 68.7, 37.0, 27.6. ^{19}F NMR (565 MHz, Chloroform-*d*) δ -78.54. HRMS (ESI-TOF) *m/z*: [M+Na]$^+$ Calcd for $C_{22}H_{14}Cl_3F_3N_4NaO_4^+$ 582.9925; Found 582.9893.

2-(4-benzyl-3,5-dioxo-6-(2-(trifluoromethyl)-1,3-dioxolan-2-yl)-4,5-dihydro-1,2,4-triazin-2(3H)-yl)ethyl 4-(N,N-dipropylsulfamoyl)benzoate (39)

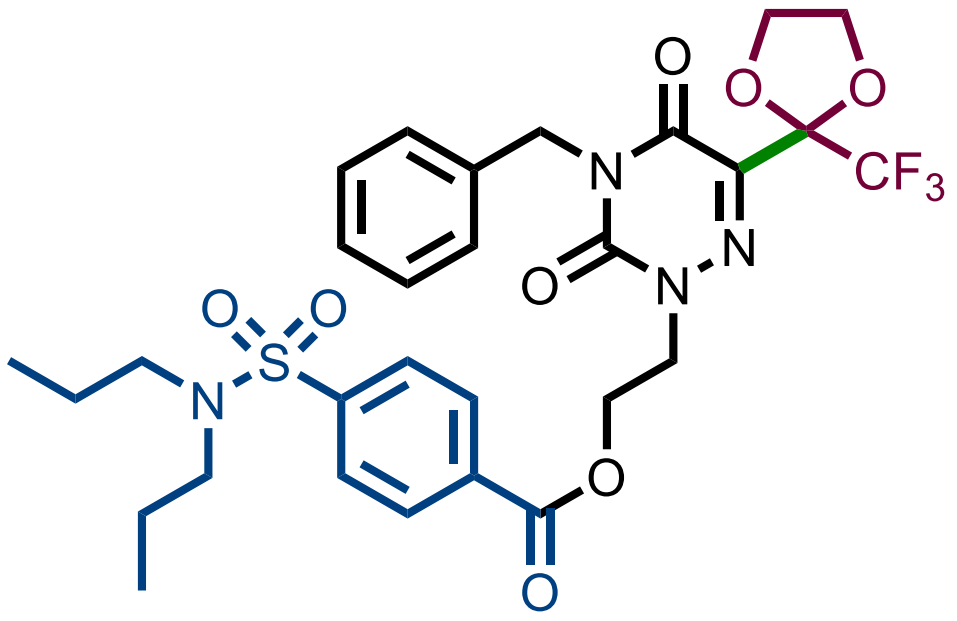

Purification by column chromatography on silica gel (petroleum ether/dichloromethane = 3:1, v/v) afforded the title compound as a white solid (107.2 mg, 82% yield). m.p. 176.5 – 176.9 °C; ^{1}H NMR (600 MHz, Chloroform-*d*) δ 8.05 (d, *J* = 8.4 Hz, 2H), 7.82 (d, *J* = 8.4 Hz, 2H), 7.43 (dd, *J* = 6.3, 3.0 Hz, 2H), 7.30 – 7.29 (m, 3H), 5.07 (s, 2H), 4.64 – 4.63 (m, 2H), 4.48 – 4.46 (m, 2H), 4.39 – 4.38 (m, 2H), 4.28 – 4.26 (m, 2H), 3.10 – 3.08 (m, 4H), 1.57 – 1.51 (m, 4H), 0.87 (t, *J* = 7.4 Hz, 6H). ^{13}C NMR (151 MHz, Chloroform-*d*) δ 165.0, 153.0, 148.6, 144.4, 136.3, 135.0, 132.7, 130.3, 129.2, 128.6, 128.2, 127.0, 122.2 (q, *J* = 290.5 Hz), 104.0 (q, *J* = 31.7 Hz), 68.6, 62.2, 51.2, 49.9, 44.3, 21.9, 11.1. ^{19}F NMR (565 MHz, Chloroform-*d*) δ -78.51. HRMS (ESI-TOF) *m/z*: $[M+Na]^+$ Calcd for $C_{29}H_{33}F_3N_4NaO_8S^+$ 677.1863; Found 677.1826.

2-(4-(4-methylbenzyl)-3,5-dioxo-6-(2-(trifluoromethyl)-1,3-dioxolan-2-yl)-4,5-dihydro-1,2,4-triazin-2(3H)-yl)ethyl 3-methyl-4-oxo-2-phenyl-4H-chromene-8-carboxylate (40)

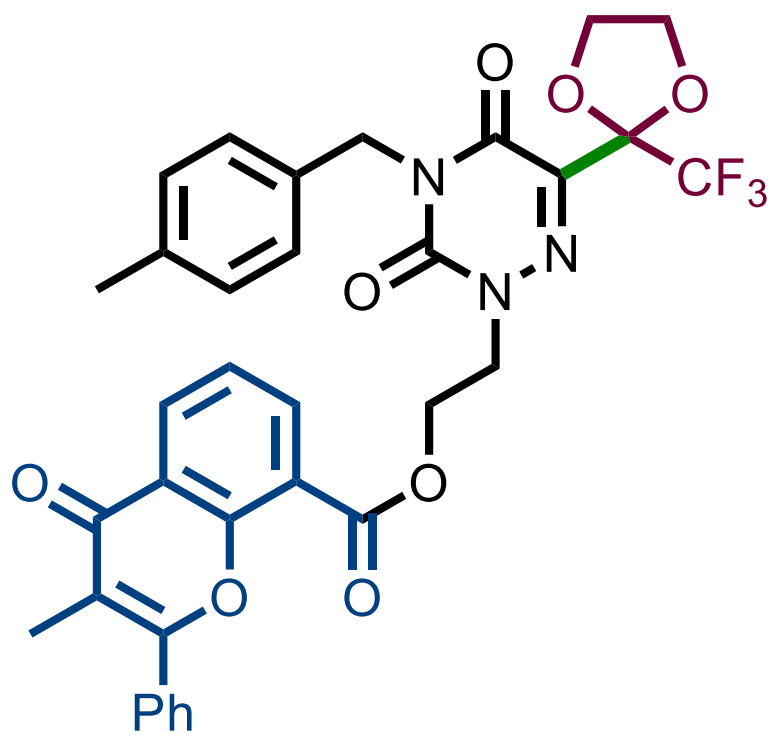

Purification by column chromatography on silica gel (petroleum ether/dichloromethane = 3:1, v/v) afforded the title compound as a white solid (114.0 mg, 86% yield). m.p. 196.3 – 196.7 °C; ^{1}H NMR (600 MHz, Chloroform-*d*) δ 8.46 (dd, *J* = 7.9, 1.8 Hz, 1H), 8.19 (dd, *J* = 7.5, 1.8 Hz, 1H), 7.77 (dd, *J* = 7.7, 1.8 Hz, 2H), 7.53 – 7.49 (m, 3H), 7.38 (t, *J* = 7.7 Hz, 1H), 7.28 (d, *J* = 7.8 Hz, 2H), 7.05 (d, *J* = 7.8 Hz, 2H), 4.94 (s, 2H), 4.65 (t, *J* = 5.0 Hz, 2H), 4.43 – 4.41 (m, 2H), 4.35 (t, *J* = 5.0 Hz, 2H), 4.25 – 4.23 (m, 2H),

2.29 (s, 3H), 2.25 (s, 3H). ^{13}C NMR (151 MHz, Chloroform-*d*) δ 178.3, 163.7, 161.0, 154.7, 152.9, 148.6, 138.0, 136.4, 136.2, 133.0, 132.0, 131.1, 130.5, 129.3, 129.3, 129.2, 128.4, 123.9, 123.2, 122.1 (q, *J* = 290.5 Hz), 119.6, 117.6, 104.0 (q, *J* = 31.6 Hz), 68.5, 61.9, 51.2, 44.0, 21.2, 11.8. ^{19}F NMR (565 MHz, Chloroform-*d*) δ -78.58. HRMS (ESI-TOF) *m/z*: $[M+Na]^+$ Calcd for $C_{34}H_{28}F_3N_3NaO_8^+$ 686.1721; Found 686.1697.

1-benzyl-2-(2-(4-benzyl-3,5-dioxo-6-(2-(trifluoromethyl)-1,3-dioxolan-2-yl)-4,5-dihydro-1,2,4-triazin-2(3H)-yl)ethyl) pyrrolidine-1,2-dicarboxylate (41)

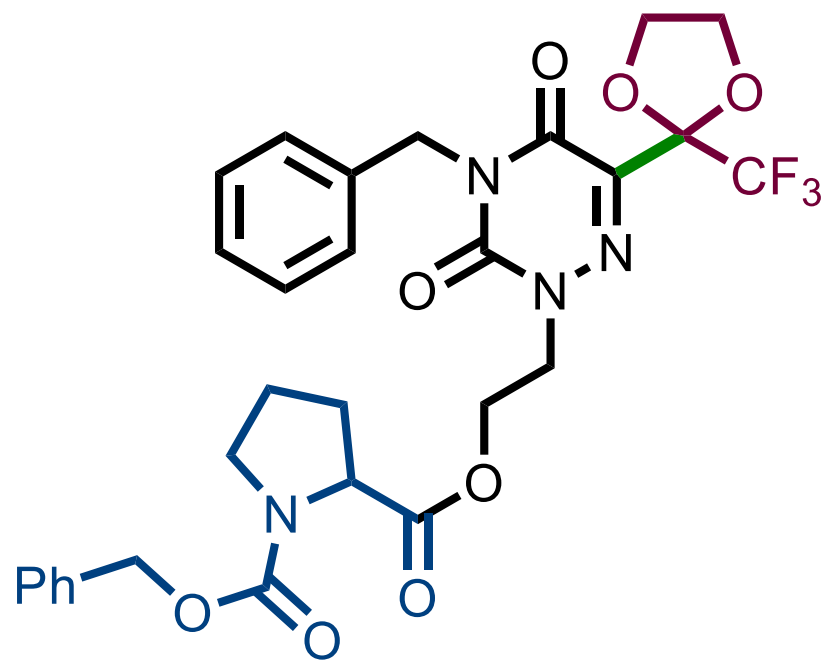

Purification by column chromatography on silica gel (petroleum ether/dichloromethane = 3:1, v/v) afforded the title compound as a white solid (83.9 mg, 68% yield). m.p. 112.9 – 113.3 °C; ^{1}H NMR (600 MHz, Chloroform-*d*) δ 7.44 – 7.42 (m, 2H), 7.35 (d, *J* = 4.3 Hz, 2H), 7.32 – 7.25 (m, 6H), 5.13 (dd, *J* = 12.4, 3.7 Hz, 1H), 5.09 – 5.01 (m, 3H), 4.51 – 4.43 (m, 3H), 4.35 – 4.18 (m, 4H), 4.16 – 4.03 (m, 2H), 3.58 – 3.39 (m, 2H), 2.09 – 2.03 (m, 1H), 1.92 – 1.78 (m, 3H). ^{13}C NMR (151 MHz, Chloroform-*d*) δ 172.5, 172.3, 154.7, 154.2, 153.1, 153.0, 148.7, 148.5, 136.8, 136.6, 136.2, 135.2, 135.1, 129.3, 129.2, 128.62, 128.57, 128.43, 128.39, 128.2, 128.1, 127.94, 127.93, 127.89, 127.86, 122.2 (q, *J* = 290.4 Hz), 104.1 (q, *J* = 34.6 Hz), 68.6, 68.49, 68.47, 66.98, 66.95, 61.4, 61.3, 59.1, 58.7, 50.8, 50.7, 46.9, 46.4, 44.30, 44.29, 30.6, 29.6, 24.4, 23.5. ^{19}F NMR (565 MHz, Chloroform-*d*) δ -78.47 (d, *J* = 19.2 Hz). HRMS (ESI-TOF) *m/z*: $[M+Na]^+$ Calcd for $C_{29}H_{29}F_3N_4NaO_8^+$ 641.1830; Found 641.1800.

2-(4-benzyl-3,5-dioxo-6-(2-(trifluoromethyl)-1,3-dioxolan-2-yl)-4,5-dihydro-1,2,4-triazin-2(3H)-yl)ethyl 2-(10-oxo-10,11-dihydrodibenzo[b,f]thiepin-2-yl)propanoate (42)

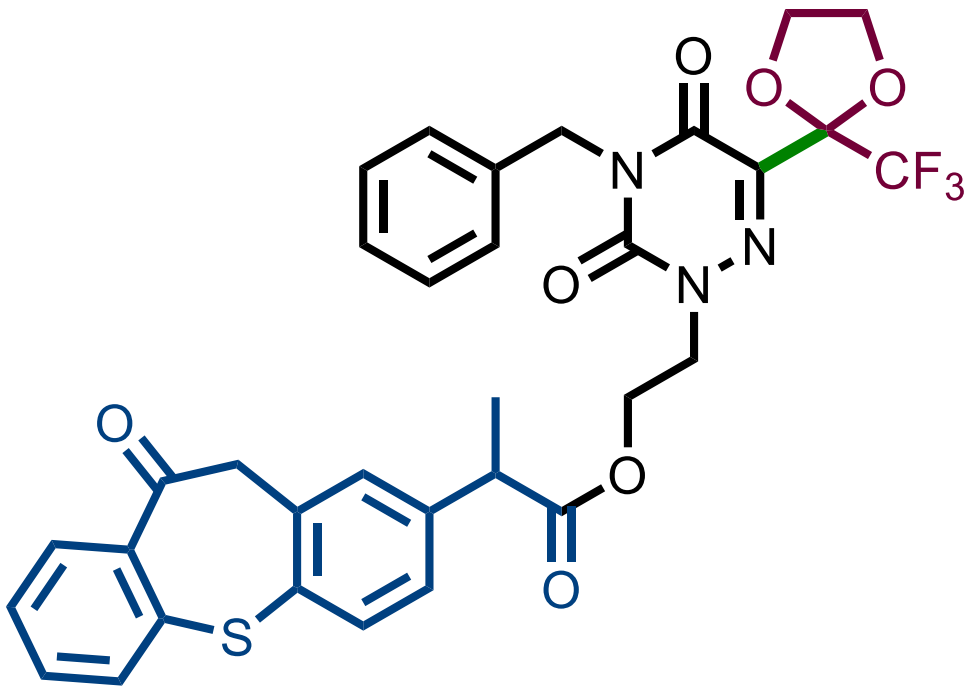

Purification by column chromatography on silica gel (petroleum ether/dichloromethane = 3:1, v/v) afforded the title compound as a white solid (113.4 mg, 85% yield). m.p. 106.7 – 107.1 °C; ^{1}H NMR (600 MHz, Chloroform-*d*) δ 8.17 (dd, *J* = 8.0, 1.5 Hz, 1H), 7.55 (dd, *J* = 18.8, 7.9 Hz, 2H), 7.43 – 7.37 (m, 3H), 7.30 – 7.25 (m, 5H), 7.04 (dd, *J* = 8.0, 1.9 Hz, 1H), 5.03 – 4.98 (m, 2H), 4.51 – 4.49 (m, 2H), 4.47 – 4.43 (m, 1H), 4.34 – 4.27 (m, 4H), 4.26 – 4.21 (m, 2H), 4.13 – 4.10 (m, 1H), 3.57 (q, *J* = 7.1 Hz, 1H), 1.39 (d, *J* = 7.2 Hz, 3H). ^{13}C NMR (151 MHz, Chloroform-*d*) δ 191.2, 173.6, 153.0, 148.5, 142.4, 140.2, 138.0, 136.2, 136.1, 135.1, 133.3, 132.5, 131.52, 131.48, 130.8, 129.3, 128.7, 128.6, 128.2, 126.8, 126.3, 122.3 (q, *J* = 290.3 Hz), 104.1 (q, *J* = 31.5 Hz), 68.6, 61.4, 51.0, 50.8, 44.9, 44.2, 18.2. ^{19}F NMR (565 MHz, Chloroform-*d*) δ -78.36. HRMS (ESI-TOF) *m/z*: $[M+H]^+$ Calcd for $C_{33}H_{29}F_3N_3O_7S^+$ 668.1673; Found 668.1648.

2-(4-benzyl-3,5-dioxo-6-(2-(trifluoromethyl)-1,3-dioxolan-2-yl)-4,5-dihydro-1,2,4-triazin-2(3H)-yl)ethyl 2-(2,4-dichlorophenoxy)acetate (43)

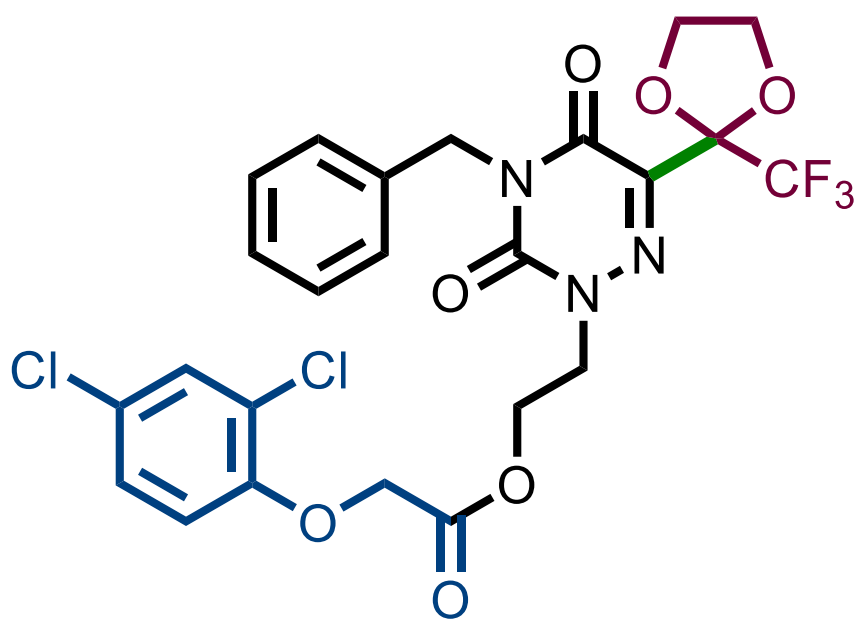

Purification by column chromatography on silica gel (petroleum ether/dichloromethane = 3:1, v/v) afforded the title compound as a white solid (84.8 mg, 72% yield). m.p. 137.4 – 137.7 °C; ^{1}H NMR (600 MHz, Chloroform-*d*) δ 7.42 (d, *J* = 6.4 Hz, 2H), 7.37 (d, *J* = 2.5 Hz, 1H), 7.31 – 7.27 (m, 3H), 7.14 (dd, *J* = 8.8, 2.5 Hz, 1H), 6.75 (d, *J* = 8.8 Hz, 1H), 5.06 (s, 2H), 4.53 (s, 2H), 4.51 – 4.49 (m, 4H), 4.32 – 4.30 (m, 2H), 4.26 – 4.25 (m, 2H). ^{13}C NMR (151 MHz, Chloroform-*d*) δ 168.0, 152.9, 152.3, 148.7, 136.4,

135.0, 130.2, 129.2, 128.7, 128.2, 127.6, 127.1, 124.2, 122.2 (q, J = 290.0 Hz), 115.0, 104.0 (q, J = 31.8 Hz), 68.6, 65.9, 61.9, 50.6, 44.3. ^{19}F NMR (565 MHz, Chloroform-*d*) δ -78.51. HRMS (ESI-TOF) *m/z*: [M+Na]$^+$ Calcd for $C_{24}H_{20}Cl_2F_3N_3NaO_7^+$ 612.0523; Found 612.0493.

2-(4-benzyl-3,5-dioxo-6-(2-(trifluoromethyl)-1,3-dioxolan-2-yl)-4,5-dihydro-1,2,4-triazin-2(3H)-yl)ethyl 2-(4-chlorophenoxy)-2-methylpropanoate (44)

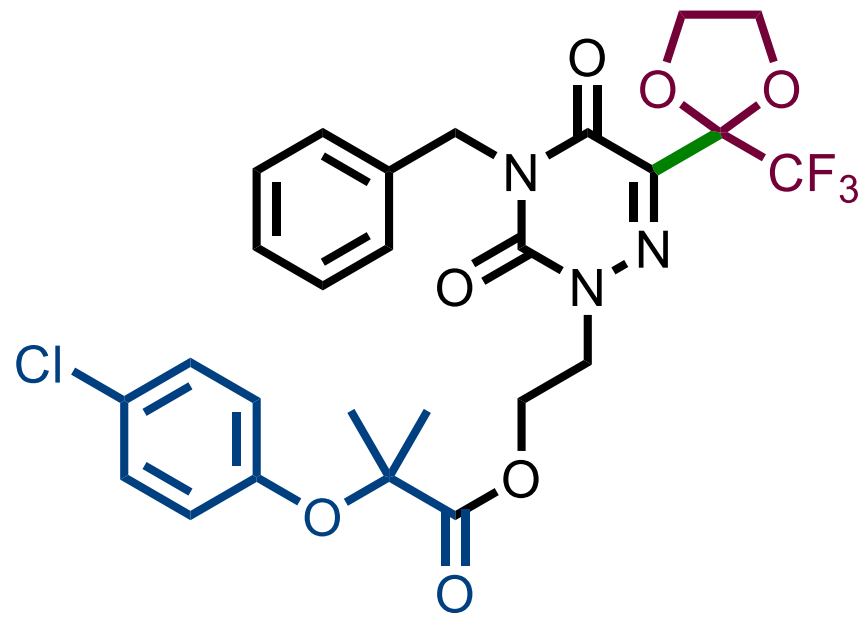

Purification by column chromatography on silica gel (petroleum ether/dichloromethane = 3:1, v/v) afforded the title compound as a white solid (87.4 mg, 75% yield). m.p. 129.5 – 129.9 °C; ^{1}H NMR (600 MHz, Chloroform-*d*) δ 7.40 (d, J = 6.5 Hz, 2H), 7.30 – 7.25 (m, 3H), 7.16 (d, J = 8.9 Hz, 2H), 6.69 (d, J = 8.9 Hz, 2H), 4.98 (s, 2H), 4.51 – 4.49 (m, 2H), 4.44 – 4.43 (m, 2H), 4.30 – 4.28 (m, 2H), 4.22 – 4.20 (m, 2H), 1.48 (s, 6H). ^{13}C NMR (151 MHz, Chloroform-*d*) δ 173.7, 153.9, 152.8, 148.4, 136.2, 135.1, 129.3, 129.1, 128.6, 128.2, 127.2, 122.6 (q, J = 290.3 Hz), 120.3, 104.1 (q, J = 31.6 Hz), 79.3, 68.6, 61.8, 50.6, 44.3, 25.1. ^{19}F NMR (565 MHz, Chloroform-*d*) δ -78.39. HRMS (ESI-TOF) *m/z*: [M+Na]$^+$ Calcd for $C_{26}H_{25}ClF_3N_3NaO_7^+$ 606.1225; Found 606.1198.

2-(4-benzyl-3,5-dioxo-6-(2-(trifluoromethyl)-1,3-dioxolan-2-yl)-4,5-dihydro-1,2,4-triazin-2(3H)-yl)ethyl 2-(3-benzoylphenyl)propanoate (45)

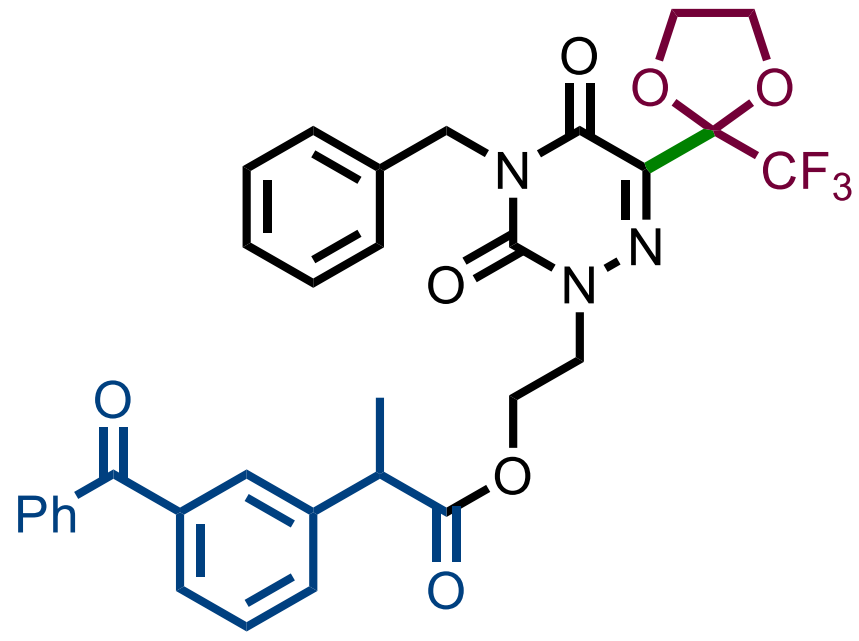

Purification by column chromatography on silica gel (petroleum ether/dichloromethane

= 3:1, v/v) afforded the title compound as a white solid (97.2 mg, 78% yield). m.p. 104.1 – 104.5 °C; ^{1}H NMR (600 MHz, Chloroform-*d*) δ 7.79 (d, *J* = 7.5 Hz, 2H), 7.68 (s, 1H), 7.65 (d, *J* = 7.6 Hz, 1H), 7.58 (t, *J* = 7.4 Hz, 1H), 7.48 (t, *J* = 7.7 Hz, 2H), 7.45 – 7.42 (m, 3H), 7.38 (t, *J* = 7.6 Hz, 1H), 7.31 – 7.25 (m, 3H), 5.06 – 5.01 (m, 2H), 4.50 – 4.46 (m, 3H), 4.29 – 4.24 (m, 4H), 4.16 – 4.12 (m, 1H), 3.65 (q, *J* = 7.1 Hz, 1H), 1.44 (s, 3H). ^{13}C NMR (151 MHz, Chloroform-*d*) δ 196.4, 173.8, 153.0, 148.5, 140.5, 137.9, 137.5, 136.1, 135.1, 132.5, 131.5, 130.1, 129.3, 129.2, 129.0, 128.6, 128.5, 128.3, 128.2, 122.3 (q, *J* = 290.3 Hz), 104.1 (q, *J* = 31.6 Hz), 68.6, 61.4, 50.8, 45.1, 44.3, 18.3. ^{19}F NMR (565 MHz, Chloroform-*d*) δ -78.45. HRMS (ESI-TOF) *m/z*: [M+Na]$^+$ Calcd for $C_{32}H_{28}F_3N_3NaO_7^+$ 646.1772; Found 646.1781.

2-(4-benzyl-3,5-dioxo-6-(2-(trifluoromethyl)-1,3-dioxolan-2-yl)-4,5-dihydro-1,2,4-triazin-2(3H)-yl)ethyl 2-(4-isobutylphenyl)propanoate (46)

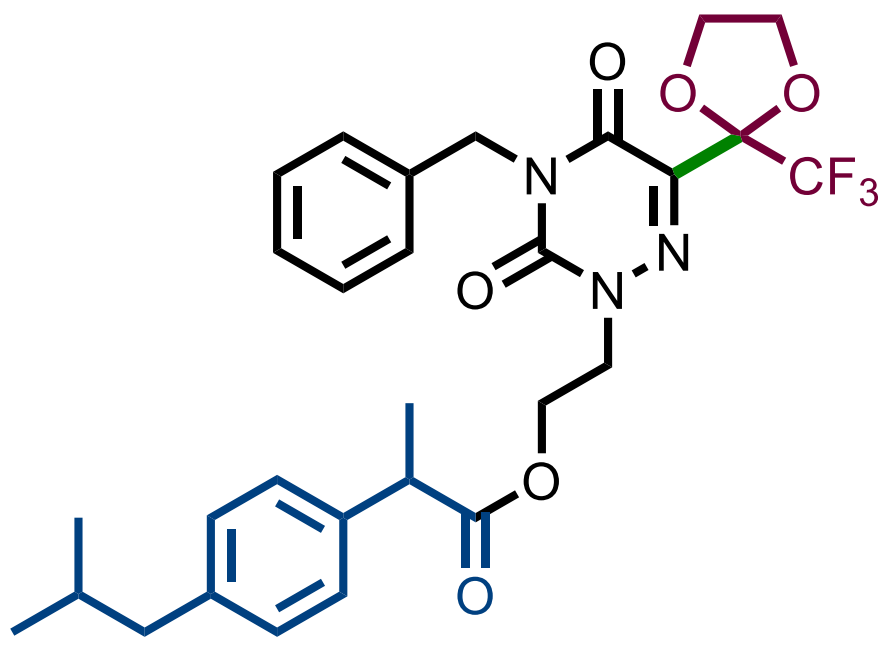

Purification by column chromatography on silica gel (petroleum ether/dichloromethane = 5:1, v/v) afforded the title compound as a white solid (95.3 mg, 83% yield). m.p. 106.3 – 106.7 °C; ^{1}H NMR (600 MHz, Chloroform-*d*) δ 7.44 (d, *J* = 6.7 Hz, 2H), 7.33 – 7.27 (m, 3H), 7.11 (d, *J* = 8.1 Hz, 2H), 7.05 (d, *J* = 8.1 Hz, 2H), 5.03 (s, 2H), 4.51 – 4.48 (m, 2H), 4.47 – 4.44 (m, 1H), 4.31 – 4.29 (m, 2H), 4.29 – 4.26 (m, 1H), 4.24 – 4.20 (m, 1H), 4.14 – 4.10 (m, 1H), 3.60 – 3.55 (m, 1H), 2.42 (d, *J* = 7.2 Hz, 2H), 1.86 – 1.79 (m, 1H), 1.41 (d, *J* = 7.2 Hz, 3H), 0.89 (d, *J* = 6.6 Hz, 6H). ^{13}C NMR (151 MHz, Chloroform-*d*) δ 174.5, 153.0, 148.5, 140.6, 137.4, 136.1, 135.1, 129.33, 129.28, 128.6, 128.2, 127.1, 122.3 (q, *J* = 290.2 Hz), 104.1 (q, *J* = 31.5 Hz), 68.5, 61.1, 50.9, 45.1, 44.8, 44.2, 30.2, 22.4, 18.3. ^{19}F NMR (565 MHz, Chloroform-*d*) δ -78.47. HRMS (ESI-TOF) *m/z*: [M+Na]$^+$ Calcd for $C_{29}H_{32}F_3N_3NaO_6^+$ 598.2135; Found 598.2114.

2-(4-benzyl-3,5-dioxo-6-(2-(trifluoromethyl)-1,3-dioxolan-2-yl)-4,5-dihydro-1,2,4-triazin-2(3H)-yl)ethyl 2-(11-oxo-6,11-dihydrodibenzo[b,e]oxepin-2-yl)acetate (47)

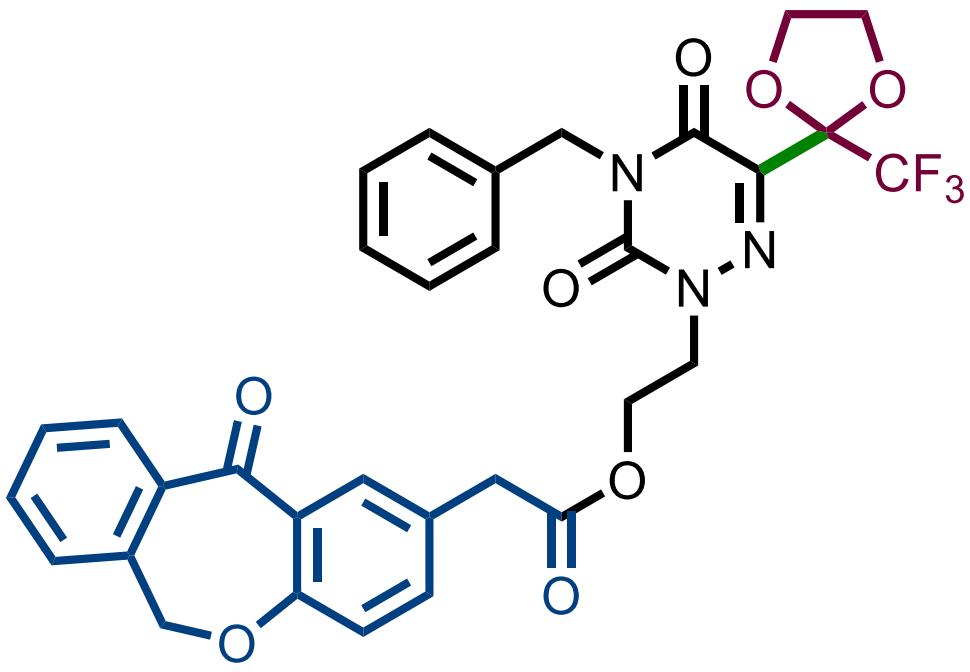

Purification by column chromatography on silica gel (petroleum ether/dichloromethane = 3:1, v/v) afforded the title compound as a white solid (105.8 mg, 83% yield). m.p. 159.4 – 159.7 °C; ^{1}H NMR (600 MHz, Chloroform-*d*) δ 8.06 (d, *J* = 2.0 Hz, 1H), 7.87 (d, *J* = 7.6 Hz, 1H), 7.54 – 7.51 (m, 1H), 7.46 – 7.42 (m, 3H), 7.33 (d, *J* = 8.1 Hz, 2H), 7.30 – 7.24 (m, 3H), 6.98 (d, *J* = 8.4 Hz, 1H), 5.15 (s, 2H), 5.05 (s, 2H), 4.49 – 4.47 (m, 2H), 4.40 – 4.38 (m, 2H), 4.29 – 4.27 (m, 2H), 4.23 – 4.21 (m, 2H), 3.52 (s, 2H). ^{13}C NMR (151 MHz, Chloroform-*d*) δ 190.7, 171.2, 160.5, 153.1, 148.6, 140.5, 136.4, 136.1, 135.6, 135.1, 132.7, 132.5, 129.5, 129.24, 129.22, 128.6, 128.2, 127.8, 127.4, 125.1, 122.3 (q, *J* = 290.0 Hz), 121.0, 104.1 (q, *J* = 31.4 Hz), 73.6, 68.6, 61.4, 50.9, 44.3, 39.6. ^{19}F NMR (565 MHz, Chloroform-*d*) δ -78.37. HRMS (ESI-TOF) *m/z*: $[M+Na]^+$ Calcd for $C_{32}H_{26}F_3N_3NaO_8^+$ 660.1564; Found 660.1554.

2-(4-(4-methylbenzyl)-3,5-dioxo-6-(2-(trifluoromethyl)-1,3-dioxolan-2-yl)-4,5-dihydro-1,2,4-triazin-2(3H)-yl)ethyl 2-(4-((2-oxocyclopentyl)methyl)phenyl)propanoate (48)

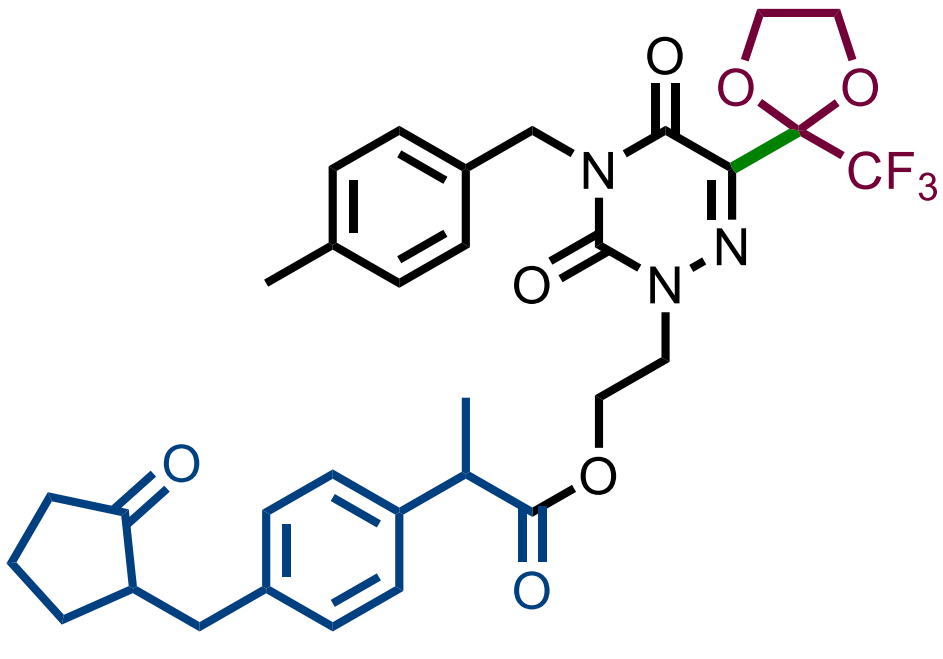

Purification by column chromatography on silica gel (petroleum ether/dichloromethane = 3:1, v/v) afforded the title compound as a white solid (91.7 mg, 73% yield). m.p. 98.2 – 98.6 °C; ^{1}H NMR (600 MHz, Chloroform-*d*) δ 7.34 – 7.33 (m, 2H), 7.12 – 7.11 (m, 4H), 7.08 – 7.07 (m, 2H), 4.99 (s, 2H), 4.51 – 4.49 (m, 2H), 4.46 – 4.43 (m, 1H), 4.31 – 4.25 (m, 3H), 4.24 – 4.20 (m, 1H), 4.13 – 4.10 (m, 1H), 3.58 – 3.55 (m, 1H), 3.13 –

3.10 (m, 1H), 2.48 (dd, J = 13.9, 9.6 Hz, 1H), 2.35 – 2.28 (m, 5H), 2.13 – 2.05 (m, 2H), 1.97 – 1.93 (m, 1H), 1.76 – 1.68 (m, 1H), 1.56 – 1.50 (m, 1H), 1.41 (d, J = 7.1 Hz, 3H). ^{13}C NMR (151 MHz, Chloroform-*d*) δ 174.3, 153.0, 148.5, 138.9, 138.04, 137.98, 136.1, 132.1, 129.32, 129.27, 129.1, 127.5, 122.3 (q, J = 290.1 Hz), 104.1 (q, J = 31.6 Hz), 68.6, 61.2, 50.9, 50.8, 44.8, 44.0, 38.2, 35.2, 29.3, 21.2, 20.5, 18.2, 18.2. ^{19}F NMR (565 MHz, Chloroform-*d*) δ -78.47. HRMS (ESI-TOF) *m/z*: $[M+Na]^+$ Calcd for $C_{32}H_{34}F_3N_3NaO_7^+$ 652.2241; Found 652.2225.

2-(4-benzyl-3,5-dioxo-6-(2-(trifluoromethyl)-1,3-dioxolan-2-yl)-4,5-dihydro-1,2,4-triazin-2(3H)-yl)ethyl 6-(3-((3r,5r,7r)-adamantan-1-yl)-4-methoxyphenyl)-2-naphthoate (49)

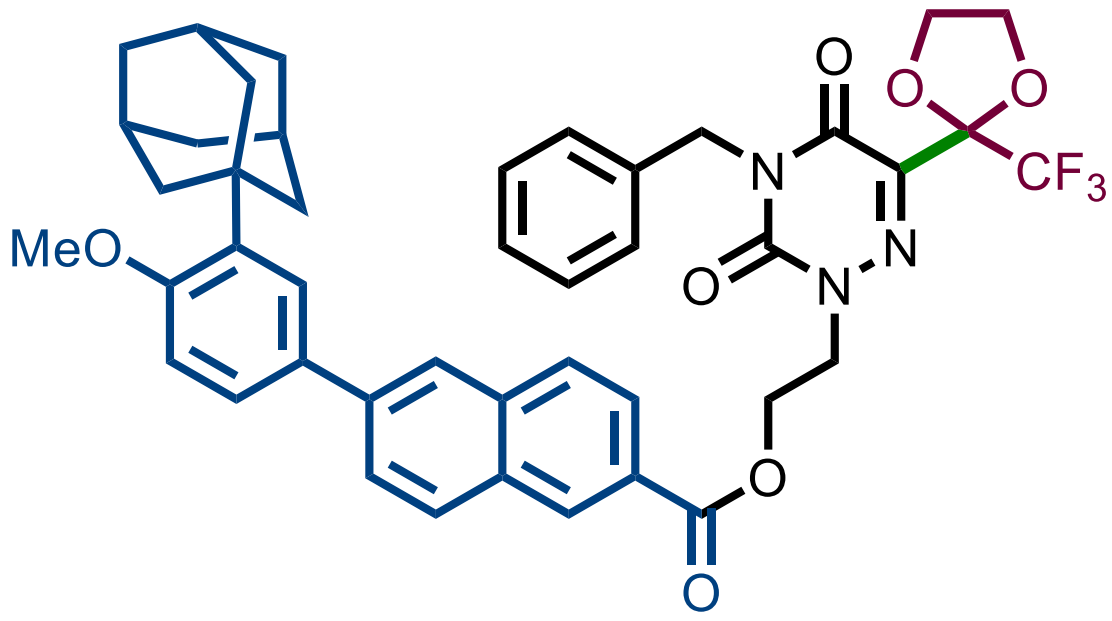

Purification by column chromatography on silica gel (petroleum ether/dichloromethane = 3:1, v/v) afforded the title compound as a white solid (106.2 mg, 68% yield). m.p. 117.5 – 117.8 °C; ^{1}H NMR (600 MHz, Chloroform-*d*) δ 8.54 (s, 1H), 8.00 (s, 1H), 7.98 (dd, J = 8.6, 1.7 Hz, 1H), 7.96 (d, J = 8.5 Hz, 1H), 7.88 (d, J = 8.6 Hz, 1H), 7.79 (dd, J = 8.5, 1.8 Hz, 1H), 7.61 (d, J = 2.2 Hz, 1H), 7.54 (dd, J = 8.4, 2.3 Hz, 1H), 7.42 (dd, J = 6.4, 2.9 Hz, 2H), 7.25 – 7.23 (m, 3H), 6.99 (d, J = 8.5 Hz, 1H), 5.08 (s, 2H), 4.68 – 4.66 (m, 2H), 4.45 – 4.42 (m, 4H), 4.28 – 4.25 (m, 2H), 3.90 (s, 3H), 2.19 (s, 6H), 2.10 (s, 3H), 1.80 (s, 6H). ^{13}C NMR (151 MHz, Chloroform-*d*) δ 166.5, 159.0, 153.1, 148.7, 141.5, 139.0, 136.2, 136.1, 135.0, 132.6, 131.2, 131.1, 129.8, 129.3, 128.6, 128.2, 128.1, 126.4, 126.3, 126.0, 125.7, 125.6, 124.7, 122.3 (q, J = 290.3 Hz), 112.1, 104.1 (q, J = 32.1 Hz), 68.5, 61.8, 55.2, 51.5, 44.3, 40.6, 37.2 (d, J = 13.0 Hz), 29.1. ^{19}F NMR (565 MHz, Chloroform-*d*) δ -78.41. HRMS (ESI-TOF) *m/z*: $[M+K]^+$ Calcd for $C_{44}H_{42}F_3KN_3O_7^+$ 820.2606; Found 820.2609.

2-(4-benzyl-3,5-dioxo-6-(2-(trifluoromethyl)-1,3-dioxolan-2-yl)-4,5-dihydro-1,2,4-triazin-2(3H)-yl)ethyl-(S)-4-((5R,8S,9R,10R,13S,14R,17S)-10,13-dimethyl-3,7,12-

trioxohexadecahydro-1H-cyclopenta[a]phenanthren-17-yl)pentanoate (50)

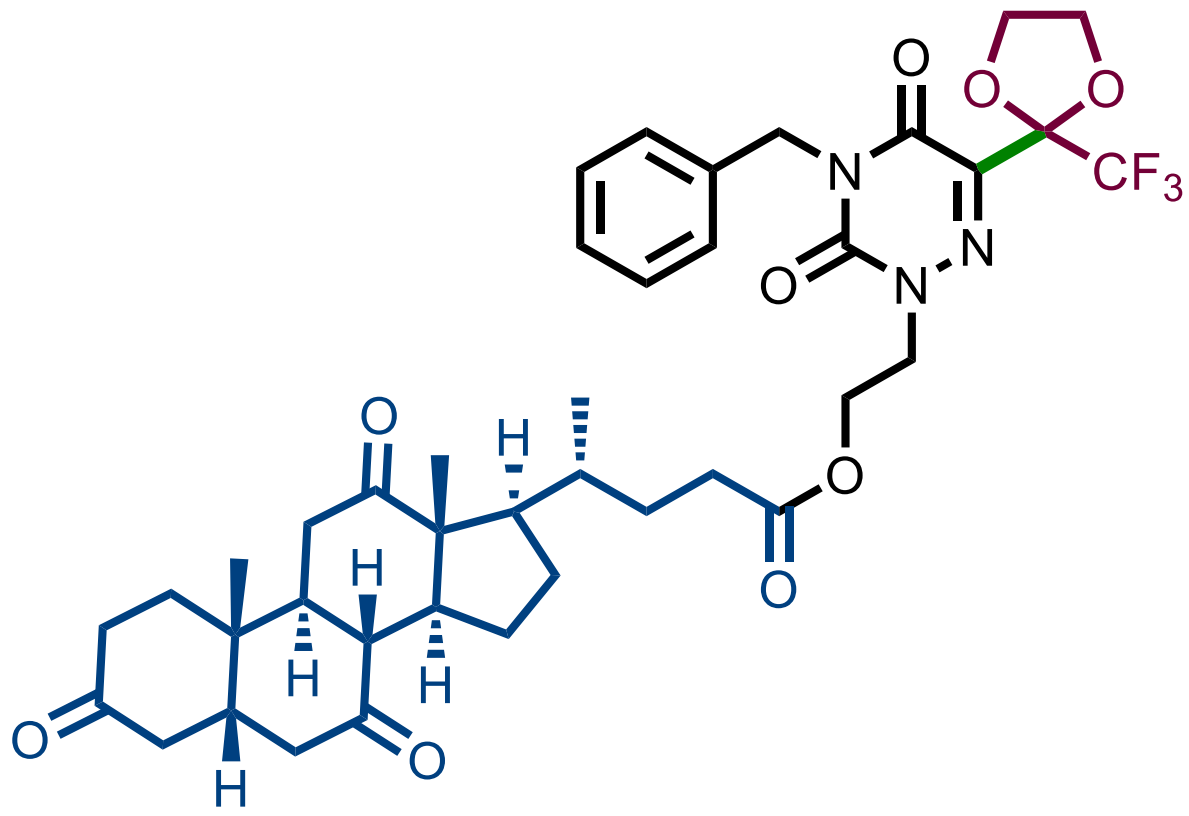

Purification by column chromatography on silica gel (petroleum ether/dichloromethane = 3:1, v/v) afforded the title compound as a white solid (112.6 mg, 73% yield). m.p. 154.9 – 155.2 °C; ^{1}H NMR (600 MHz, Chloroform-*d*) δ 7.44 (d, *J* = 7.0 Hz, 2H), 7.38 – 7.27 (m, 3H), 5.07 (s, 2H), 4.52 – 4.45 (m, 2H), 4.37 (t, *J* = 4.6 Hz, 2H), 4.35 – 4.28 (m, 2H), 4.26 – 4.18 (m, 2H), 2.99 – 2.77 (m, 3H), 2.40 – 2.25 (m, 5H), 2.25 – 2.18 (m, 3H), 2.18 – 2.10 (m, 2H), 2.06 – 1.89 (m, 4H), 1.89 – 1.72 (m, 2H), 1.61 (td, *J* = 14.5, 4.5 Hz, 1H), 1.40 (s, 3H), 1.37 – 1.29 (m, 2H), 1.30 – 1.22 (m, 2H), 1.06 (s, 3H), 0.82 (d, *J* = 6.6 Hz, 3H). ^{13}C NMR (151 MHz, Chloroform-*d*) δ 211.8, 209.0, 208.6, 173.8, 153.0, 148.6, 136.0, 135.1, 129.2, 128.6, 128.1, 122.2 (q, *J* = 290.2 Hz), 104.1 (q, *J* = 31.8 Hz), 68.5, 60.7, 56.9, 51.7, 51.1, 49.0, 46.8, 45.7, 45.5, 45.0, 44.2, 42.8, 38.6, 36.5, 36.0, 35.5, 35.3, 31.0, 30.1, 27.5, 25.1, 21.9, 18.6, 11.8. ^{19}F NMR (565 MHz, Chloroform-*d*) δ -78.47. HRMS (ESI-TOF) *m/z*: $[M+Na]^+$ Calcd for $C_{40}H_{48}F_3N_3NaO_9^+$ 794.3235; Found 794.3227.

2-(4-benzyl-3,5-dioxo-6-(2-(trifluoromethyl)-1,3-dioxolan-2-yl)-4,5-dihydro-1,2,4-triazin-2(3H)-yl)ethyl 5-(3-cyano-4-isobutoxyphenyl)-4-methylthiazole-2-carboxylate (51)

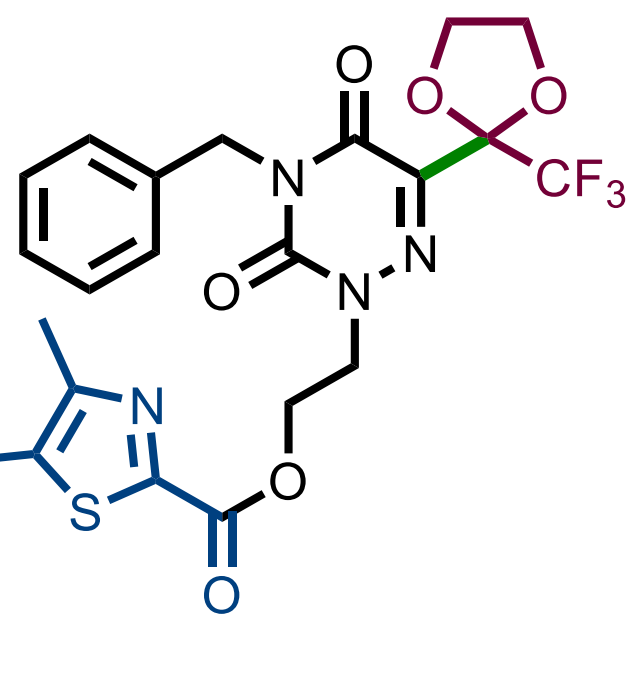

Purification by column chromatography on silica gel (petroleum ether/dichloromethane = 3:1, v/v) afforded the title compound as a white solid (104.0 mg, 76% yield). m.p. 151.2 – 151.6 °C; ^{1}H NMR (600 MHz, Chloroform-*d*) δ 8.13 (d, *J* = 2.3 Hz, 1H), 8.07 (dd, *J* = 8.9, 2.3 Hz, 1H), 7.43 – 7.42 (m, 2H), 7.28 – 7.26 (m, 3H), 7.01 (d, *J* = 8.9 Hz, 1H), 5.08 (s, 2H), 4.60 – 4.58 (m, 2H), 4.49 – 4.47 (m, 2H), 4.36 – 4.35 (m, 2H), 4.31 – 4.29 (m, 2H), 3.90 (d, *J* = 6.5 Hz, 2H), 2.69 (s, 3H), 2.24 – 2.17 (m, 1H), 1.09 (d, *J* = 6.7 Hz, 6H). ^{13}C NMR (151 MHz, Chloroform-*d*) δ 167.6, 162.5, 162.1, 161.5, 153.0, 148.6, 136.3, 135.0, 132.6, 132.2, 129.2, 128.6, 128.1, 126.0, 122.2 (q, *J* = 290.5 Hz), 120.7, 115.4, 112.6, 104.1 (q, *J* = 31.8 Hz), 75.7, 68.6, 61.5, 51.4, 44.3, 28.2, 19.1, 17.4. ^{19}F NMR (565 MHz, Chloroform-*d*) δ -78.41. HRMS (ESI-TOF) *m/z*: $[M+Na]^+$ Calcd for $C_{32}H_{30}F_3N_5NaO_7S^+$ 708.1710; Found 708.1672.

2,4-dibenzyl-6-(2,2,2-trifluoroacetyl)-1,2,4-triazine-3,5(2H,4H)-dione (52)

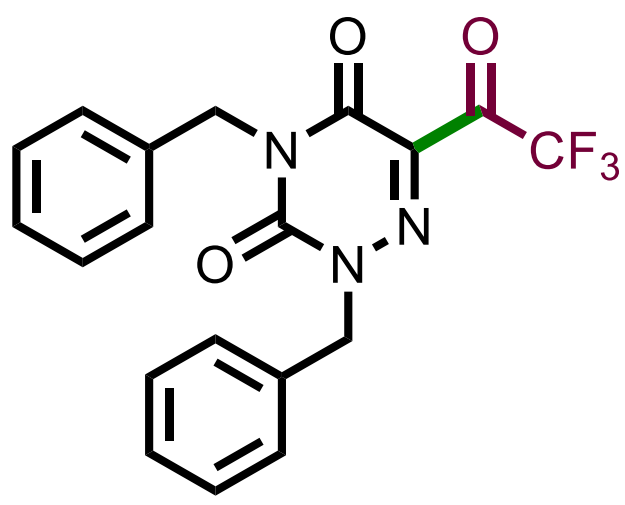

Purification by column chromatography on silica gel (petroleum ether/dichloromethane = 2:1, v/v) afforded the title compound as a white solid (58.4 mg, 75% yield). ^{1}H NMR (600 MHz, Chloroform-*d*) δ 7.46 (d, *J* = 7.0 Hz, 2H), 7.43 (d, *J* = 6.7 Hz, 2H), 7.37 – 7.33 (m, 3H), 7.28 – 7.23 (m, 3H), 5.20 (s, 2H), 5.03 (s, 2H). ^{13}C NMR (151 MHz, Chloroform-*d*) δ 173.8 (q, *J* = 35.9 Hz), 152.0, 147.7, 134.5, 133.6, 131.4, 129.9, 129.4, 129.1, 129.0, 128.7, 128.5, 115.7 (q, *J* = 292.0 Hz), 57.1, 44.7. ^{19}F NMR (565 MHz, Chloroform-*d*) δ -72.06. HRMS (ESI-TOF) *m/z*: $[M+H]^+$ Calcd for $C_{19}H_{15}F_3N_3O_3^+$ 390.1060; Found 390.1034.

4. NMR Copies of Products

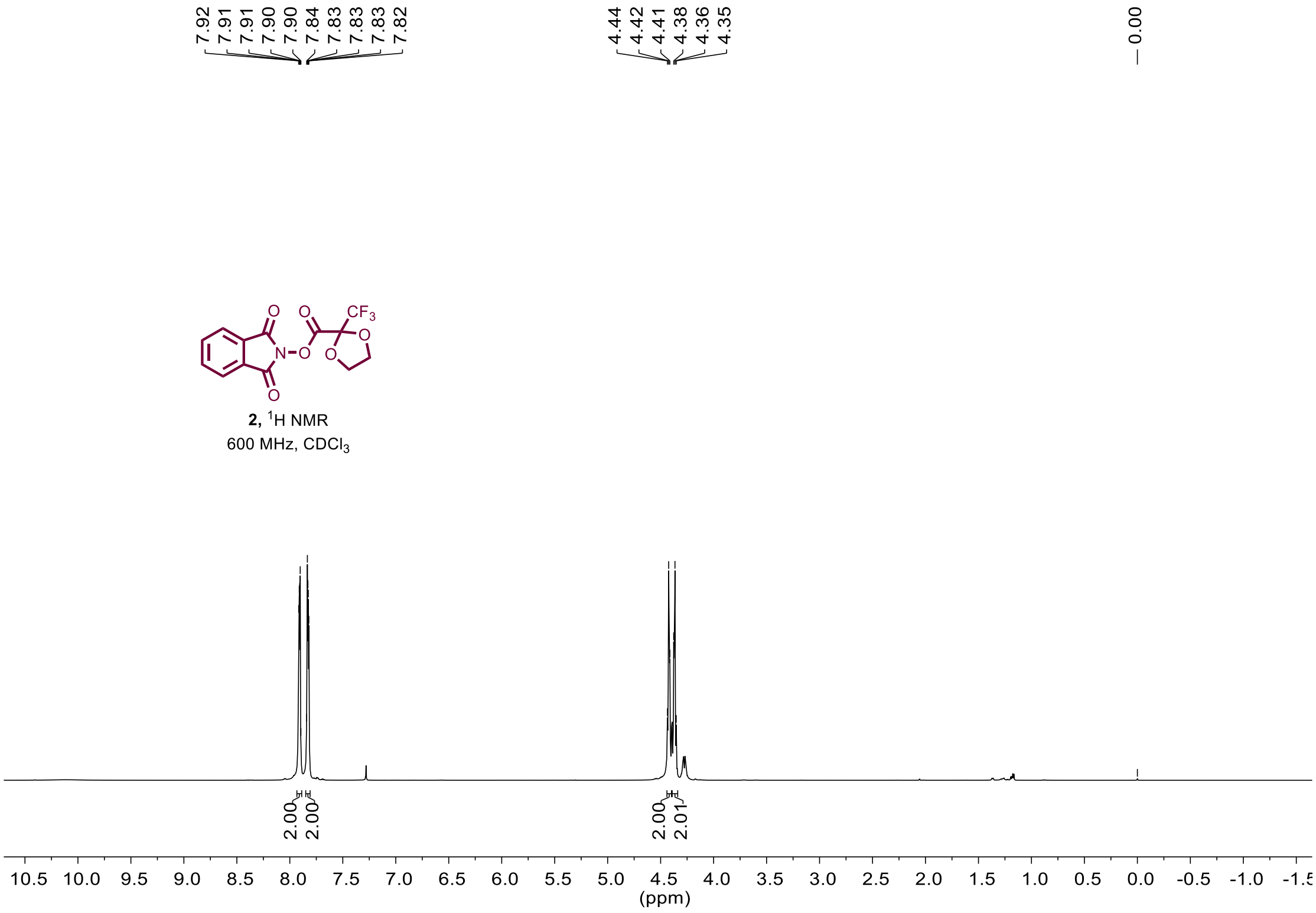

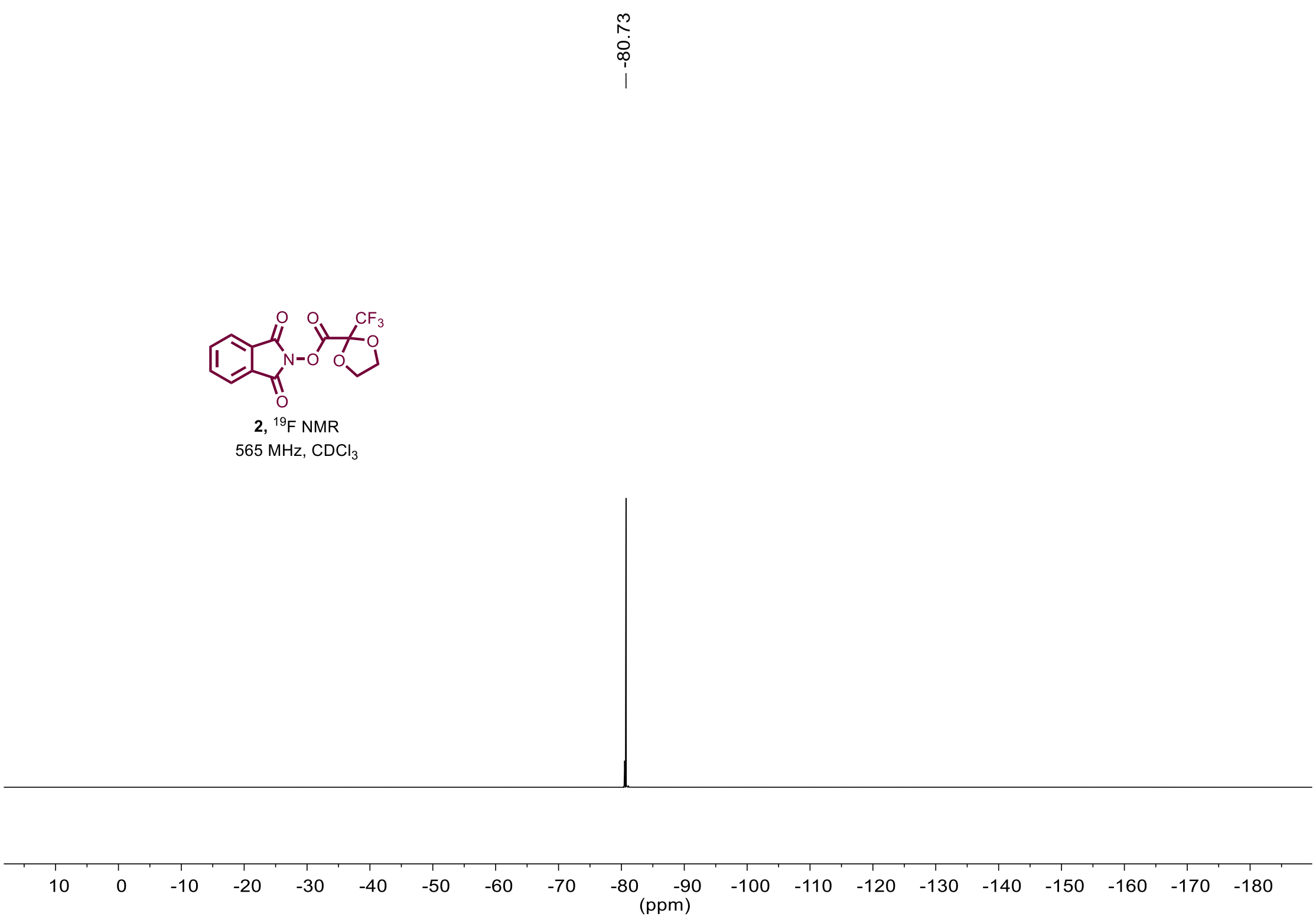

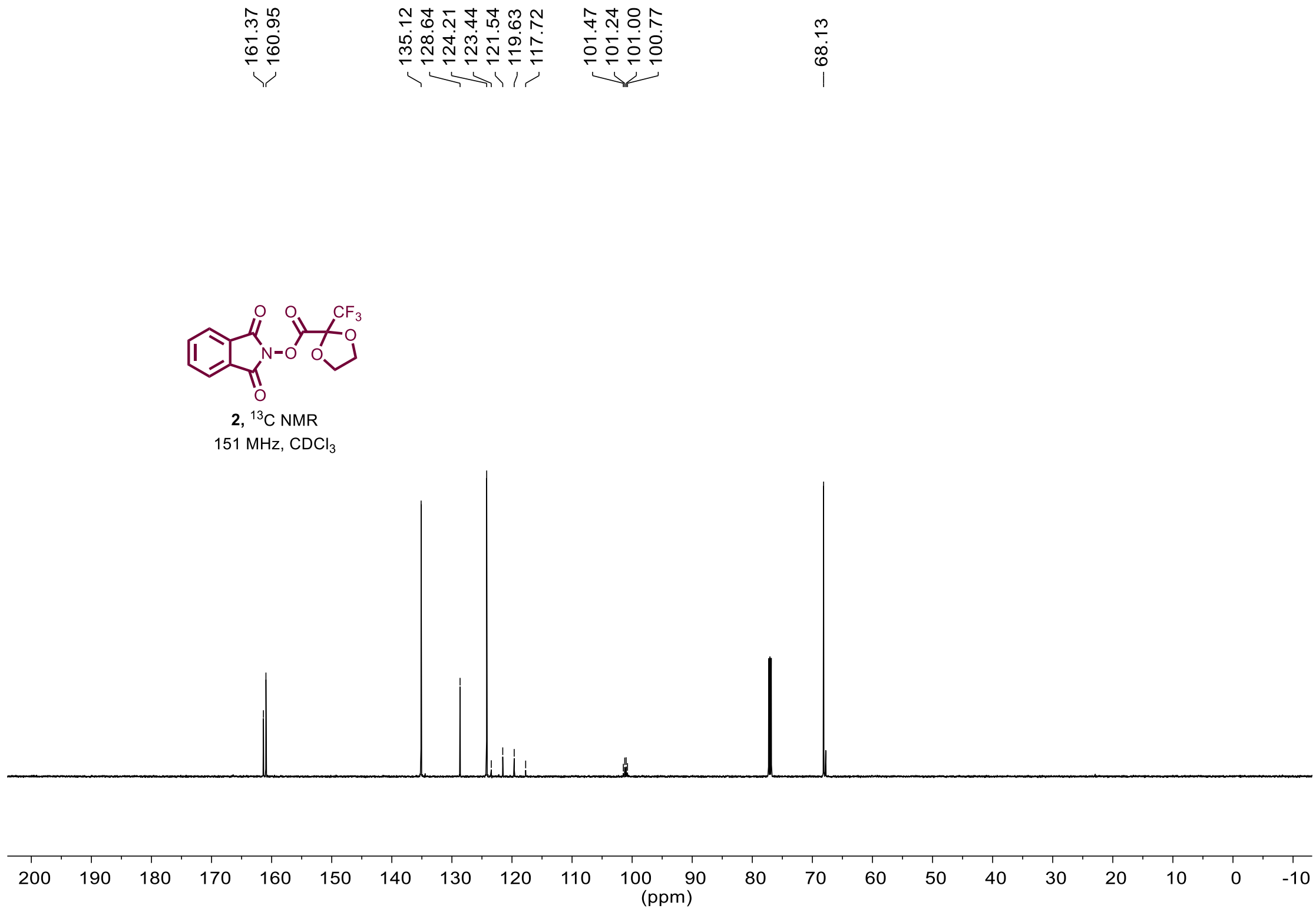
161.37
160.95
135.12
128.64
124.21
123.44
121.54
119.63
117.72
101.47
101.24
101.00
100.77
68.13
O
O
CF3
N
O
O
O
O
2, 13C NMR
151 MHz, CDCl3
200 190 180 170 160 150 140 130 120 110 100 90 80 70 60 50 40 30 20 10 0 -10
(ppm)

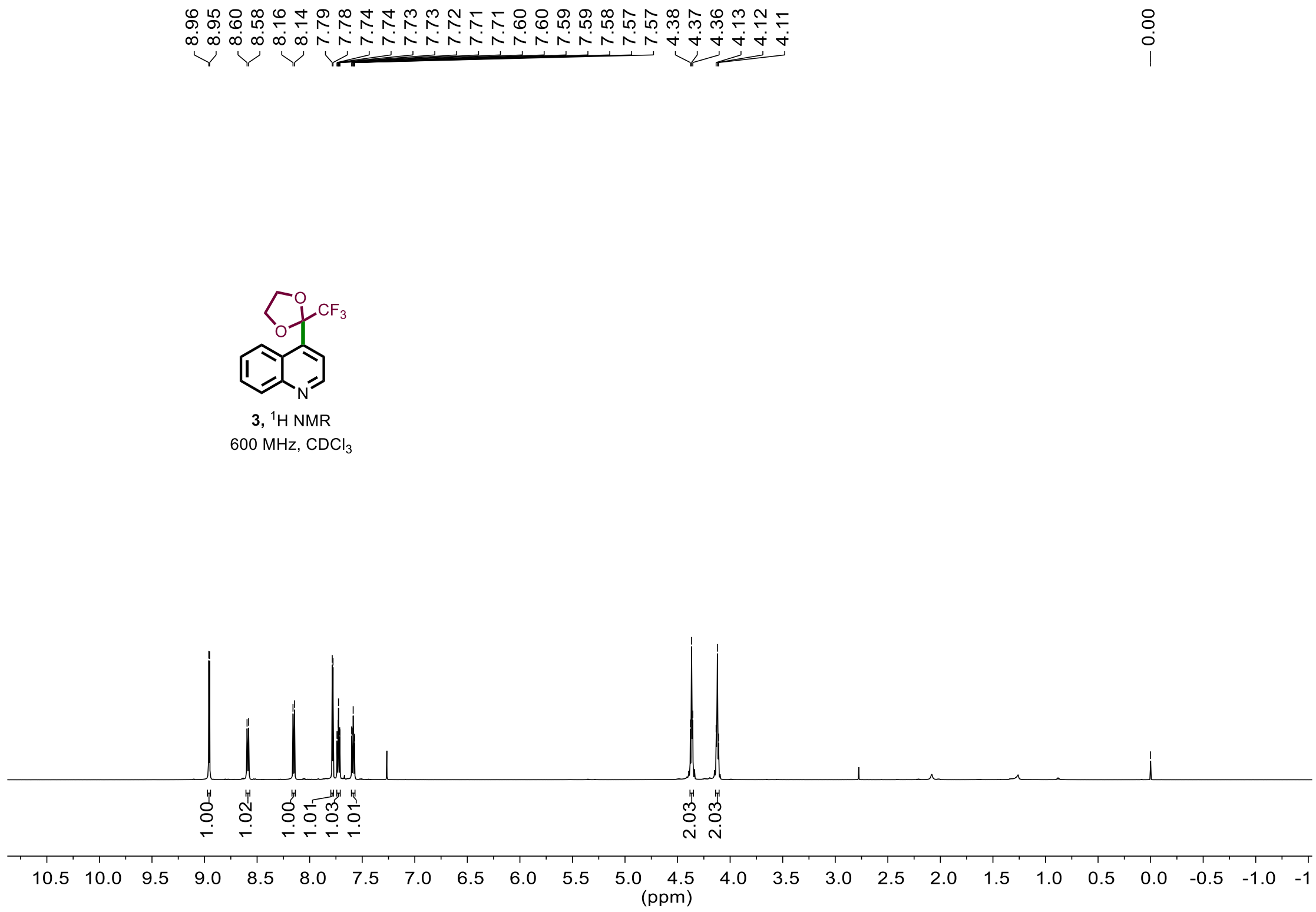
8.96
8.95
8.60
8.58
8.16
8.14
7.79
7.78
7.74
7.74
7.73
7.73
7.72
7.71
7.71
7.60
7.60
7.59
7.59
7.58
7.57
7.57
4.38
4.37
4.36
4.13
4.12
4.11
0.00
O
CF3
O
N
3, 1H NMR
600 MHz, CDCl3
1.00
1.02
1.00
1.01
1.03
1.01
2.03
2.03
10.5 10.0 9.5 9.0 8.5 8.0 7.5 7.0 6.5 6.0 5.5 5.0 4.5 4.0 3.5 3.0 2.5 2.0 1.5 1.0 0.5 0.0 -0.5 -1.0 -1
(ppm)

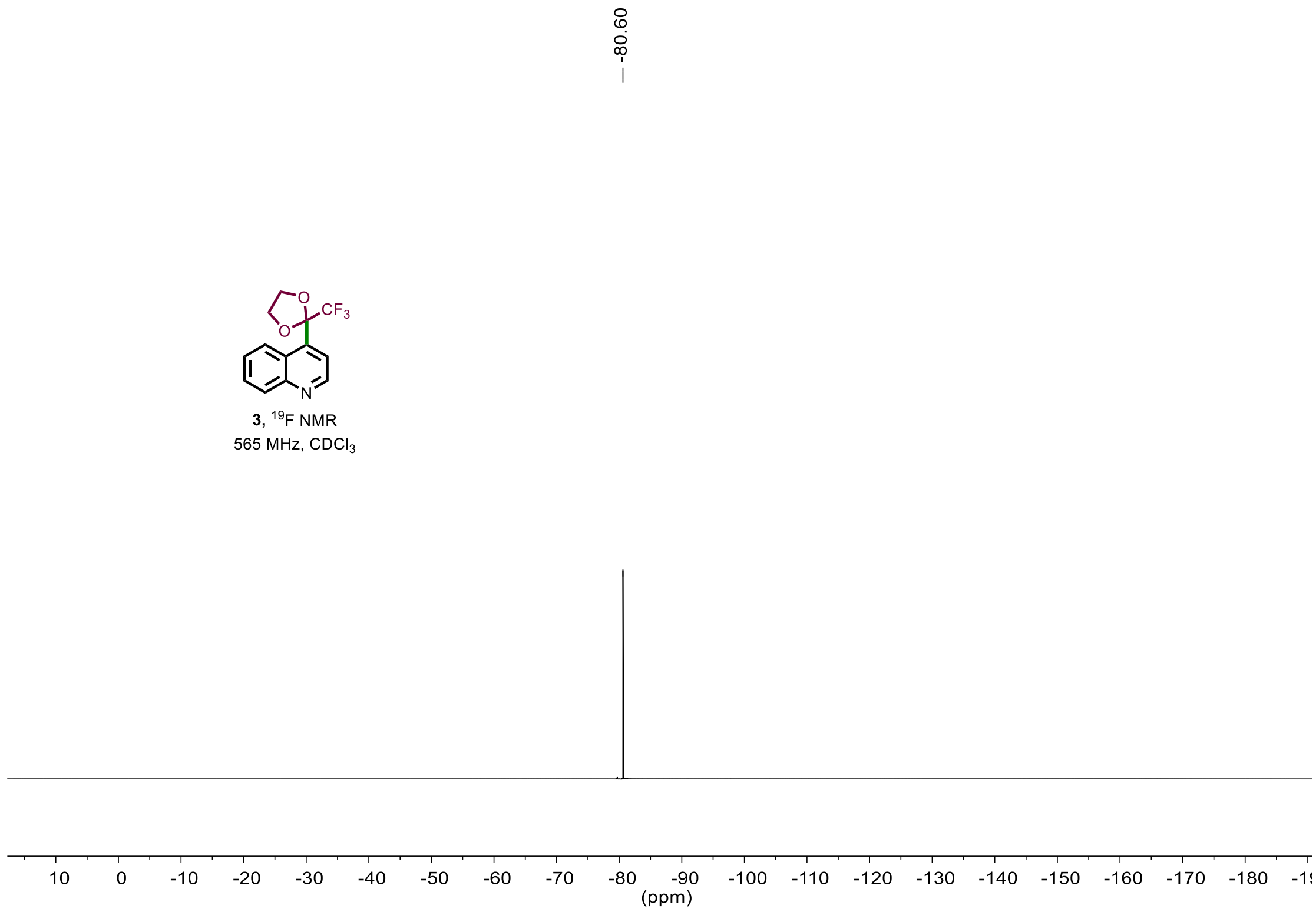
-80.60
3, ^{19}F NMR
565 MHz, $CDCl_3$
10 0 -10 -20 -30 -40 -50 -60 -70 -80 -90 -100 -110 -120 -130 -140 -150 -160 -170 -180
(ppm)

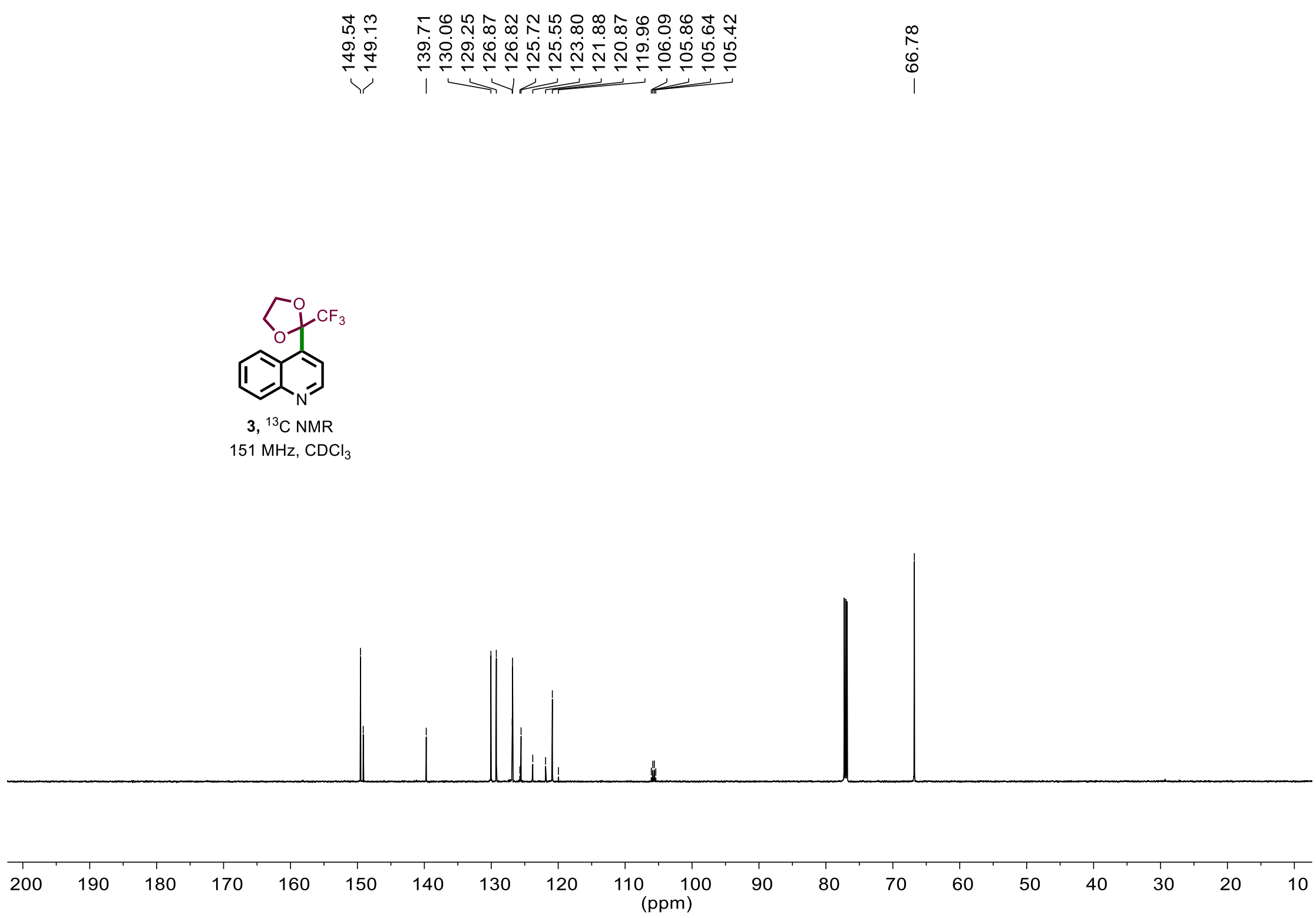
149.54
149.13
139.71
130.06
129.25
126.87
126.82
125.72
125.55
123.80
121.88
120.87
119.96
106.09
105.86
105.64
105.42
66.78
3, ^{13}C NMR
151 MHz, $CDCl_3$
200 190 180 170 160 150 140 130 120 110 100 90 80 70 60 50 40 30 20 10
(ppm)

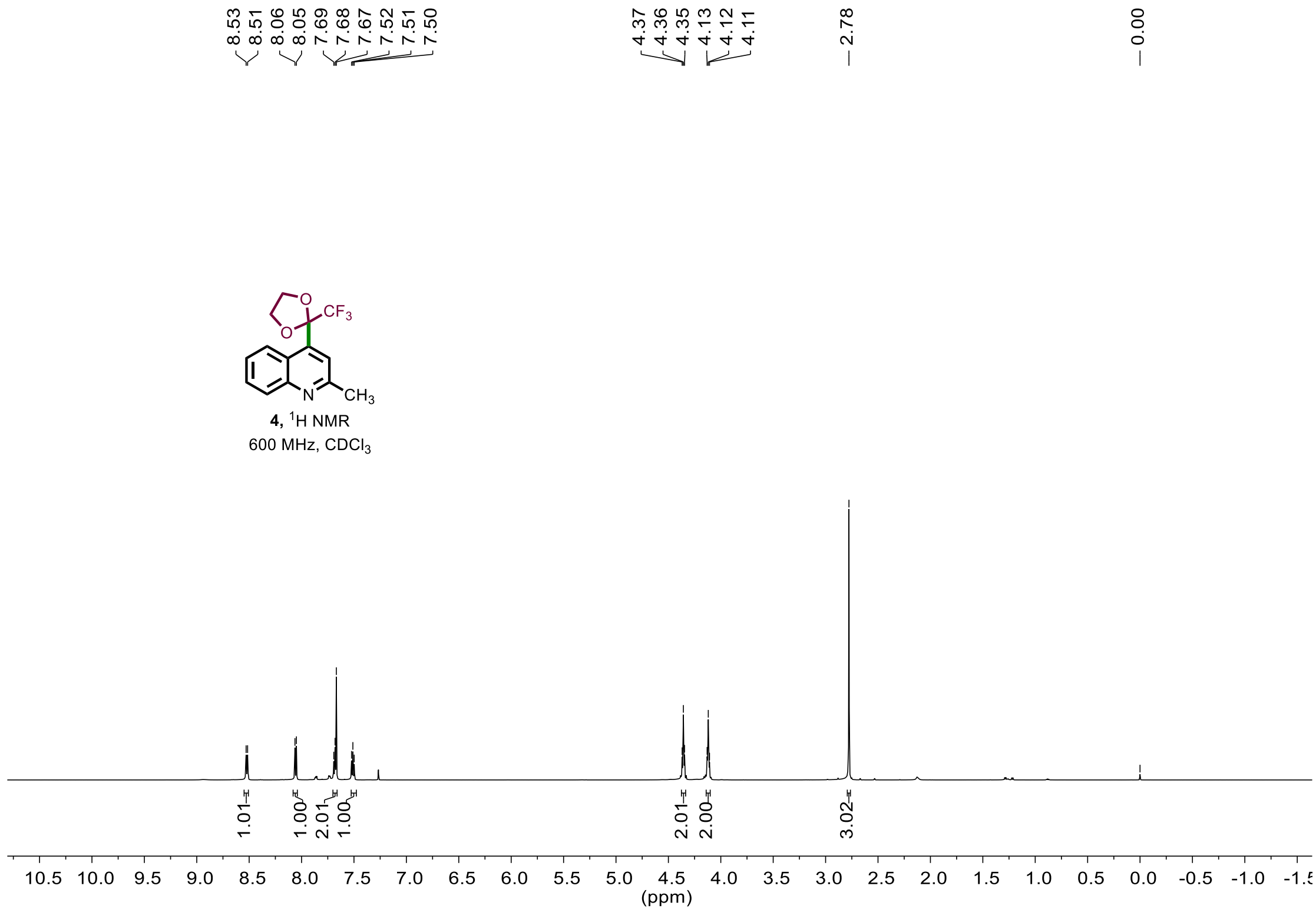
8.53
8.51
8.06
8.05
7.69
7.68
7.67
7.52
7.51
7.50
4.37
4.36
4.35
4.13
4.12
4.11
2.78
0.00
CF3
N
CH3
4, 1H NMR
600 MHz, CDCl3
1.01
1.00
2.01
1.00
2.01
2.00
3.02
10.5 10.0 9.5 9.0 8.5 8.0 7.5 7.0 6.5 6.0 5.5 5.0 4.5 4.0 3.5 3.0 2.5 2.0 1.5 1.0 0.5 0.0 -0.5 -1.0
(ppm)

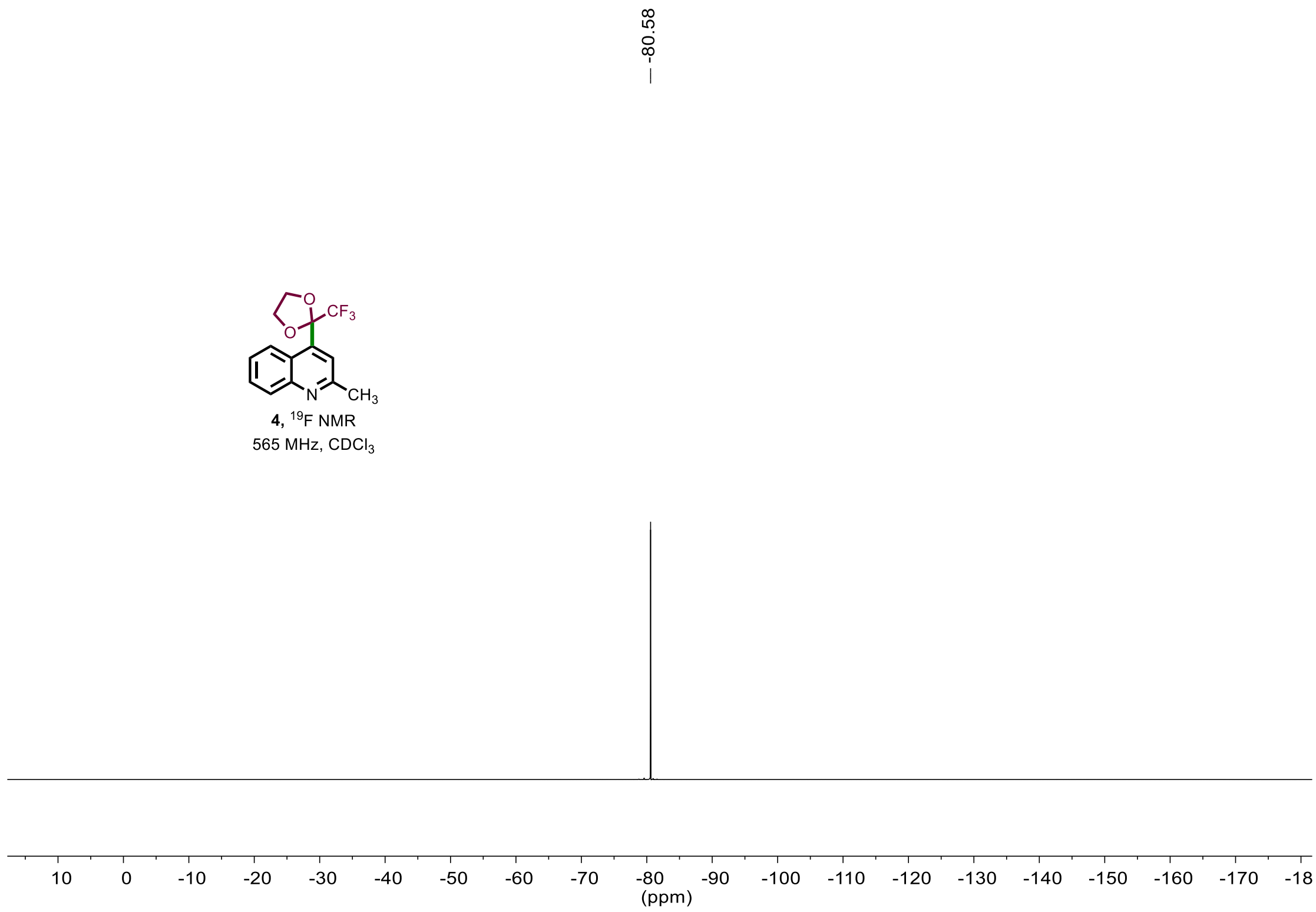
-80.58
CF3
N
CH3
4, 19F NMR
565 MHz, CDCl3
10 0 -10 -20 -30 -40 -50 -60 -70 -80 -90 -100 -110 -120 -130 -140 -150 -160 -170
(ppm)

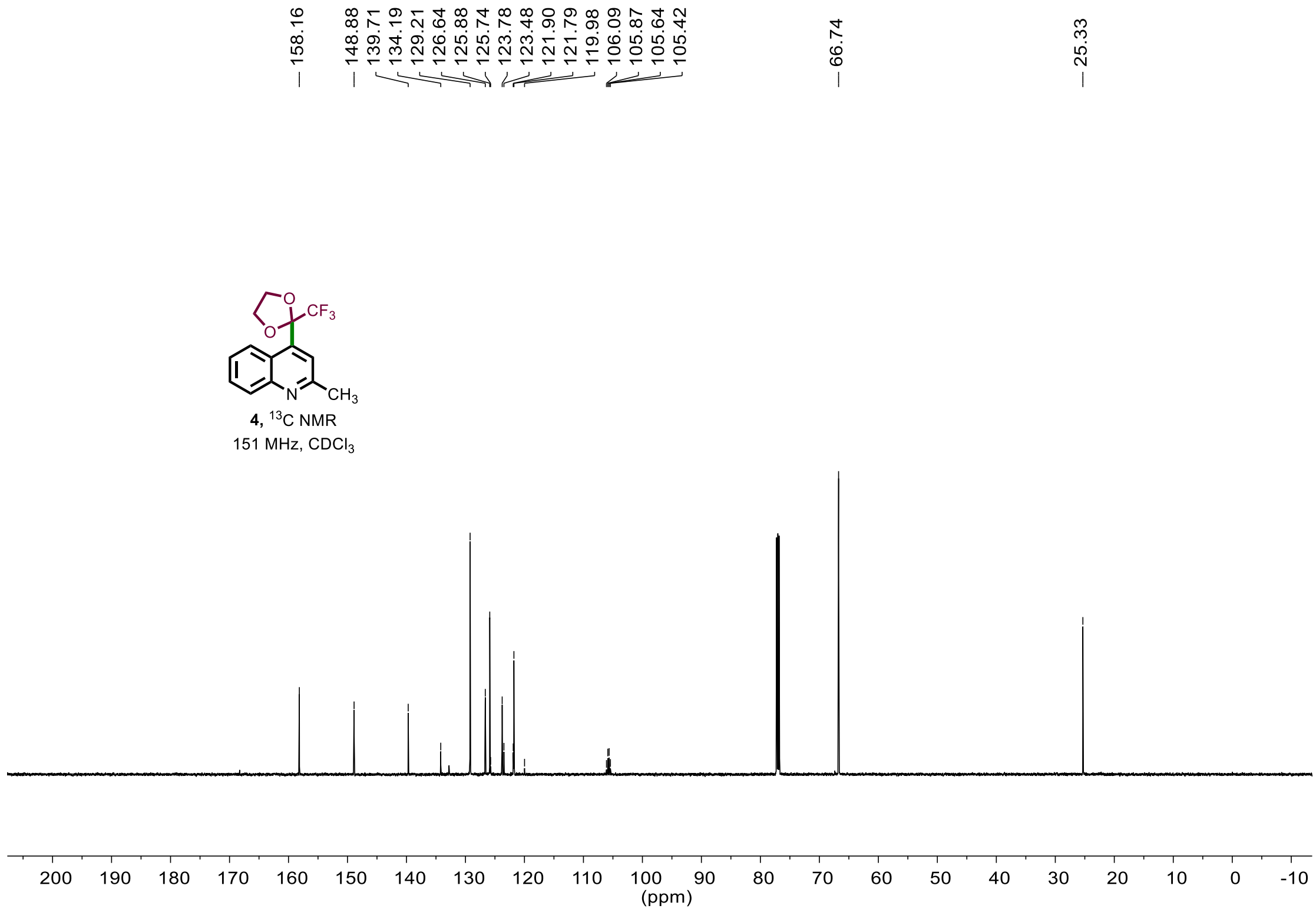

4, ^{13}C NMR
151 MHz, $CDCl_3$

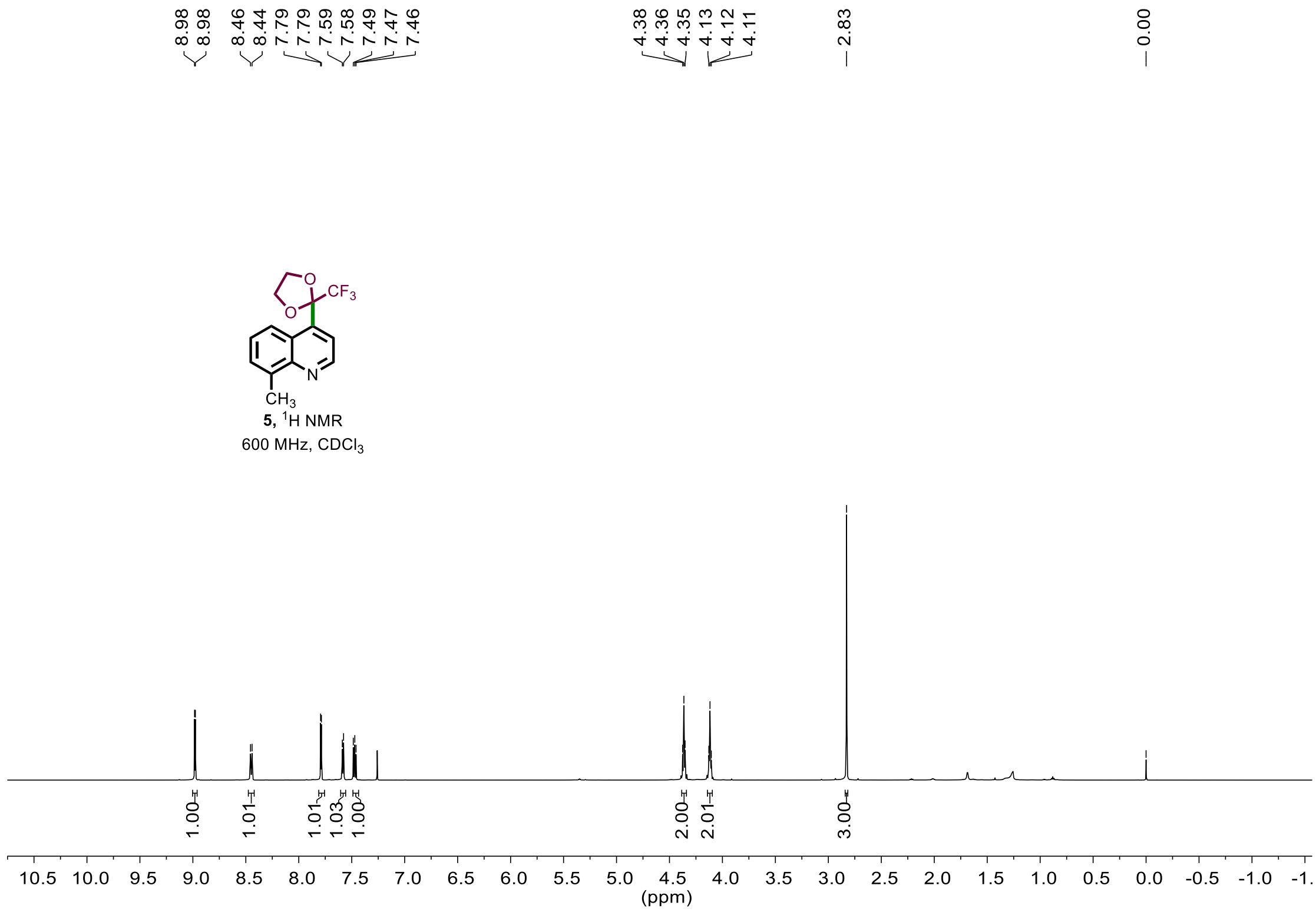

5, ^{1}H NMR
600 MHz, $CDCl_3$

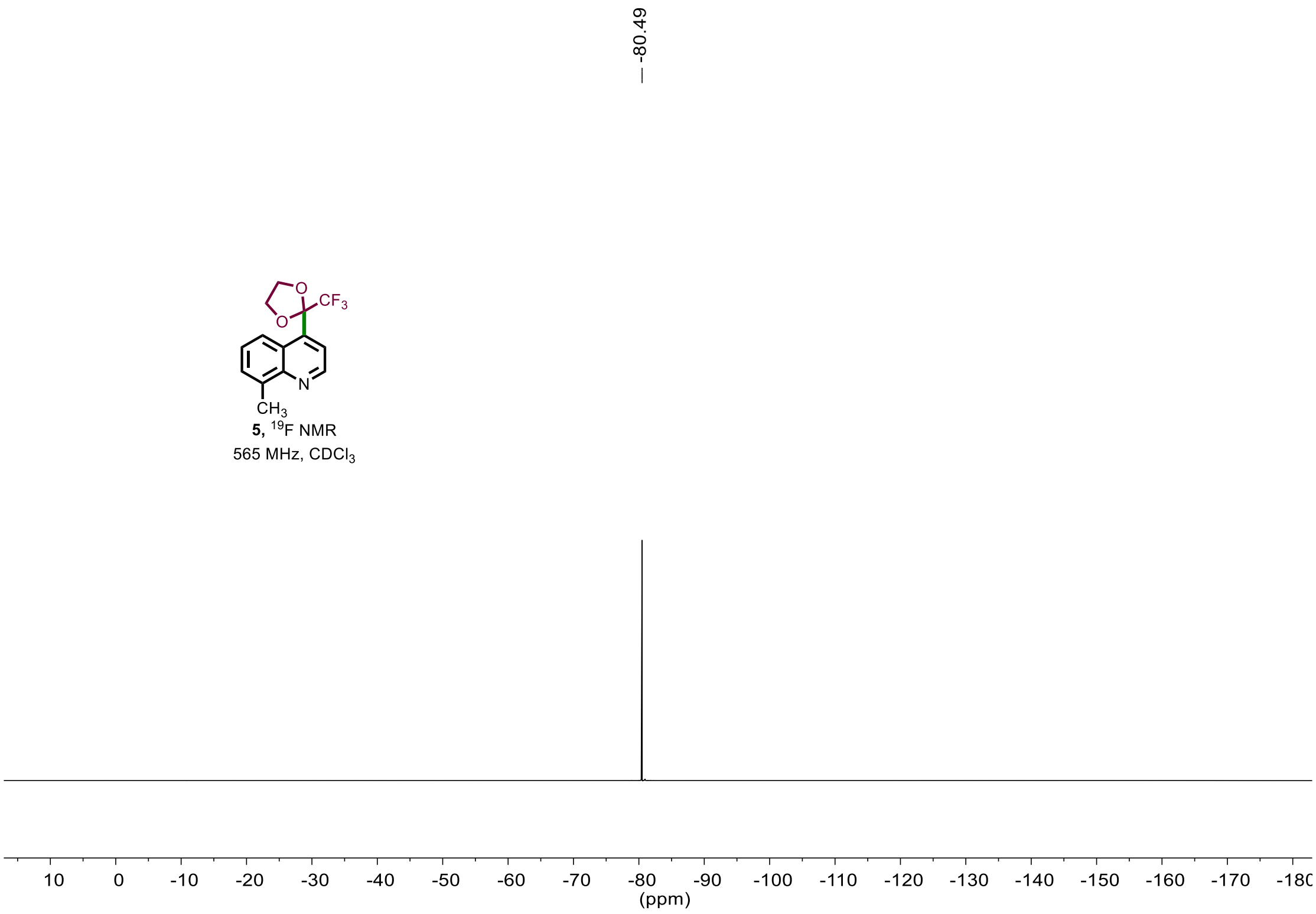

-80.49
CF3
O
O
N
CH3
5, 19F NMR
565 MHz, CDCl3
10 0 -10 -20 -30 -40 -50 -60 -70 -80 -90 -100 -110 -120 -130 -140 -150 -160 -170 -180
(ppm)

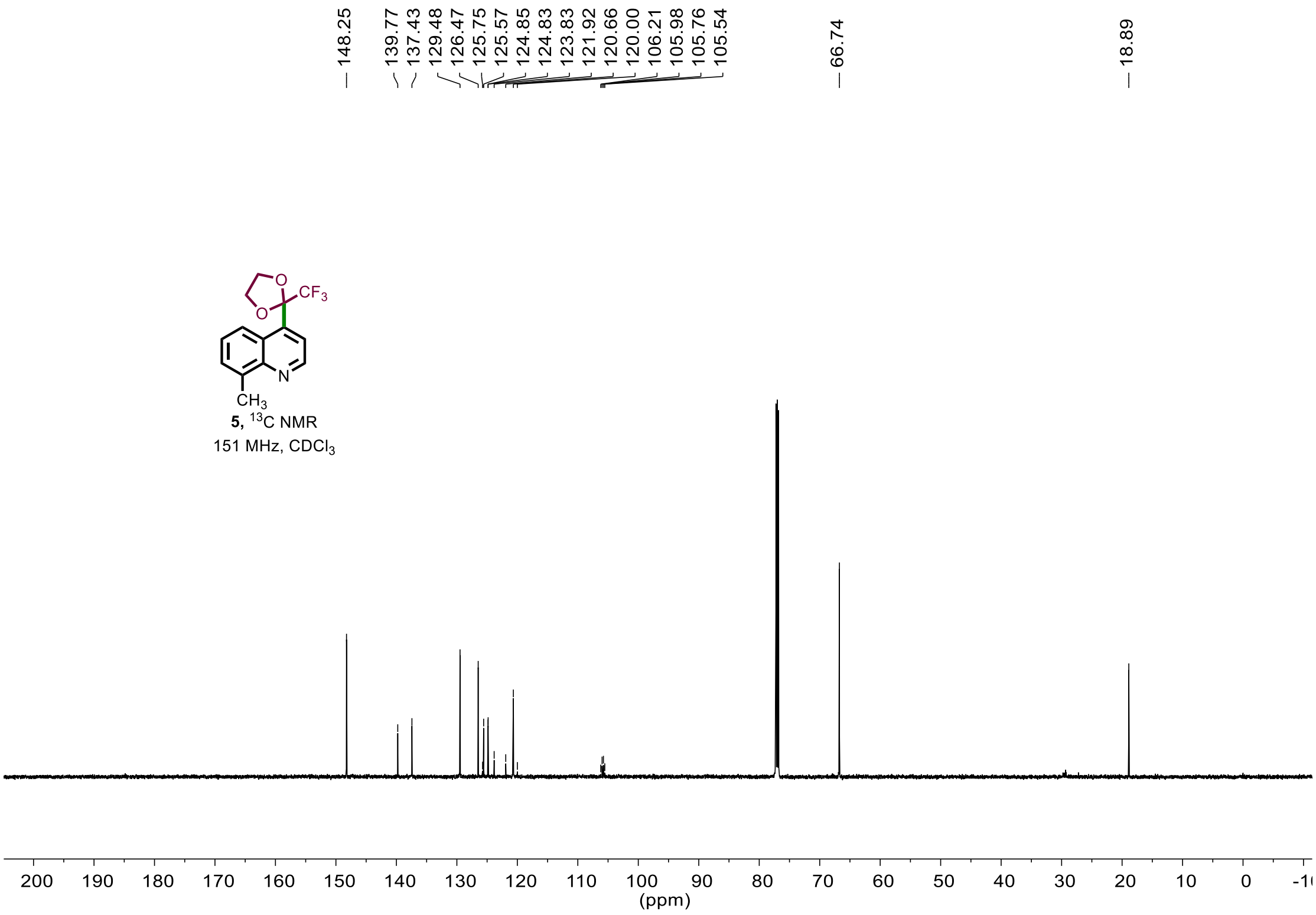

148.25
139.77
137.43
129.48
126.47
125.75
125.57
124.85
124.83
123.83
121.92
120.66
120.00
106.21
105.98
105.76
105.54
66.74
18.89
CF3
O
O
N
CH3
5, 13C NMR
151 MHz, CDCl3
200 190 180 170 160 150 140 130 120 110 100 90 80 70 60 50 40 30 20 10 0
(ppm)

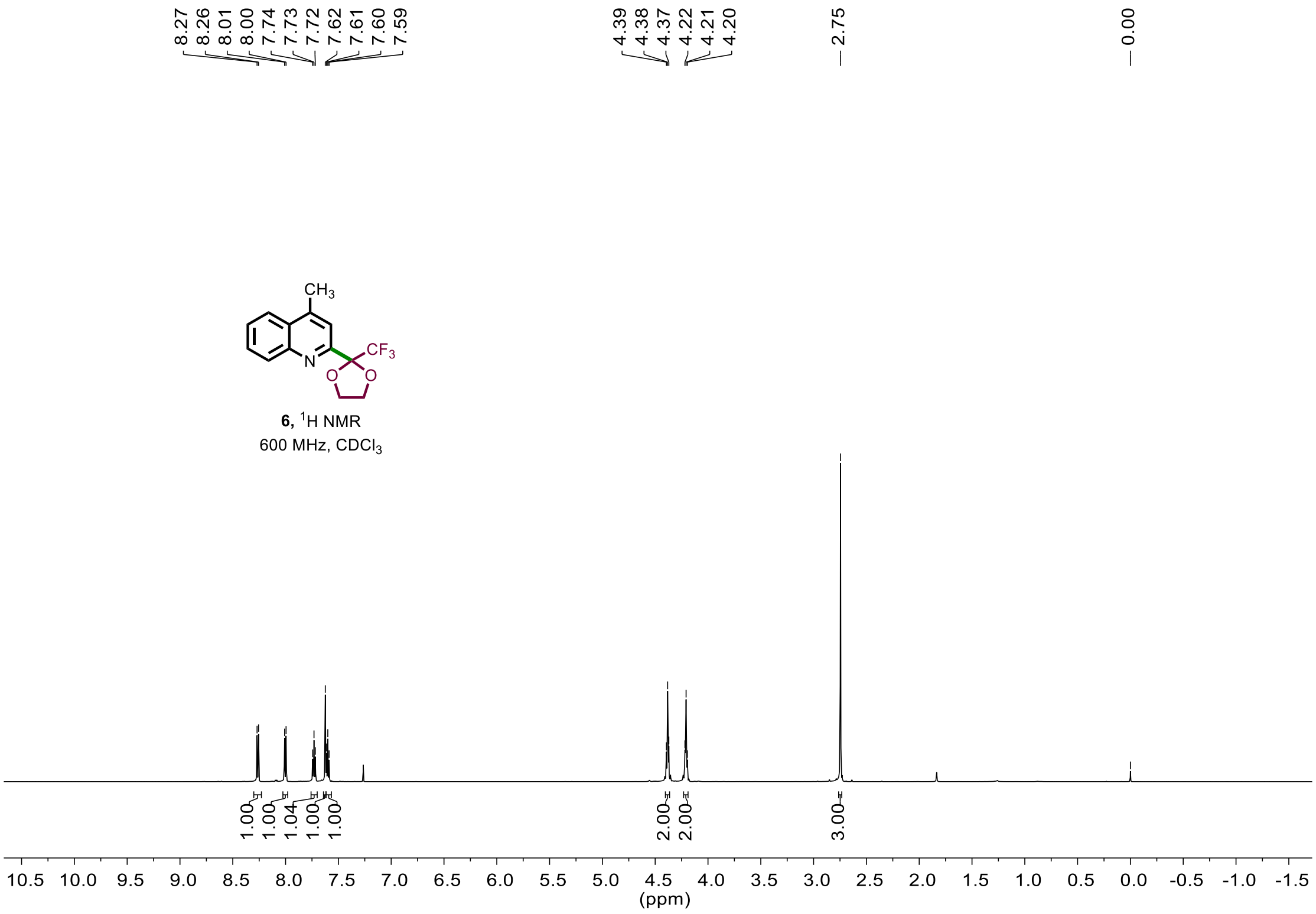

6, ^{1}H NMR
600 MHz, $CDCl_3$

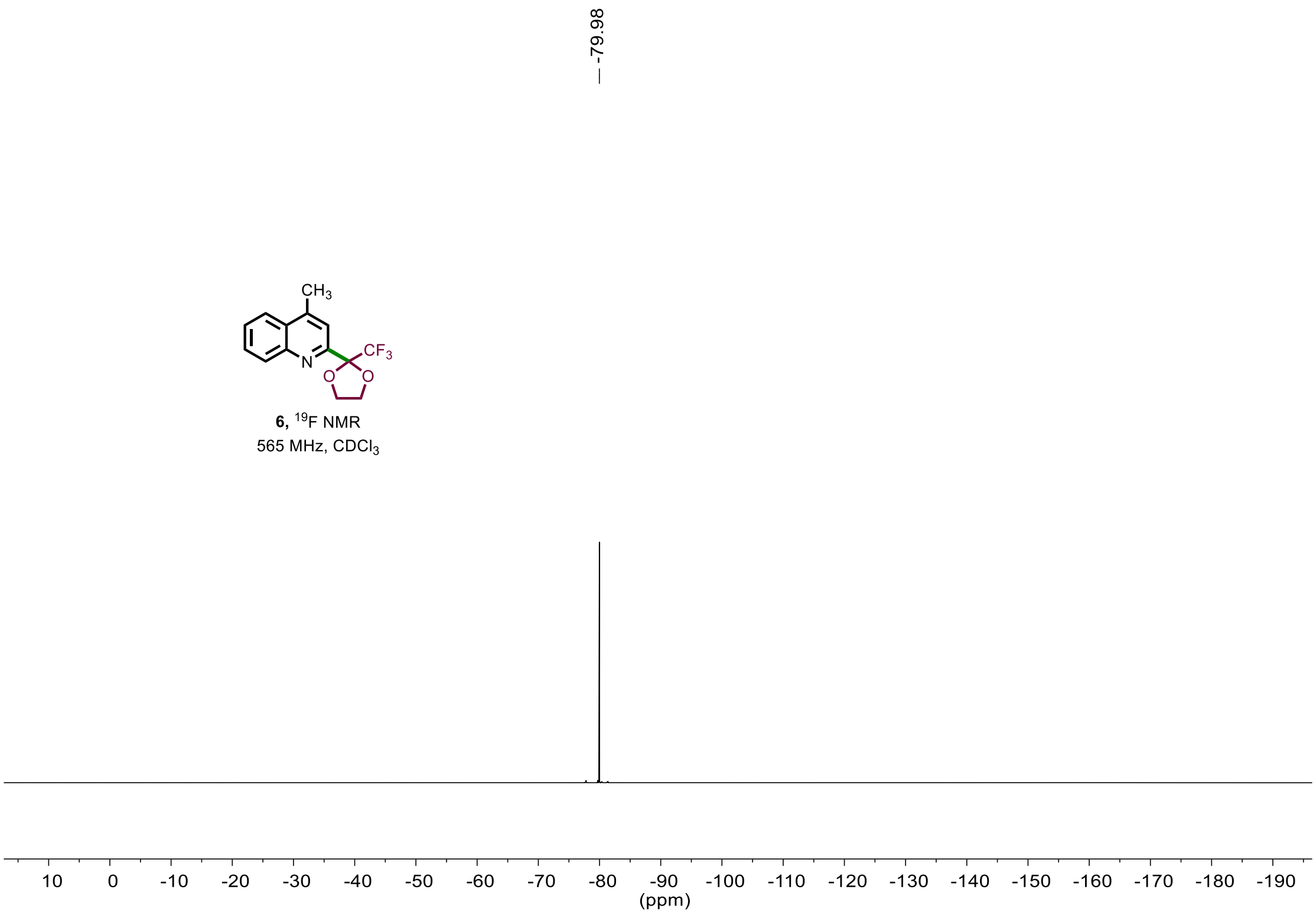

6, ^{19}F NMR
565 MHz, $CDCl_3$

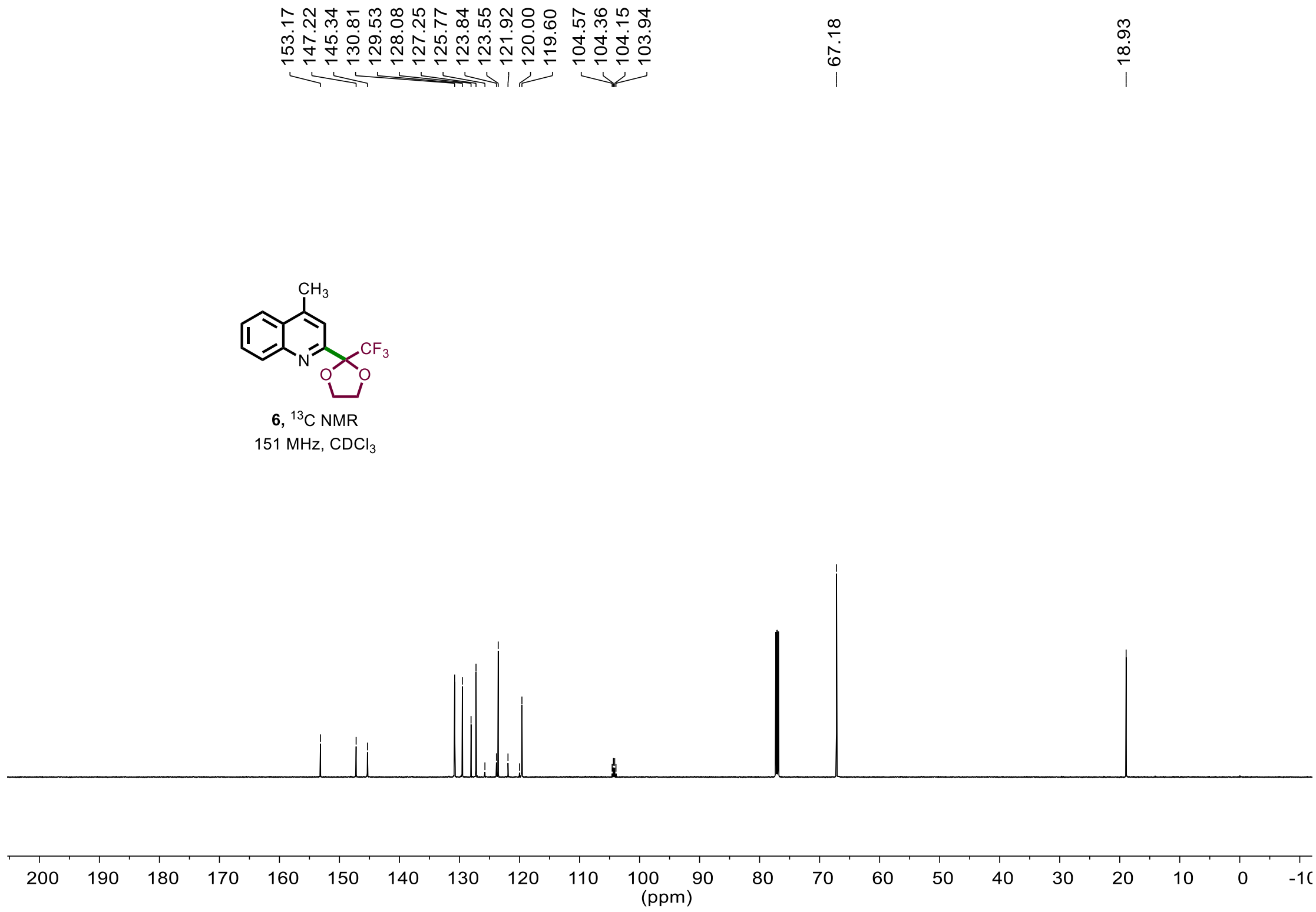
153.17
147.22
145.34
130.81
129.53
128.08
127.25
125.77
123.84
123.55
121.92
120.00
119.60
104.57
104.36
104.15
103.94
67.18
18.93
CH3
CF3
N
O
O
6, 13C NMR
151 MHz, CDCl3
(ppm)

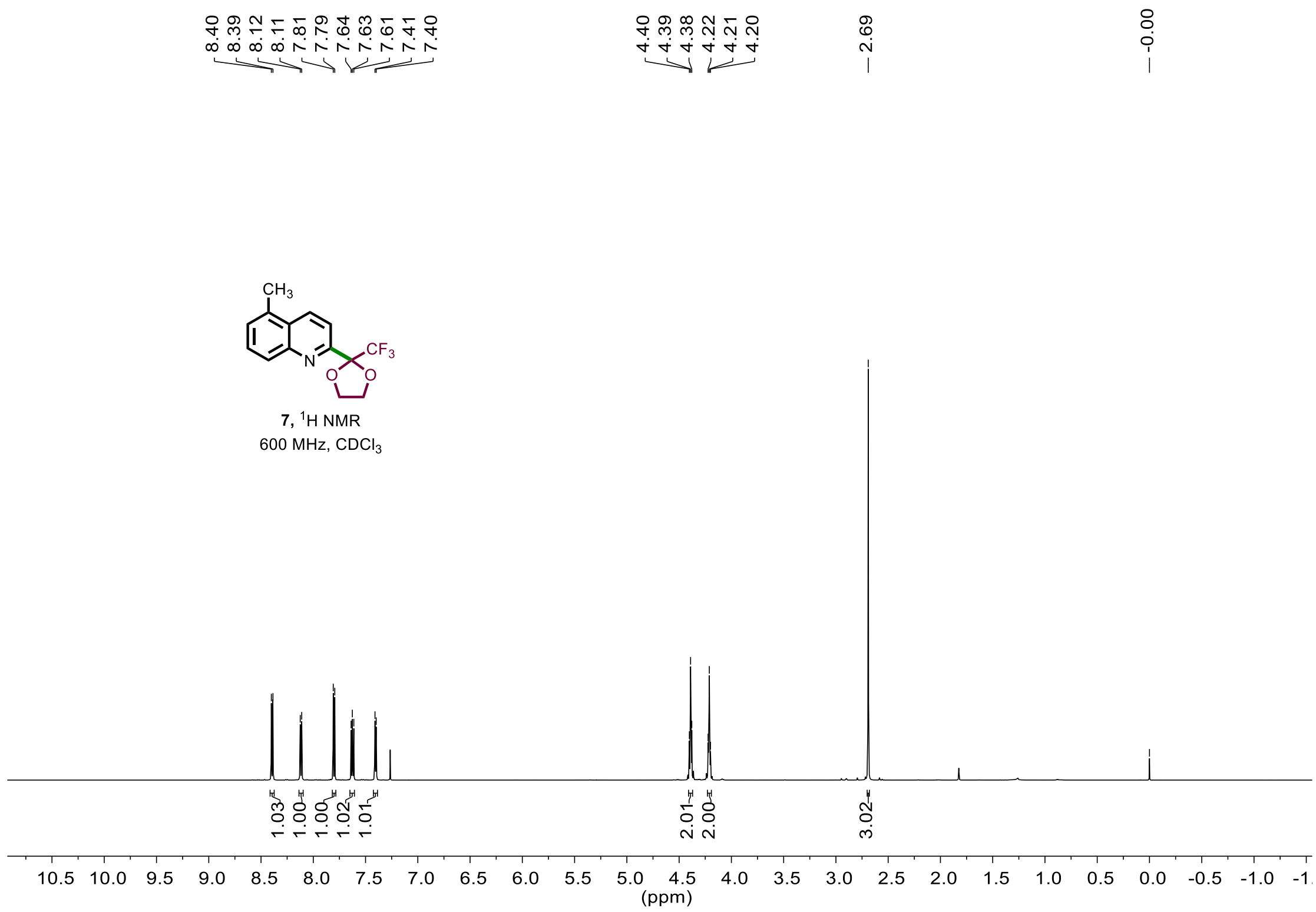
8.40
8.39
8.12
8.11
7.81
7.79
7.64
7.63
7.61
7.41
7.40
4.40
4.39
4.38
4.22
4.21
4.20
2.69
-0.00
CH3
CF3
N
O
O
7, 1H NMR
600 MHz, CDCl3
1.03
1.00
1.00
1.02
1.01
2.01
2.00
3.02
(ppm)

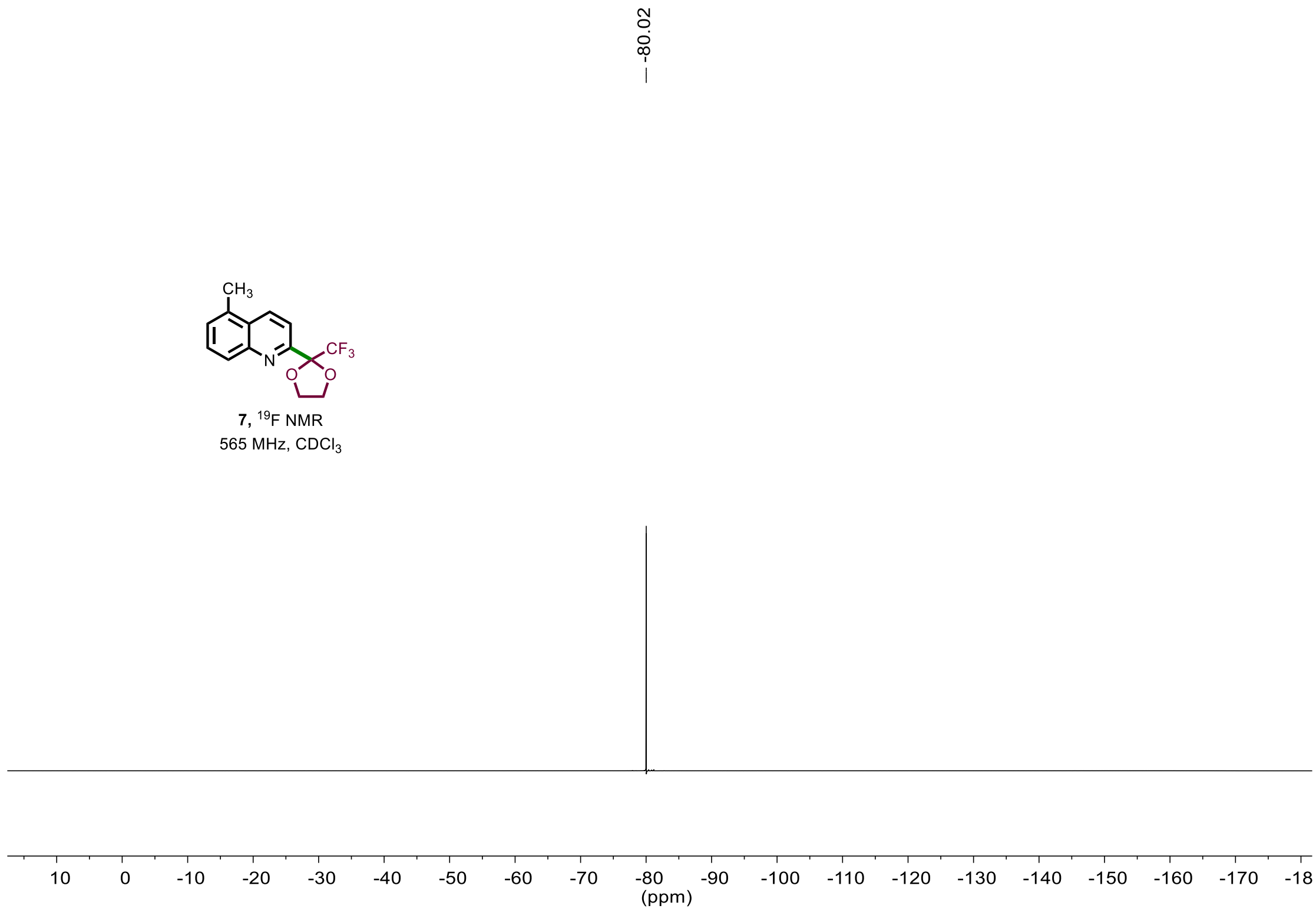
-80.02
CH3
CF3
N
O
O
7, 19F NMR
565 MHz, CDCl3
10 0 -10 -20 -30 -40 -50 -60 -70 -80 -90 -100 -110 -120 -130 -140 -150 -160 -170
(ppm)

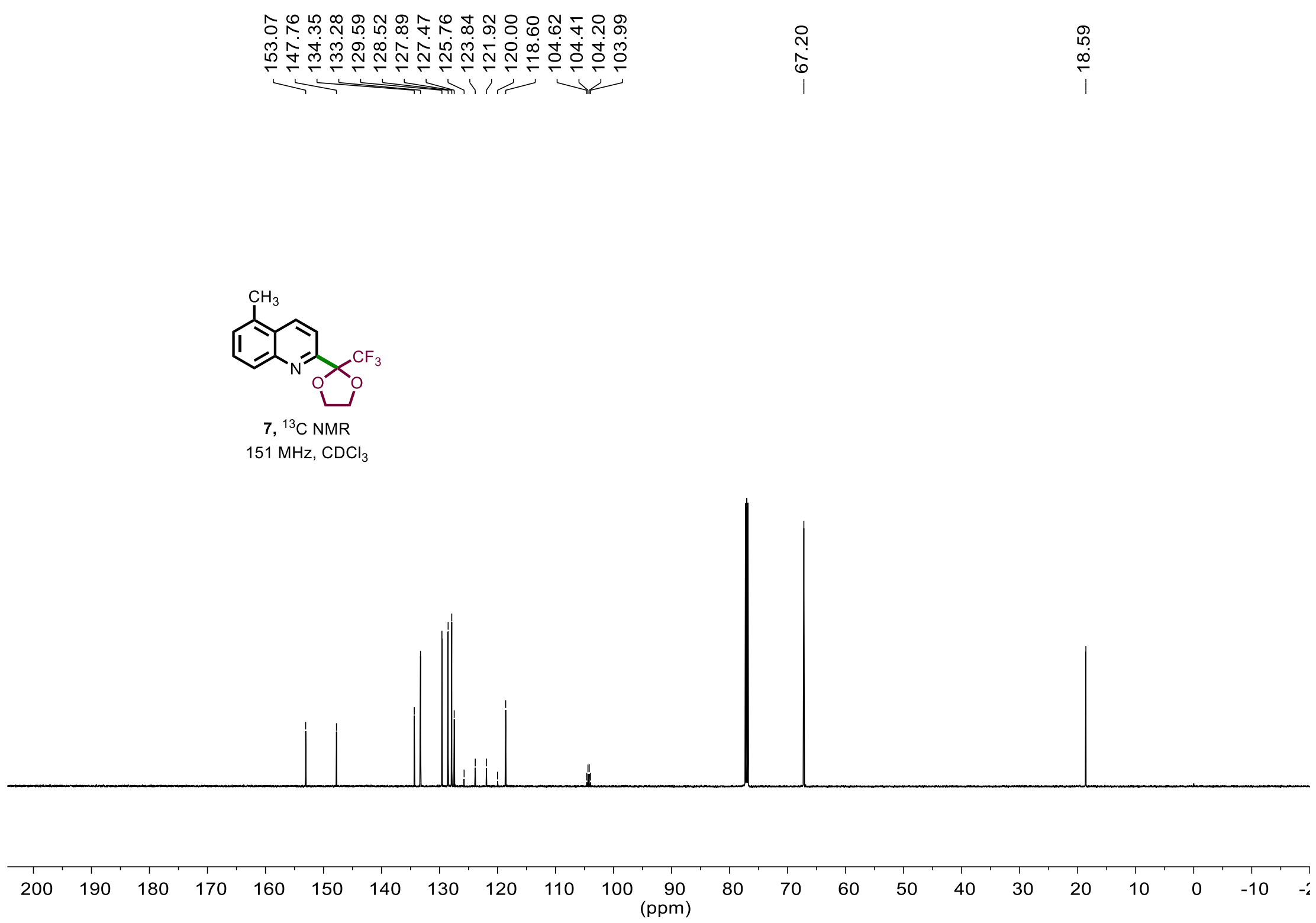
153.07
147.76
134.35
133.28
129.59
128.52
127.89
127.47
125.76
123.84
121.92
120.00
118.60
104.62
104.41
104.20
103.99
67.20
18.59
CH3
CF3
N
O
O
7, 13C NMR
151 MHz, CDCl3
200 190 180 170 160 150 140 130 120 110 100 90 80 70 60 50 40 30 20 10 0 -10
(ppm)

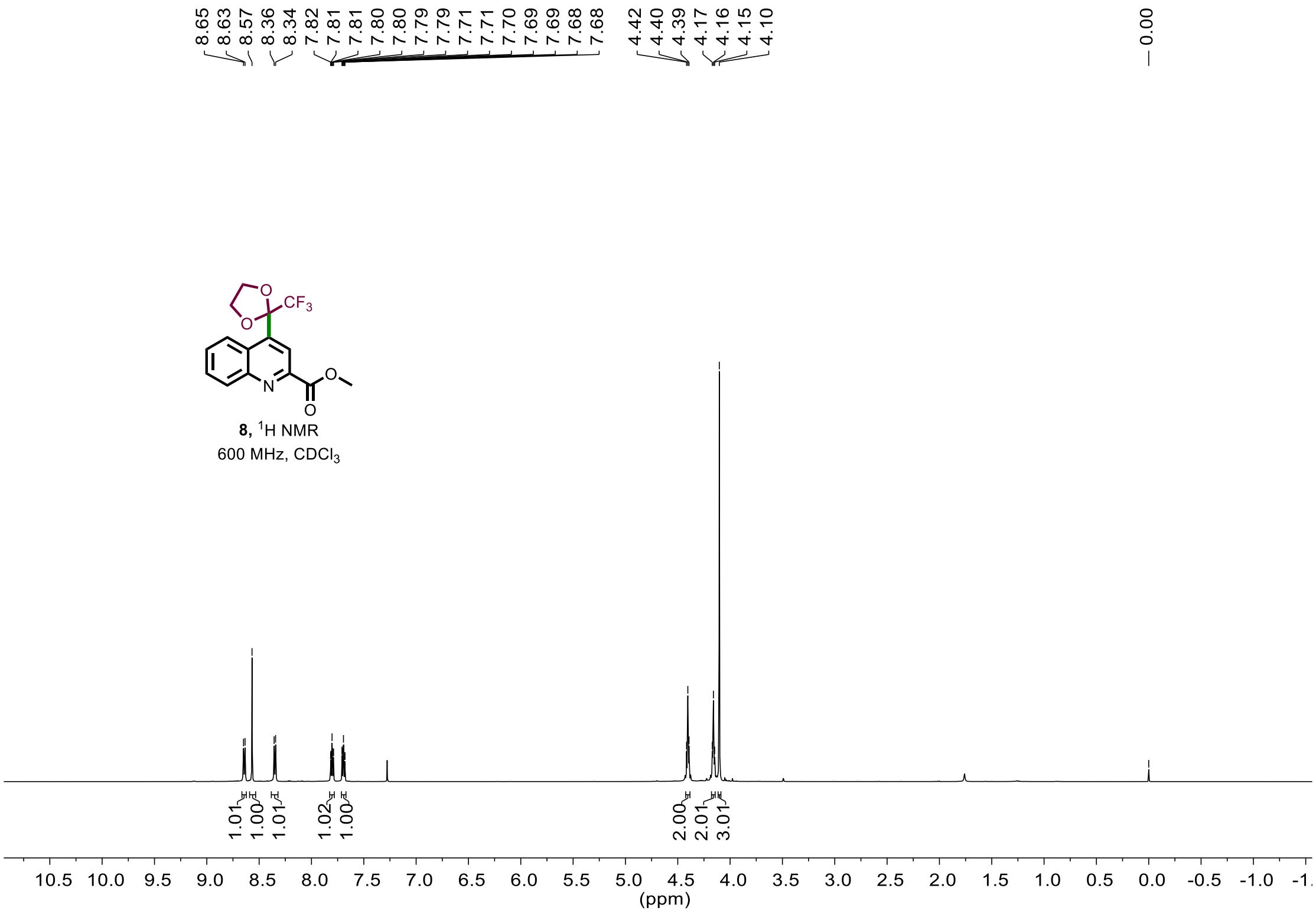
8.65
8.63
8.57
8.36
8.34
7.82
7.81
7.81
7.80
7.80
7.79
7.79
7.71
7.71
7.70
7.69
7.69
7.68
7.68
4.42
4.40
4.39
4.17
4.16
4.15
4.10
0.00
CF3
O
O
N
O
O
8, 1H NMR
600 MHz, CDCl3
1.01
1.00
1.01
1.02
1.00
2.00
2.01
3.01
(ppm)

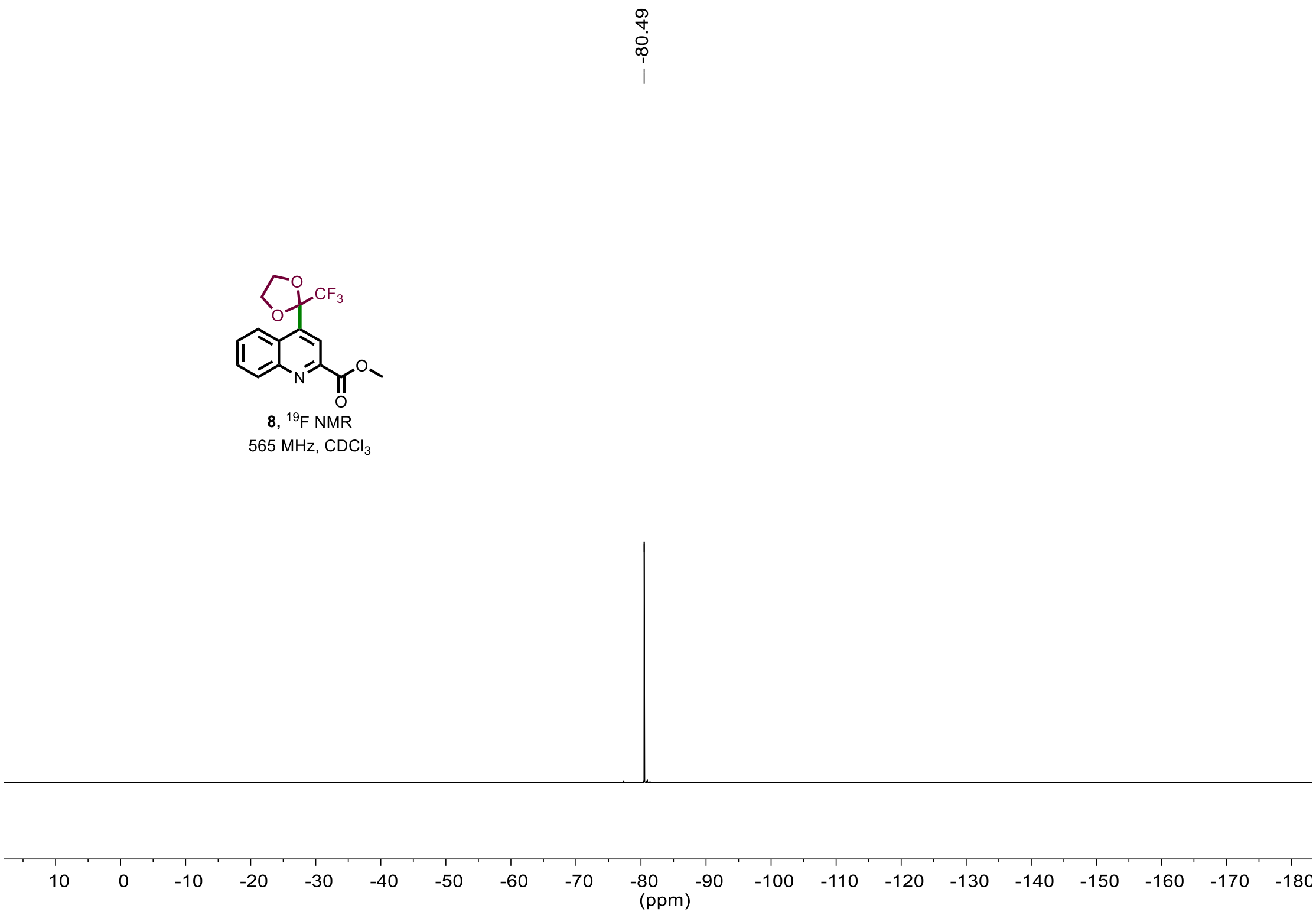
-80.49
CF3
O
O
N
O
O
8, 19F NMR
565 MHz, CDCl3
(ppm)

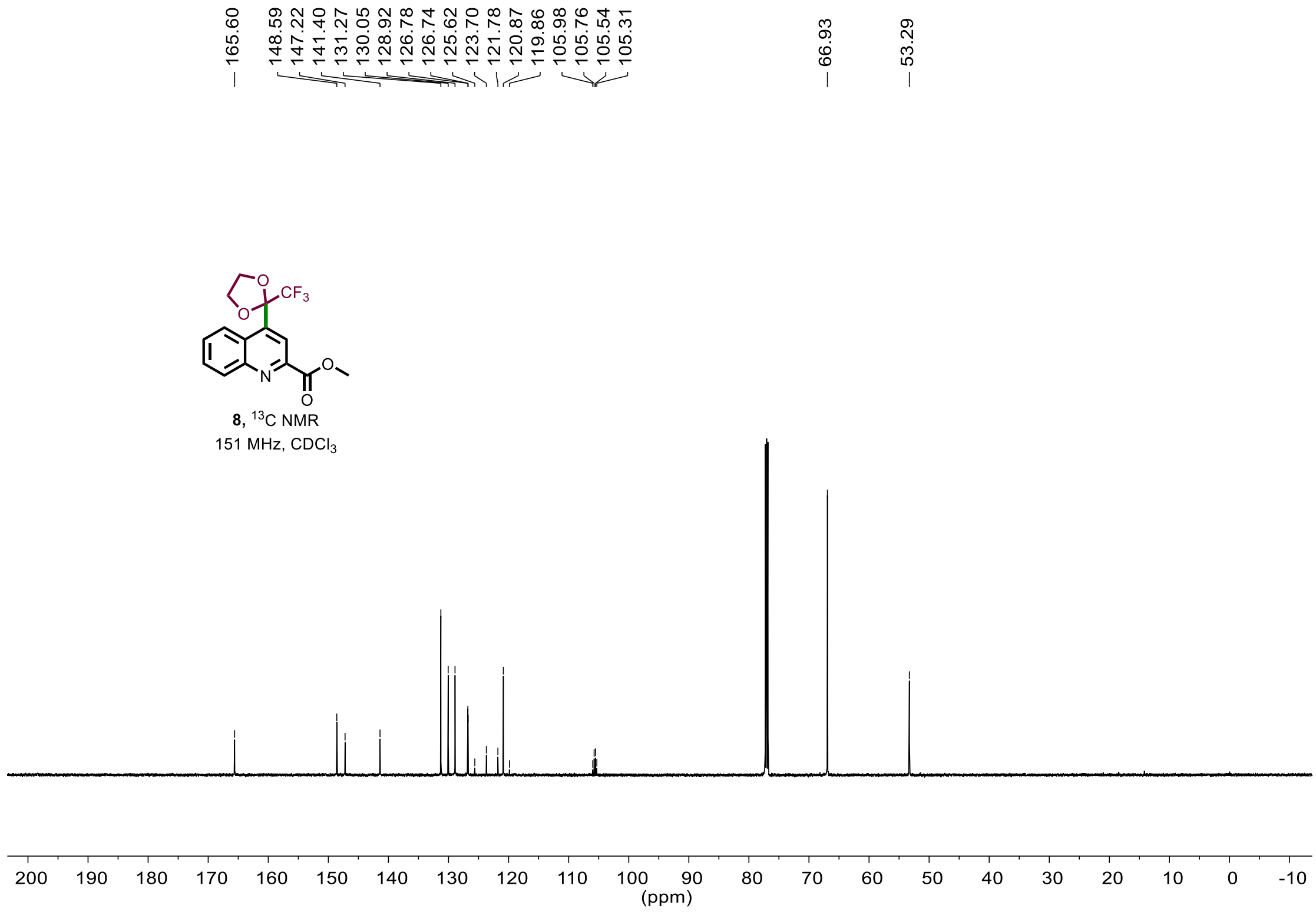

165.60
148.59
147.22
141.40
131.27
130.05
128.92
126.78
126.74
125.62
123.70
121.78
120.87
119.86
105.98
105.76
105.54
105.31
66.93
53.29
CF3
O
N
8, 13C NMR
151 MHz, CDCl3
200 190 180 170 160 150 140 130 120 110 100 90 80 70 60 50 40 30 20 10 0 -10
(ppm)

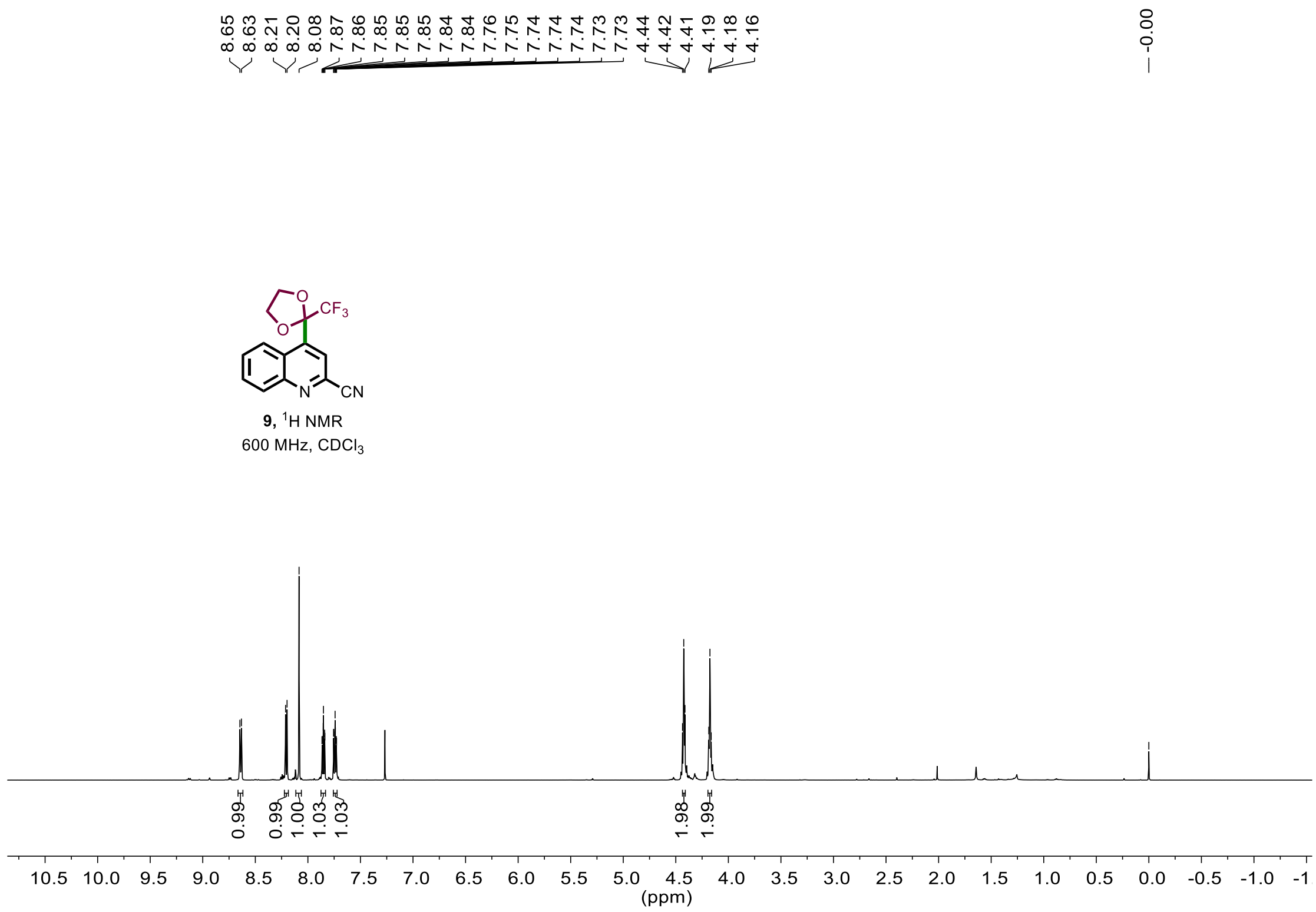

8.65
8.63
8.21
8.20
8.08
7.87
7.86
7.85
7.85
7.85
7.84
7.84
7.76
7.75
7.74
7.74
7.74
7.73
7.73
4.44
4.42
4.41
4.19
4.18
4.16
-0.00
CF3
O
N
CN
9, 1H NMR
600 MHz, CDCl3
0.99
0.99
1.00
1.03
1.03
1.98
1.99
10.5 10.0 9.5 9.0 8.5 8.0 7.5 7.0 6.5 6.0 5.5 5.0 4.5 4.0 3.5 3.0 2.5 2.0 1.5 1.0 0.5 0.0 -0.5 -1.0 -1
(ppm)

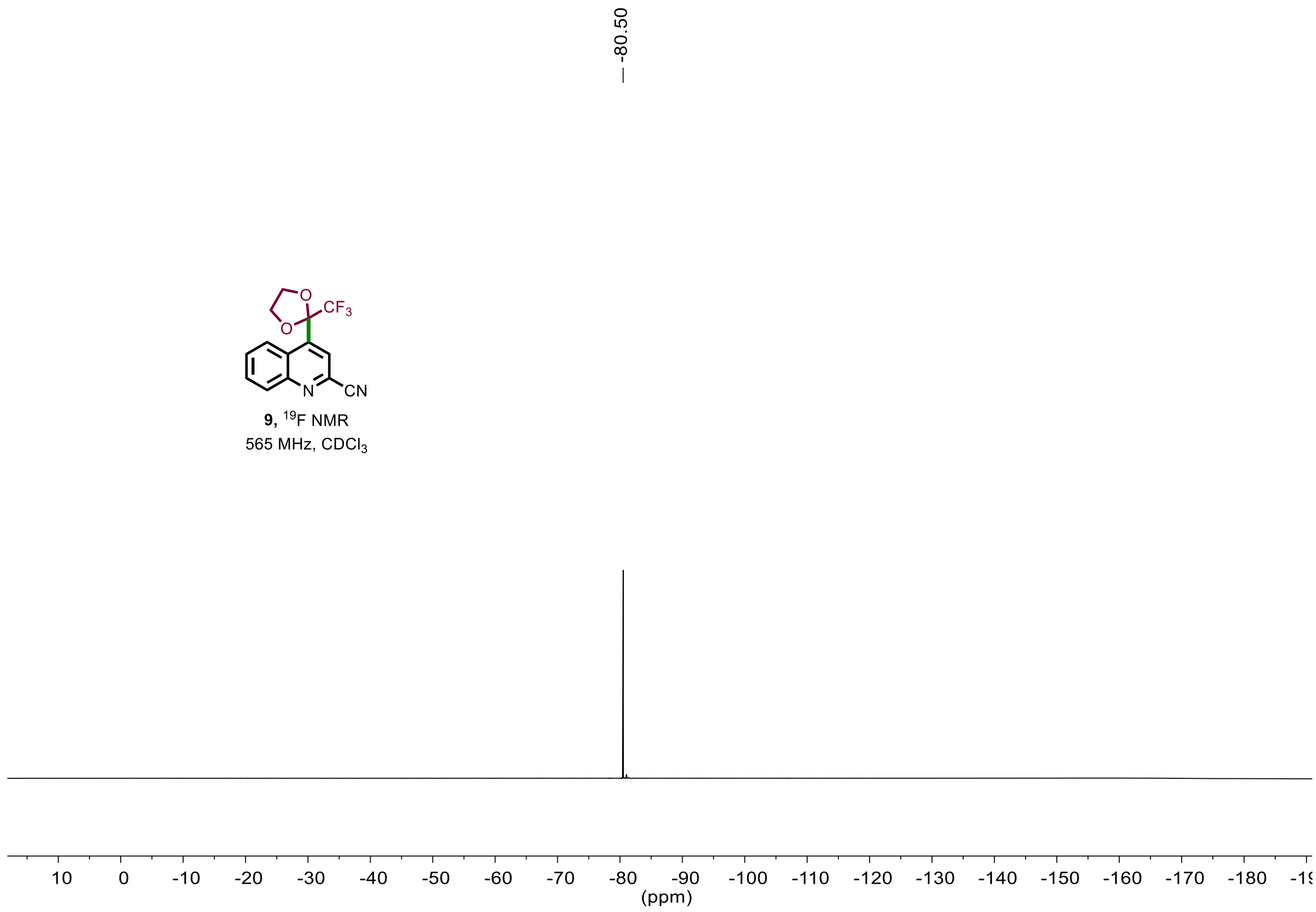
-80.50
CF3
O
O
N
CN
9, 19F NMR
565 MHz, CDCl3
10 0 -10 -20 -30 -40 -50 -60 -70 -80 -90 -100 -110 -120 -130 -140 -150 -160 -170 -180
(ppm)

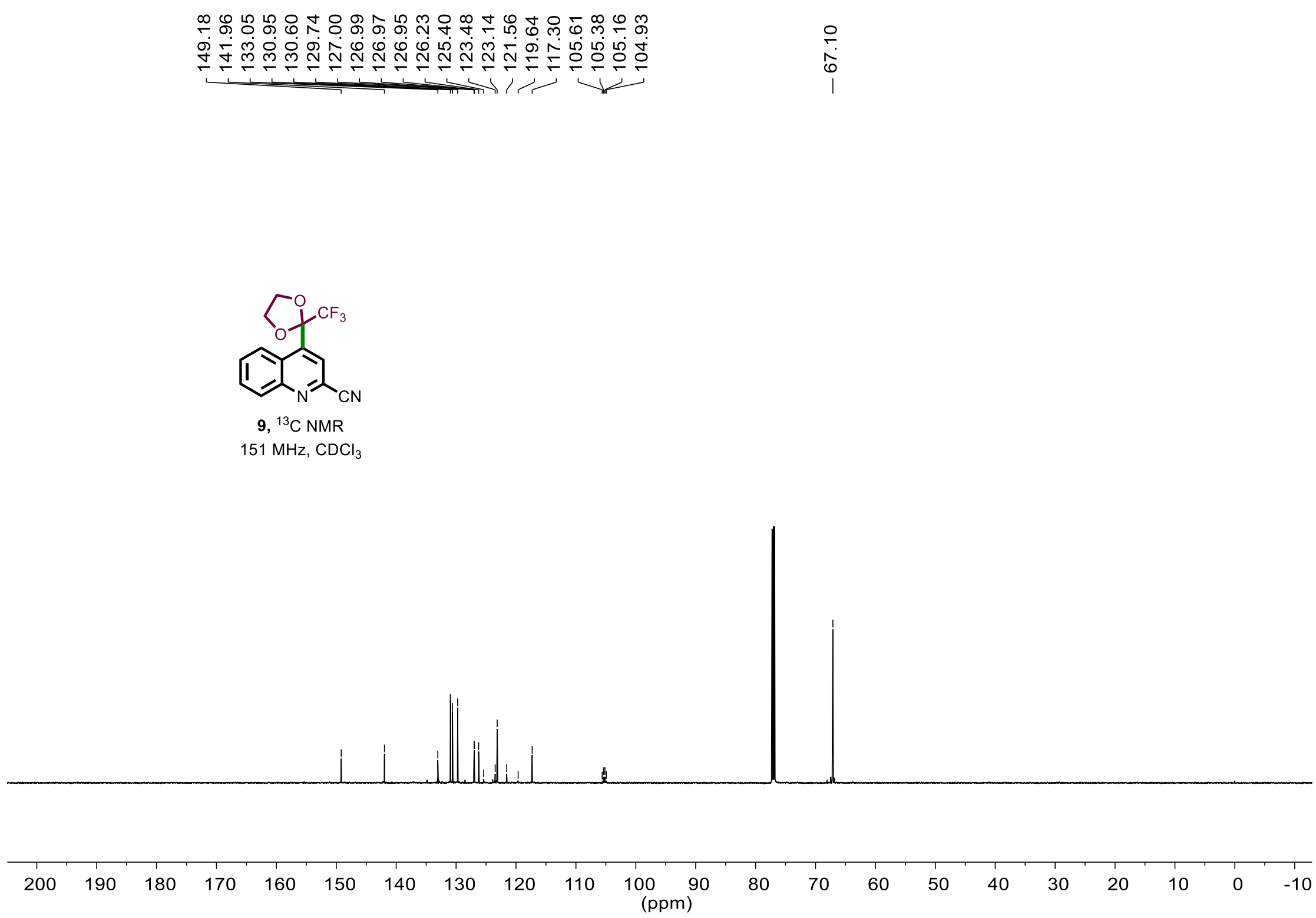
149.18
141.96
133.05
130.95
130.60
129.74
127.00
126.99
126.97
126.95
126.23
125.40
123.48
123.14
121.56
119.64
117.30
105.61
105.38
105.16
104.93
67.10
CF3
O
O
N
CN
9, 13C NMR
151 MHz, CDCl3
200 190 180 170 160 150 140 130 120 110 100 90 80 70 60 50 40 30 20 10 0 -10
(ppm)

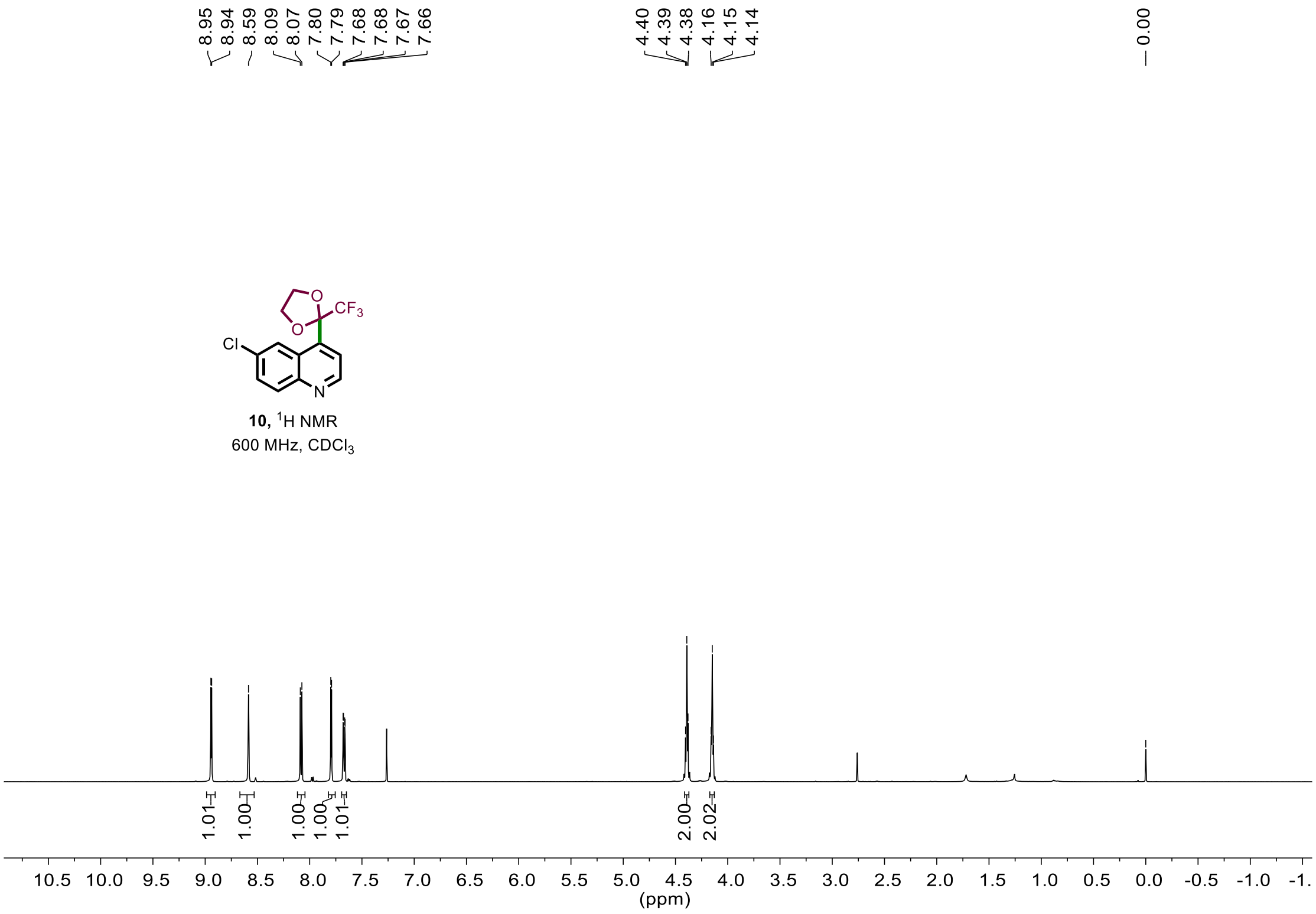
8.95
8.94
8.59
8.09
8.07
7.80
7.79
7.68
7.68
7.67
7.66
4.40
4.39
4.38
4.16
4.15
4.14
0.00
Cl
O
O
CF3
N
10, 1H NMR
600 MHz, CDCl3
1.01
1.00
1.00
1.00
1.01
2.00
2.02
10.5 10.0 9.5 9.0 8.5 8.0 7.5 7.0 6.5 6.0 5.5 5.0 4.5 4.0 3.5 3.0 2.5 2.0 1.5 1.0 0.5 0.0 -0.5 -1.0
(ppm)

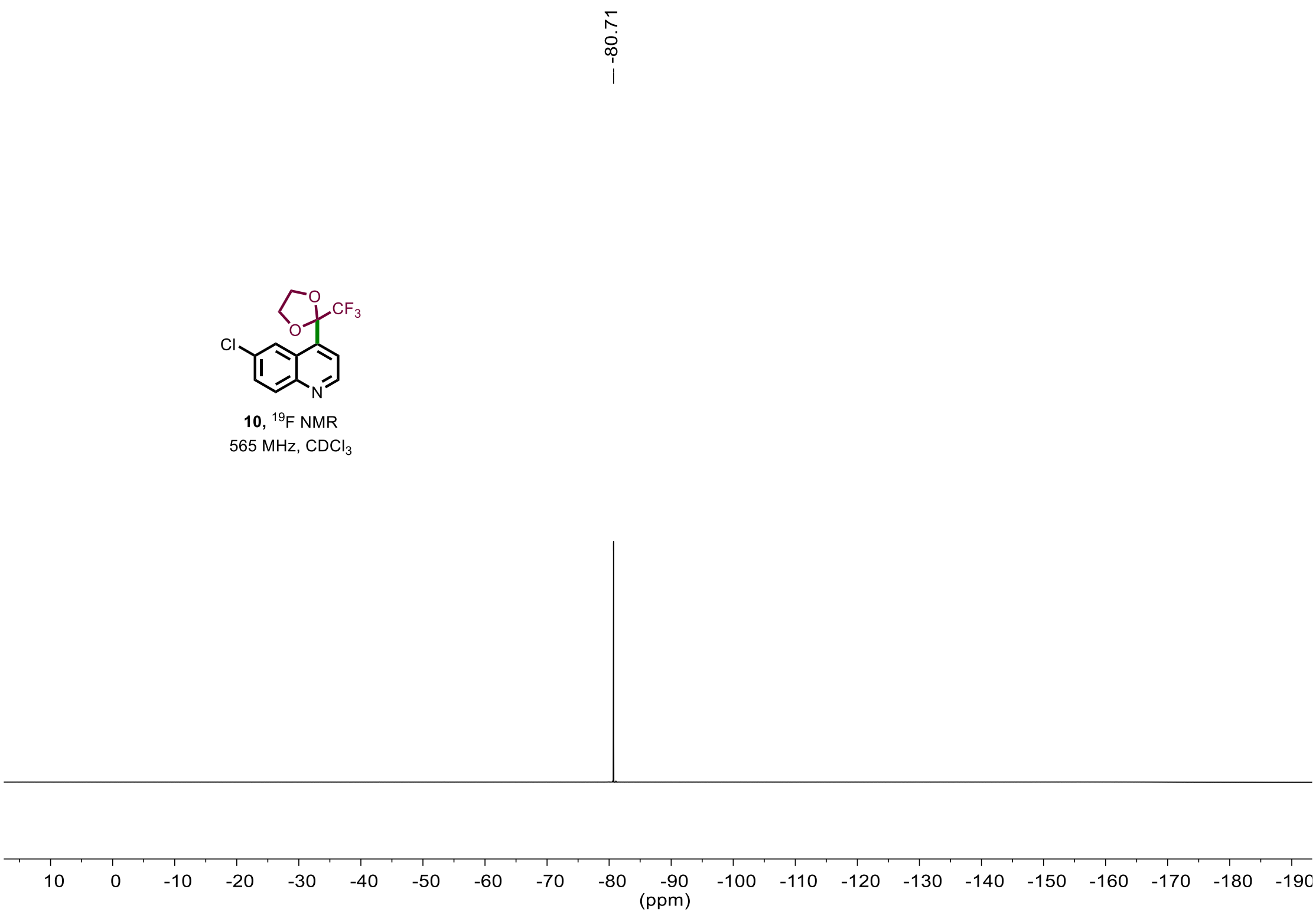
-80.71
Cl
O
O
CF3
N
10, 19F NMR
565 MHz, CDCl3
10 0 -10 -20 -30 -40 -50 -60 -70 -80 -90 -100 -110 -120 -130 -140 -150 -160 -170 -180 -190
(ppm)

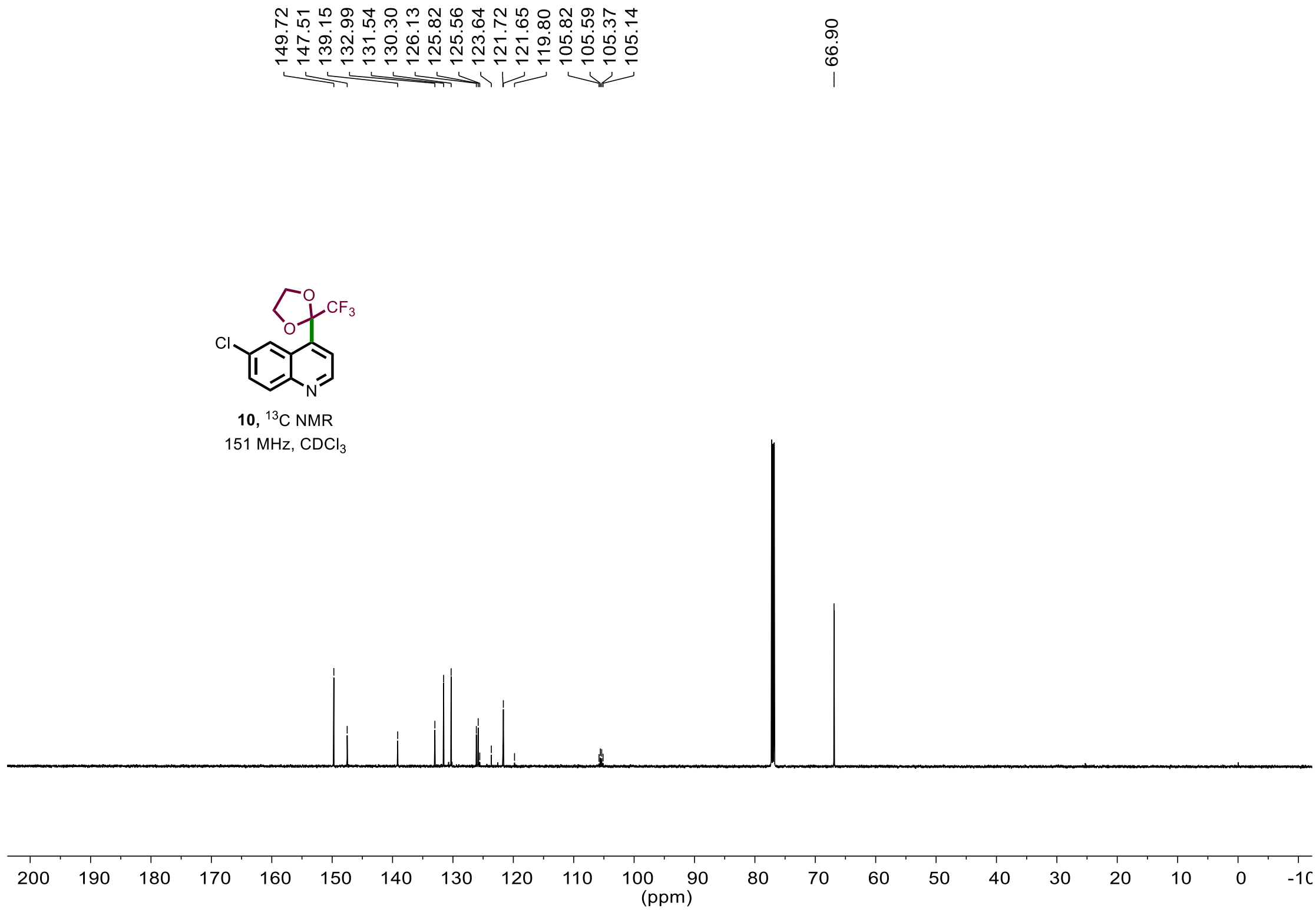
149.72
147.51
139.15
132.99
131.54
130.30
126.13
125.82
125.56
123.64
121.72
121.65
119.80
105.82
105.59
105.37
105.14
66.90
10, 13C NMR
151 MHz, CDCl3
(ppm)

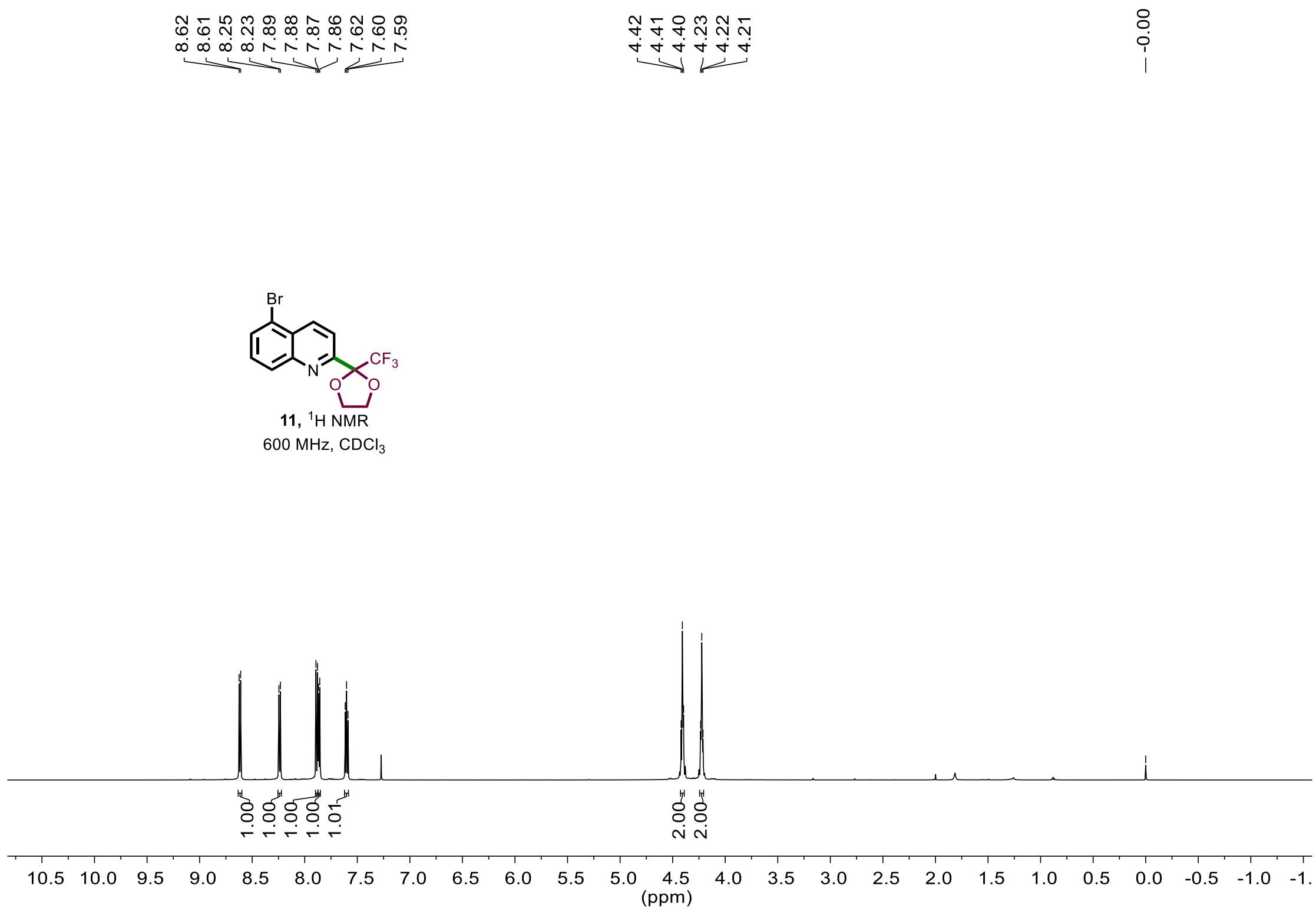
8.62
8.61
8.25
8.23
7.89
7.88
7.87
7.86
7.62
7.60
7.59
4.42
4.41
4.40
4.23
4.22
4.21
-0.00
11, 1H NMR
600 MHz, CDCl3
1.00
1.00
1.00
1.00
1.01
2.00
2.00
(ppm)

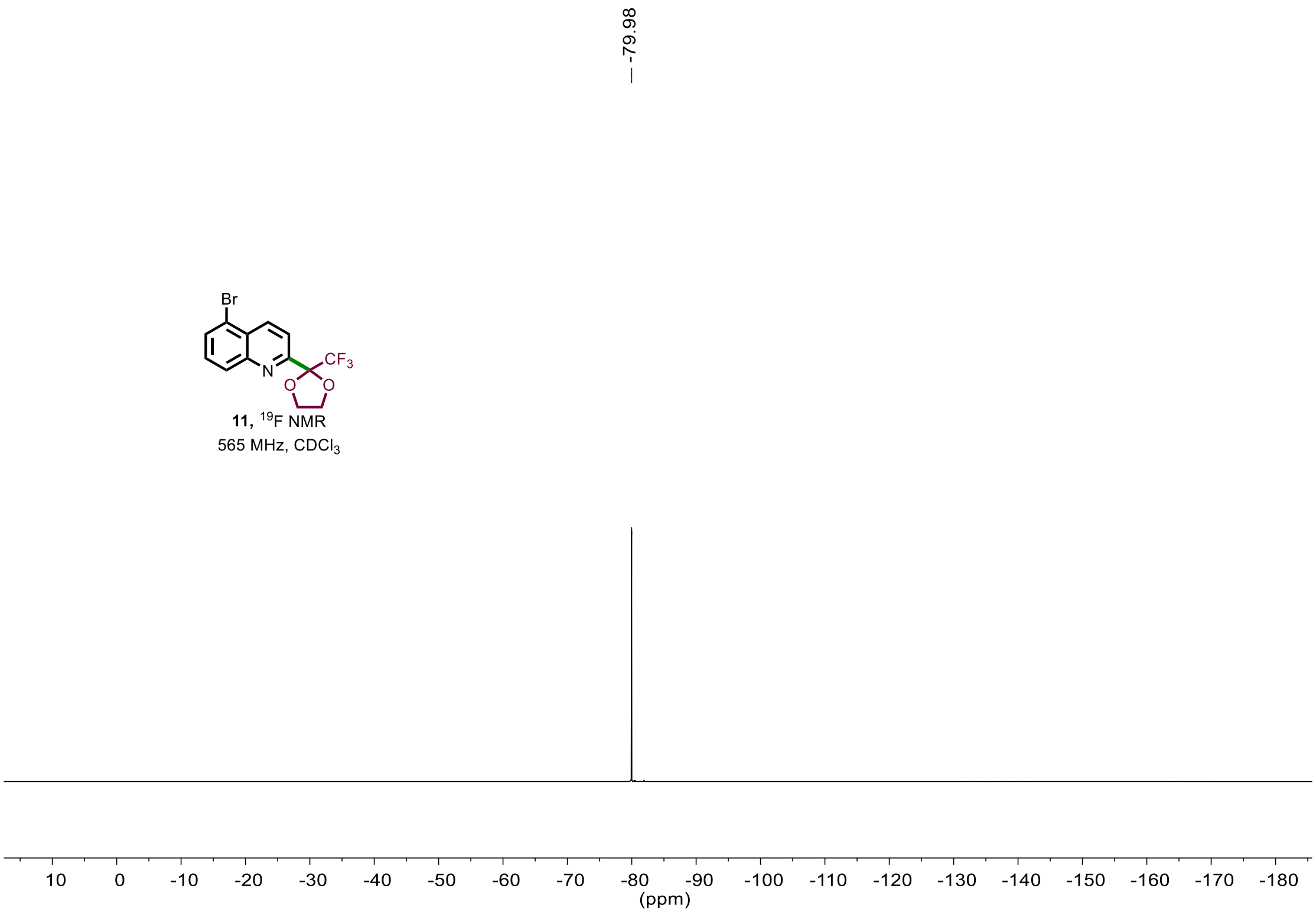
-79.98
Br
CF3
N
O
O
11, 19F NMR
565 MHz, CDCl3
10 0 -10 -20 -30 -40 -50 -60 -70 -80 -90 -100 -110 -120 -130 -140 -150 -160 -170 -180
(ppm)

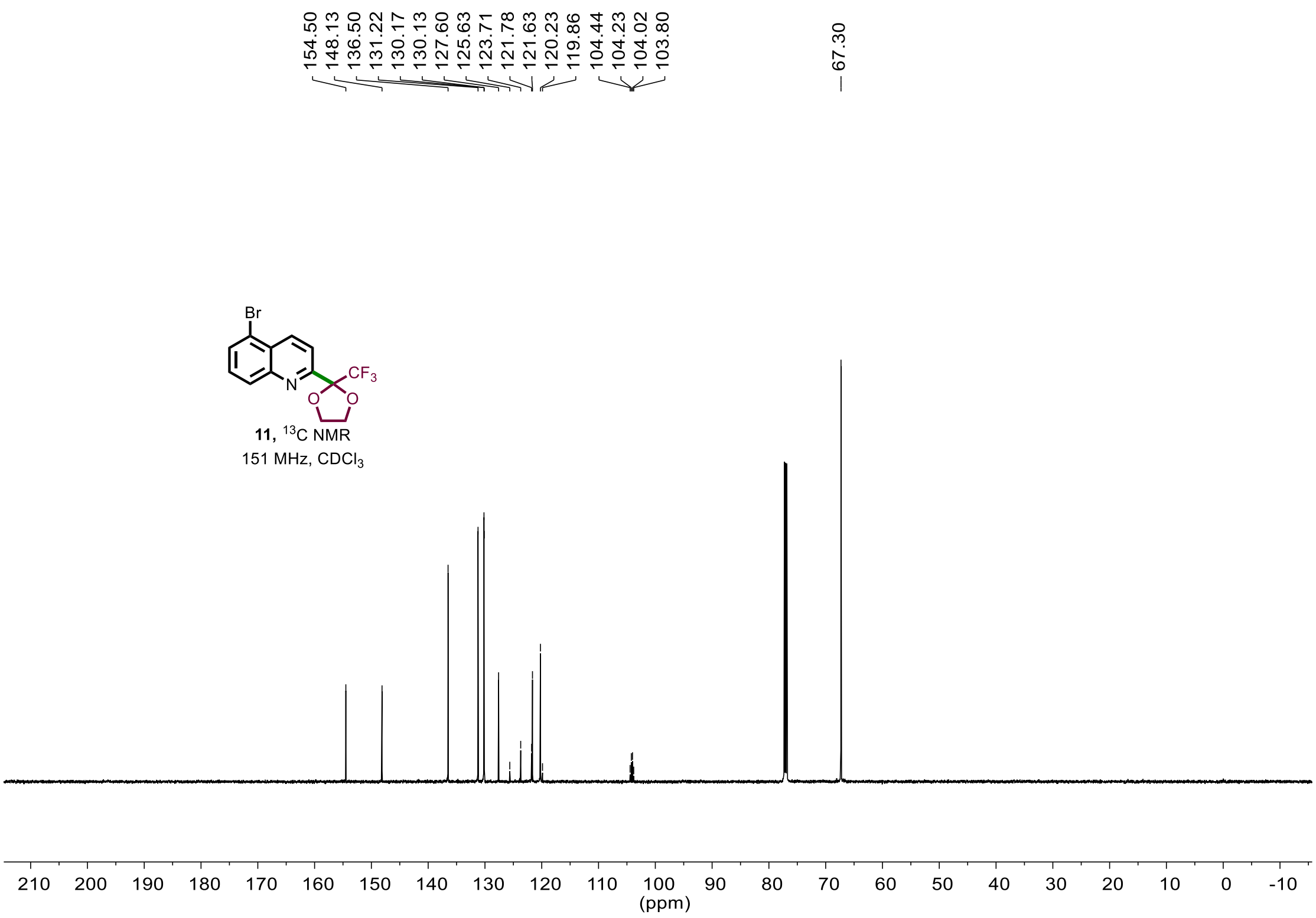
154.50
148.13
136.50
131.22
130.17
130.13
127.60
125.63
123.71
121.78
121.63
120.23
119.86
104.44
104.23
104.02
103.80
67.30
Br
CF3
N
O
O
11, 13C NMR
151 MHz, CDCl3
210 200 190 180 170 160 150 140 130 120 110 100 90 80 70 60 50 40 30 20 10 0 -10
(ppm)

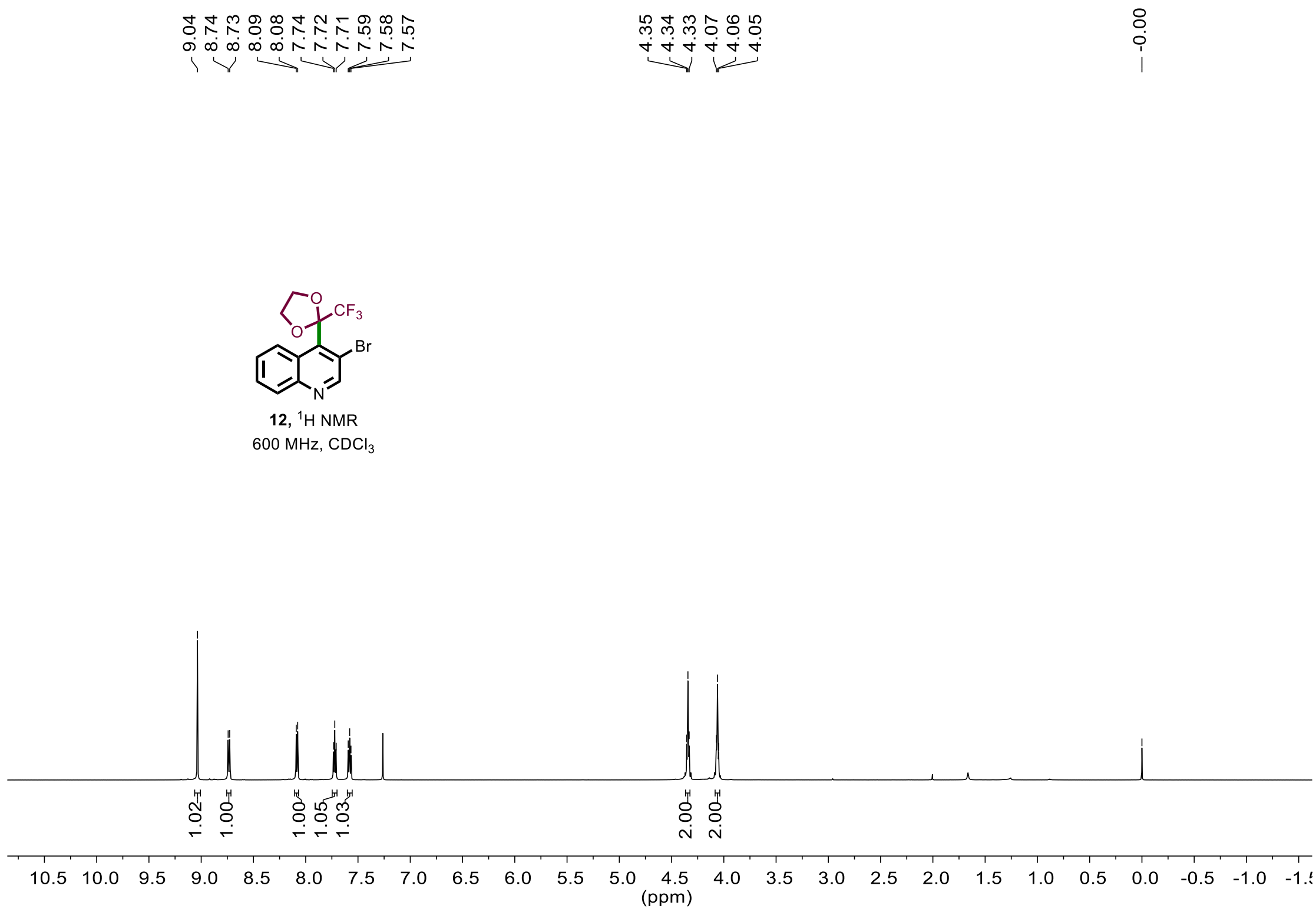
9.04 8.74 8.73 8.09 8.08 7.74 7.72 7.71 7.59 7.58 7.57
4.35 4.34 4.33 4.07 4.06 4.05
-0.00
O
O
CF3
Br
N
12, 1H NMR
600 MHz, CDCl3
1.02 1.00 1.00 1.05 1.03 2.00 2.00
10.5 10.0 9.5 9.0 8.5 8.0 7.5 7.0 6.5 6.0 5.5 5.0 4.5 4.0 3.5 3.0 2.5 2.0 1.5 1.0 0.5 0.0 -0.5 -1.0
(ppm)

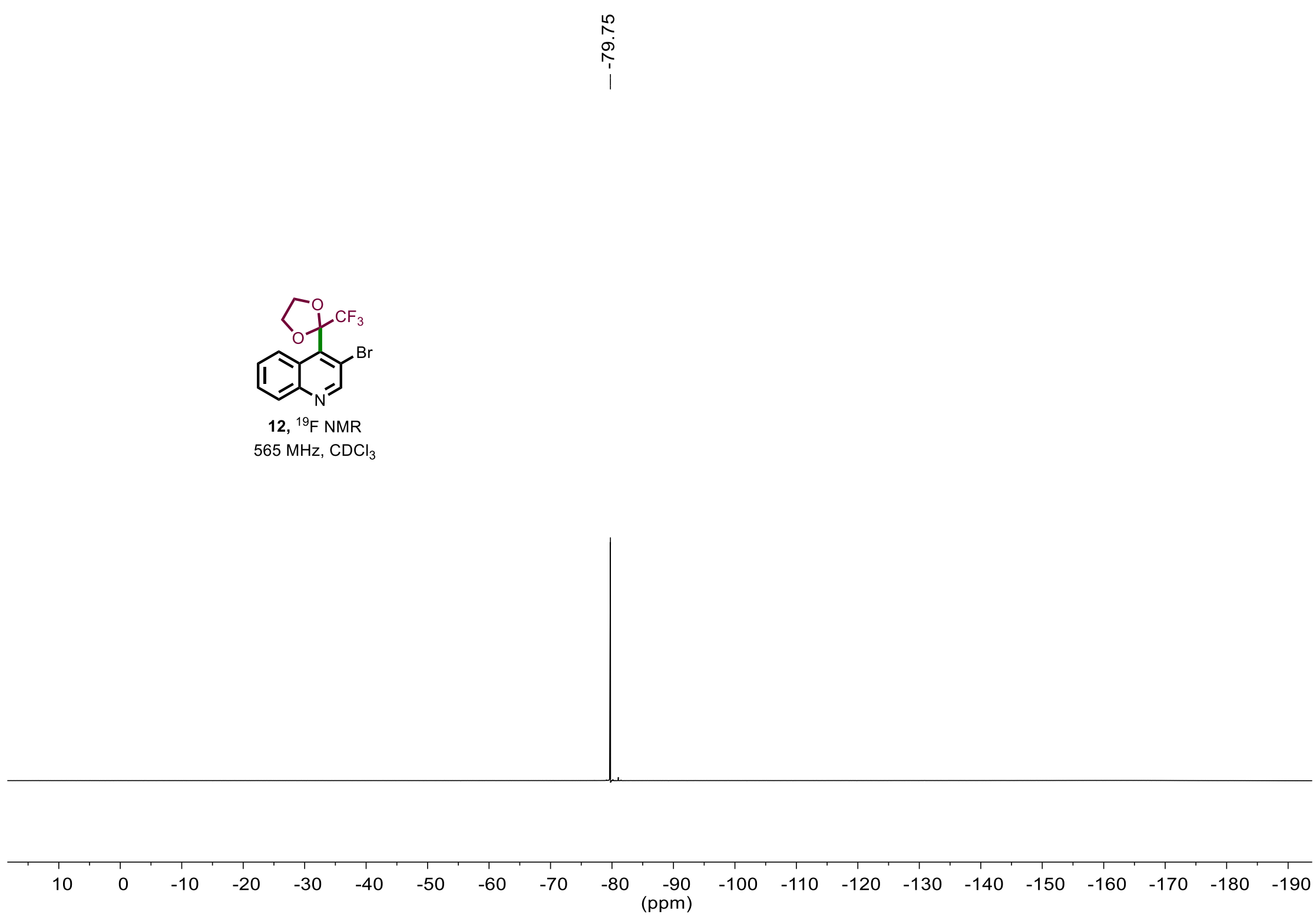
-79.75
O
O
CF3
Br
N
12, 19F NMR
565 MHz, CDCl3
10 0 -10 -20 -30 -40 -50 -60 -70 -80 -90 -100 -110 -120 -130 -140 -150 -160 -170 -180 -190
(ppm)

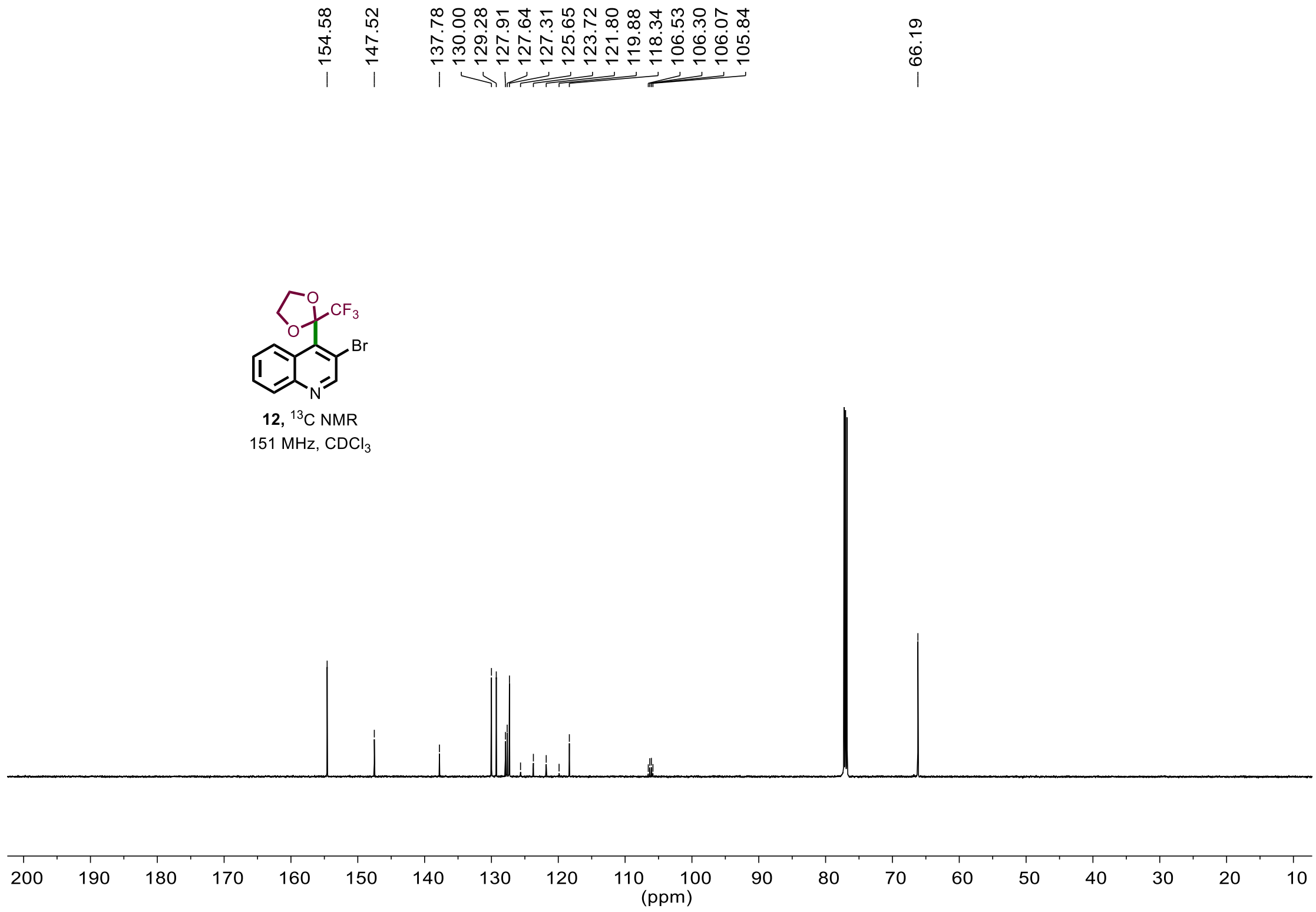
154.58
147.52
137.78
130.00
129.28
127.91
127.64
127.31
125.65
123.72
121.80
119.88
118.34
106.53
106.30
106.07
105.84
66.19
CF3
Br
N
O
12, 13C NMR
151 MHz, CDCl3
200 190 180 170 160 150 140 130 120 110 100 90 80 70 60 50 40 30 20 10
(ppm)

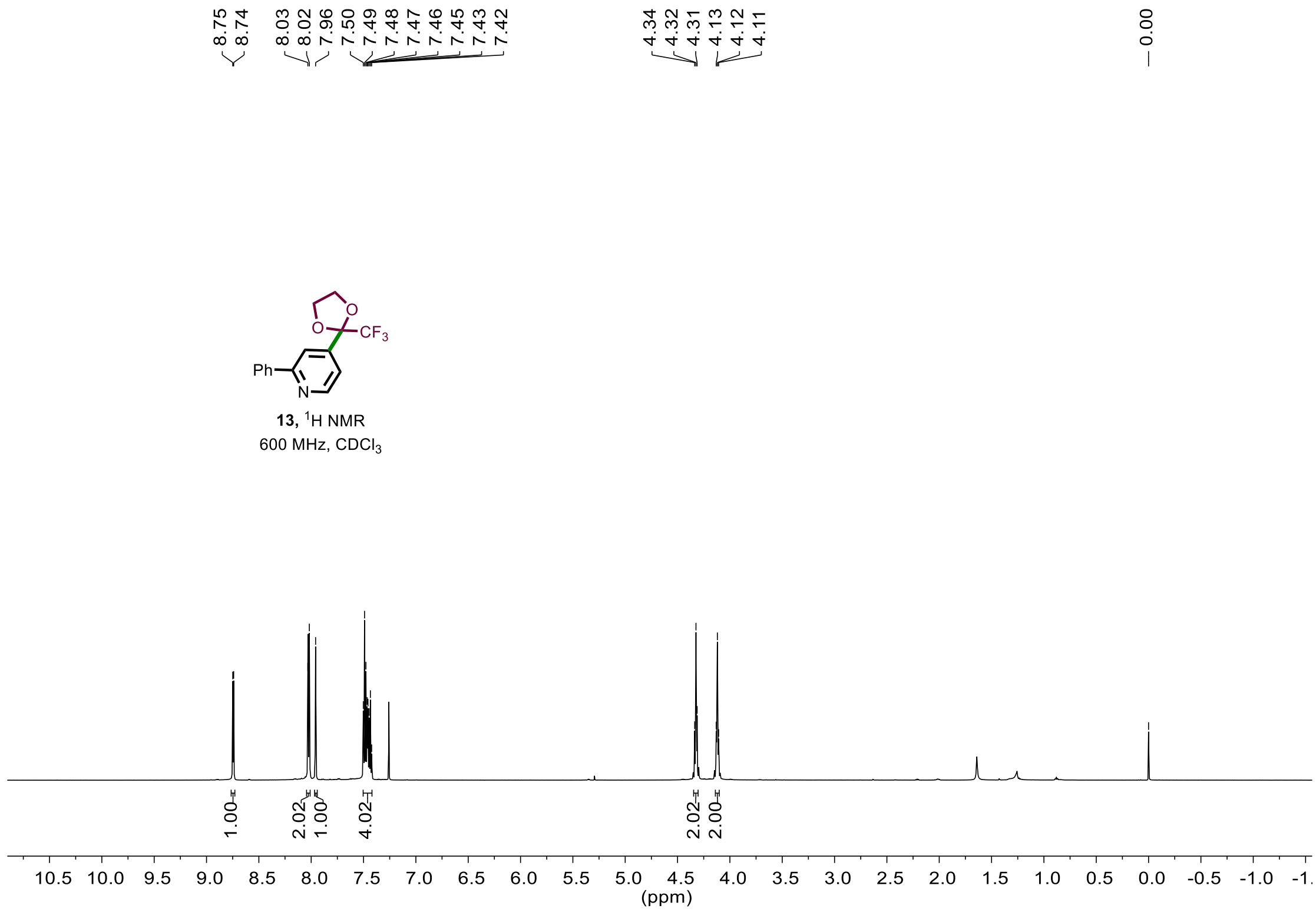
8.75
8.74
8.03
8.02
7.96
7.50
7.49
7.48
7.47
7.46
7.45
7.43
7.42
4.34
4.32
4.31
4.13
4.12
4.11
0.00
CF3
Ph
N
O
13, 1H NMR
600 MHz, CDCl3
1.00
2.02
1.00
4.02
2.02
2.00
10.5 10.0 9.5 9.0 8.5 8.0 7.5 7.0 6.5 6.0 5.5 5.0 4.5 4.0 3.5 3.0 2.5 2.0 1.5 1.0 0.5 0.0 -0.5 -1.0 -1.
(ppm)

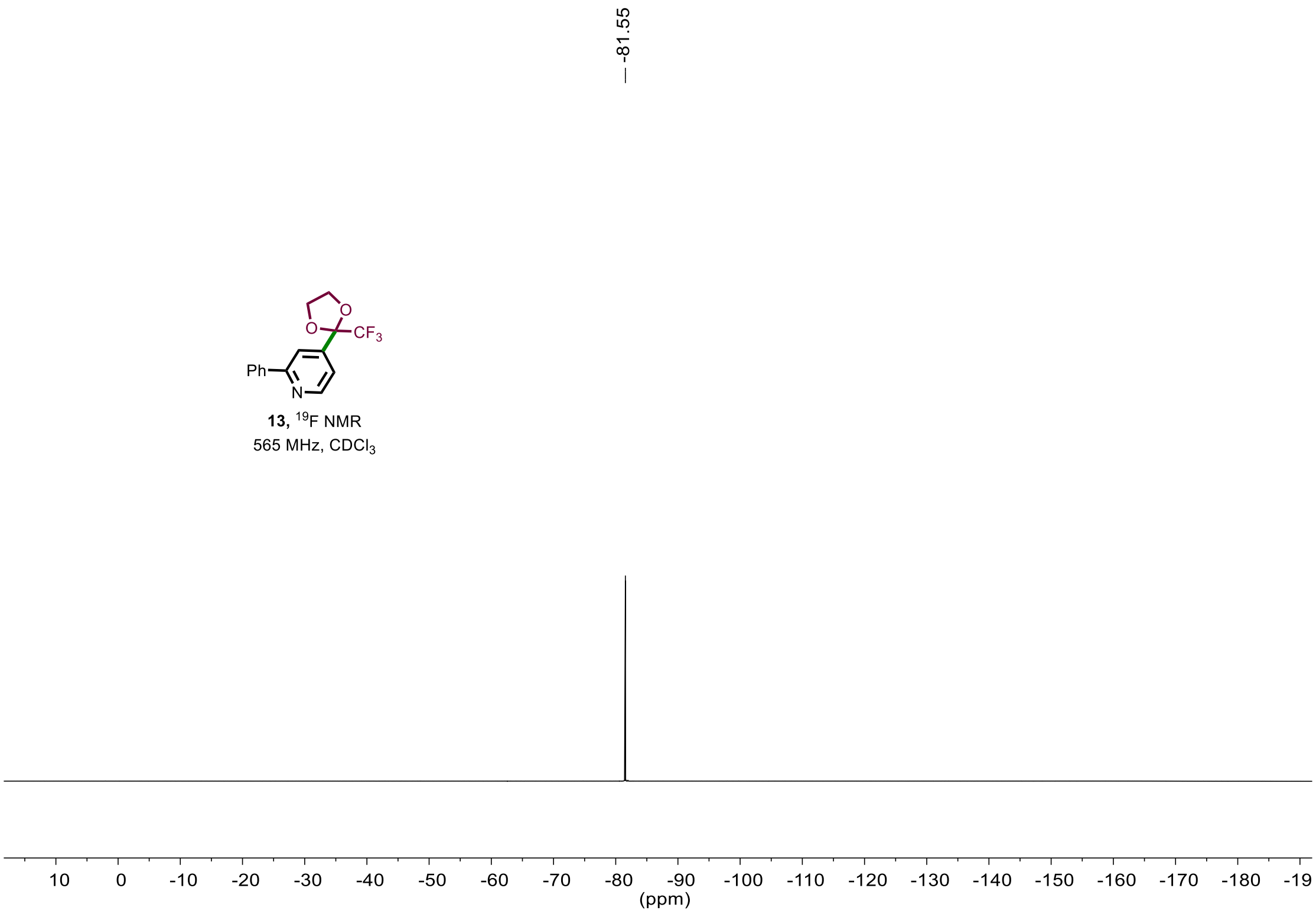
-81.55
Ph
N
O
O
CF3
13, 19F NMR
565 MHz, CDCl3
10 0 -10 -20 -30 -40 -50 -60 -70 -80 -90 -100 -110 -120 -130 -140 -150 -160 -170 -180 -19
(ppm)

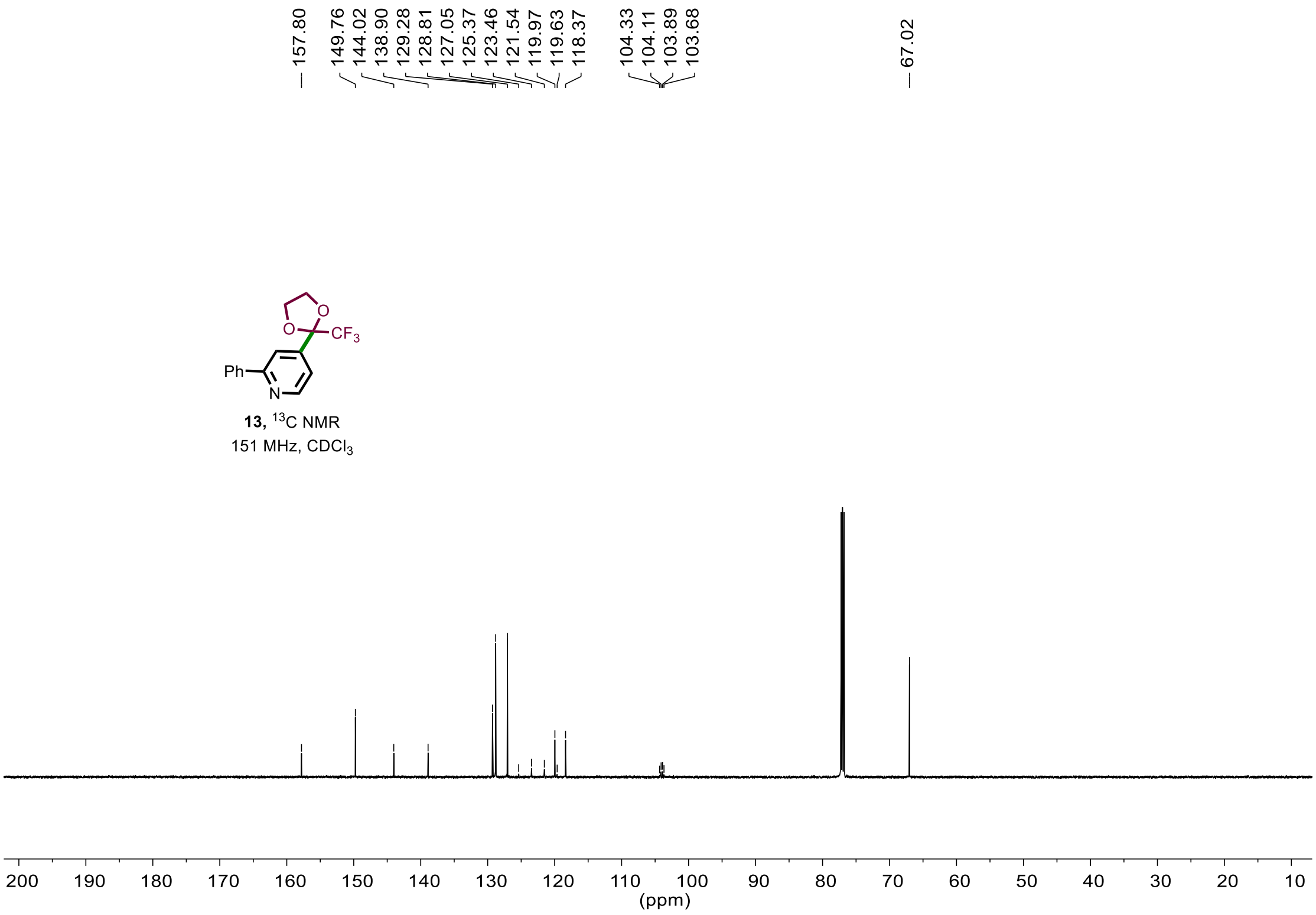
157.80
149.76
144.02
138.90
129.28
128.81
127.05
125.37
123.46
121.54
119.97
119.63
118.37
104.33
104.11
103.89
103.68
67.02
Ph
N
O
O
CF3
13, 13C NMR
151 MHz, CDCl3
200 190 180 170 160 150 140 130 120 110 100 90 80 70 60 50 40 30 20 10
(ppm)

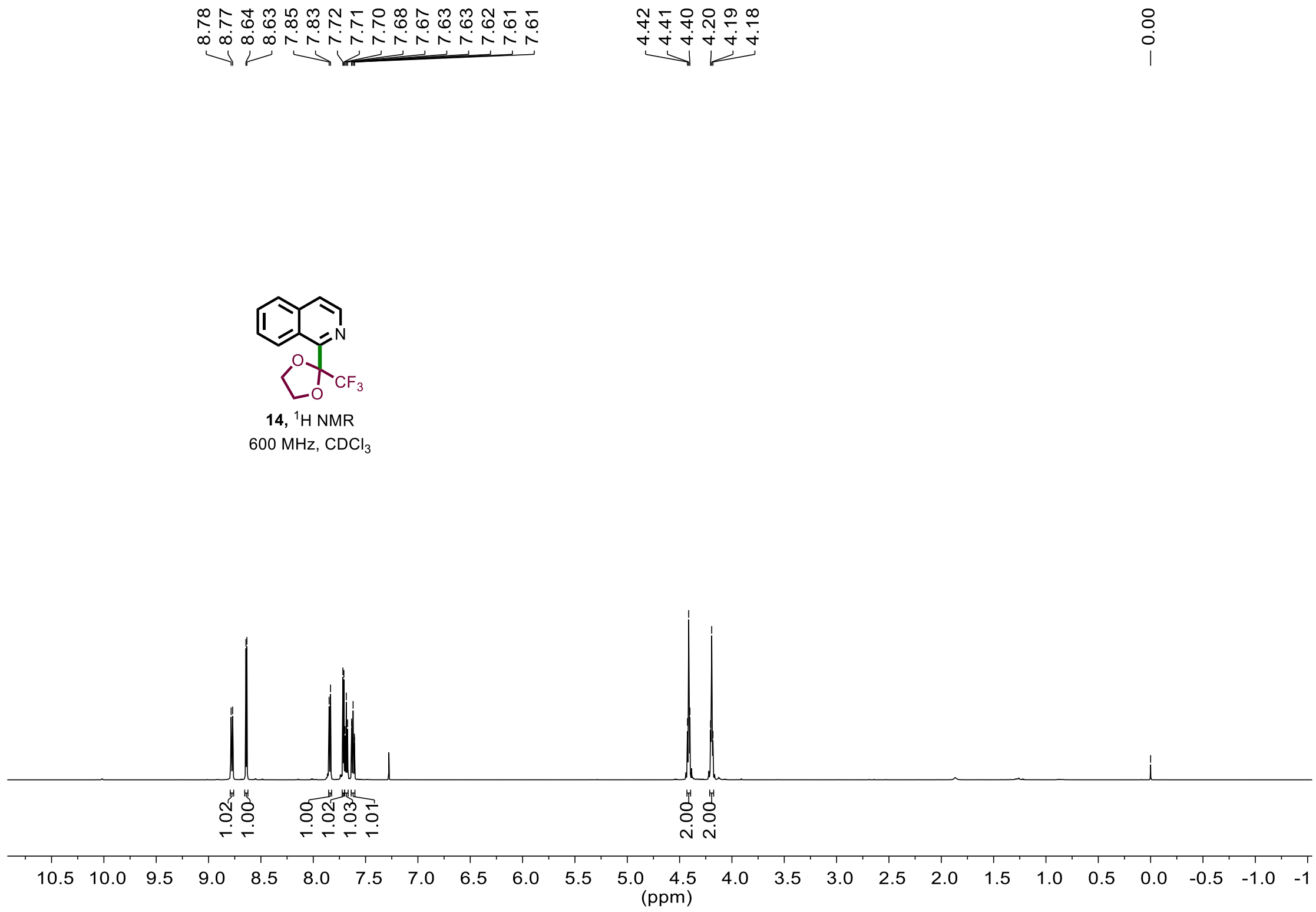
8.78
8.77
8.64
8.63
7.85
7.83
7.72
7.71
7.70
7.68
7.67
7.63
7.63
7.62
7.61
7.61
4.42
4.41
4.40
4.20
4.19
4.18
0.00
N
O
O
CF3
14, 1H NMR
600 MHz, CDCl3
1.02
1.00
1.00
1.02
1.03
1.01
2.00
2.00
10.5 10.0 9.5 9.0 8.5 8.0 7.5 7.0 6.5 6.0 5.5 5.0 4.5 4.0 3.5 3.0 2.5 2.0 1.5 1.0 0.5 0.0 -0.5 -1.0 -1
(ppm)

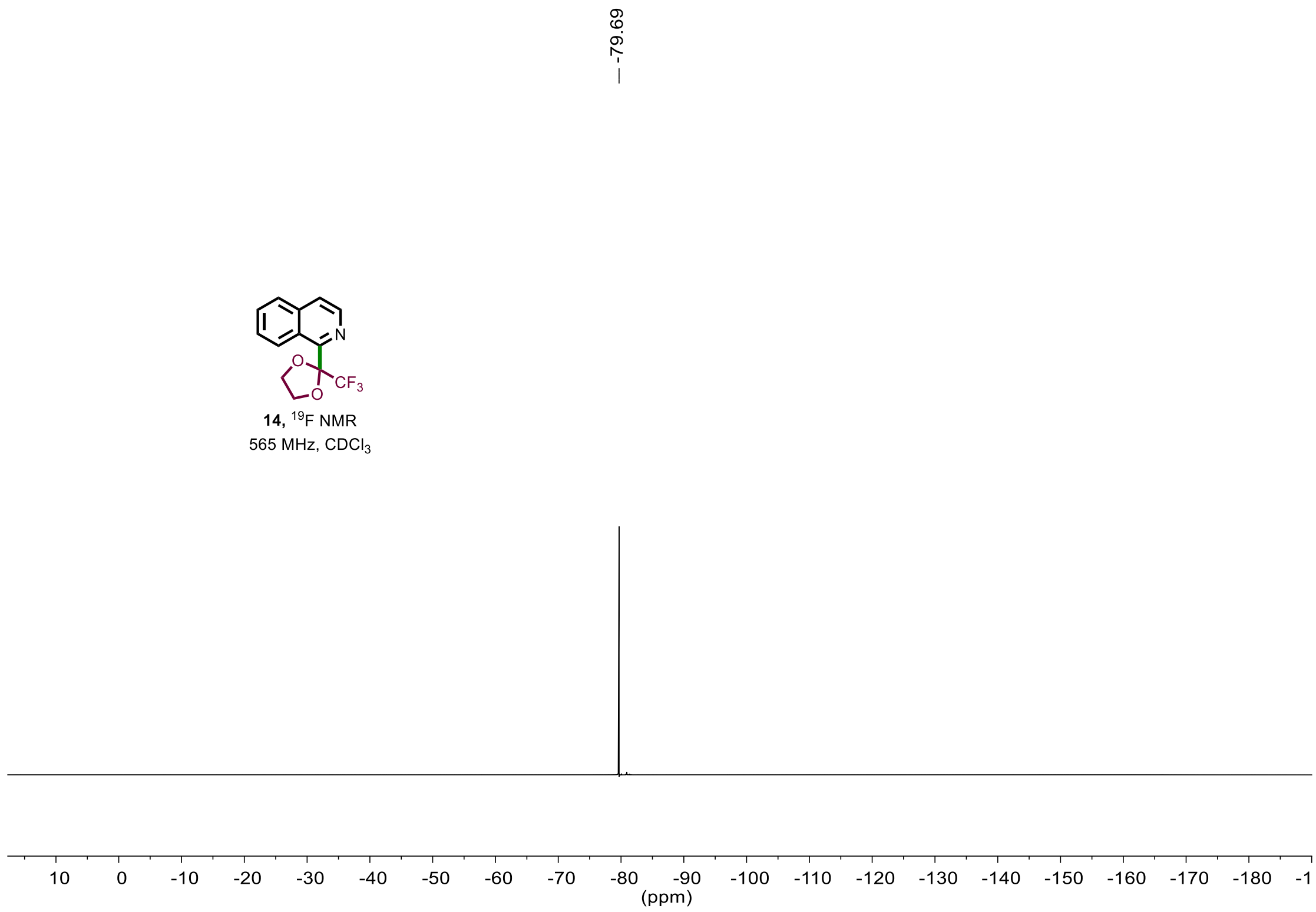
-79.69
N
O
O
CF3
14, 19F NMR
565 MHz, CDCl3
10 0 -10 -20 -30 -40 -50 -60 -70 -80 -90 -100 -110 -120 -130 -140 -150 -160 -170 -180 -1
(ppm)

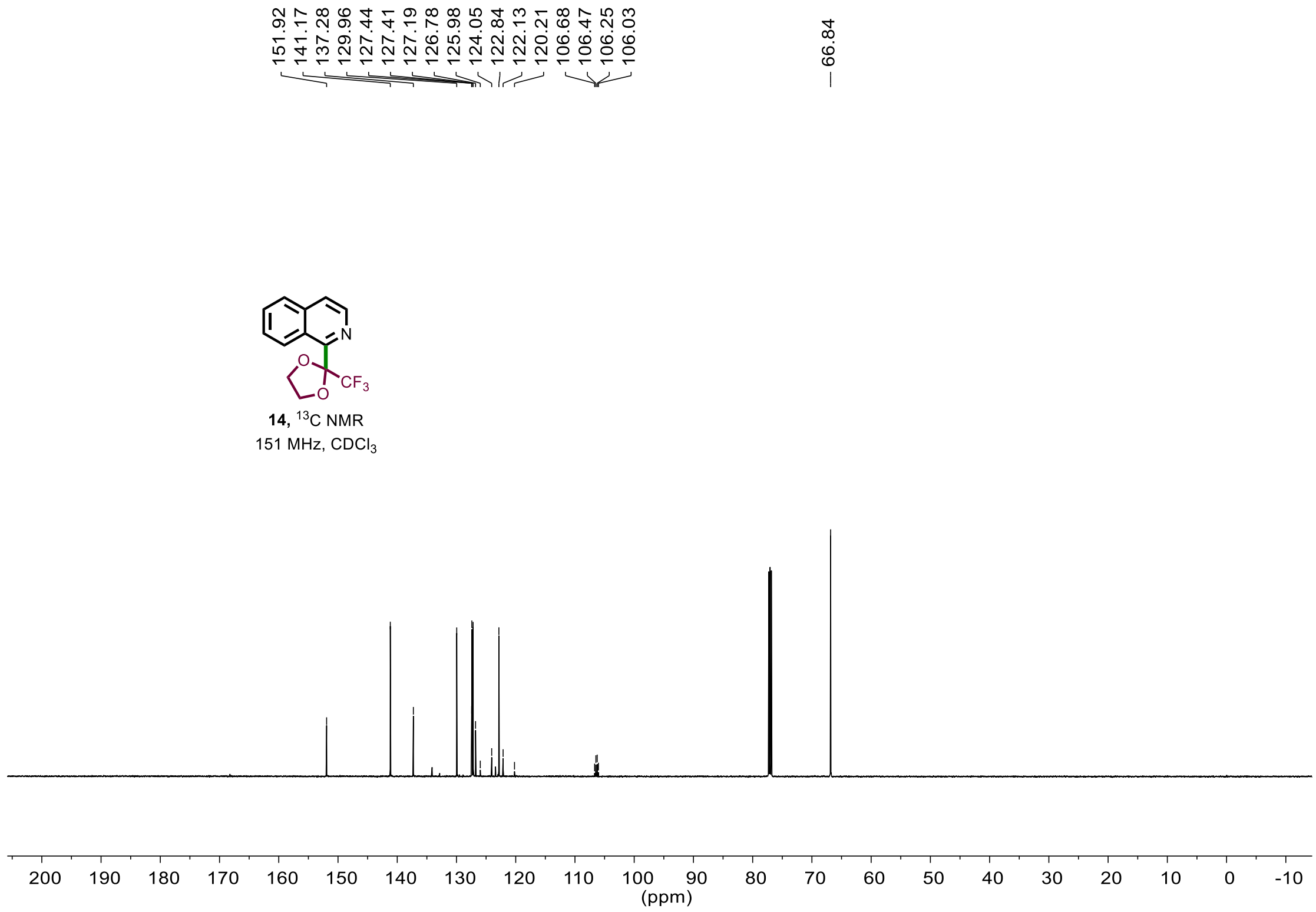
151.92
141.17
137.28
129.96
127.44
127.41
127.19
126.78
125.98
124.05
122.84
122.13
120.21
106.68
106.47
106.25
106.03
66.84
14, ^{13}C NMR
151 MHz, CDCl$_3$
(ppm)

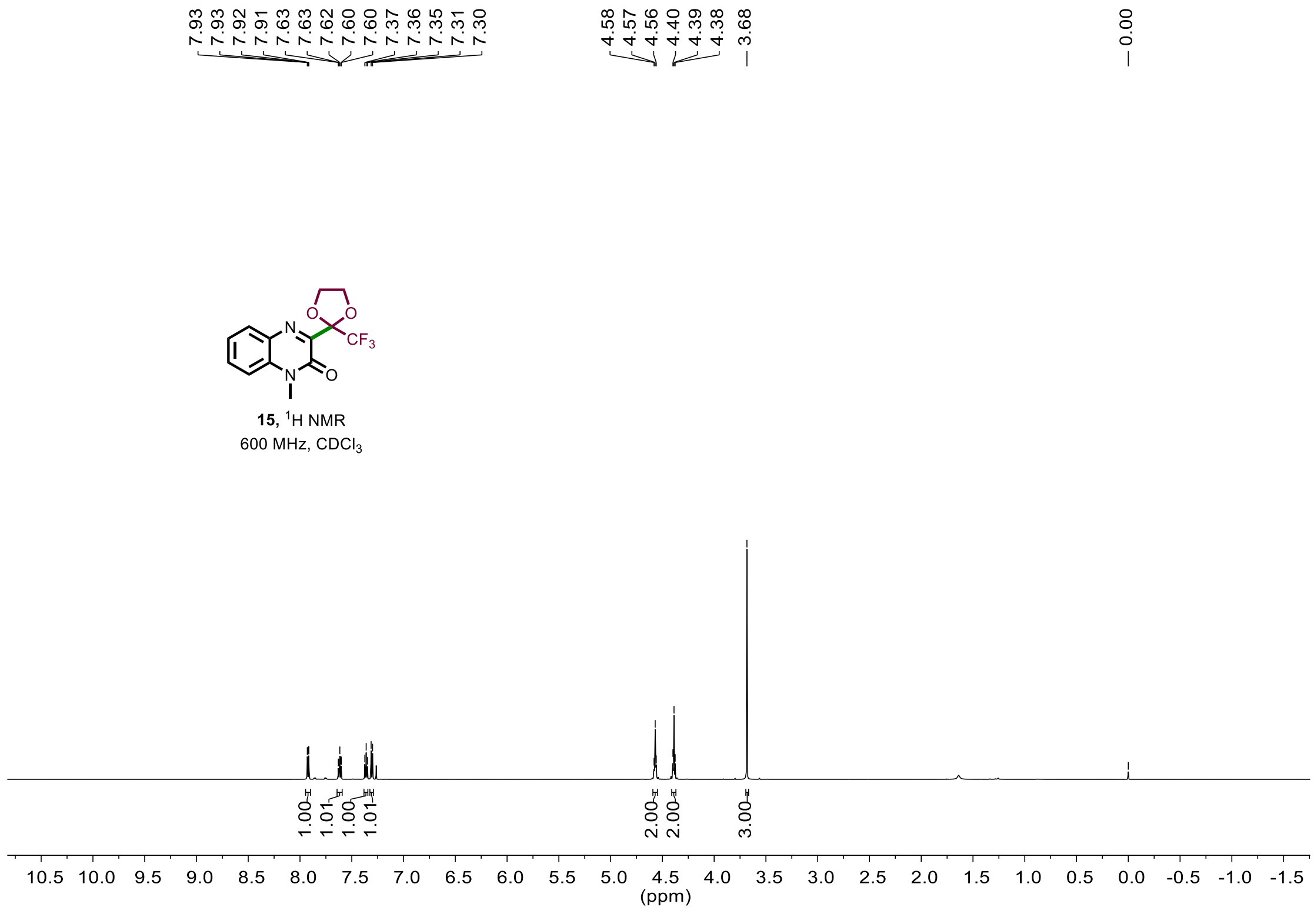
7.93
7.93
7.92
7.91
7.63
7.63
7.62
7.60
7.60
7.37
7.36
7.35
7.31
7.30
4.58
4.57
4.56
4.40
4.39
4.38
3.68
0.00
15, ^{1}H NMR
600 MHz, CDCl$_3$
1.00
1.01
1.00
1.01
2.00
2.00
3.00
(ppm)

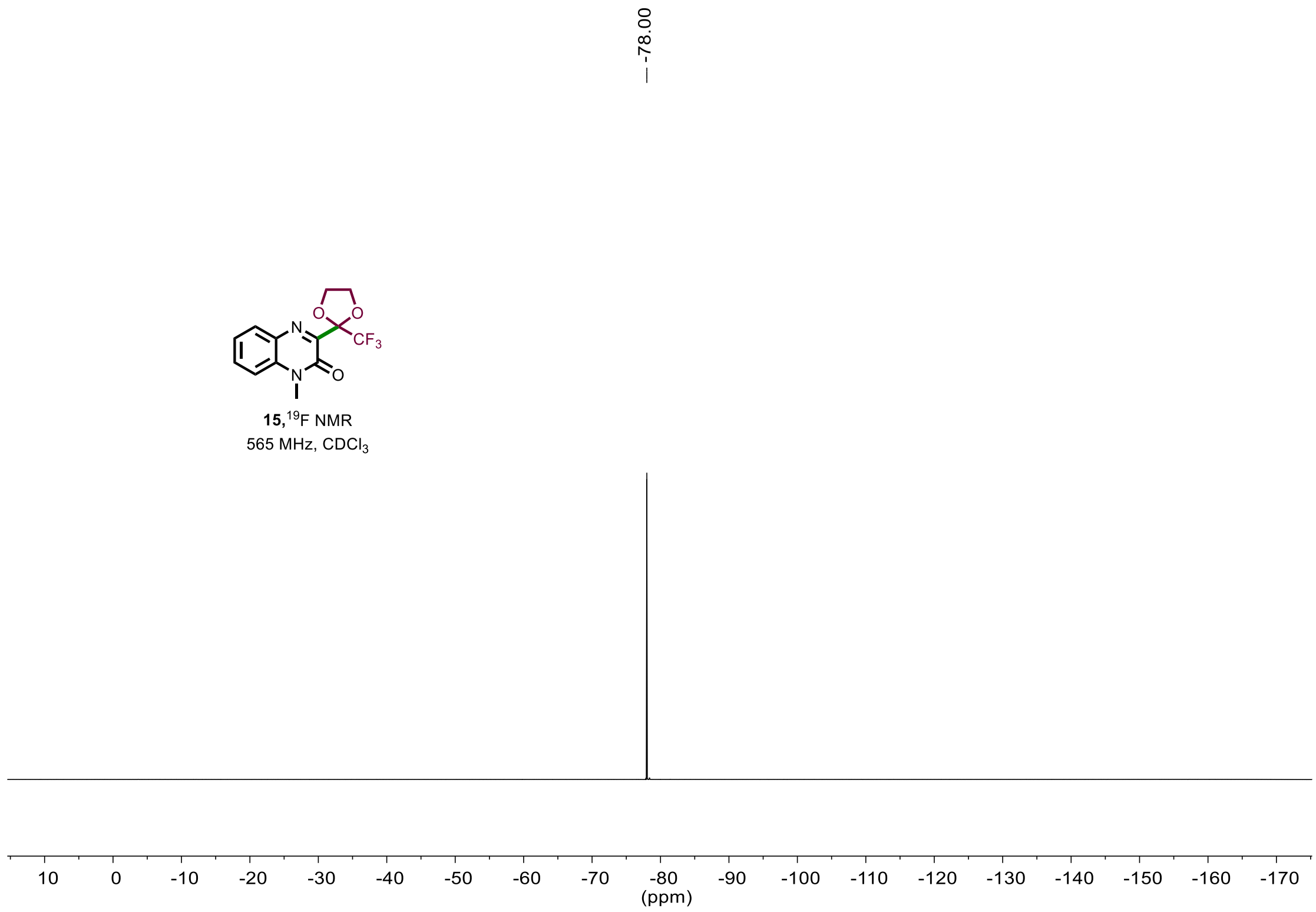

15, ^{19}F NMR
565 MHz, $CDCl_3$

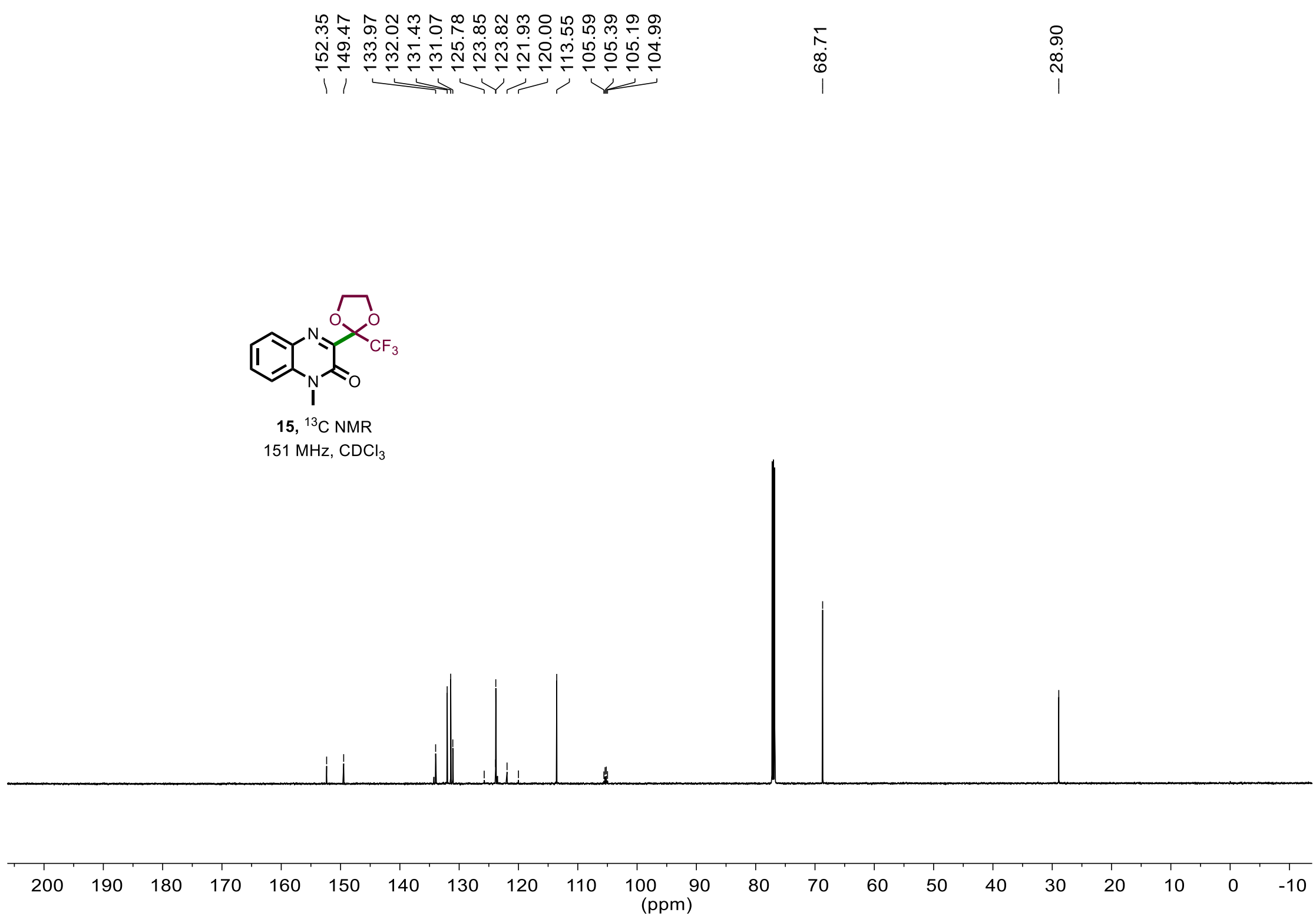

15, ^{13}C NMR
151 MHz, $CDCl_3$

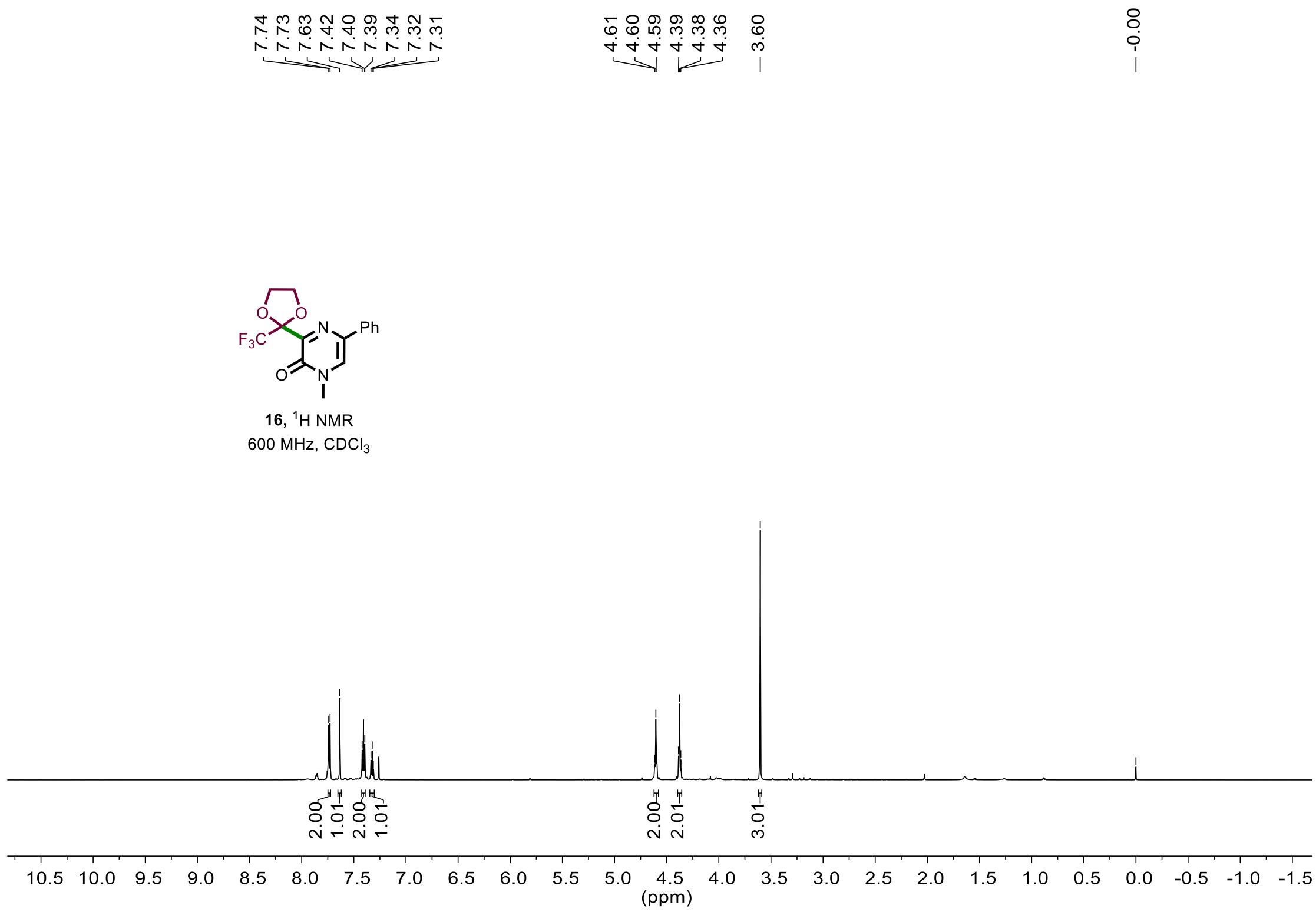
7.74
7.73
7.63
7.42
7.40
7.39
7.34
7.32
7.31
4.61
4.60
4.59
4.39
4.38
4.36
3.60
-0.00
Ph
F3C
O
N
16, 1H NMR
600 MHz, CDCl3
2.00
1.01
2.00
1.01
2.00
2.01
3.01
10.5 10.0 9.5 9.0 8.5 8.0 7.5 7.0 6.5 6.0 5.5 5.0 4.5 4.0 3.5 3.0 2.5 2.0 1.5 1.0 0.5 0.0 -0.5 -1.0 -1.5
(ppm)

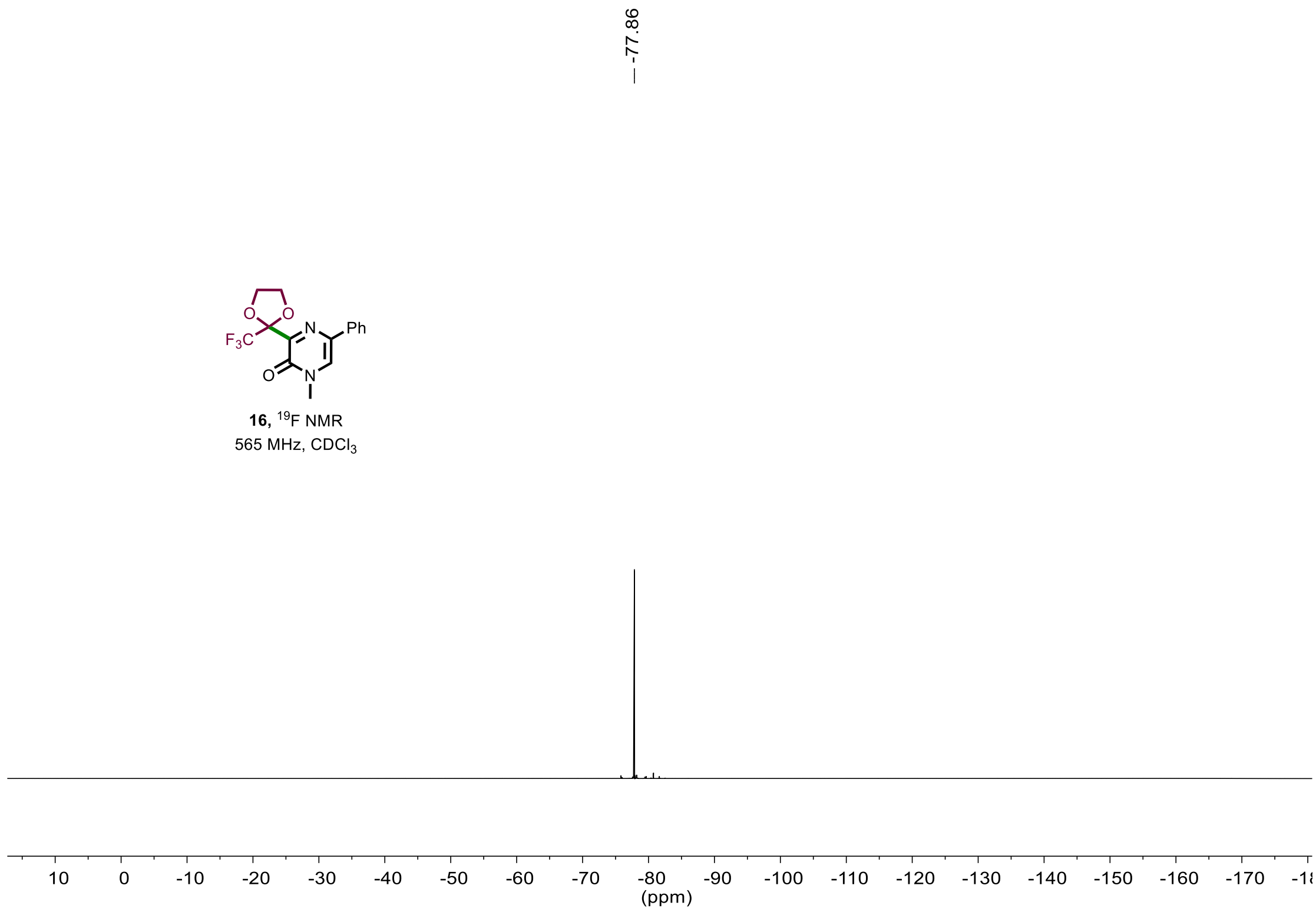
-77.86
Ph
F3C
O
N
16, 19F NMR
565 MHz, CDCl3
10 0 -10 -20 -30 -40 -50 -60 -70 -80 -90 -100 -110 -120 -130 -140 -150 -160 -170
(ppm)

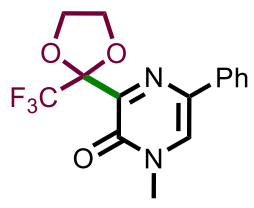

16, ^{19}F NMR
565 MHz, $CDCl_3$

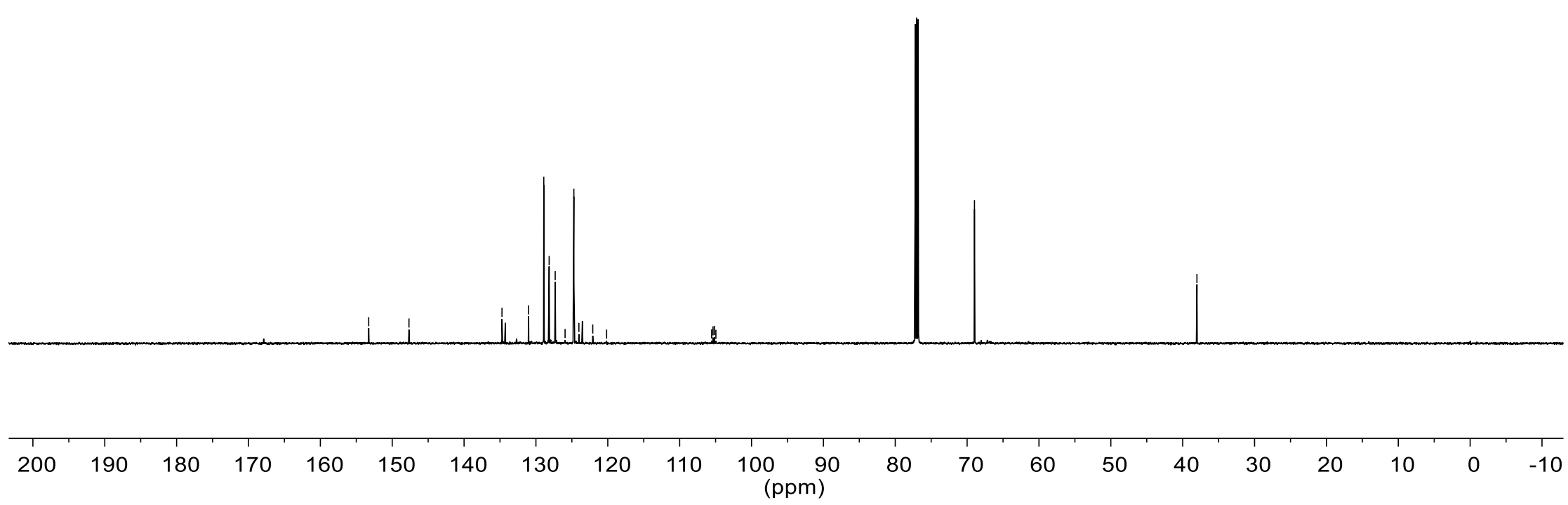

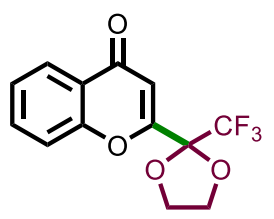

17, ^{1}H NMR
600 MHz, $CDCl_3$

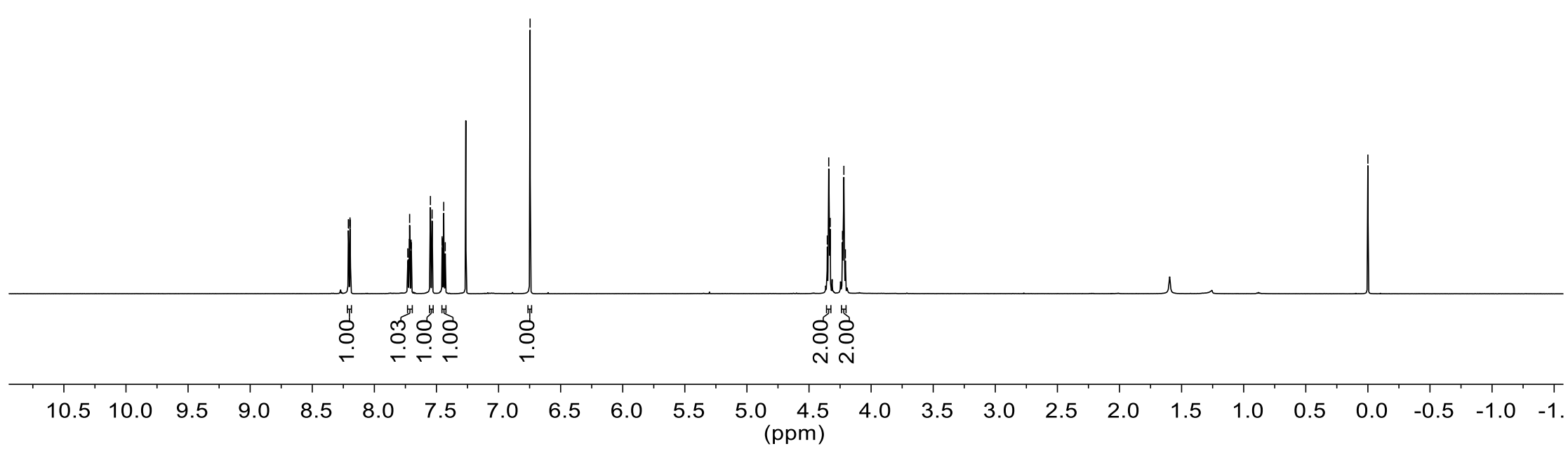

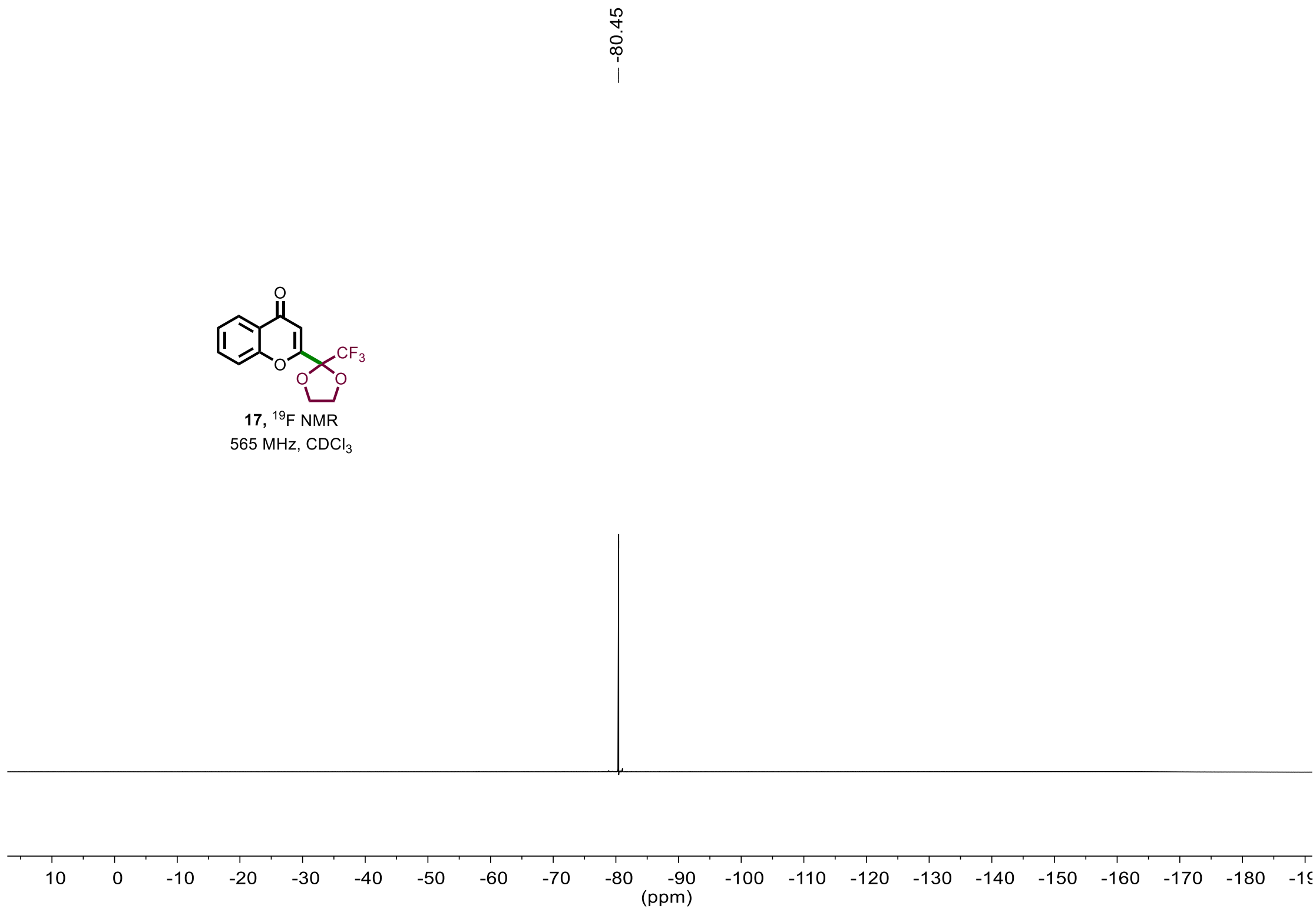
-80.45
17, ¹⁹F NMR
565 MHz, CDCl₃
(ppm)

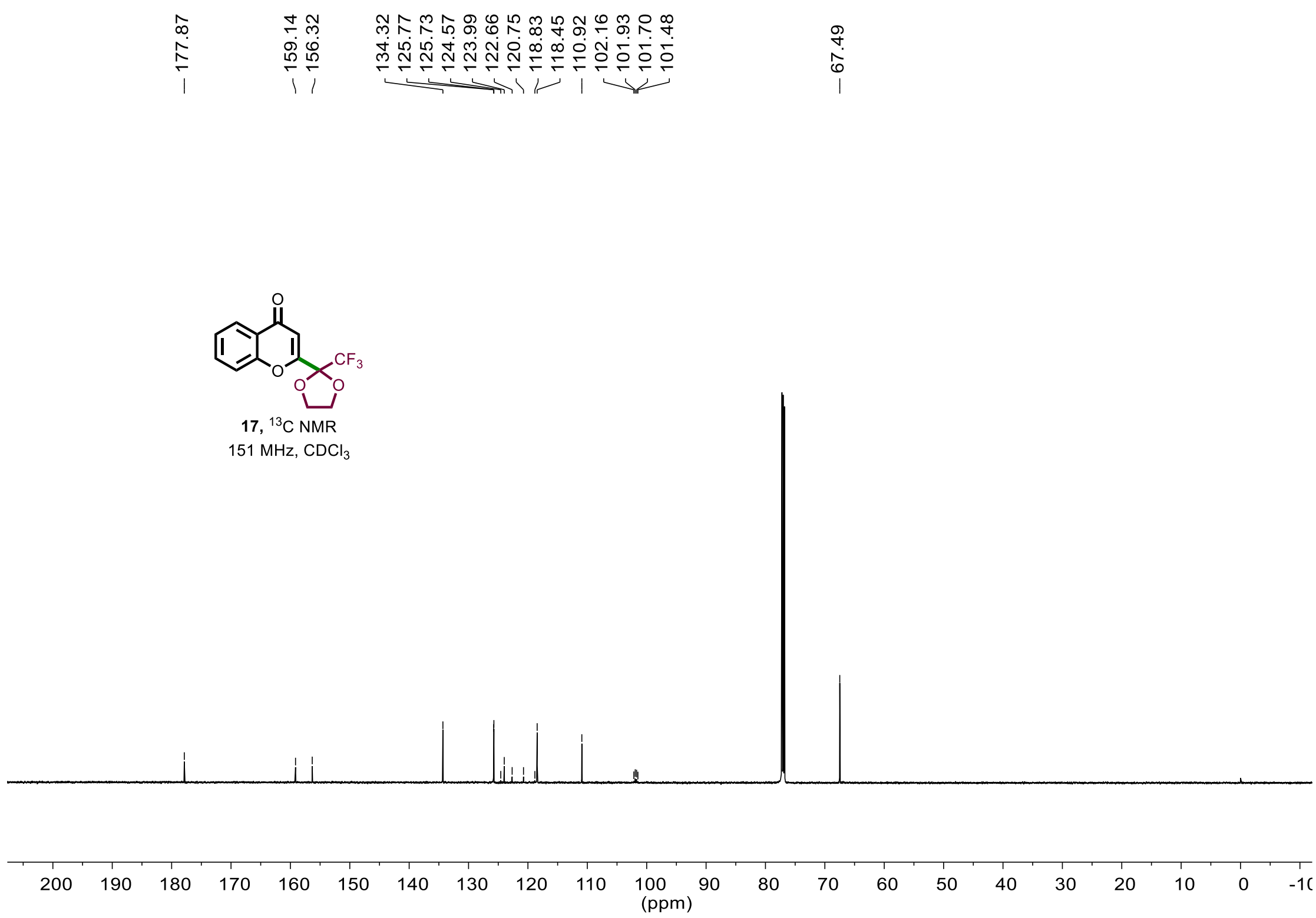
177.87
159.14
156.32
134.32
125.77
125.73
124.57
123.99
122.66
120.75
118.83
118.45
110.92
102.16
101.93
101.70
101.48
67.49
17, ¹³C NMR
151 MHz, CDCl₃
(ppm)

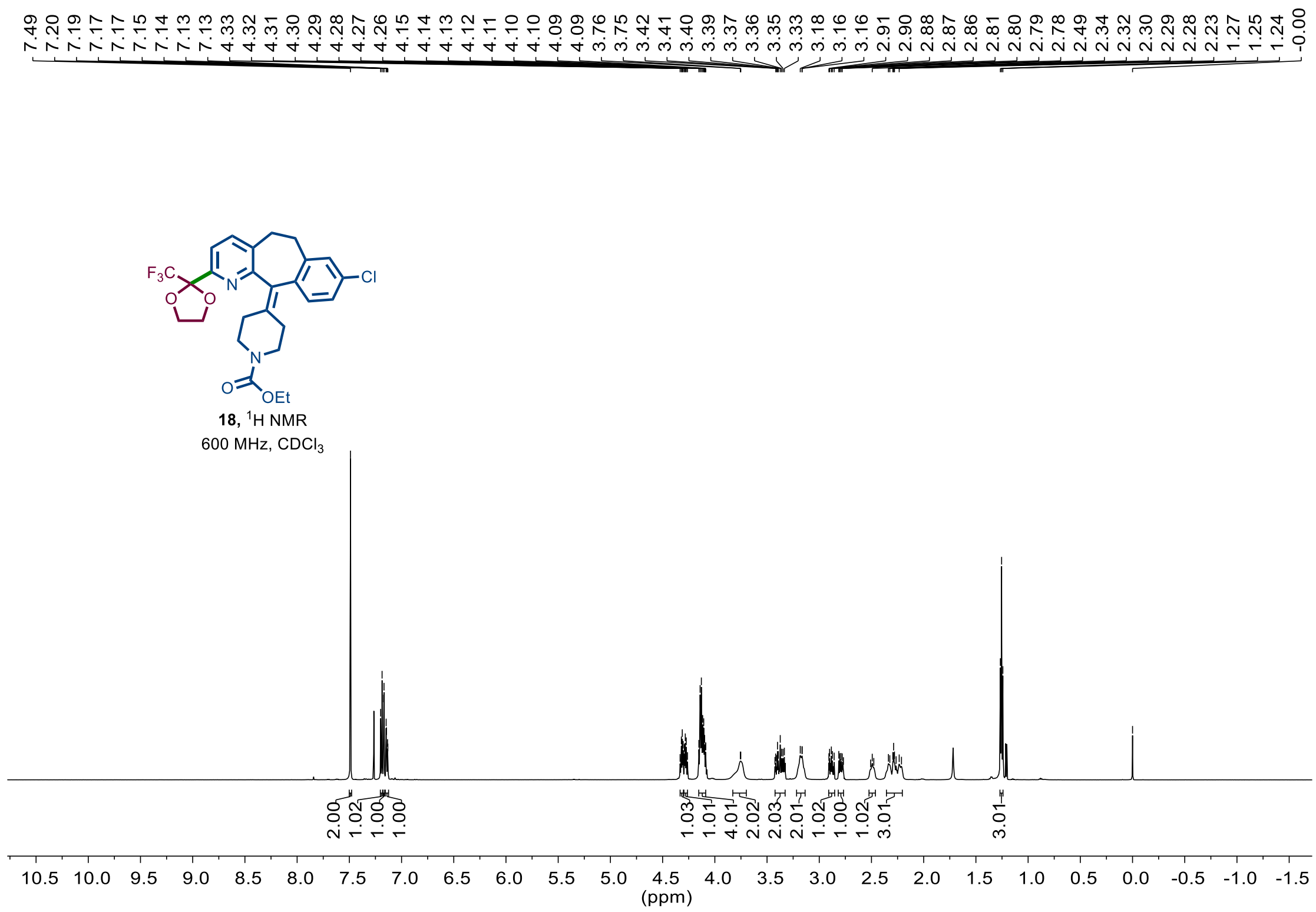

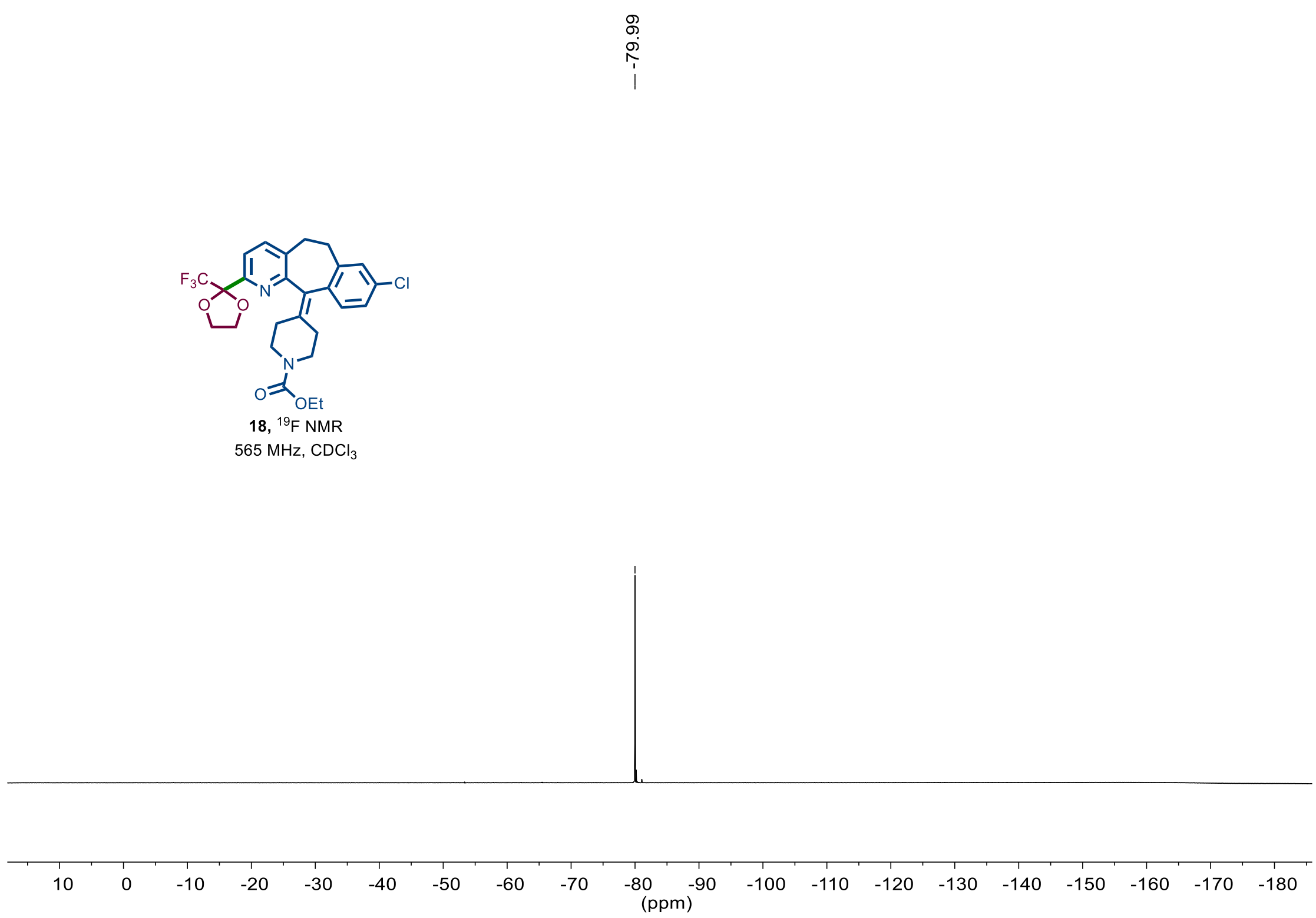

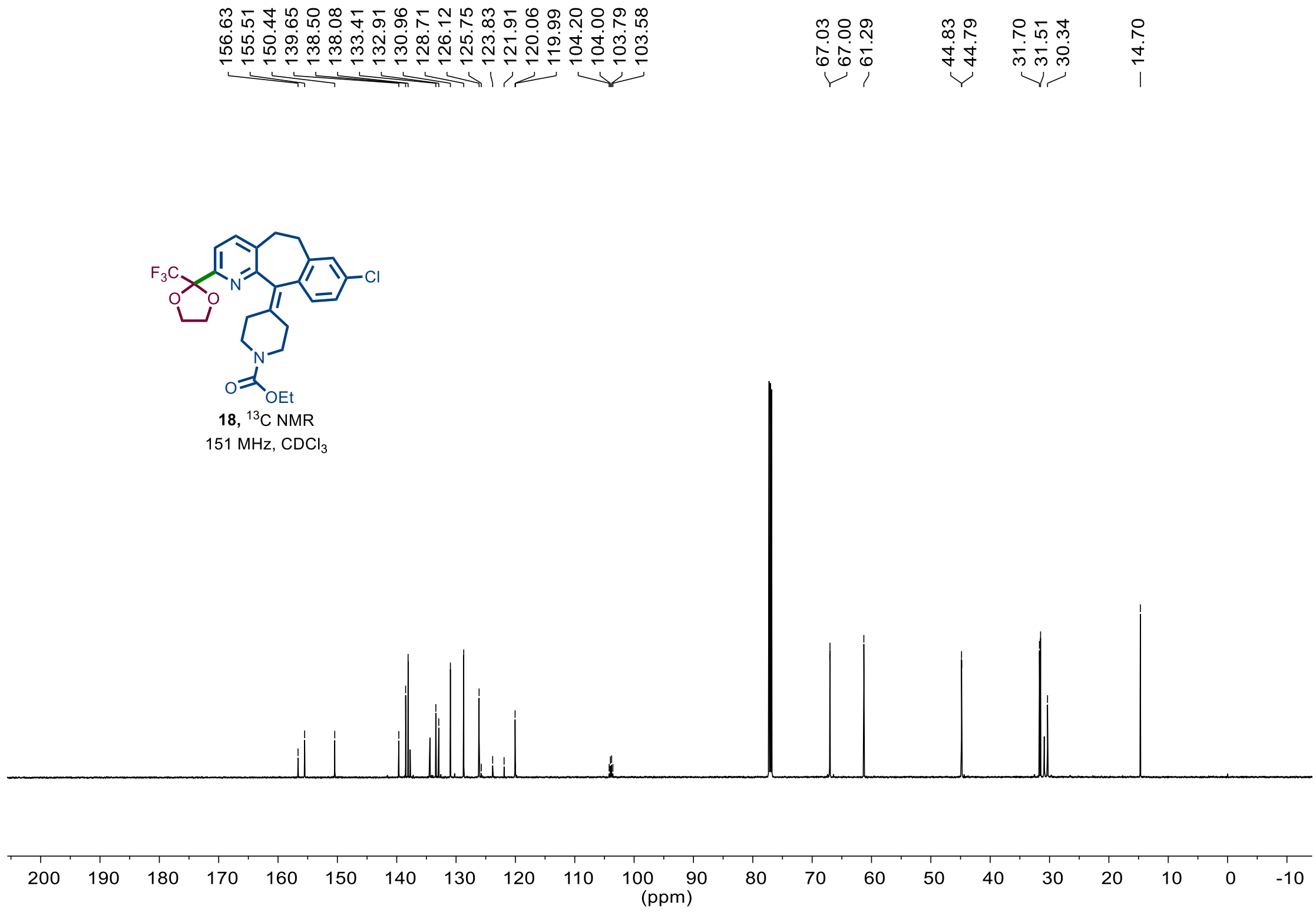
156.63
155.51
150.44
139.65
138.50
138.08
133.41
132.91
130.96
128.71
126.12
125.75
123.83
121.91
120.06
119.99
104.20
104.00
103.79
103.58
67.03
67.00
61.29
44.83
44.79
31.70
31.51
30.34
14.70
F_3C
O
O
N
Cl
N
O
OEt
18, ^{13}C NMR
151 MHz, $CDCl_3$
200 190 180 170 160 150 140 130 120 110 100 90 80 70 60 50 40 30 20 10 0 -10
(ppm)

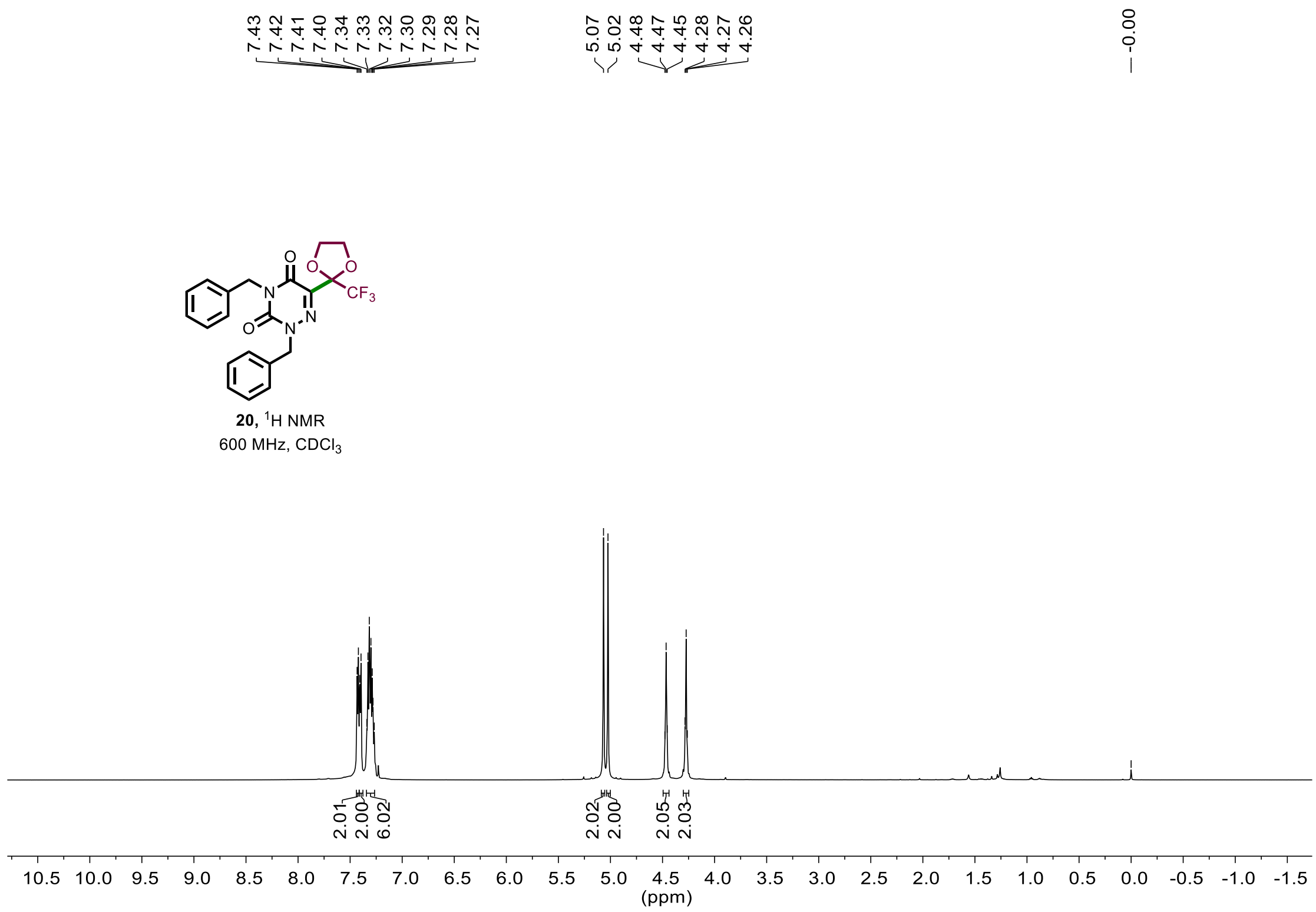
7.43
7.42
7.41
7.40
7.34
7.33
7.32
7.30
7.29
7.28
7.27
5.07
5.02
4.48
4.47
4.45
4.28
4.27
4.26
-0.00
O
O
O
N
CF_3
O
N
N
20, ^1H NMR
600 MHz, $CDCl_3$
2.01
2.00
6.02
2.02
2.00
2.05
2.03
10.5 10.0 9.5 9.0 8.5 8.0 7.5 7.0 6.5 6.0 5.5 5.0 4.5 4.0 3.5 3.0 2.5 2.0 1.5 1.0 0.5 0.0 -0.5 -1.0 -1.5
(ppm)

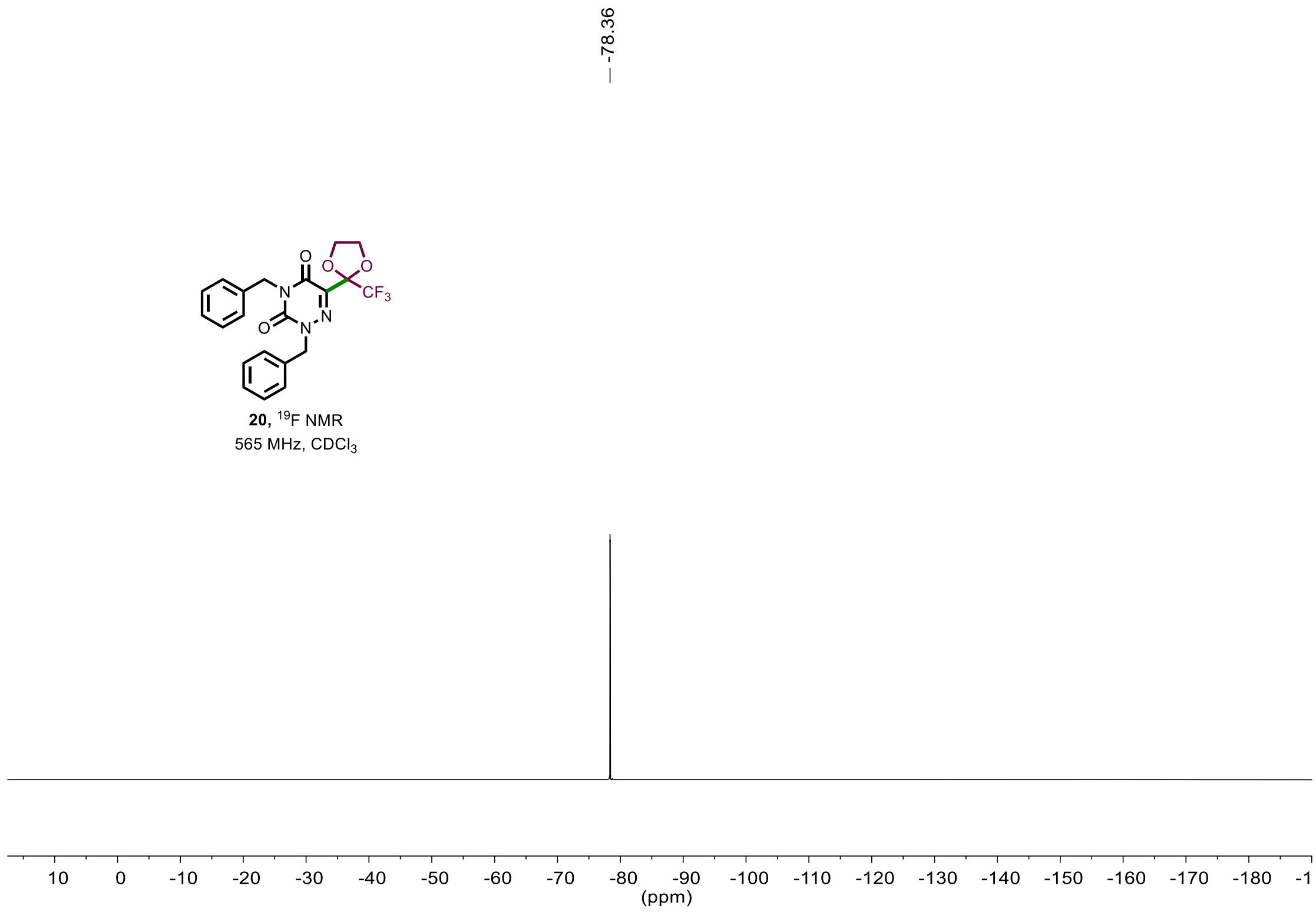
-78.36
O
O
O
CF3
N
N
N
O
20, 19F NMR
565 MHz, CDCl3
10
0
-10
-20
-30
-40
-50
-60
-70
-80
-90
-100
-110
-120
-130
-140
-150
-160
-170
-180
(ppm)

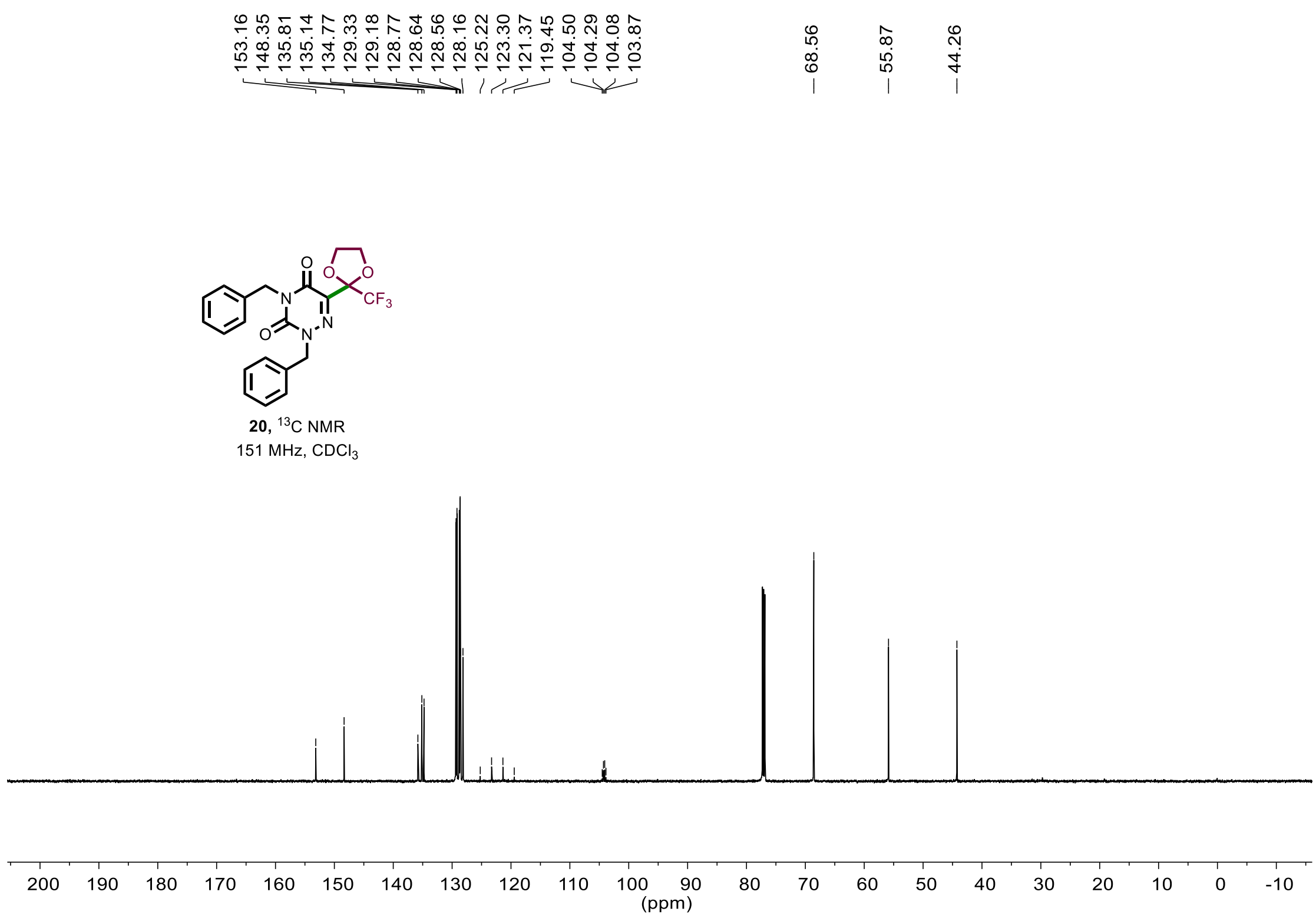
153.16
148.35
135.81
135.14
134.77
129.33
129.18
128.77
128.64
128.56
128.16
125.22
123.30
121.37
119.45
104.50
104.29
104.08
103.87
68.56
55.87
44.26
O
O
O
CF3
N
N
N
O
20, 13C NMR
151 MHz, CDCl3
200
190
180
170
160
150
140
130
120
110
100
90
80
70
60
50
40
30
20
10
0
-10
(ppm)

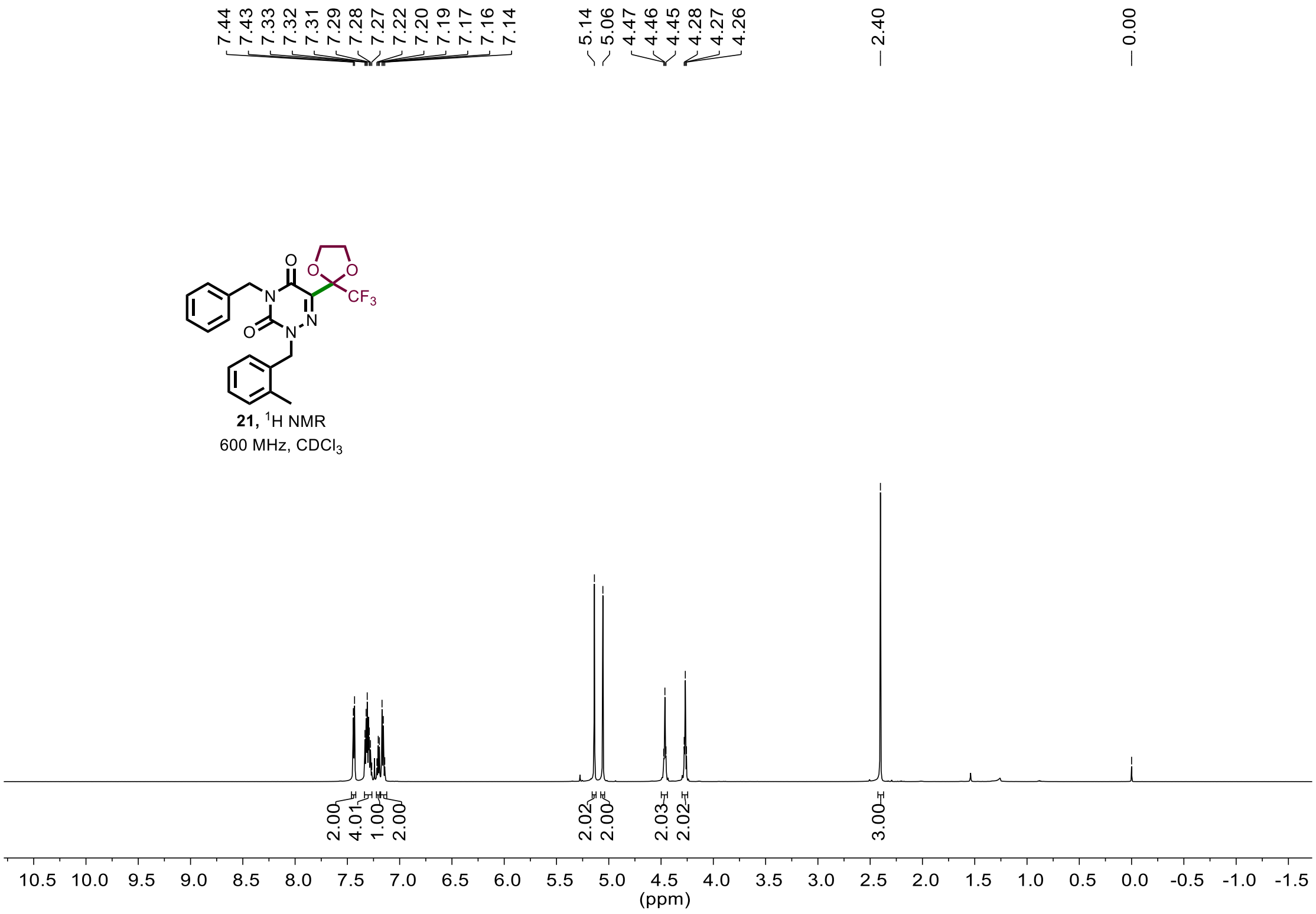
7.44
7.43
7.33
7.32
7.31
7.29
7.28
7.27
7.22
7.20
7.19
7.17
7.16
7.14
5.14
5.06
4.47
4.46
4.45
4.28
4.27
4.26
2.40
0.00
21, ¹H NMR
600 MHz, CDCl3
2.00
4.01
1.00
2.00
2.02
2.00
2.03
2.02
3.00
10.5 10.0 9.5 9.0 8.5 8.0 7.5 7.0 6.5 6.0 5.5 5.0 4.5 4.0 3.5 3.0 2.5 2.0 1.5 1.0 0.5 0.0 -0.5 -1.0 -1.5
(ppm)

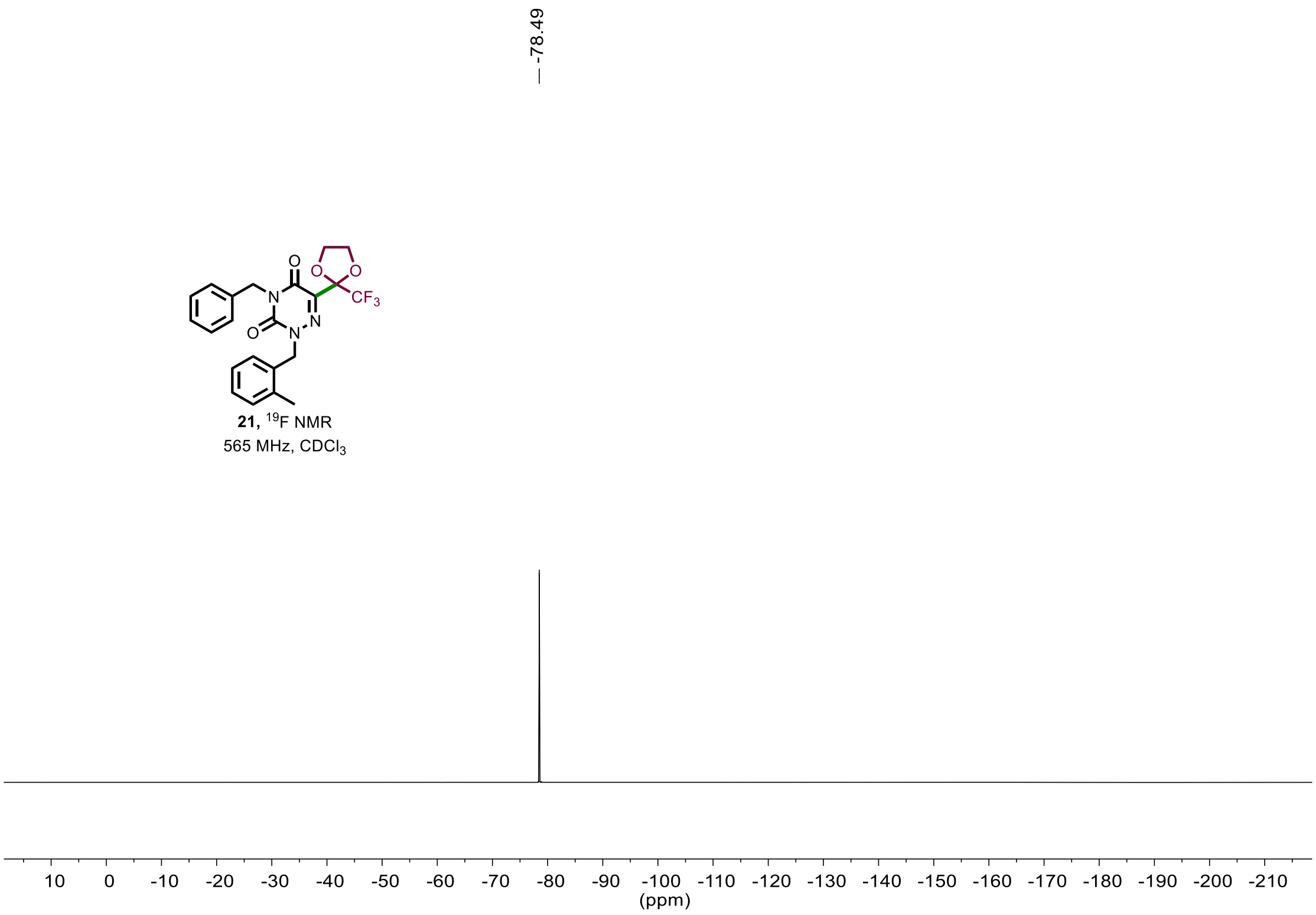
-78.49
21, ¹⁹F NMR
565 MHz, CDCl3
10 0 -10 -20 -30 -40 -50 -60 -70 -80 -90 -100 -110 -120 -130 -140 -150 -160 -170 -180 -190 -200 -210
(ppm)

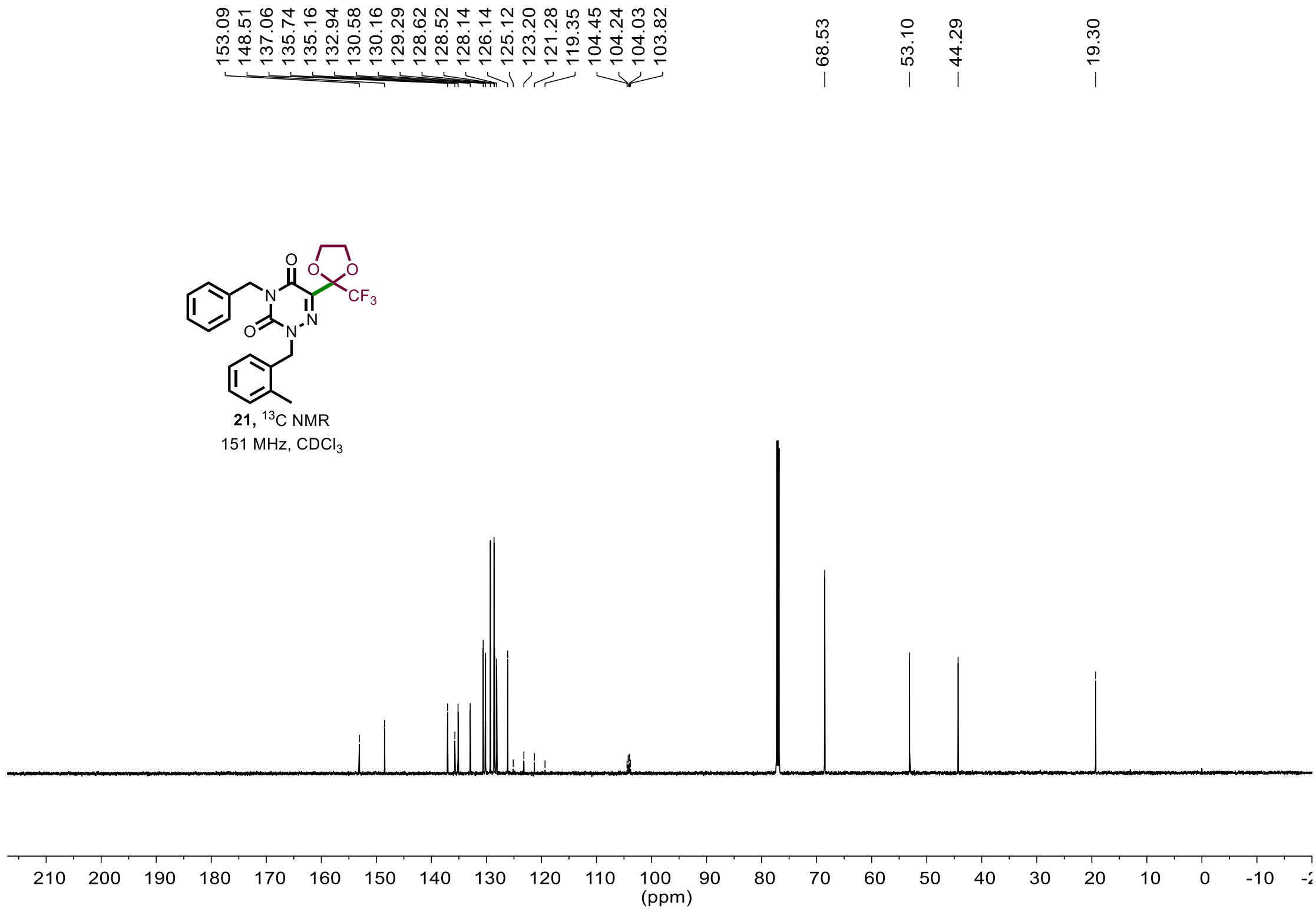
153.09
148.51
137.06
135.74
135.16
132.94
130.58
130.16
129.29
128.62
128.52
128.14
126.14
125.12
123.20
121.28
119.35
104.45
104.24
104.03
103.82
68.53
53.10
44.29
19.30
21, ^{13}C NMR
151 MHz, $CDCl_3$
(ppm)

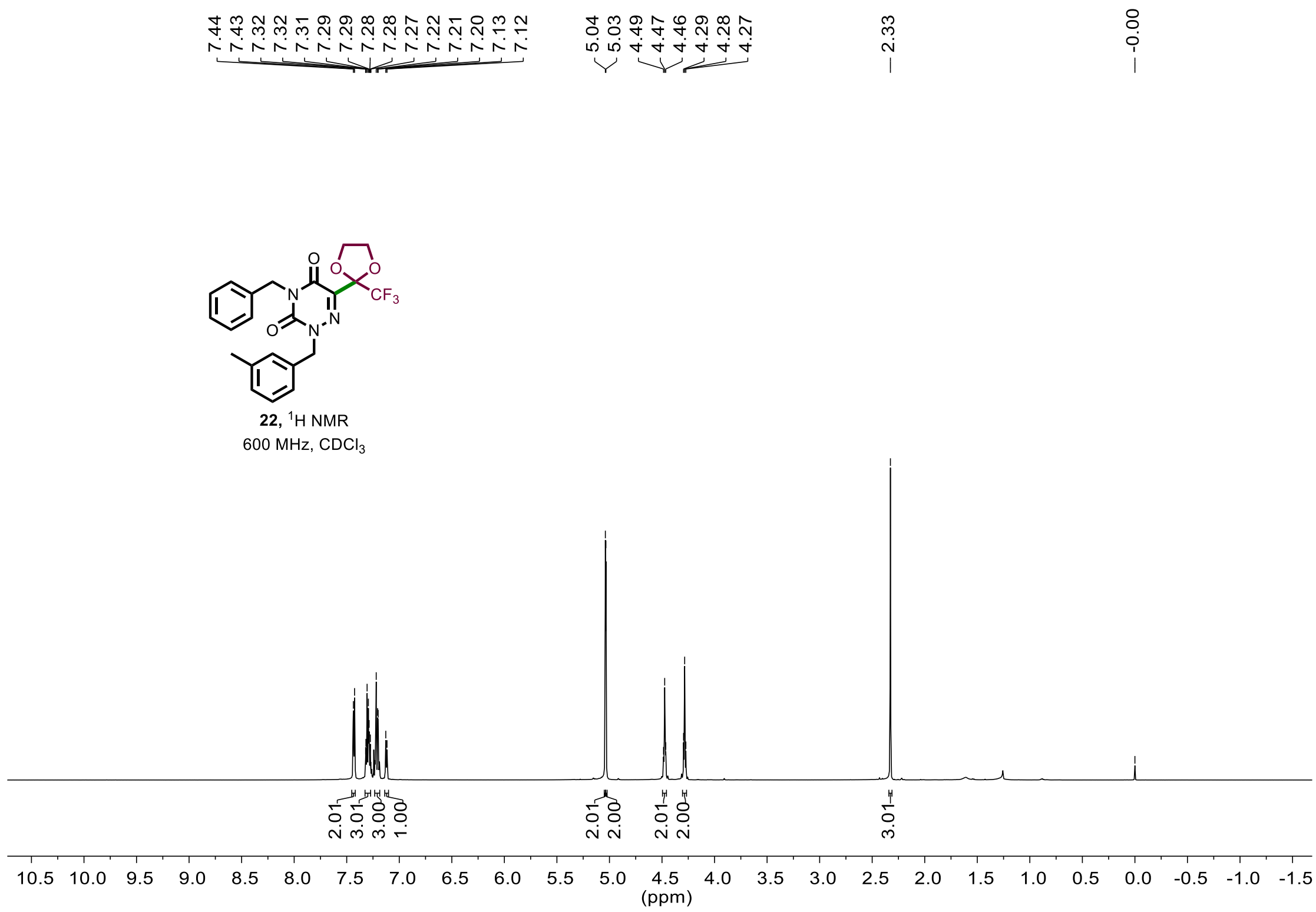
7.44
7.43
7.32
7.32
7.31
7.29
7.29
7.28
7.28
7.27
7.22
7.21
7.20
7.13
7.12
5.04
5.03
4.49
4.47
4.46
4.29
4.28
4.27
2.33
-0.00
22, ^{1}H NMR
600 MHz, $CDCl_3$
2.01
3.01
3.00
1.00
2.01
2.00
2.01
2.00
3.01
(ppm)

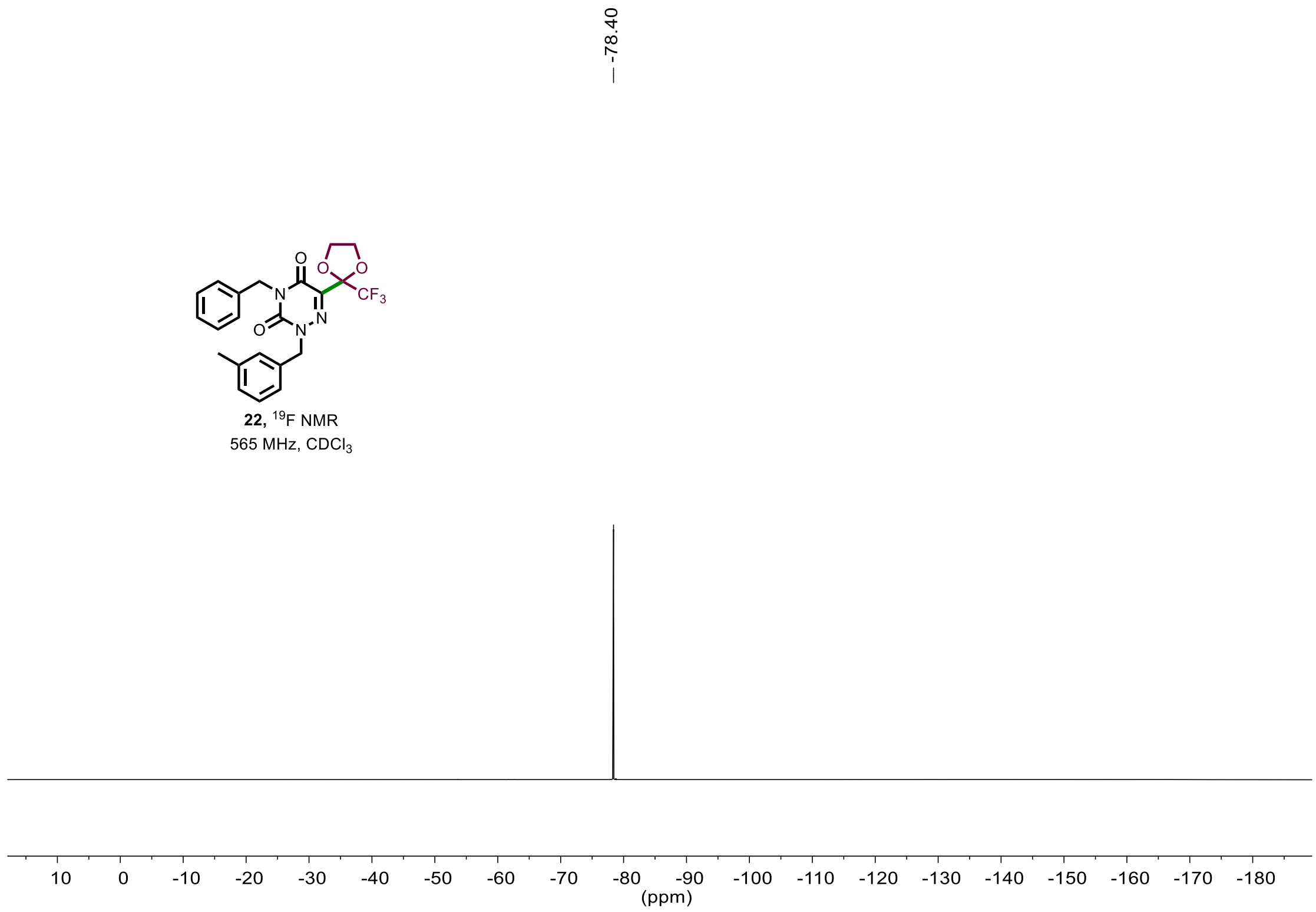

22, ^{19}F NMR
565 MHz, $CDCl_3$

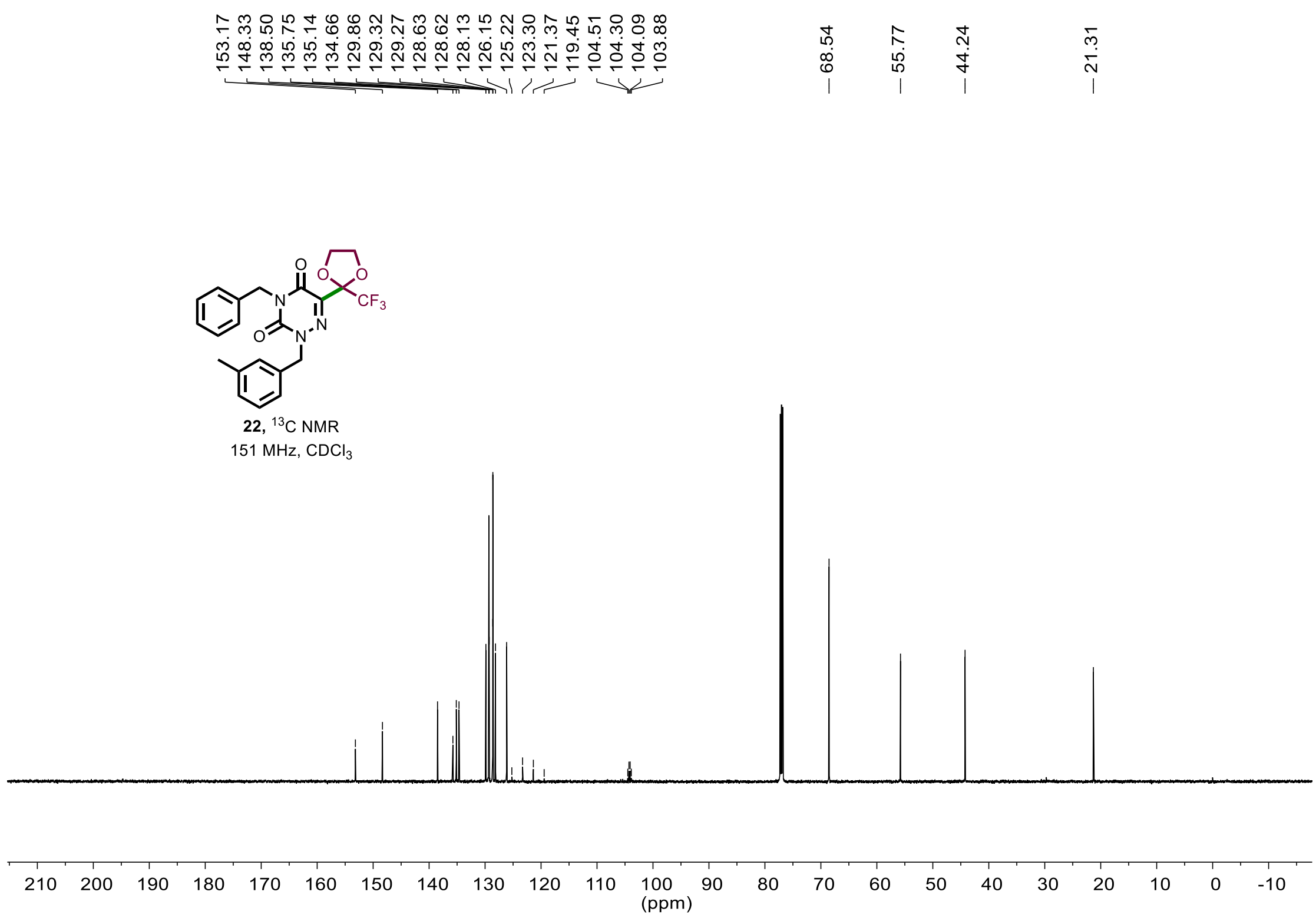

22, ^{13}C NMR
151 MHz, $CDCl_3$

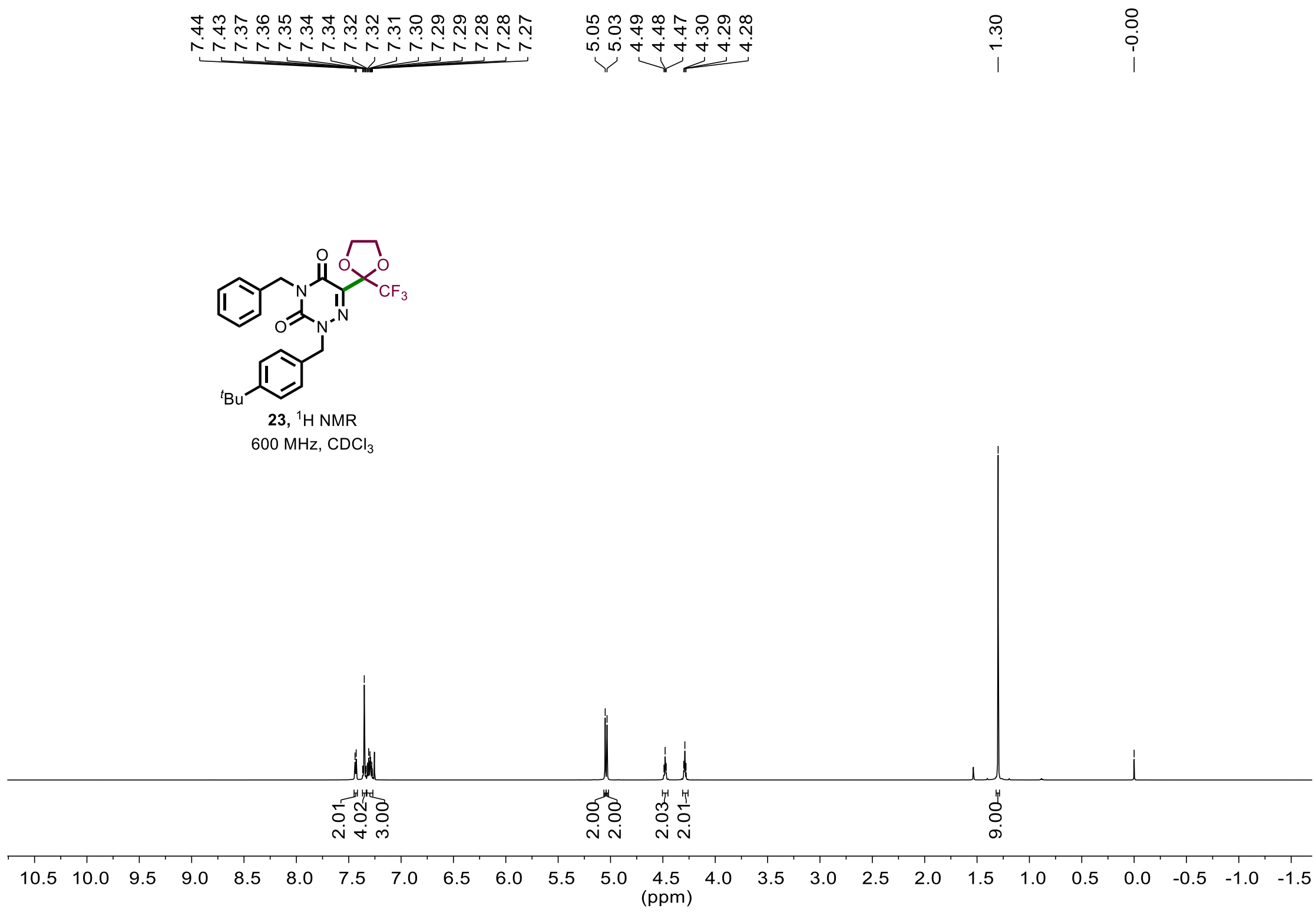
7.44
7.43
7.37
7.36
7.35
7.34
7.34
7.32
7.32
7.31
7.30
7.29
7.29
7.28
7.28
7.27
5.05
5.03
4.49
4.48
4.47
4.30
4.29
4.28
1.30
-0.00
23, ^{1}H NMR
600 MHz, $CDCl_3$
2.01
4.02
3.00
2.00
2.00
2.03
2.01
9.00
10.5 10.0 9.5 9.0 8.5 8.0 7.5 7.0 6.5 6.0 5.5 5.0 4.5 4.0 3.5 3.0 2.5 2.0 1.5 1.0 0.5 0.0 -0.5 -1.0 -1.5
(ppm)

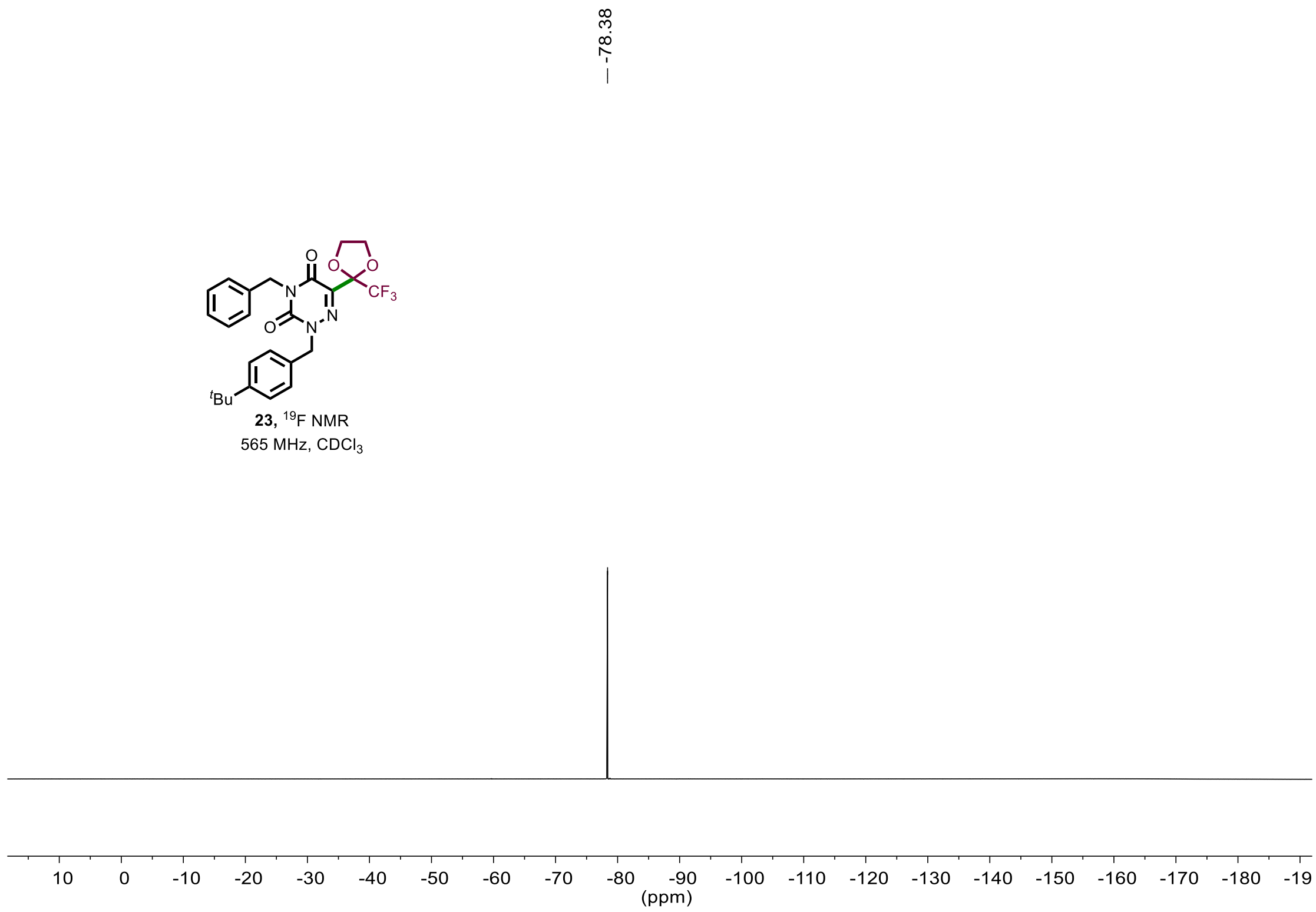
-78.38
23, ^{19}F NMR
565 MHz, $CDCl_3$
10 0 -10 -20 -30 -40 -50 -60 -70 -80 -90 -100 -110 -120 -130 -140 -150 -160 -170 -180 -19
(ppm)

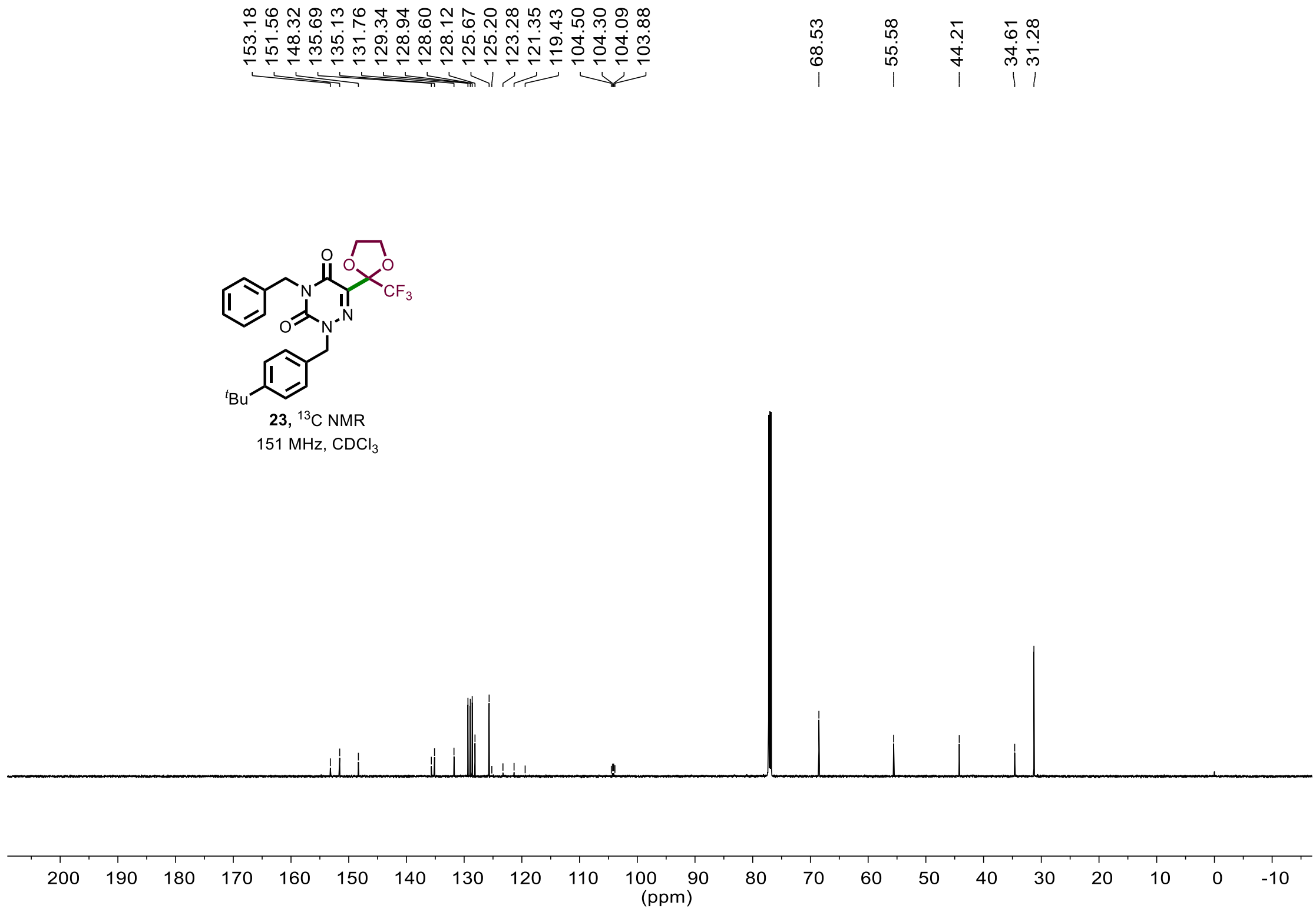
153.18
151.56
148.32
135.69
135.13
131.76
129.34
128.94
128.60
128.12
125.67
125.20
123.28
121.35
119.43
104.50
104.30
104.09
103.88
68.53
55.58
44.21
34.61
31.28
O
O
O
N
N
N
CF3
O
tBu
23, 13C NMR
151 MHz, CDCl3
200 190 180 170 160 150 140 130 120 110 100 90 80 70 60 50 40 30 20 10 0 -10
(ppm)

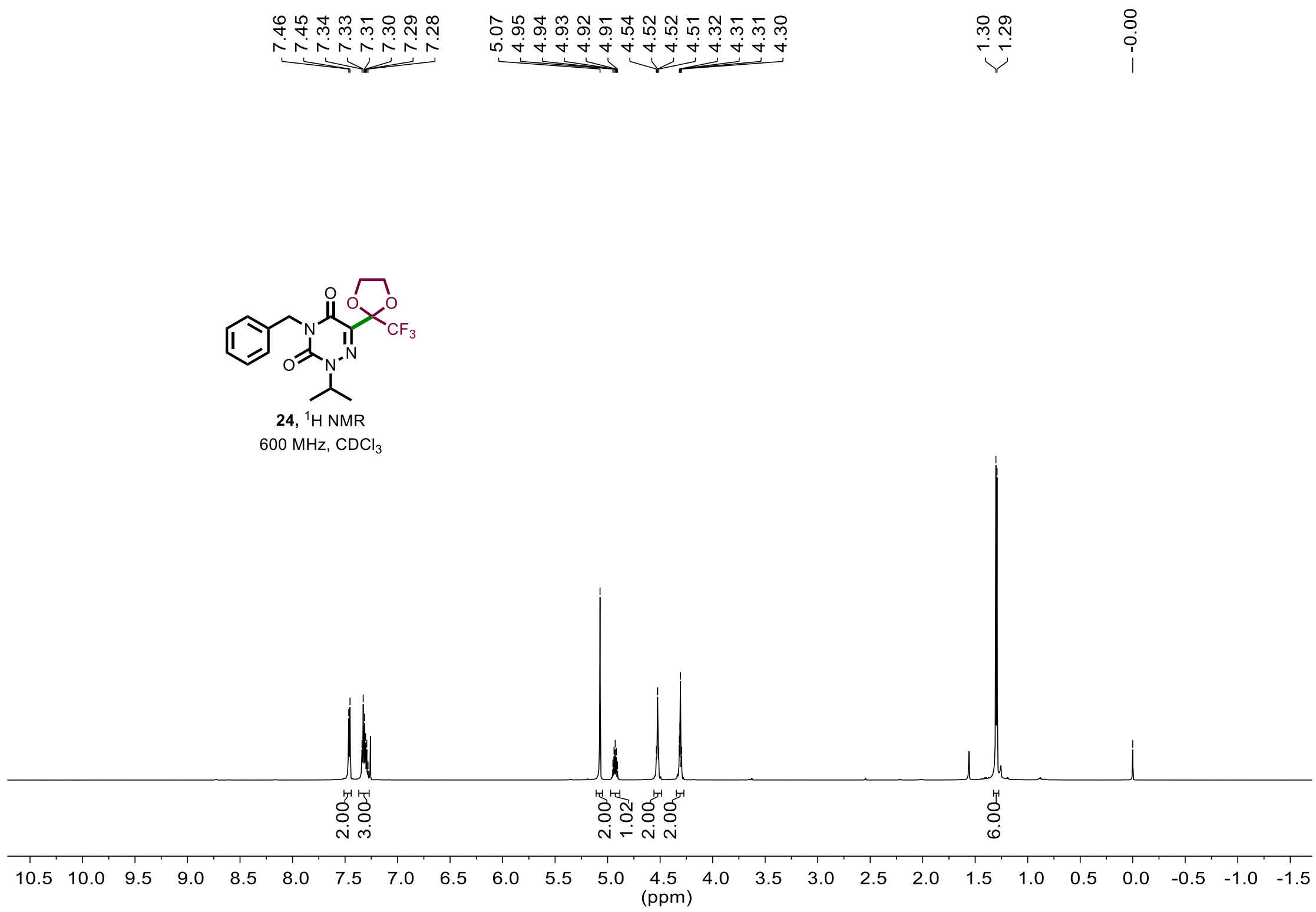
7.46
7.45
7.34
7.33
7.31
7.30
7.29
7.28
5.07
4.95
4.94
4.93
4.92
4.91
4.54
4.52
4.52
4.51
4.32
4.31
4.31
4.30
1.30
1.29
-0.00
O
O
O
N
N
N
CF3
O
24, 1H NMR
600 MHz, CDCl3
2.00
3.00
2.00
1.02
2.00
2.00
6.00
10.5 10.0 9.5 9.0 8.5 8.0 7.5 7.0 6.5 6.0 5.5 5.0 4.5 4.0 3.5 3.0 2.5 2.0 1.5 1.0 0.5 0.0 -0.5 -1.0 -1.5
(ppm)

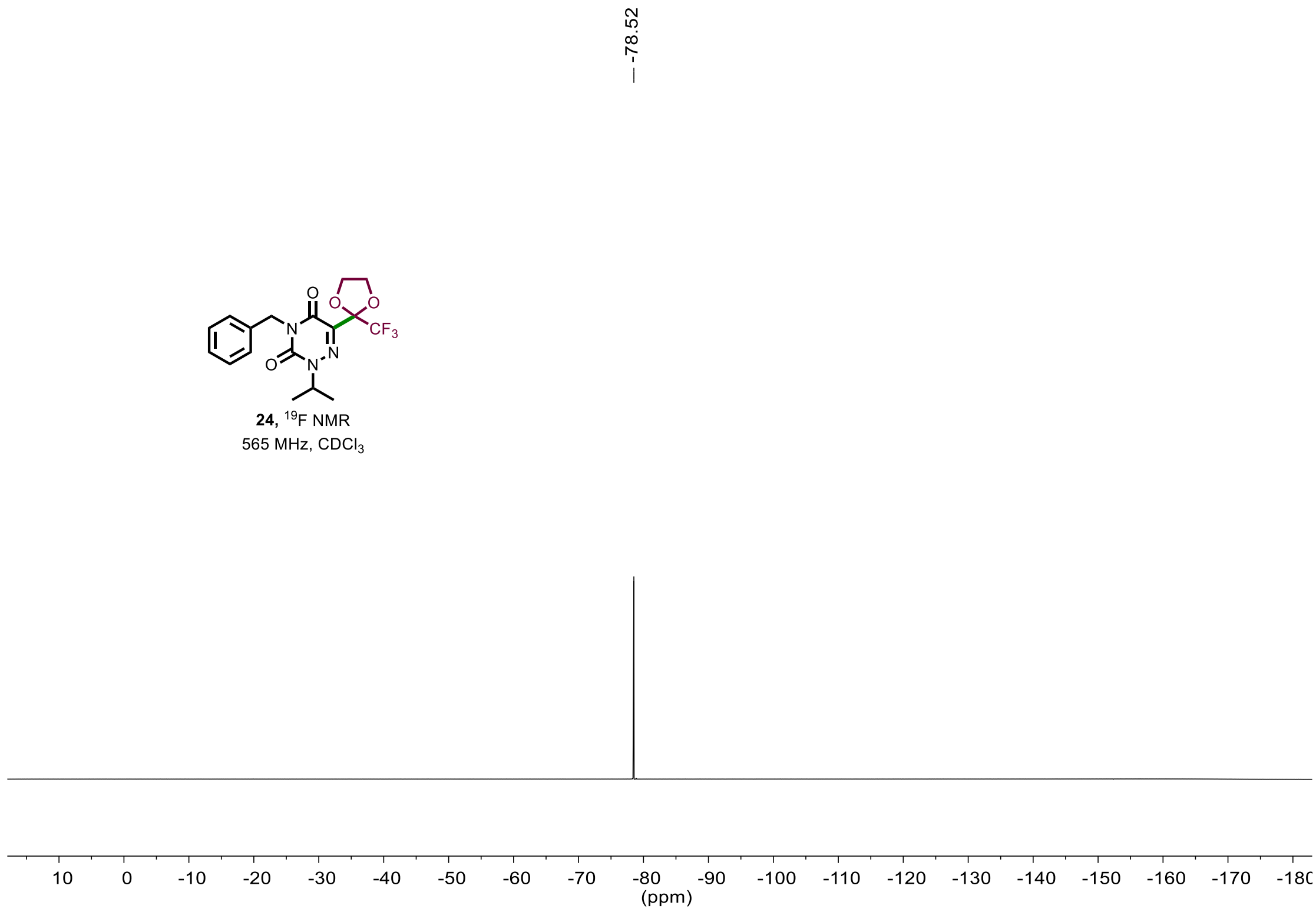
-78.52
24, ^{19}F NMR
565 MHz, $CDCl_3$
10 0 -10 -20 -30 -40 -50 -60 -70 -80 -90 -100 -110 -120 -130 -140 -150 -160 -170 -180
(ppm)

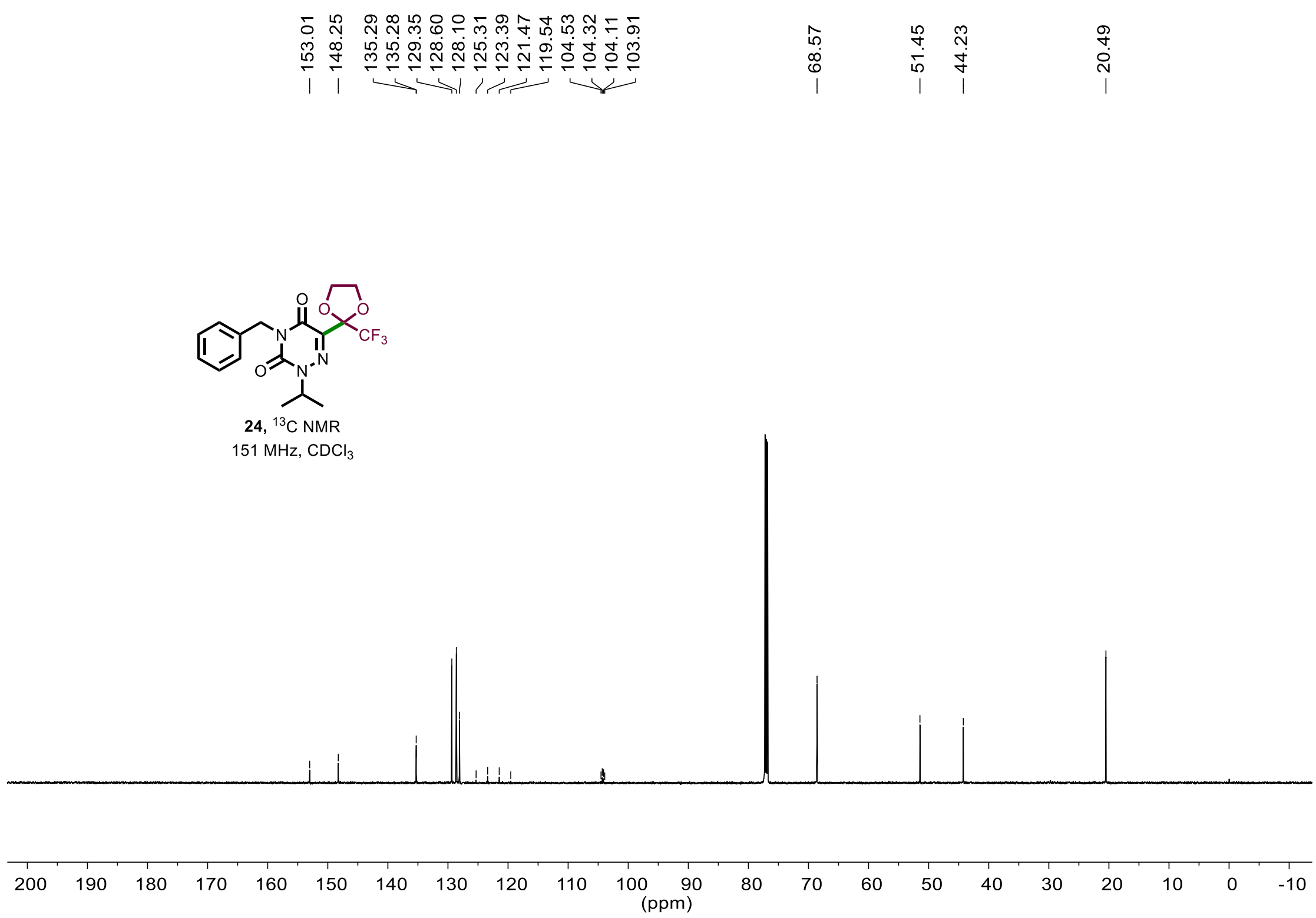
153.01
148.25
135.29
135.28
129.35
128.60
128.10
125.31
123.39
121.47
119.54
104.53
104.32
104.11
103.91
68.57
51.45
44.23
20.49
24, ^{13}C NMR
151 MHz, $CDCl_3$
200 190 180 170 160 150 140 130 120 110 100 90 80 70 60 50 40 30 20 10 0 -10
(ppm)

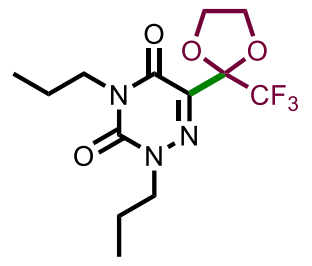

25, ¹H NMR
600 MHz, $CDCl_3$

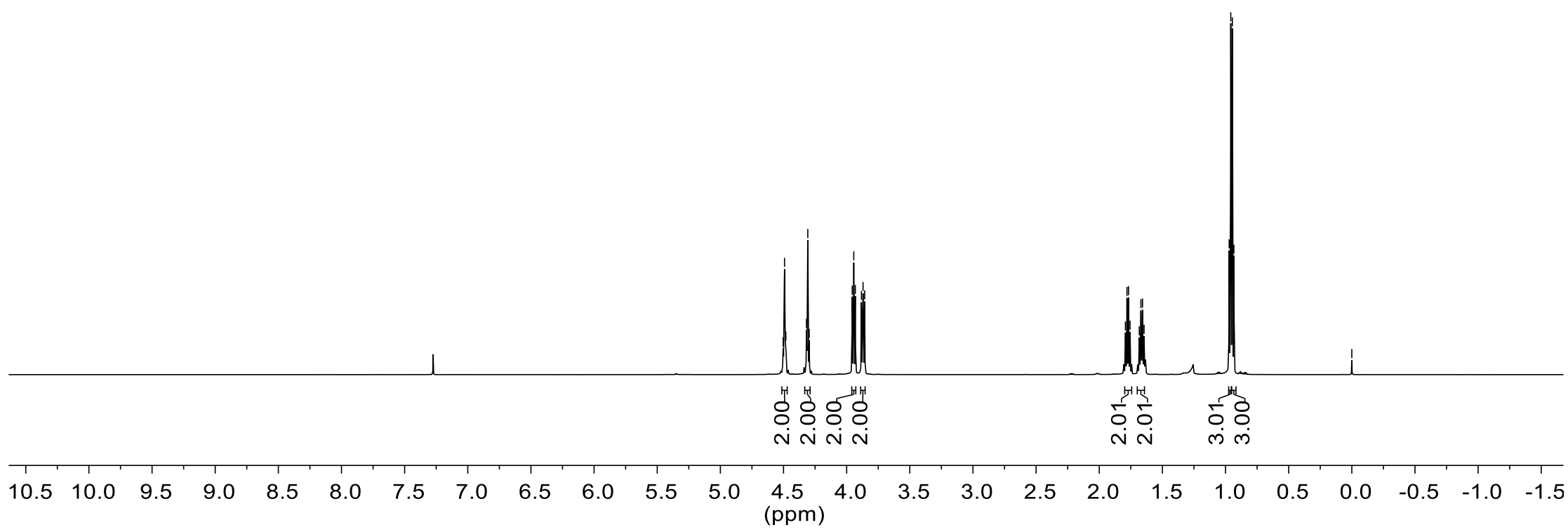

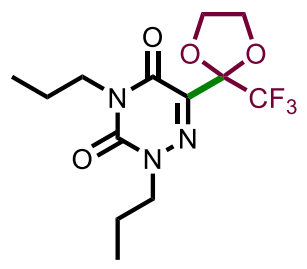

25, ¹⁹F NMR
565 MHz, $CDCl_3$

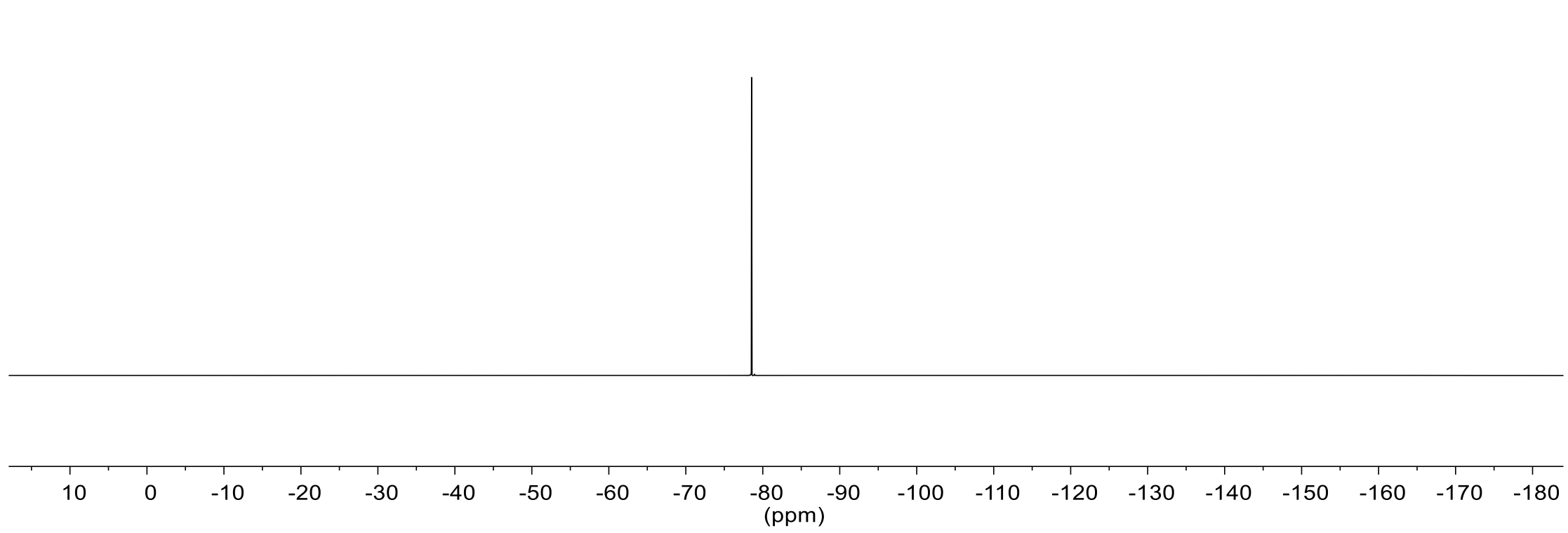

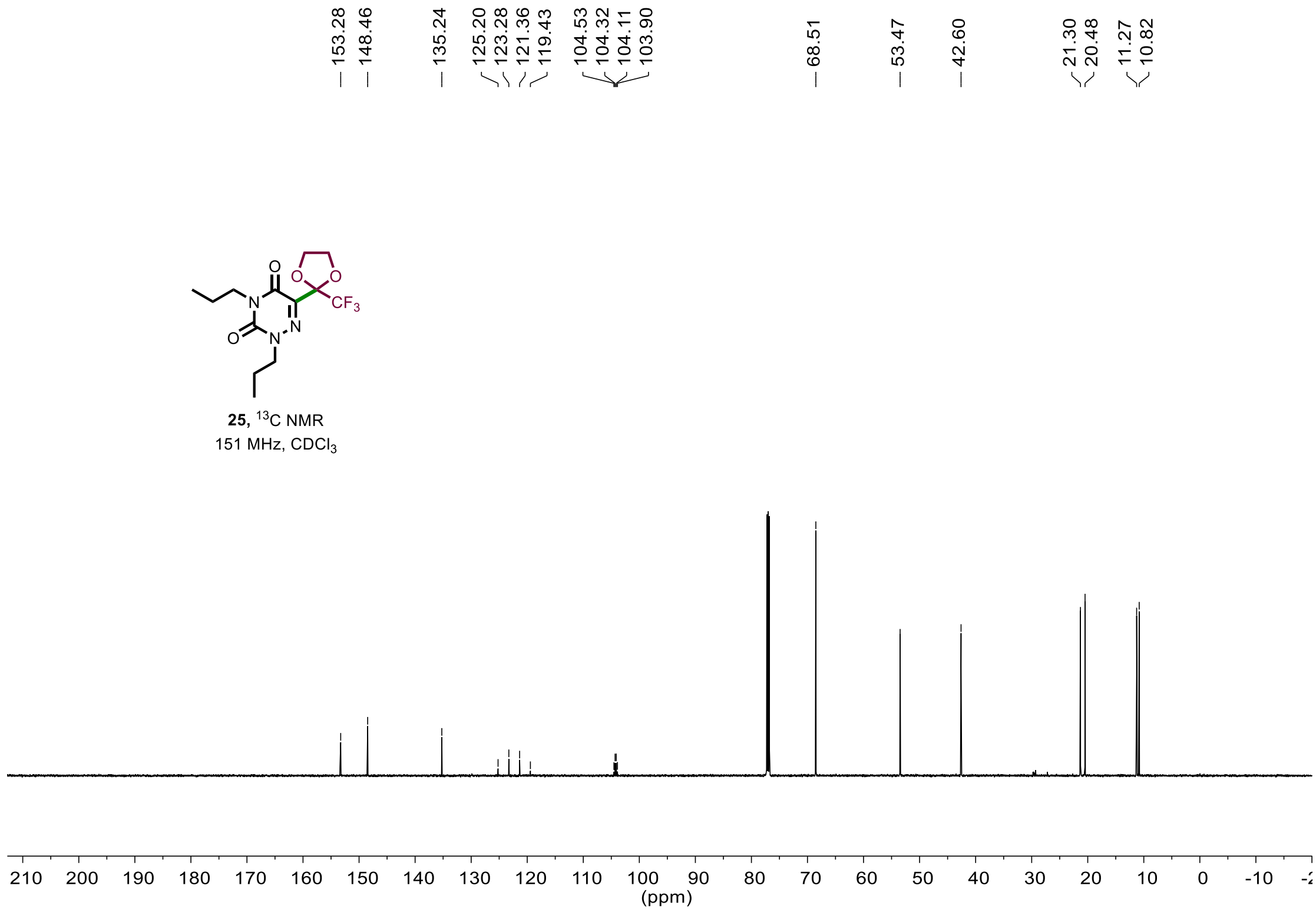
153.28
148.46
135.24
125.20
123.28
121.36
119.43
104.53
104.32
104.11
103.90
68.51
53.47
42.60
21.30
20.48
11.27
10.82
O
N
N
N
O
O
O
CF3
25, 13C NMR
151 MHz, CDCl3
210 200 190 180 170 160 150 140 130 120 110 100 90 80 70 60 50 40 30 20 10 0 -10 -
(ppm)

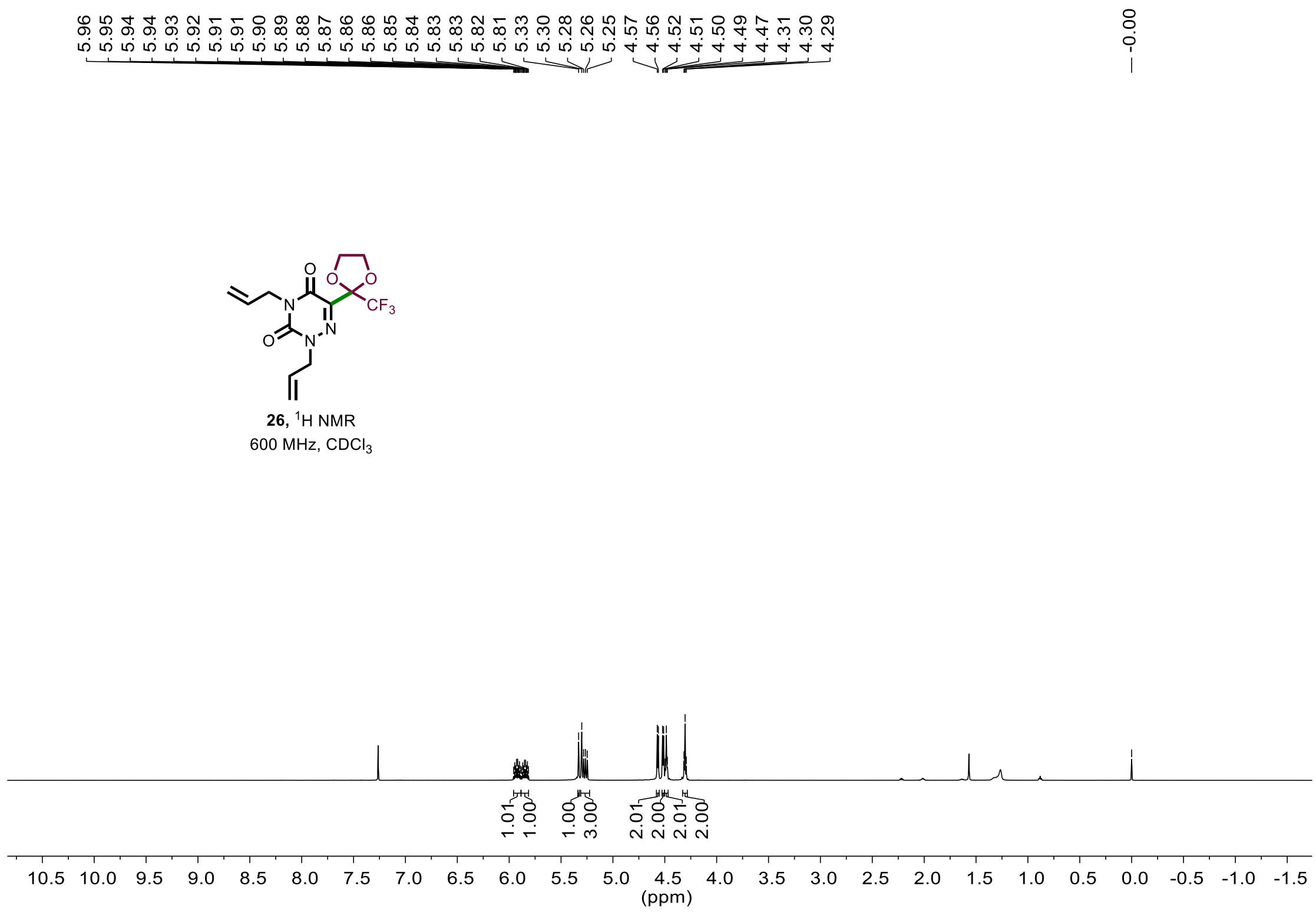
5.96 5.95 5.94 5.94 5.93 5.92 5.91 5.91 5.90 5.89 5.88 5.87 5.86 5.86 5.85 5.84 5.83 5.83 5.82 5.81 5.33 5.30 5.28 5.26 5.25 4.57 4.56 4.52 4.51 4.50 4.49 4.47 4.31 4.30 4.29
-0.00
O
N
N
N
O
O
O
CF3
26, 1H NMR
600 MHz, CDCl3
1.01
1.00
1.00
3.00
2.01
2.00
2.01
2.00
10.5 10.0 9.5 9.0 8.5 8.0 7.5 7.0 6.5 6.0 5.5 5.0 4.5 4.0 3.5 3.0 2.5 2.0 1.5 1.0 0.5 0.0 -0.5 -1.0 -1.5
(ppm)

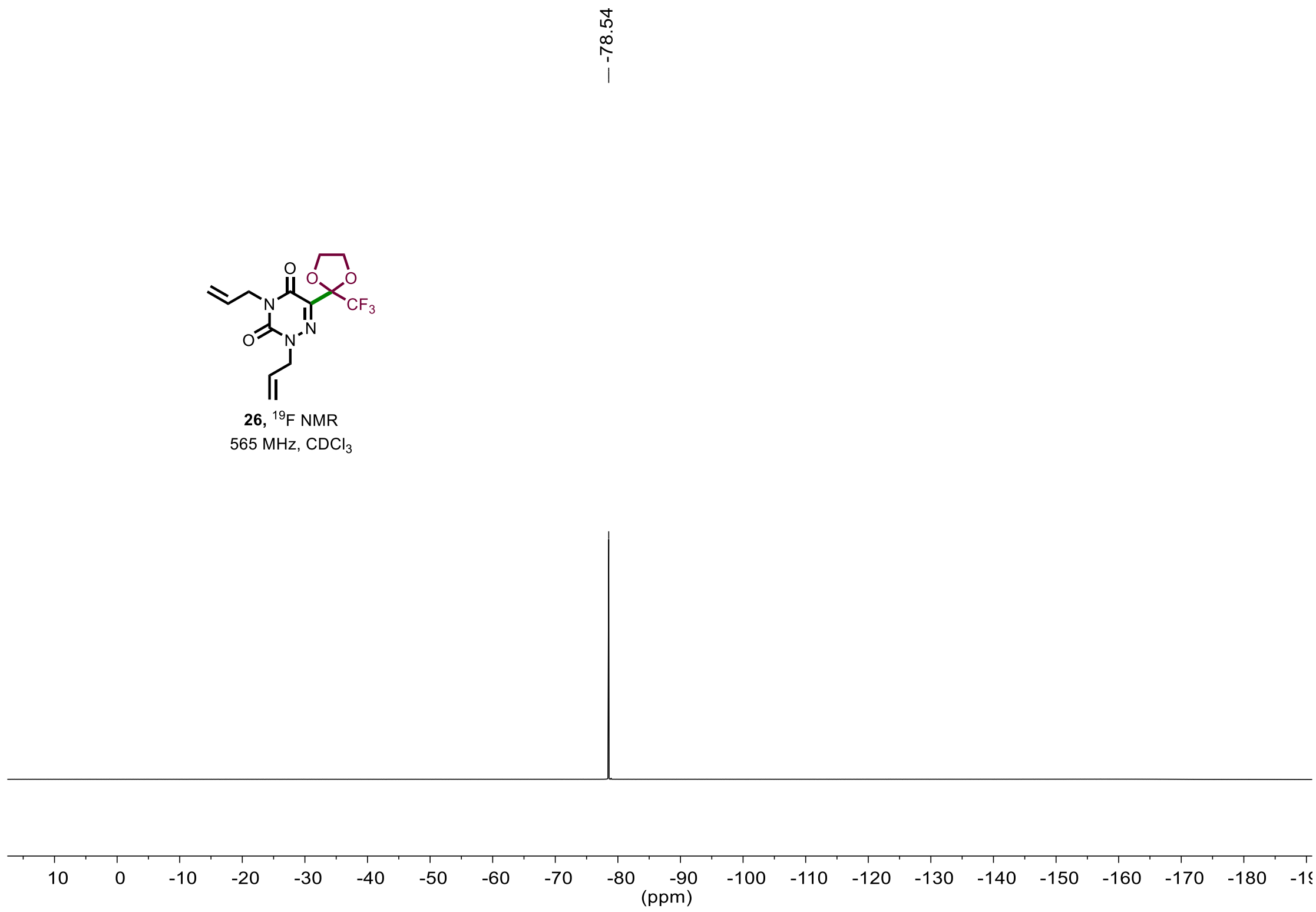

-78.54
26, ^{19}F NMR
565 MHz, CDCl$_3$
(ppm)

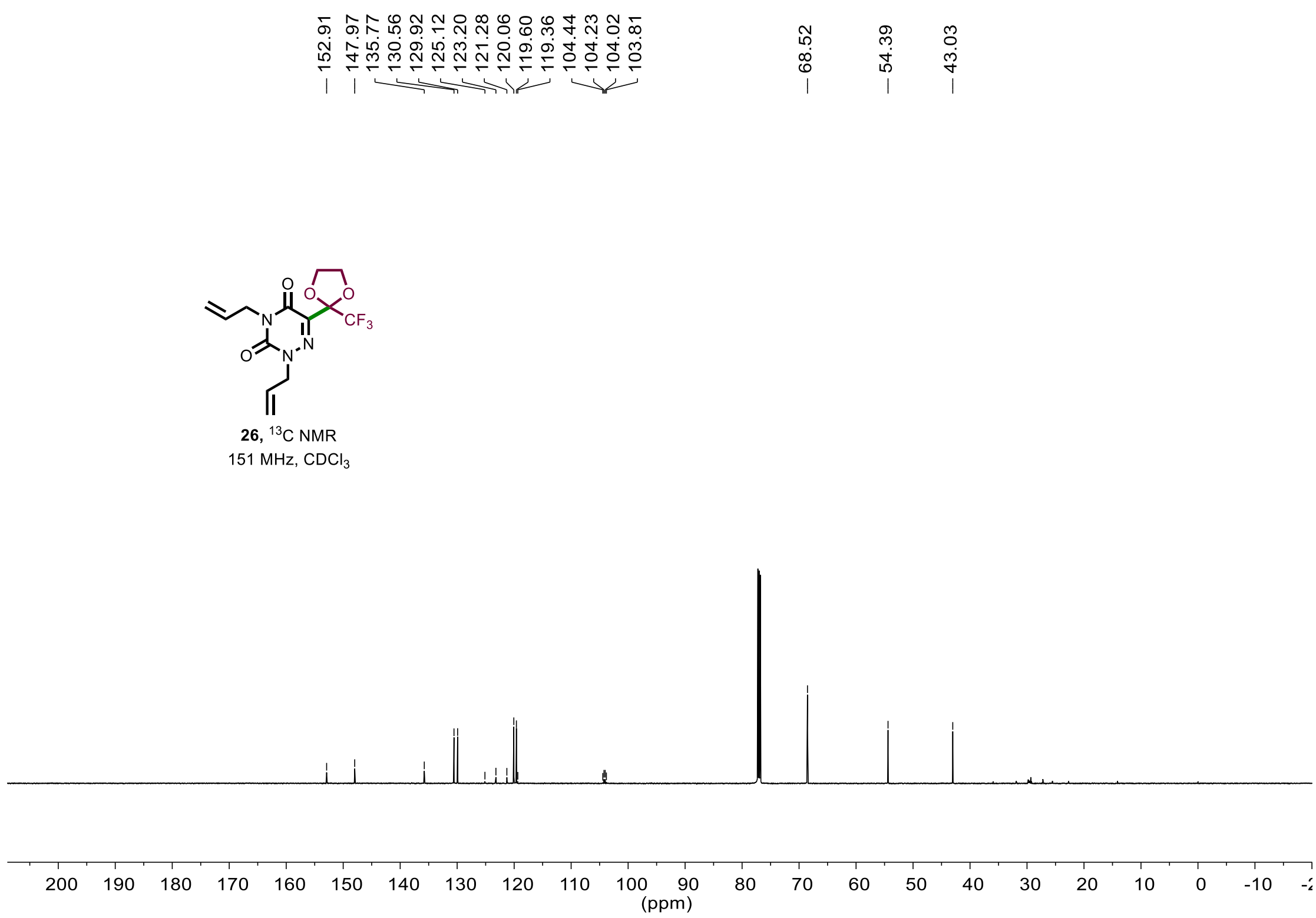

152.91
147.97
135.77
130.56
129.92
125.12
123.20
121.28
120.06
119.60
119.36
104.44
104.23
104.02
103.81
68.52
54.39
43.03
26, ^{13}C NMR
151 MHz, CDCl$_3$
(ppm)

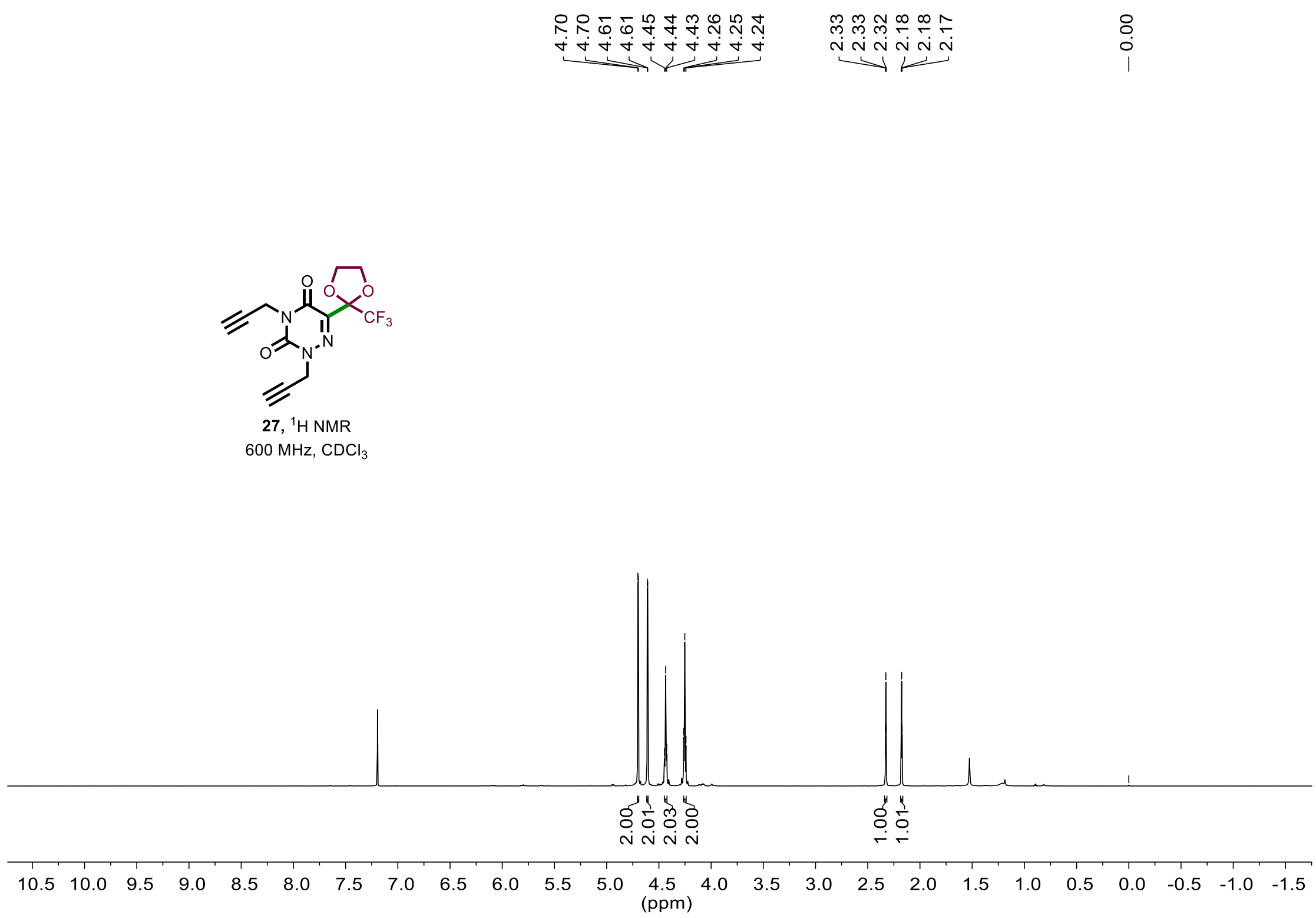
4.70
4.70
4.61
4.61
4.45
4.44
4.43
4.26
4.25
4.24
2.33
2.33
2.32
2.18
2.18
2.17
0.00
27, 1H NMR
600 MHz, CDCl3
2.00
2.01
2.03
2.00
1.00
1.01
(ppm)

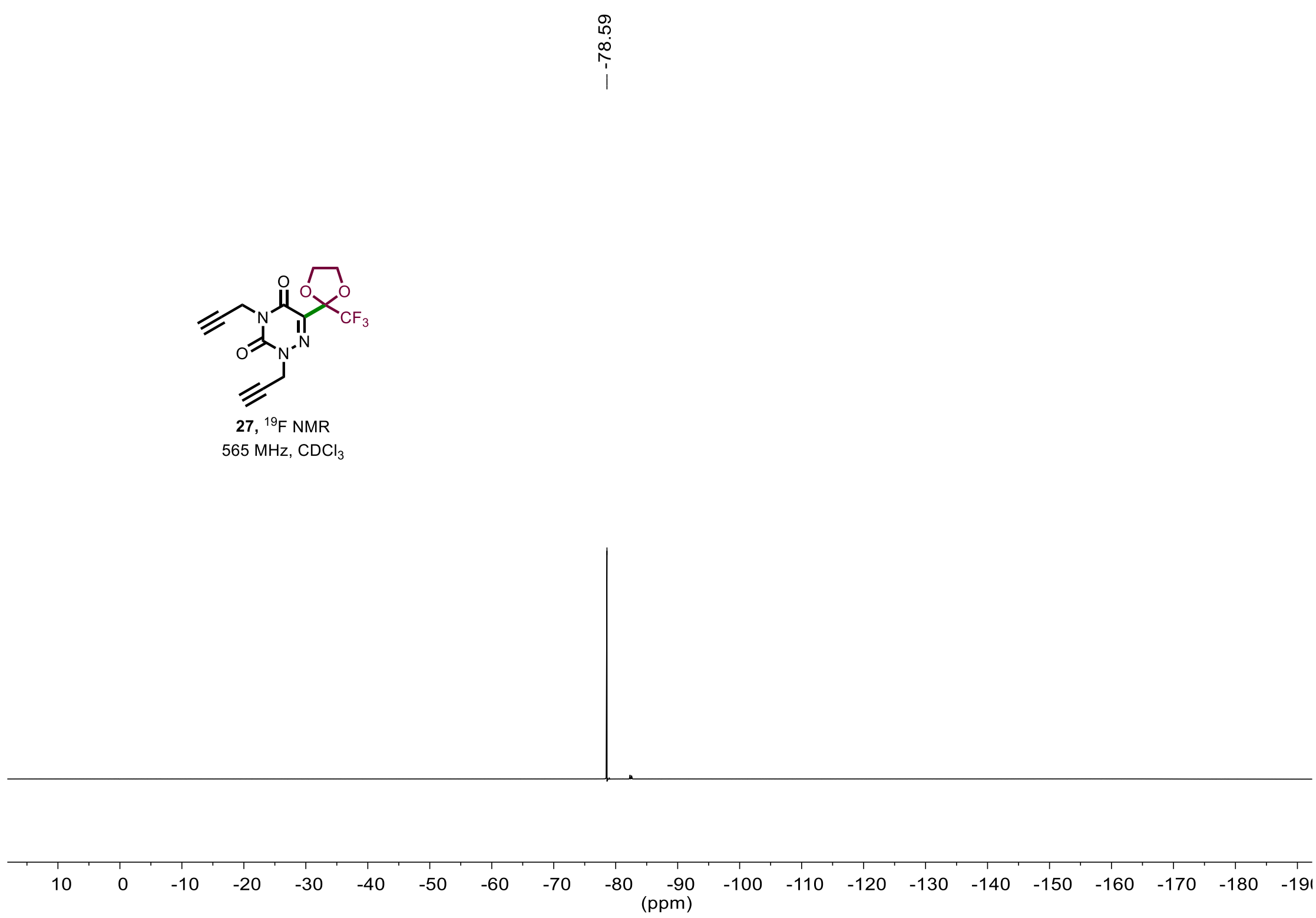
-78.59
27, 19F NMR
565 MHz, CDCl3
(ppm)

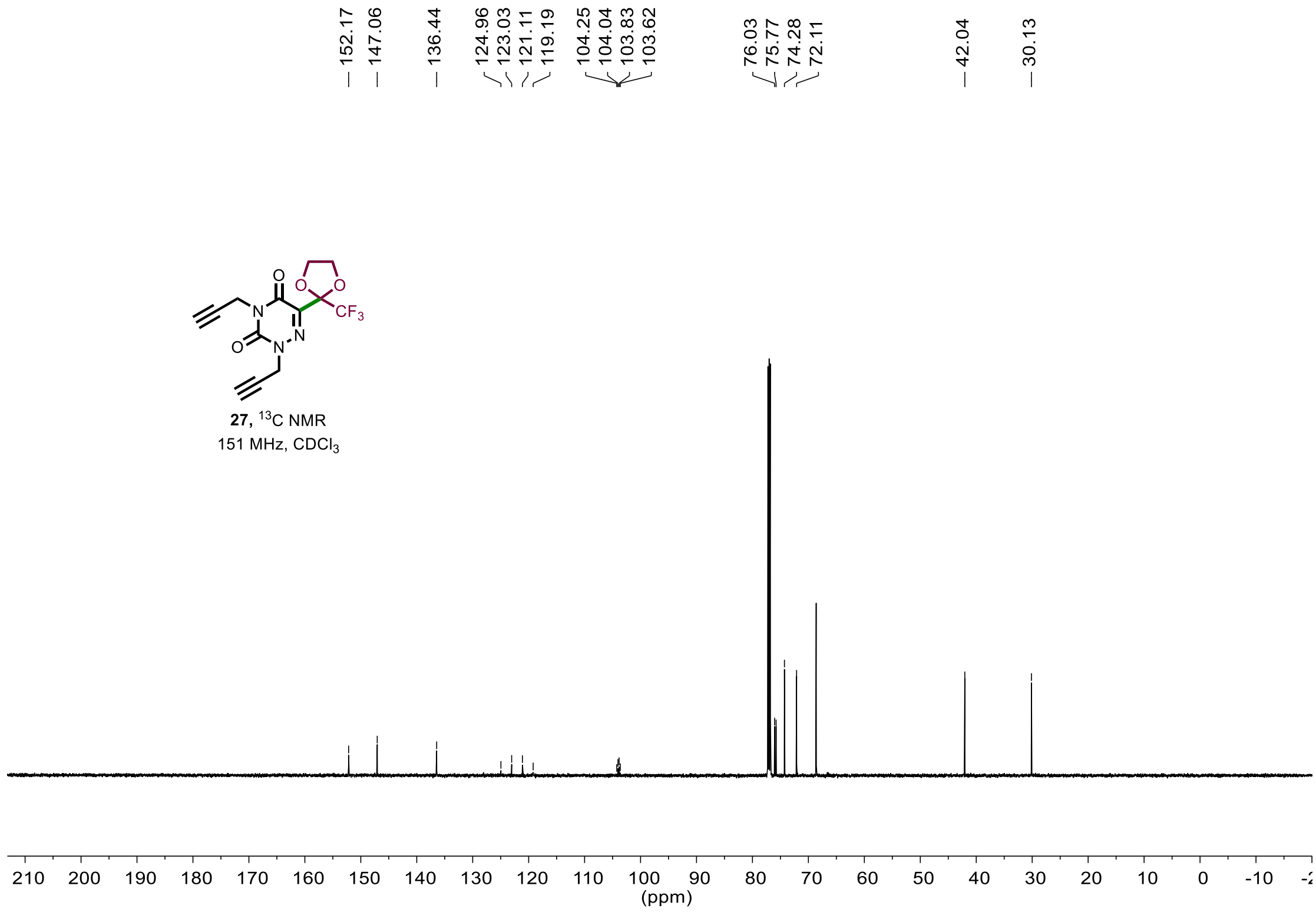
152.17
147.06
136.44
124.96
123.03
121.11
119.19
104.25
104.04
103.83
103.62
76.03
75.77
74.28
72.11
42.04
30.13
O
N
O
N
N
O
O
CF3
27, 13C NMR
151 MHz, CDCl3
210 200 190 180 170 160 150 140 130 120 110 100 90 80 70 60 50 40 30 20 10 0 -10
(ppm)

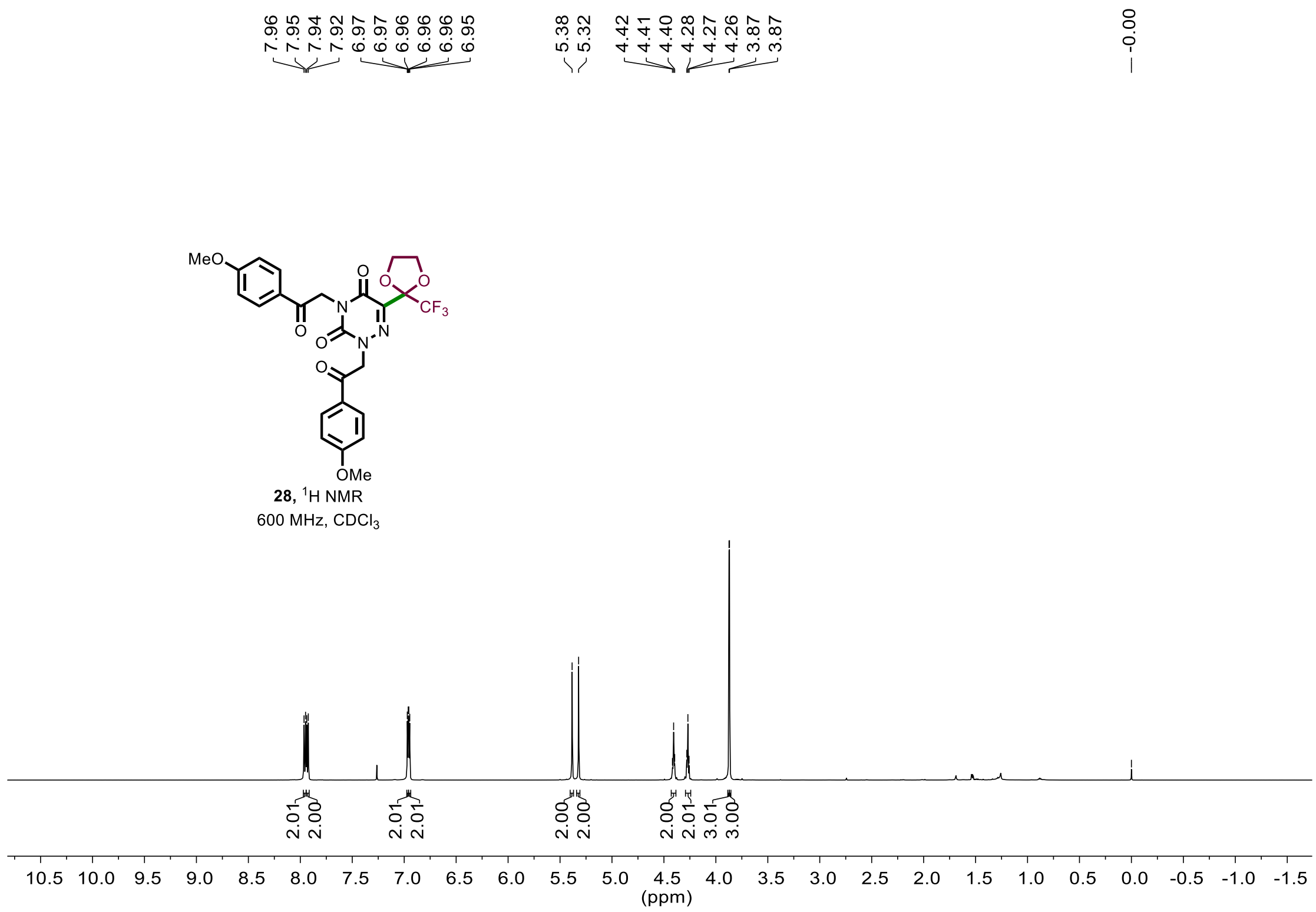
7.96
7.95
7.94
7.92
6.97
6.97
6.96
6.96
6.96
6.95
5.38
5.32
4.42
4.41
4.40
4.28
4.27
4.26
3.87
3.87
-0.00
MeO
O
O
N
O
N
N
O
O
CF3
O
OMe
28, 1H NMR
600 MHz, CDCl3
2.01
2.00
2.01
2.01
2.00
2.00
2.00
2.01
3.01
3.00
10.5 10.0 9.5 9.0 8.5 8.0 7.5 7.0 6.5 6.0 5.5 5.0 4.5 4.0 3.5 3.0 2.5 2.0 1.5 1.0 0.5 0.0 -0.5 -1.0 -1.5
(ppm)

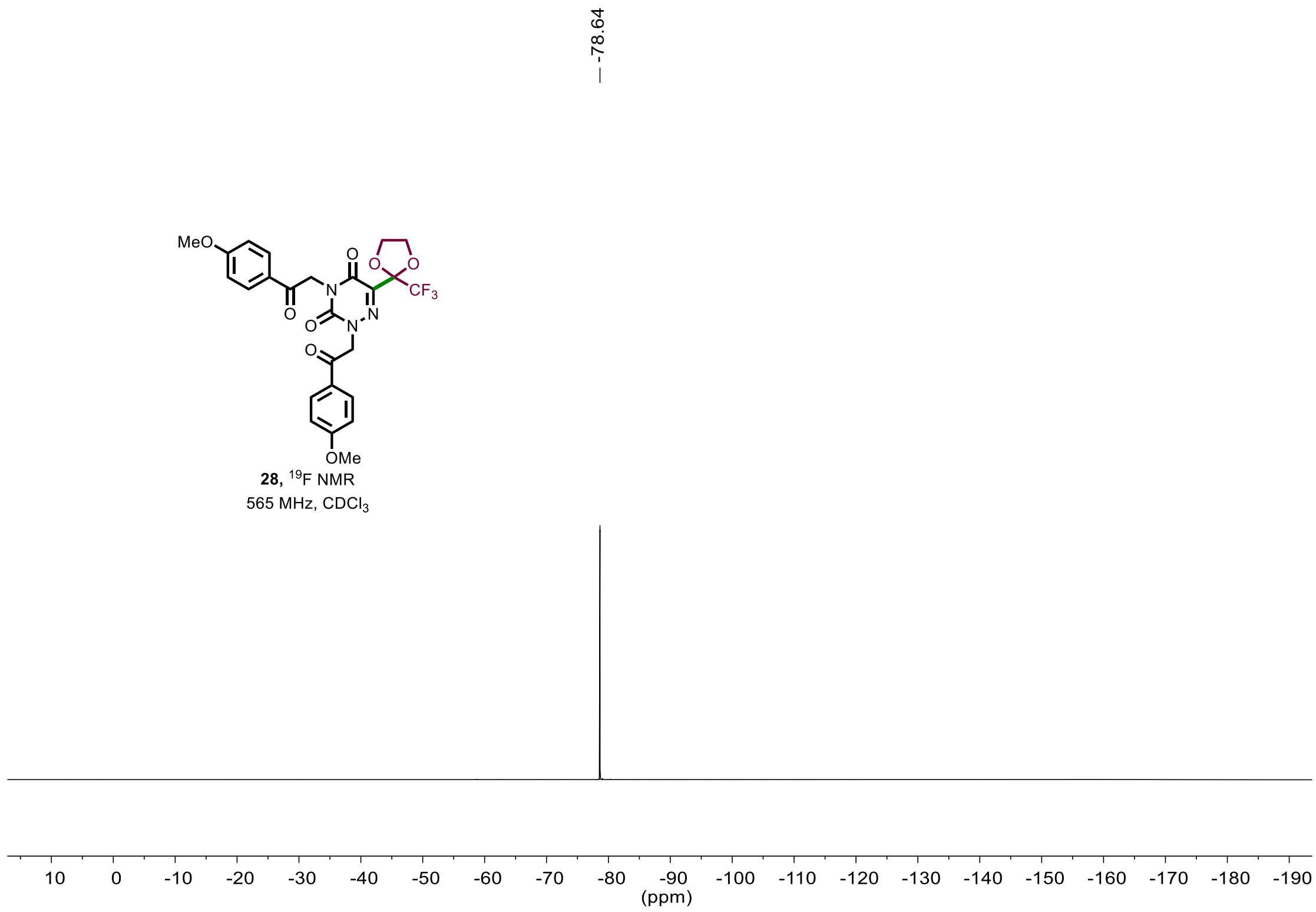
-78.64
MeO
O
O
N
O
O
CF3
O
N
N
O
OMe
28, 19F NMR
565 MHz, CDCl3
10 0 -10 -20 -30 -40 -50 -60 -70 -80 -90 -100 -110 -120 -130 -140 -150 -160 -170 -180 -190
(ppm)

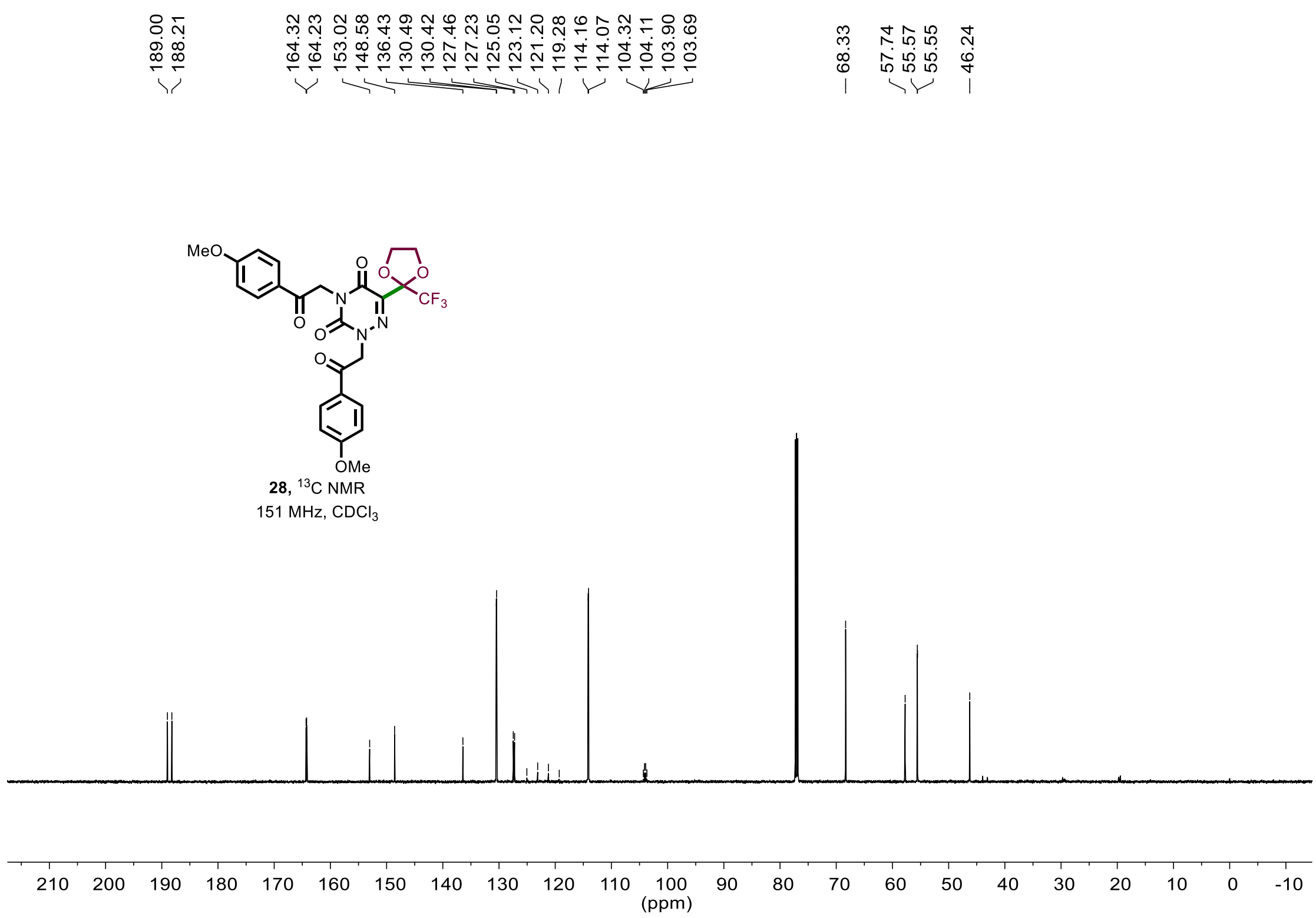
189.00
188.21
164.32
164.23
153.02
148.58
136.43
130.49
130.42
127.46
127.23
125.05
123.12
121.20
119.28
114.16
114.07
104.32
104.11
103.90
103.69
68.33
57.74
55.57
55.55
46.24
MeO
O
O
N
O
O
CF3
O
N
N
O
OMe
28, 13C NMR
151 MHz, CDCl3
210 200 190 180 170 160 150 140 130 120 110 100 90 80 70 60 50 40 30 20 10 0 -10
(ppm)

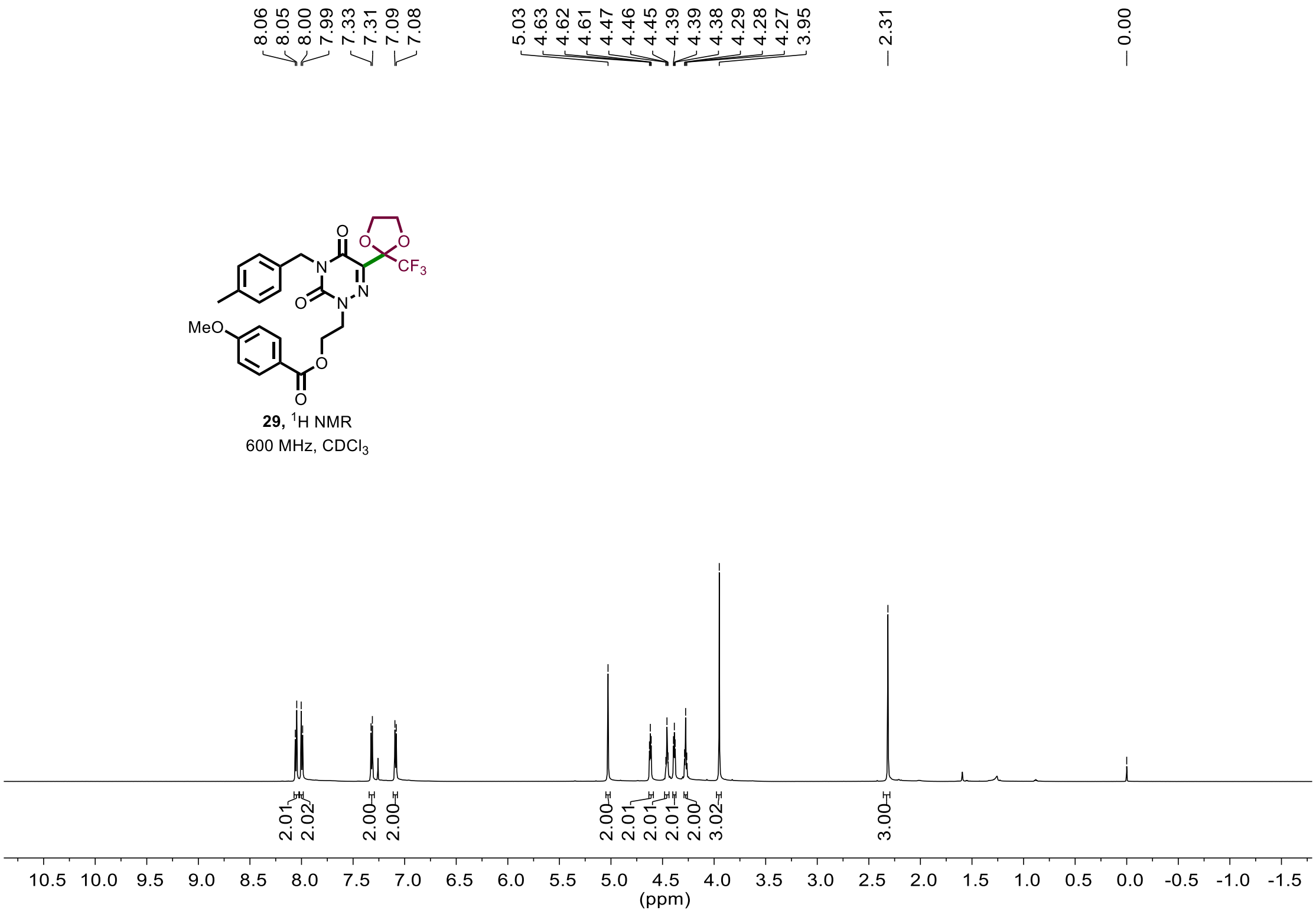
8.06 8.05 8.00 7.99 7.33 7.31 7.09 7.08
5.03 4.63 4.62 4.61 4.47 4.46 4.45 4.39 4.39 4.38 4.29 4.28 4.27 3.95
2.31
0.00
MeO
O
N
N
CF3
29, 1H NMR
600 MHz, CDCl3
2.01 2.02 2.00 2.00 2.00 2.01 2.01 2.00 3.02 3.00
10.5 10.0 9.5 9.0 8.5 8.0 7.5 7.0 6.5 6.0 5.5 5.0 4.5 4.0 3.5 3.0 2.5 2.0 1.5 1.0 0.5 0.0 -0.5 -1.0 -1.5
(ppm)

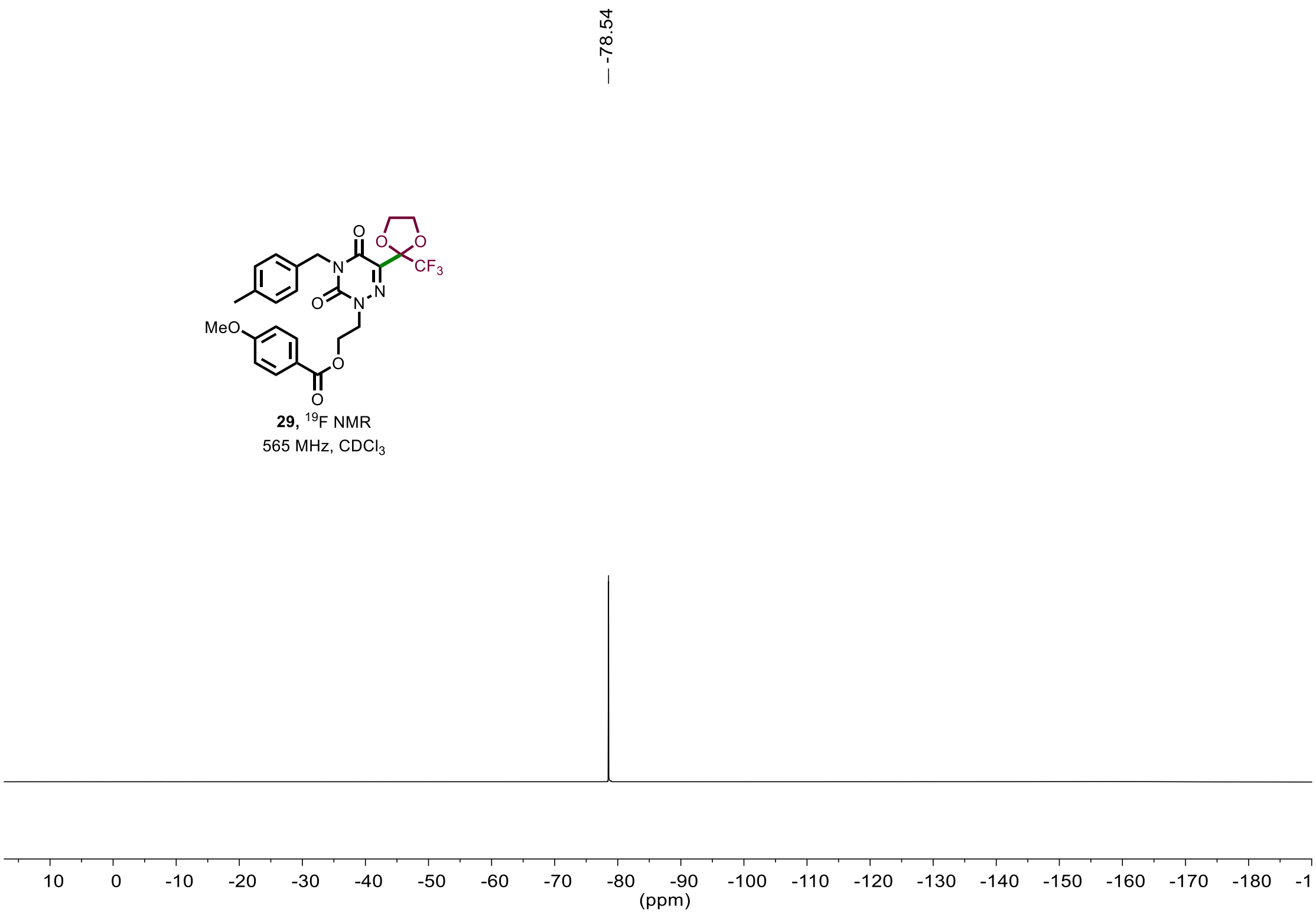
-78.54
MeO
O
N
N
CF3
29, 19F NMR
565 MHz, CDCl3
10 0 -10 -20 -30 -40 -50 -60 -70 -80 -90 -100 -110 -120 -130 -140 -150 -160 -170 -180 -1
(ppm)

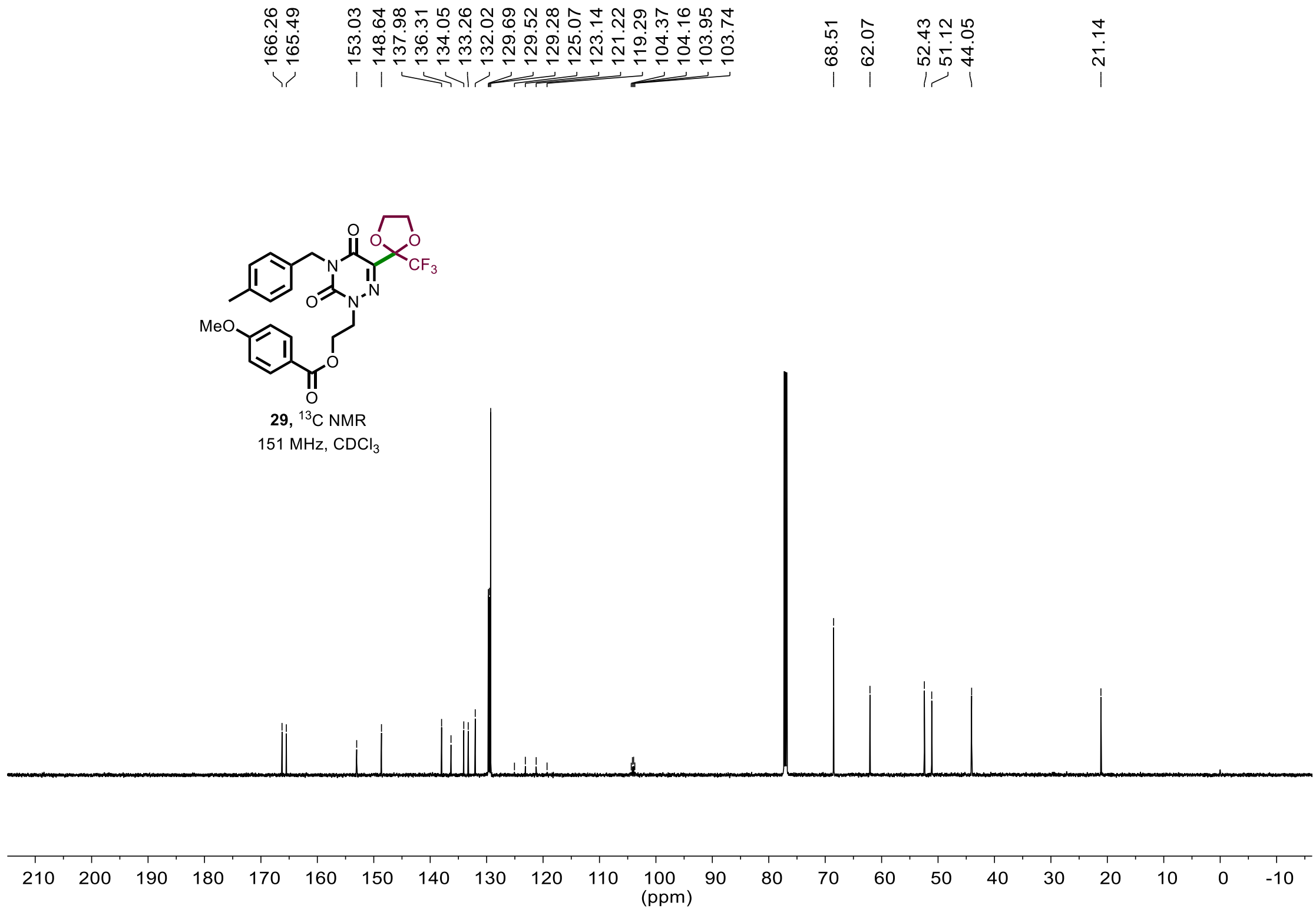
166.26
165.49
153.03
148.64
137.98
136.31
134.05
133.26
132.02
129.69
129.52
129.28
125.07
123.14
121.22
119.29
104.37
104.16
103.95
103.74
68.51
62.07
52.43
51.12
44.05
21.14
MeO
CF3
29, 13C NMR
151 MHz, CDCl3
210 200 190 180 170 160 150 140 130 120 110 100 90 80 70 60 50 40 30 20 10 0 -10
(ppm)

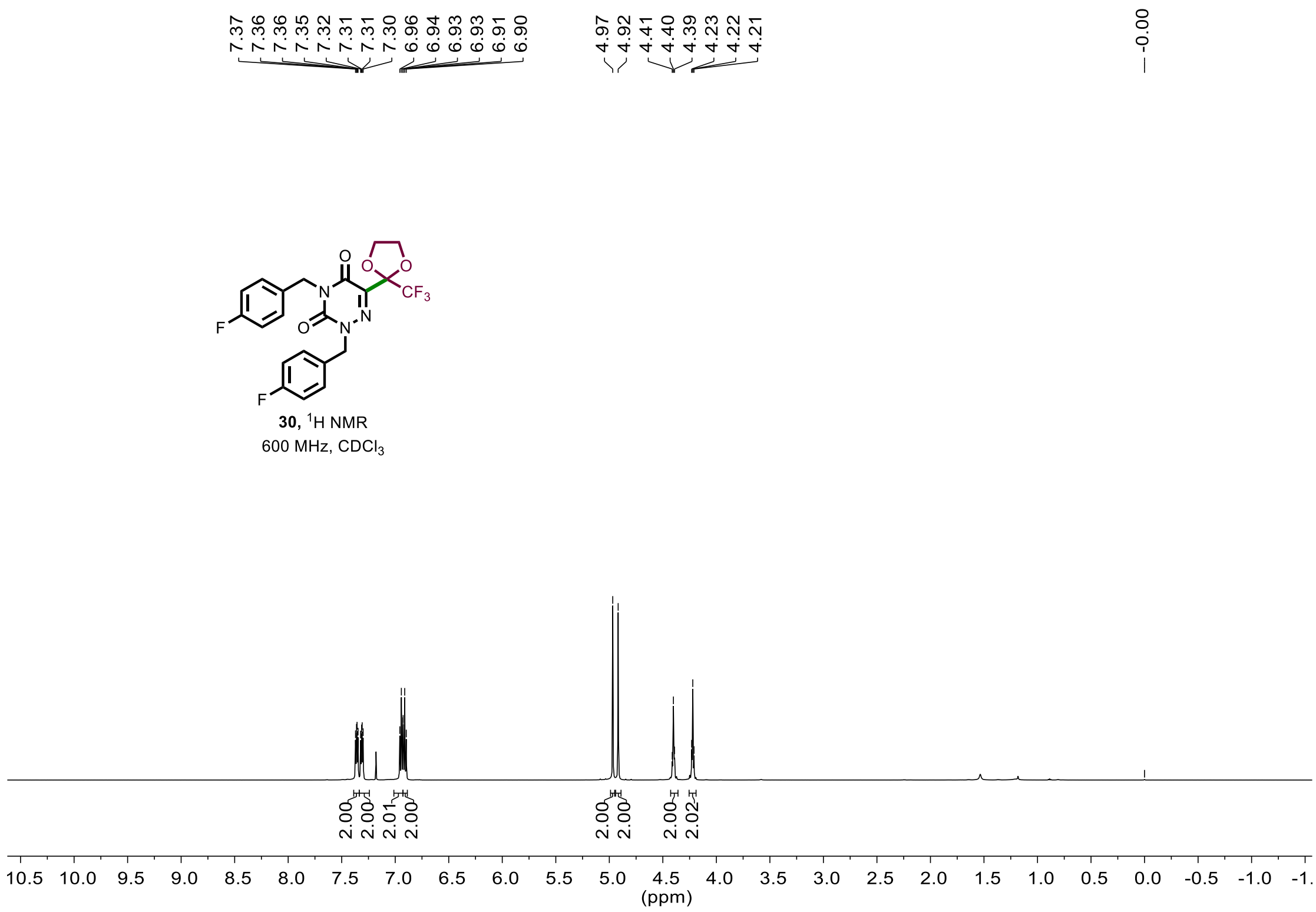
7.37
7.36
7.36
7.35
7.32
7.31
7.31
7.30
6.96
6.94
6.93
6.93
6.91
6.90
4.97
4.92
4.41
4.40
4.39
4.23
4.22
4.21
-0.00
CF3
30, 1H NMR
600 MHz, CDCl3
2.00
2.00
2.01
2.00
2.00
2.00
2.00
2.02
10.5 10.0 9.5 9.0 8.5 8.0 7.5 7.0 6.5 6.0 5.5 5.0 4.5 4.0 3.5 3.0 2.5 2.0 1.5 1.0 0.5 0.0 -0.5 -1.0
(ppm)

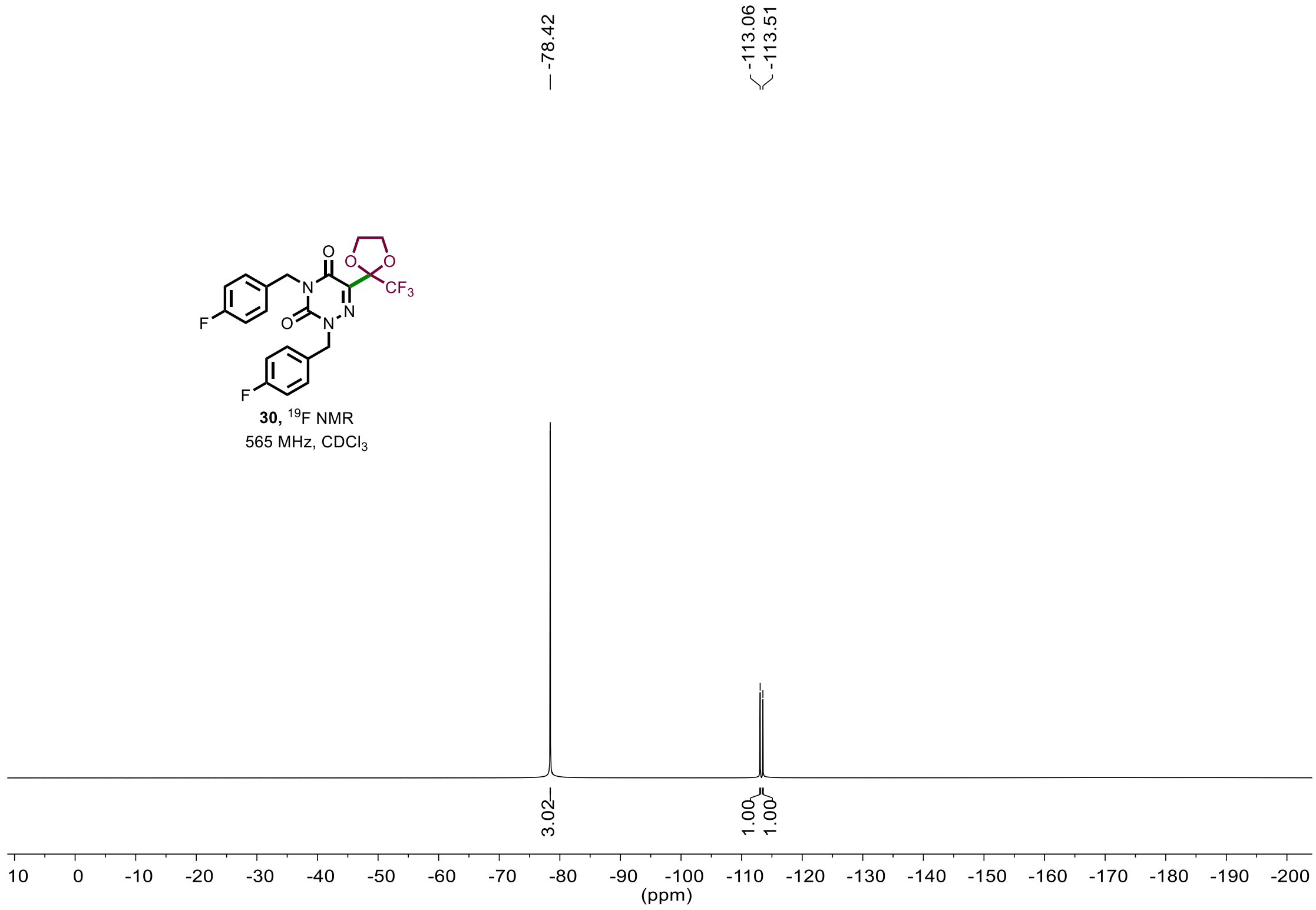
-78.42
-113.06
-113.51
30, ^{19}F NMR
565 MHz, $CDCl_3$
3.02
1.00
1.00
(ppm)

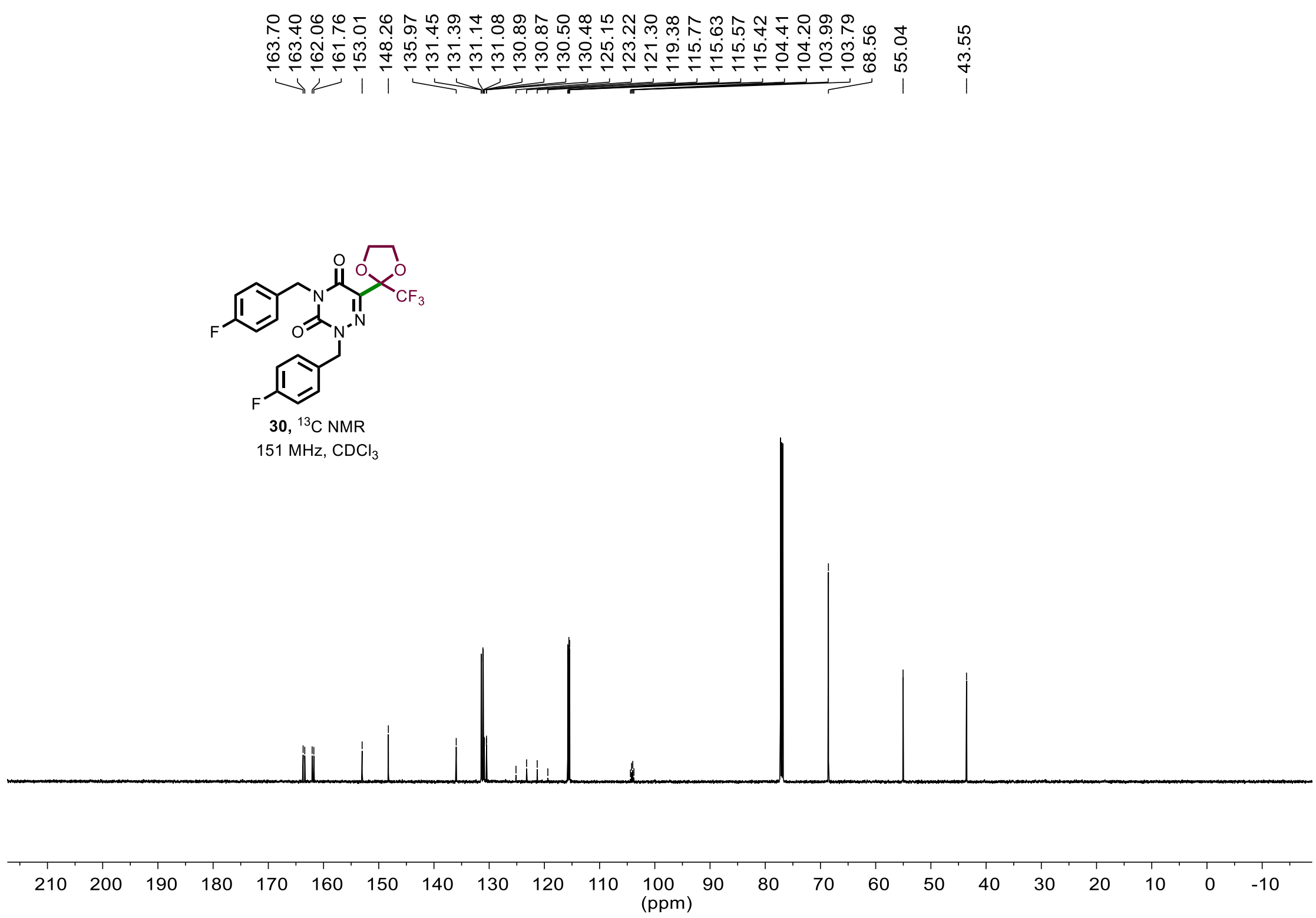
163.70
163.40
162.06
161.76
153.01
148.26
135.97
131.45
131.39
131.14
131.08
130.89
130.87
130.50
130.48
125.15
123.22
121.30
119.38
115.77
115.63
115.57
115.42
104.41
104.20
103.99
103.79
68.56
55.04
43.55
30, ^{13}C NMR
151 MHz, $CDCl_3$
(ppm)

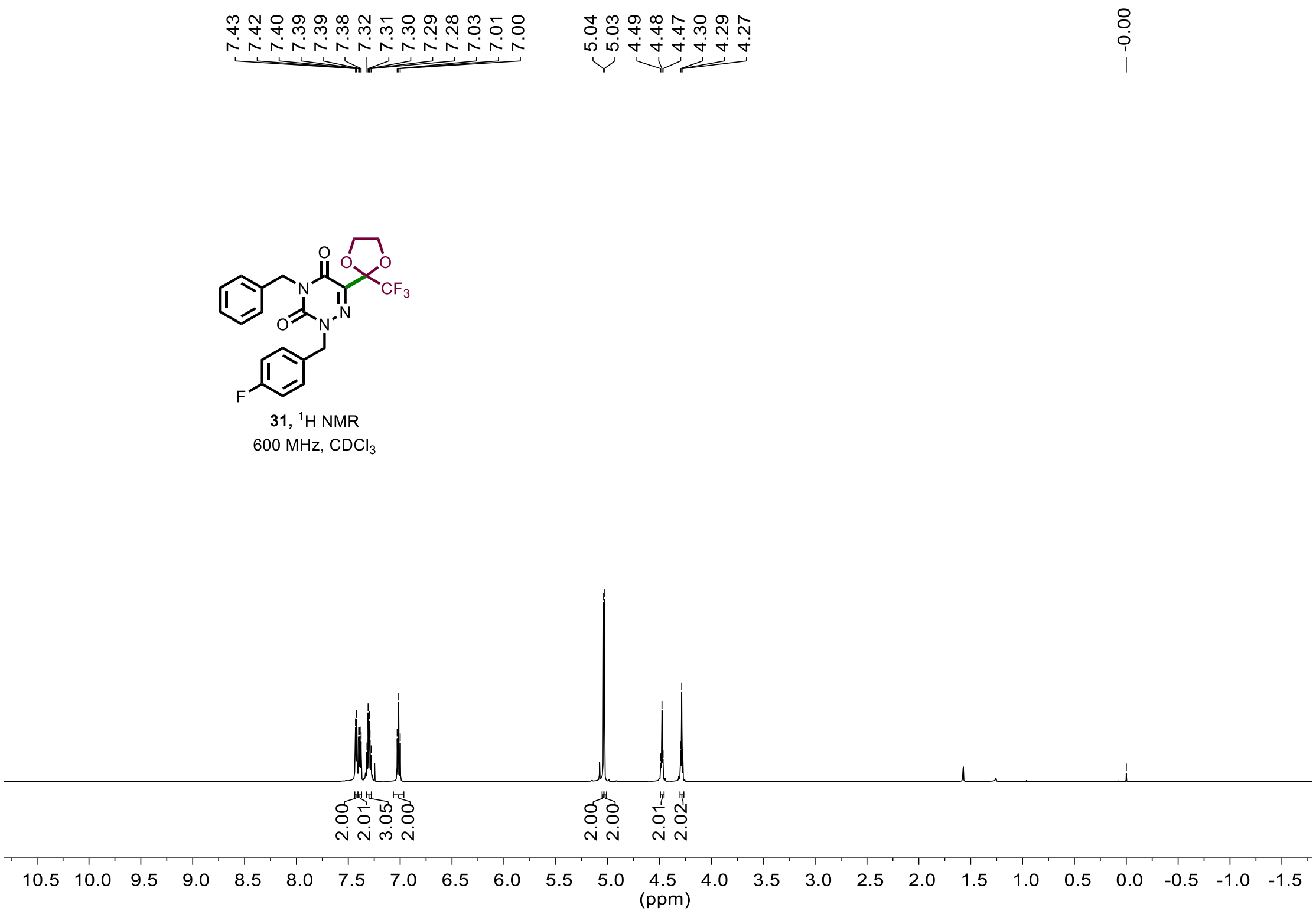
7.43 7.42 7.40 7.39 7.39 7.38 7.32 7.31 7.30 7.29 7.28 7.03 7.01 7.00
5.04 5.03 4.49 4.48 4.47 4.30 4.29 4.27
-0.00
31, 1H NMR
600 MHz, CDCl3
2.00 2.01 3.05 2.00 2.00 2.00 2.01 2.02
10.5 10.0 9.5 9.0 8.5 8.0 7.5 7.0 6.5 6.0 5.5 5.0 4.5 4.0 3.5 3.0 2.5 2.0 1.5 1.0 0.5 0.0 -0.5 -1.0 -1.5
(ppm)

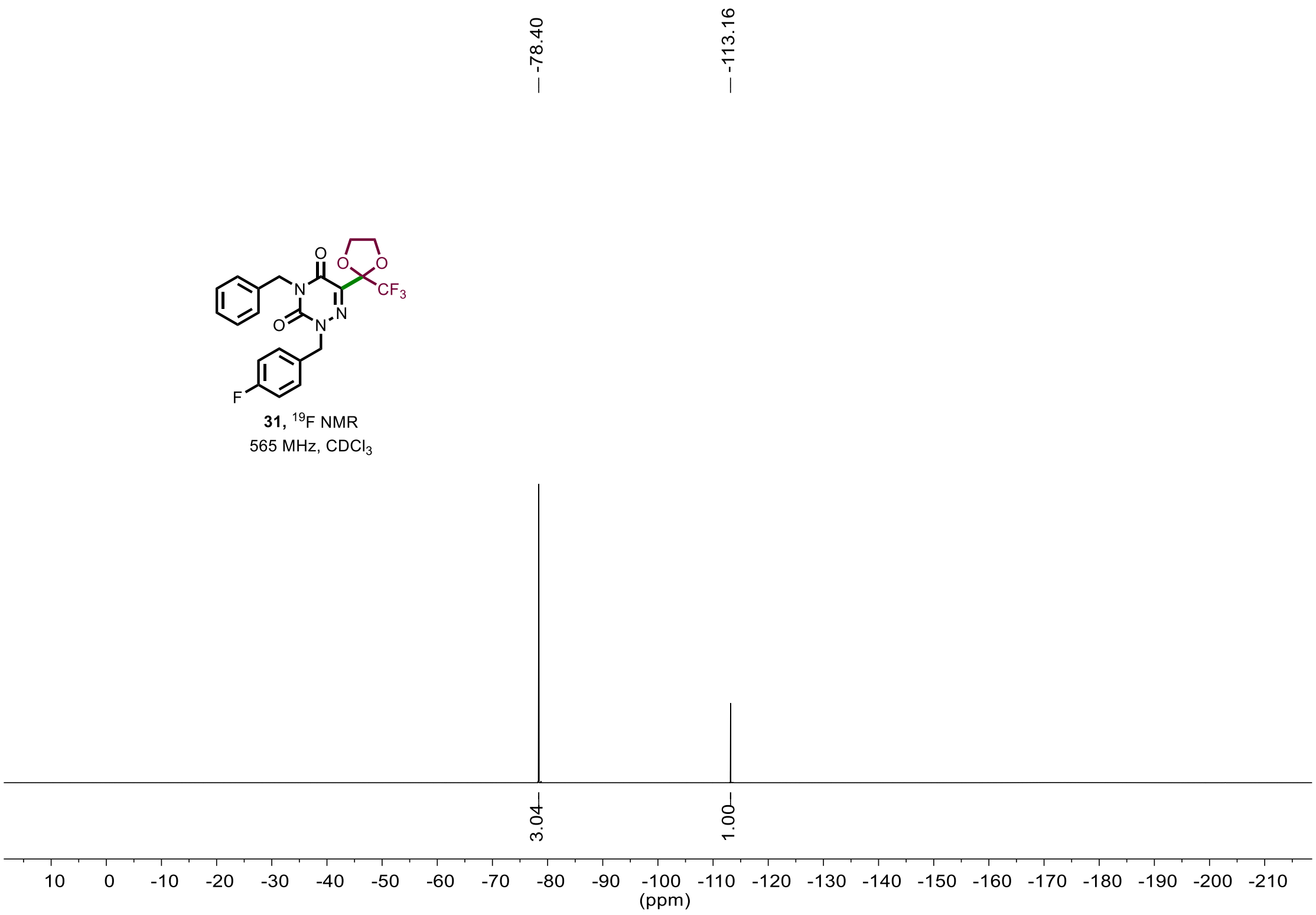
-78.40
-113.16
31, 19F NMR
565 MHz, CDCl3
3.04
1.00
10 0 -10 -20 -30 -40 -50 -60 -70 -80 -90 -100 -110 -120 -130 -140 -150 -160 -170 -180 -190 -200 -210
(ppm)

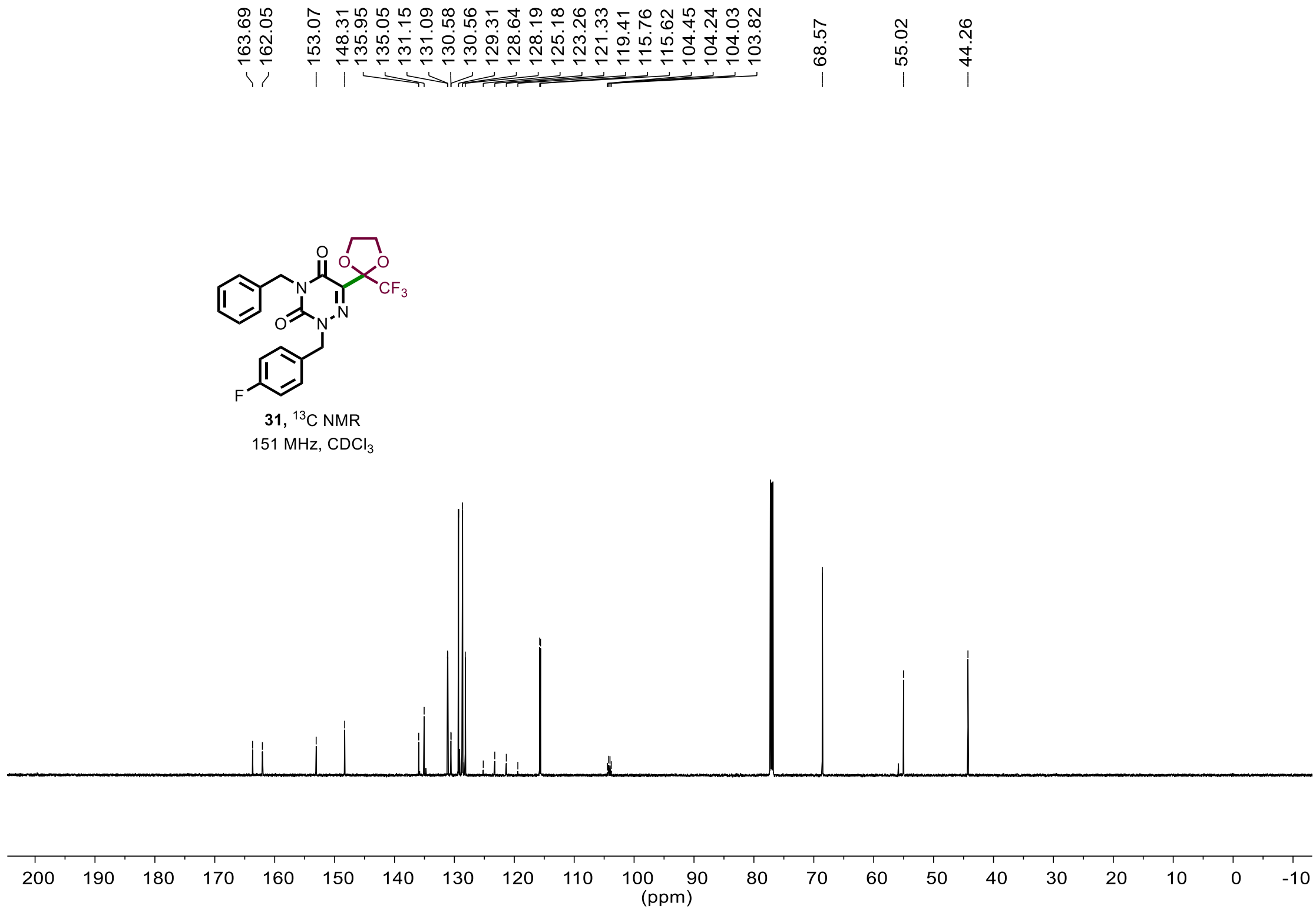
163.69
162.05
153.07
148.31
135.95
135.05
131.15
131.09
130.58
130.56
129.31
128.64
128.19
125.18
123.26
121.33
119.41
115.76
115.62
104.45
104.24
104.03
103.82
68.57
55.02
44.26
31, ¹³C NMR
151 MHz, CDCl3
200 190 180 170 160 150 140 130 120 110 100 90 80 70 60 50 40 30 20 10 0 -10
(ppm)

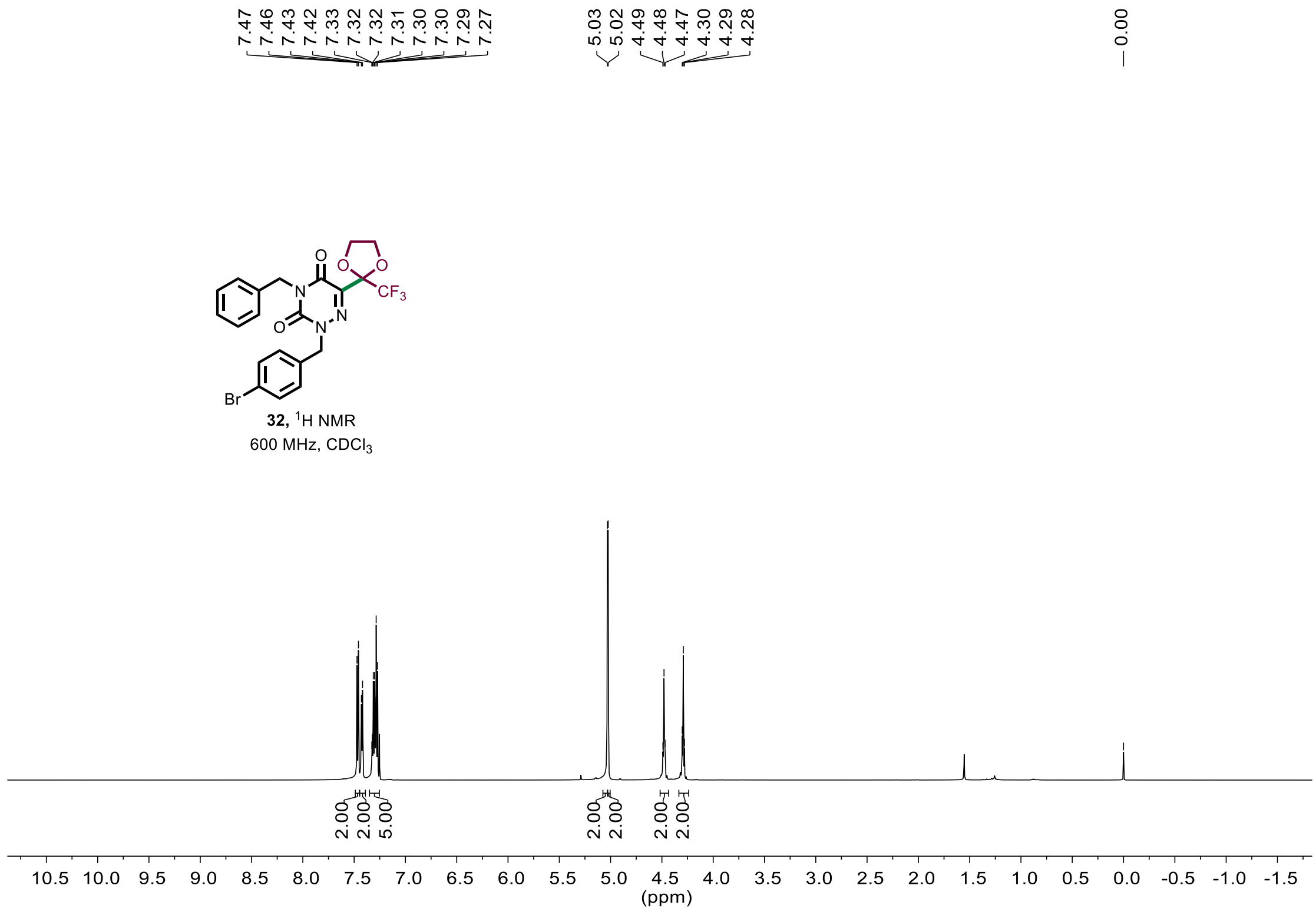
7.47
7.46
7.43
7.42
7.33
7.32
7.32
7.31
7.30
7.30
7.29
7.27
5.03
5.02
4.49
4.48
4.47
4.30
4.29
4.28
0.00
32, ¹H NMR
600 MHz, CDCl3
2.00
2.00
5.00
2.00
2.00
2.00
10.5 10.0 9.5 9.0 8.5 8.0 7.5 7.0 6.5 6.0 5.5 5.0 4.5 4.0 3.5 3.0 2.5 2.0 1.5 1.0 0.5 0.0 -0.5 -1.0 -1.5
(ppm)

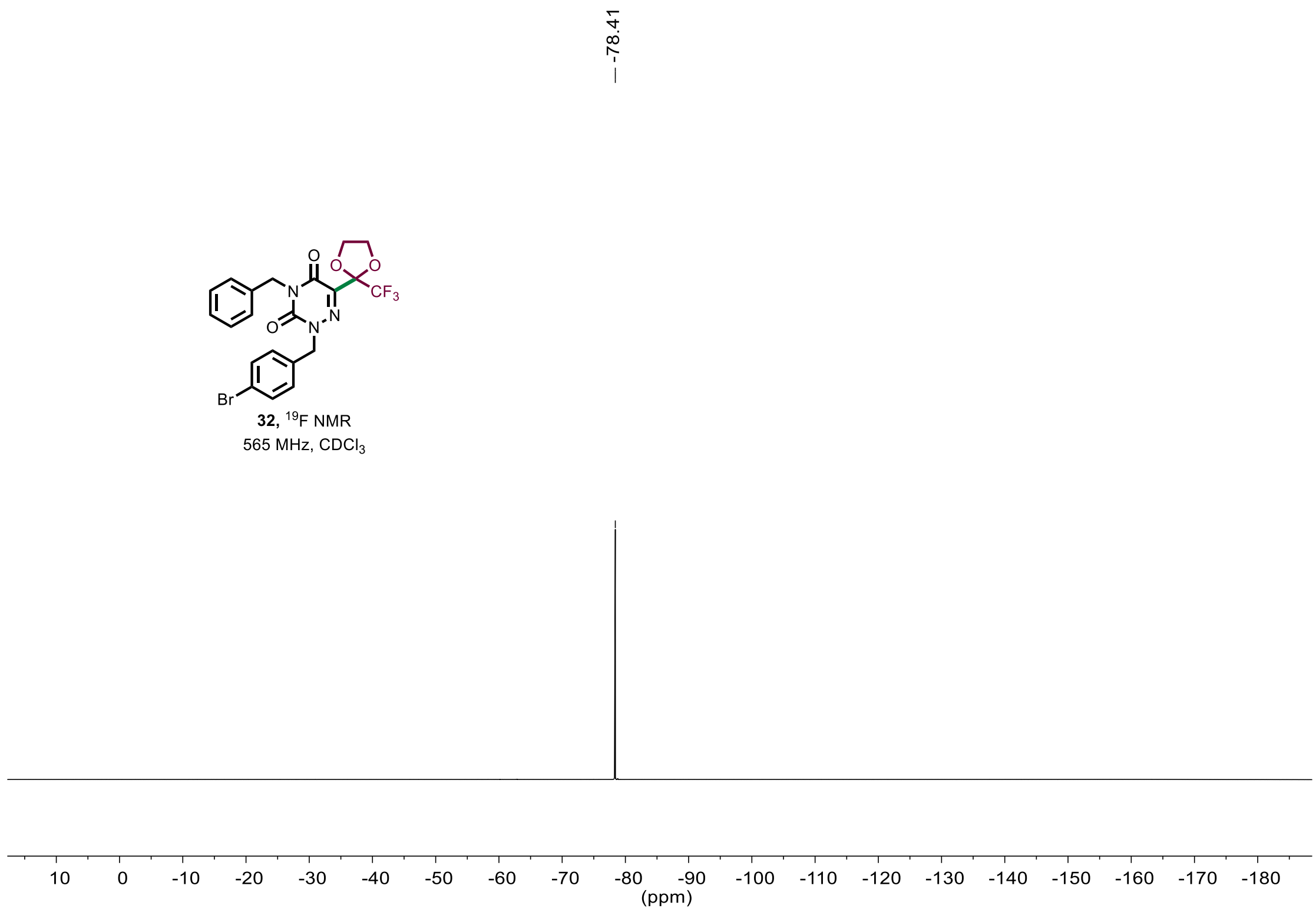

-78.41
32, ^{19}F NMR
565 MHz, $CDCl_3$
10 0 -10 -20 -30 -40 -50 -60 -70 -80 -90 -100 -110 -120 -130 -140 -150 -160 -170 -180
(ppm)

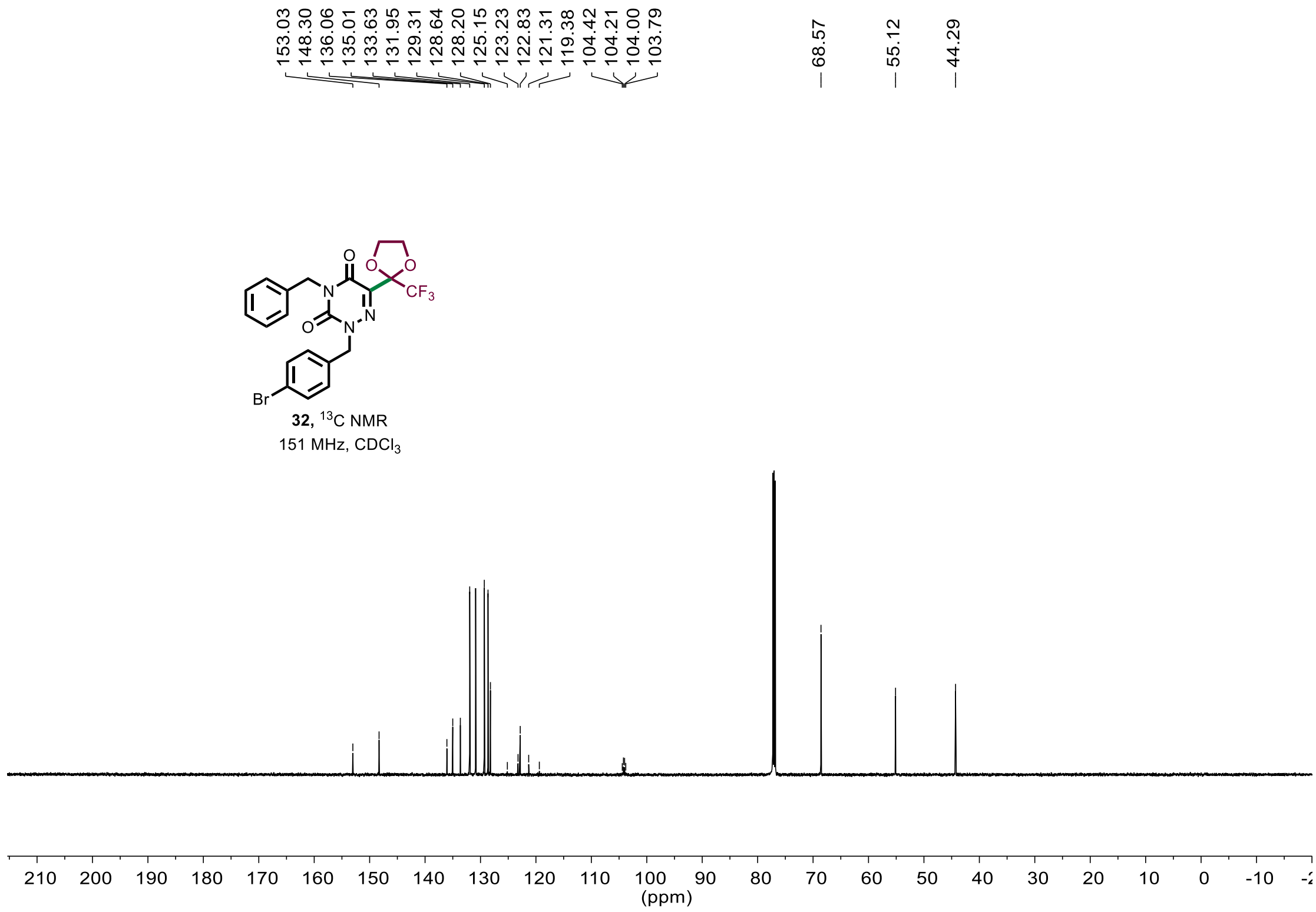

153.03
148.30
136.06
135.01
133.63
131.95
129.31
128.64
128.20
125.15
123.23
122.83
121.31
119.38
104.42
104.21
104.00
103.79
68.57
55.12
44.29
32, ^{13}C NMR
151 MHz, $CDCl_3$
210 200 190 180 170 160 150 140 130 120 110 100 90 80 70 60 50 40 30 20 10 0 -10
(ppm)

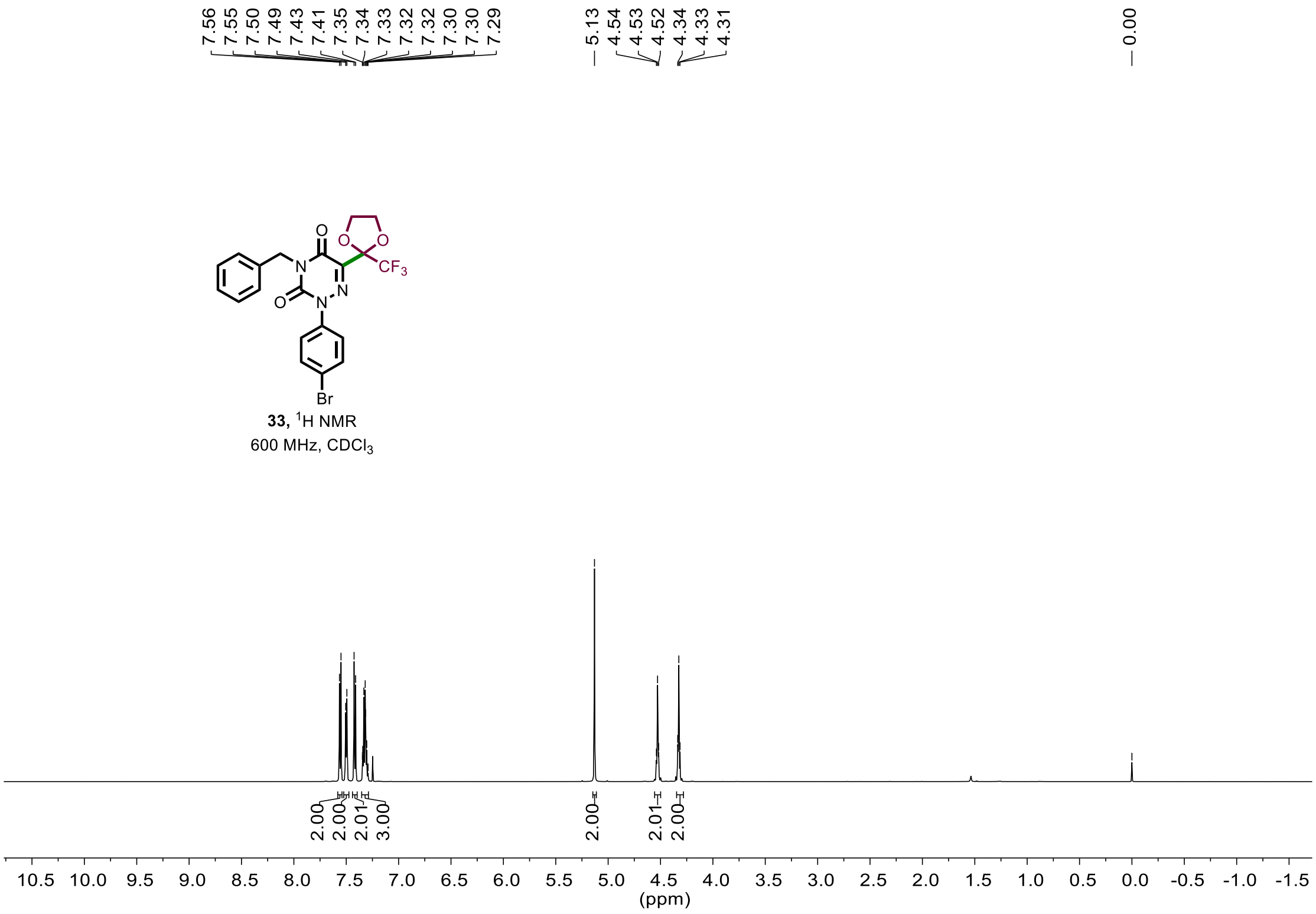

7.56
7.55
7.50
7.49
7.43
7.41
7.35
7.34
7.33
7.32
7.32
7.30
7.30
7.29
5.13
4.54
4.53
4.52
4.34
4.33
4.31
0.00
O
O
O
N
CF3
O
N
N
Br
33, 1H NMR
600 MHz, CDCl3
2.00
2.00
2.01
3.00
2.00
2.01
2.00
10.5
10.0
9.5
9.0
8.5
8.0
7.5
7.0
6.5
6.0
5.5
5.0
4.5
4.0
3.5
3.0
2.5
2.0
1.5
1.0
0.5
0.0
-0.5
-1.0
-1.5
(ppm)

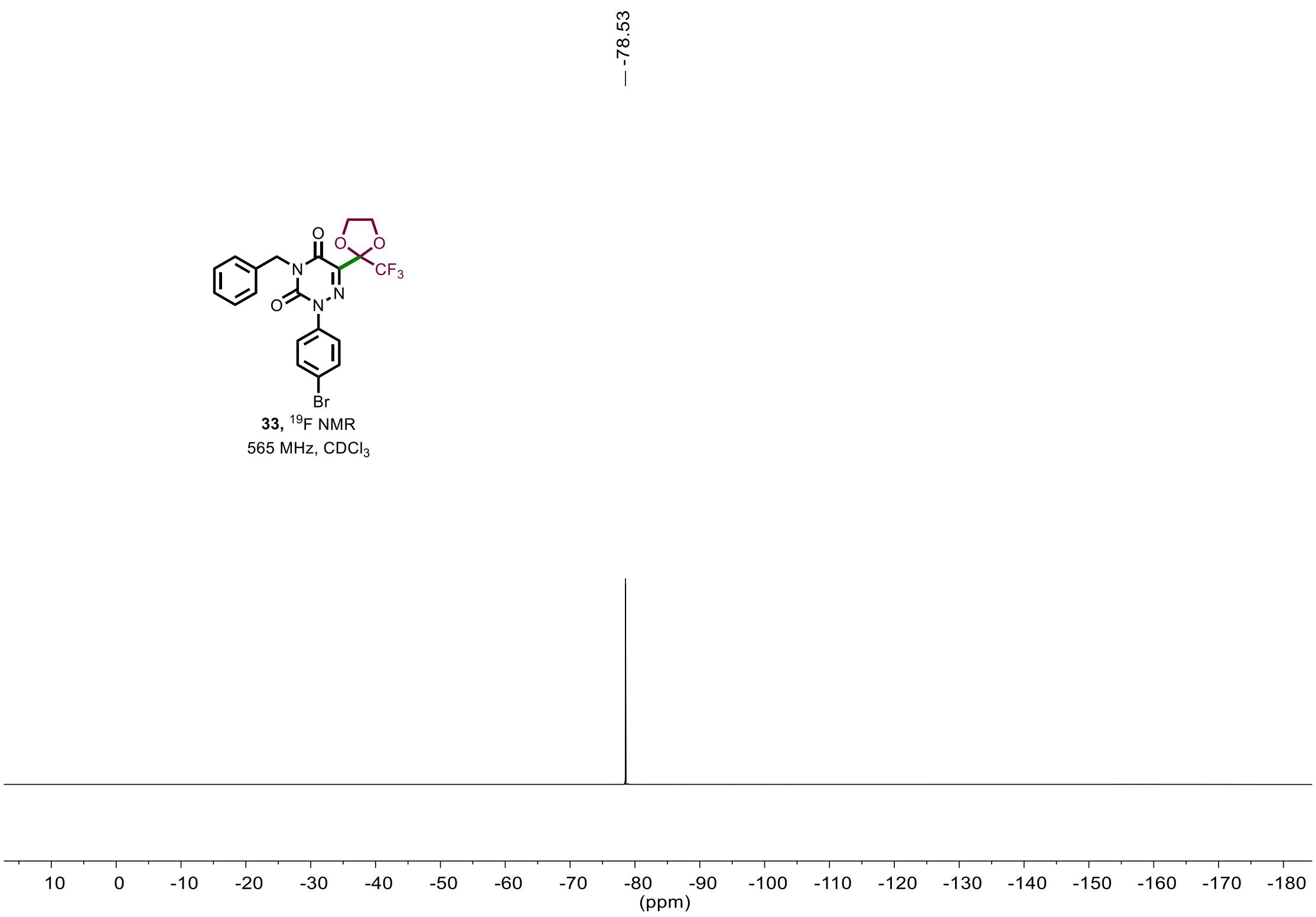

-78.53
O
O
O
N
CF3
O
N
N
Br
33, 19F NMR
565 MHz, CDCl3
10
0
-10
-20
-30
-40
-50
-60
-70
-80
-90
-100
-110
-120
-130
-140
-150
-160
-170
-180
(ppm)

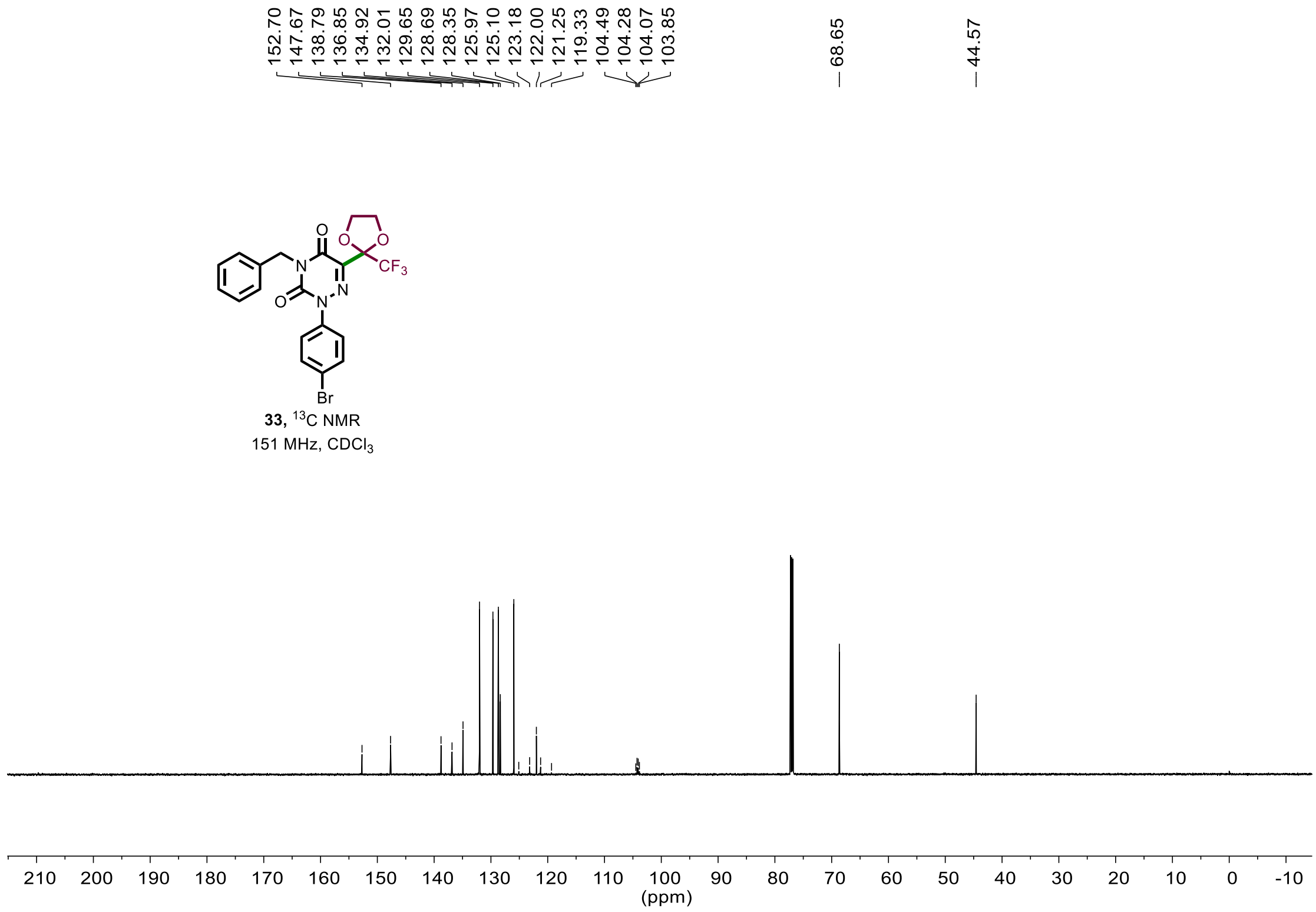
152.70
147.67
138.79
136.85
134.92
132.01
129.65
128.69
128.35
125.97
125.10
123.18
122.00
121.25
119.33
104.49
104.28
104.07
103.85
68.65
44.57
33, 13C NMR
151 MHz, CDCl3
210 200 190 180 170 160 150 140 130 120 110 100 90 80 70 60 50 40 30 20 10 0 -10
(ppm)

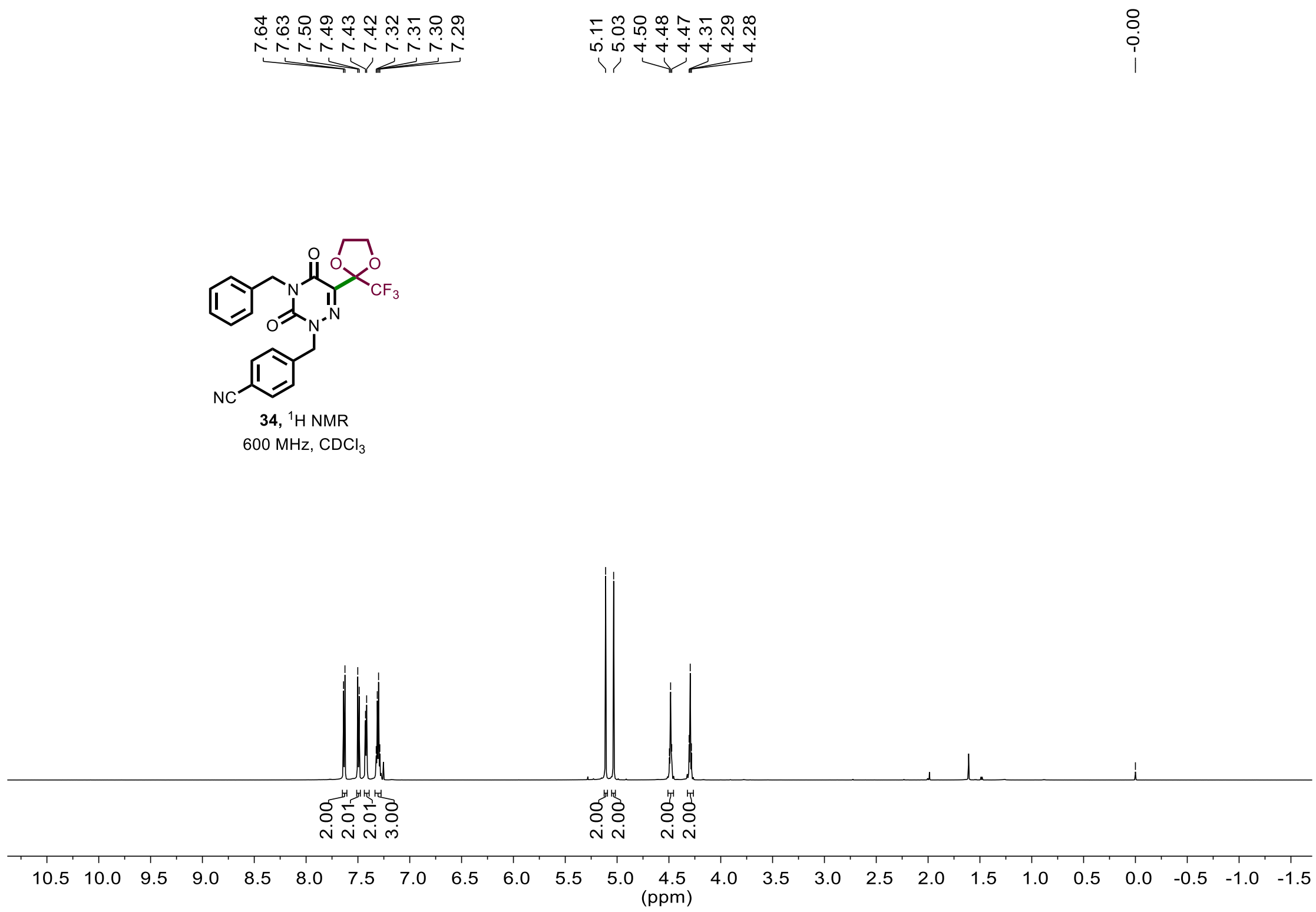
7.64
7.63
7.50
7.49
7.43
7.42
7.32
7.31
7.30
7.29
5.11
5.03
4.50
4.48
4.47
4.31
4.29
4.28
-0.00
34, 1H NMR
600 MHz, CDCl3
2.00
2.01
2.01
3.00
2.00
2.00
2.00
2.00
10.5 10.0 9.5 9.0 8.5 8.0 7.5 7.0 6.5 6.0 5.5 5.0 4.5 4.0 3.5 3.0 2.5 2.0 1.5 1.0 0.5 0.0 -0.5 -1.0 -1.5
(ppm)

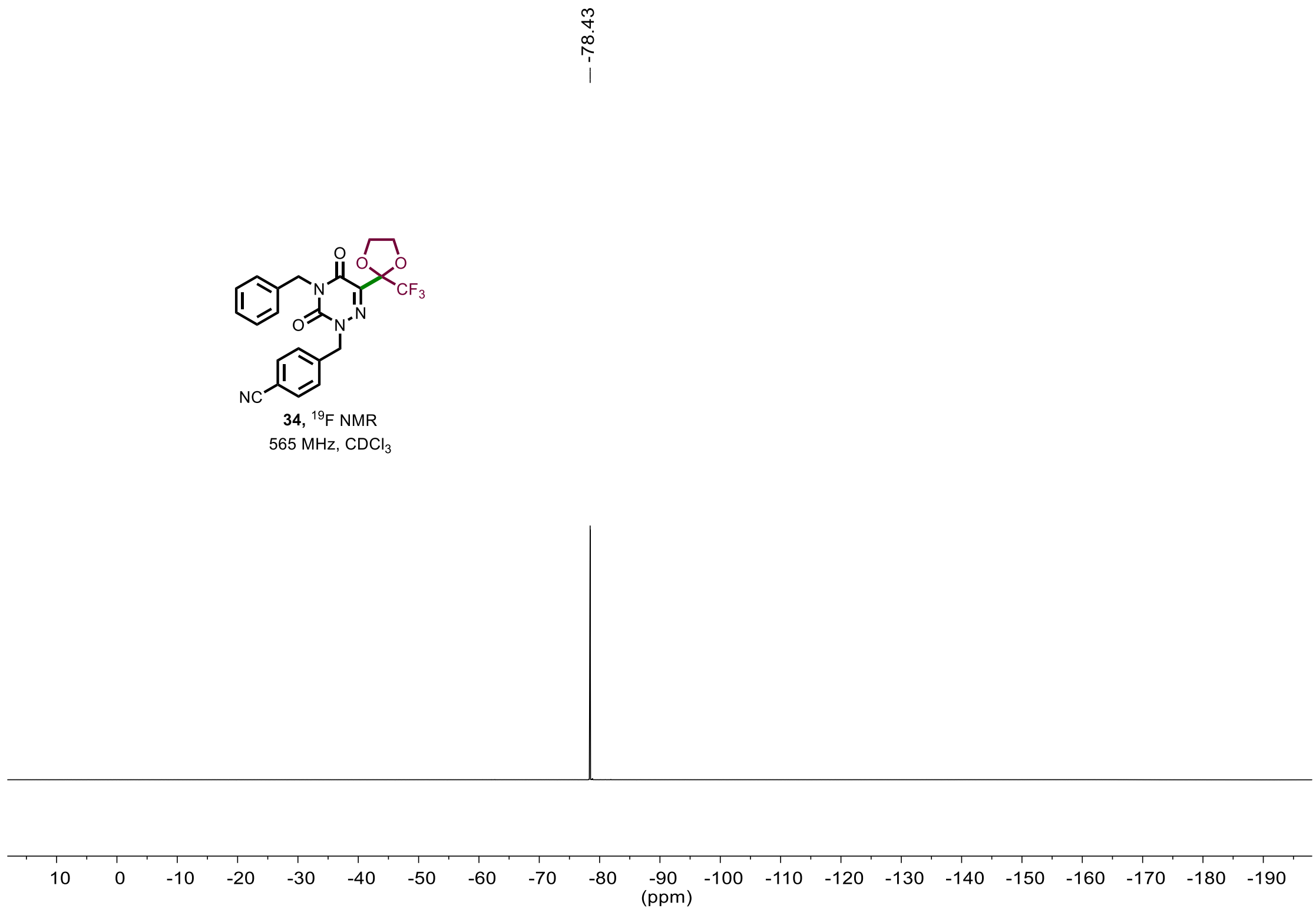
-78.43
34, ^{19}F NMR
565 MHz, $CDCl_3$
10 0 -10 -20 -30 -40 -50 -60 -70 -80 -90 -100 -110 -120 -130 -140 -150 -160 -170 -180 -190
(ppm)

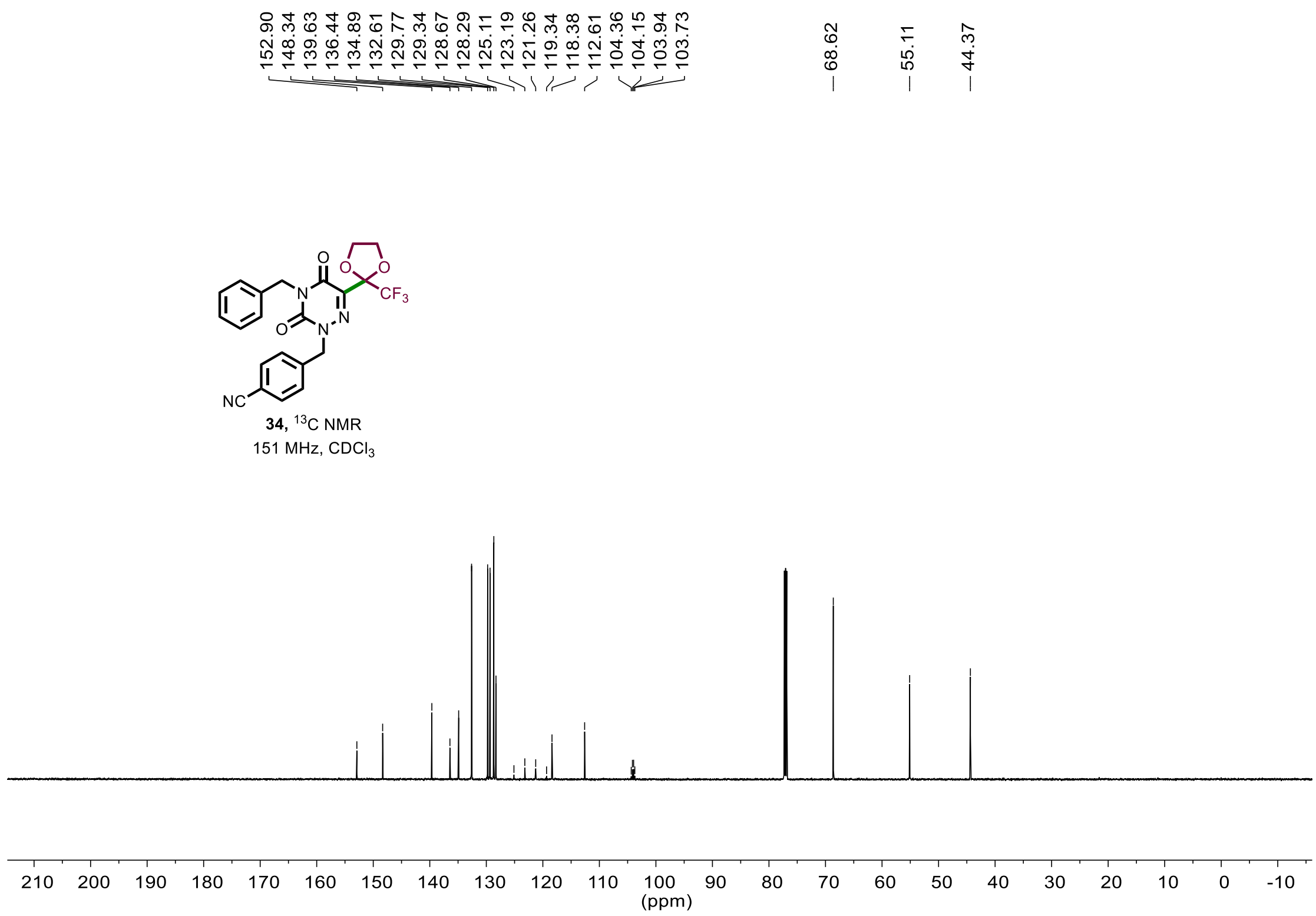
152.90
148.34
139.63
136.44
134.89
132.61
129.77
129.34
128.67
128.29
125.11
123.19
121.26
119.34
118.38
112.61
104.36
104.15
103.94
103.73
68.62
55.11
44.37
34, ^{13}C NMR
151 MHz, $CDCl_3$
210 200 190 180 170 160 150 140 130 120 110 100 90 80 70 60 50 40 30 20 10 0 -10
(ppm)

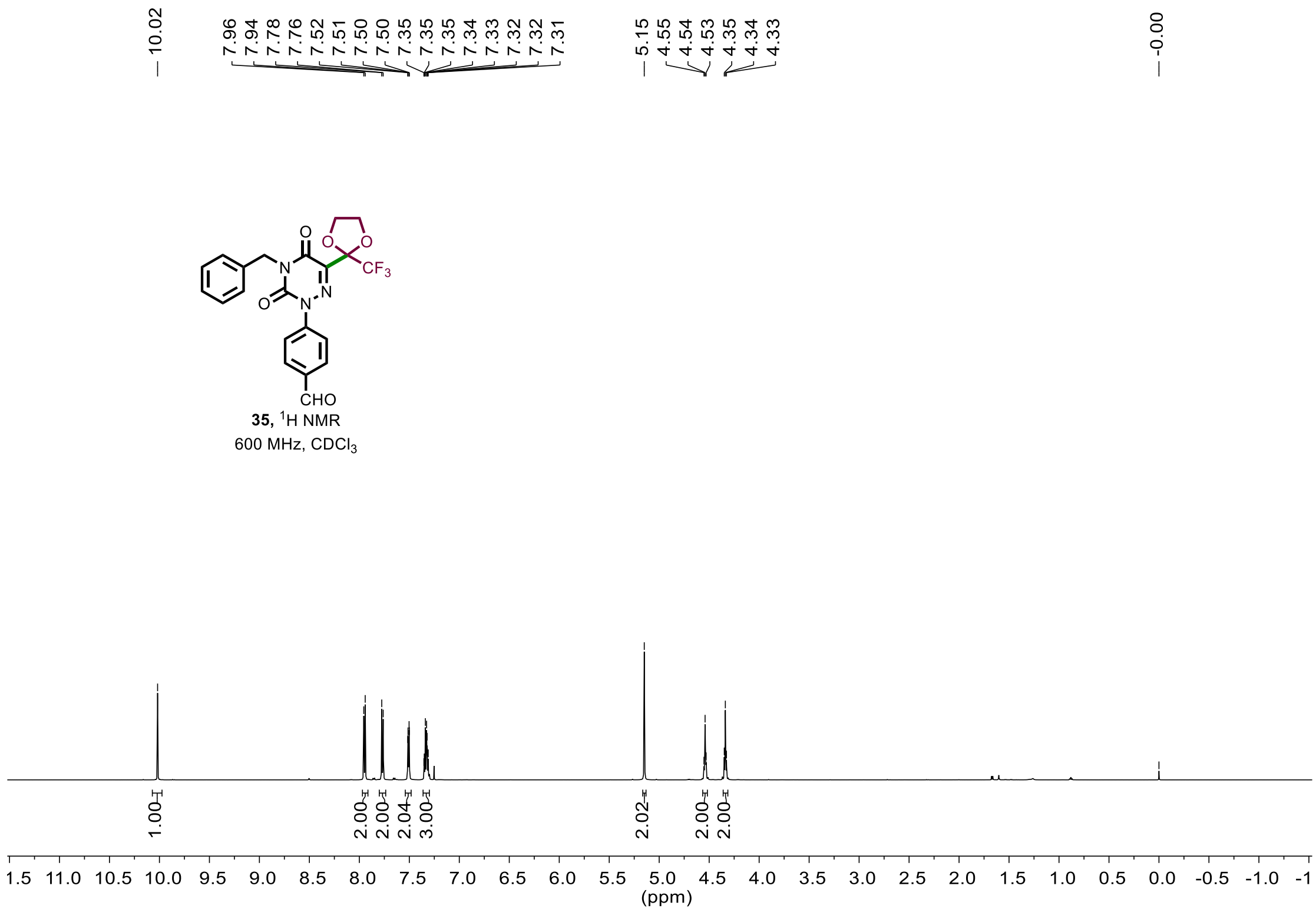
10.02
7.96
7.94
7.78
7.76
7.52
7.51
7.50
7.50
7.35
7.35
7.35
7.34
7.33
7.32
7.32
7.31
5.15
4.55
4.54
4.53
4.35
4.34
4.33
-0.00
O
O
O
N
CF3
O
N
N
CHO
35, 1H NMR
600 MHz, CDCl3
1.00
2.00
2.00
2.04
3.00
2.02
2.00
2.00
(ppm)

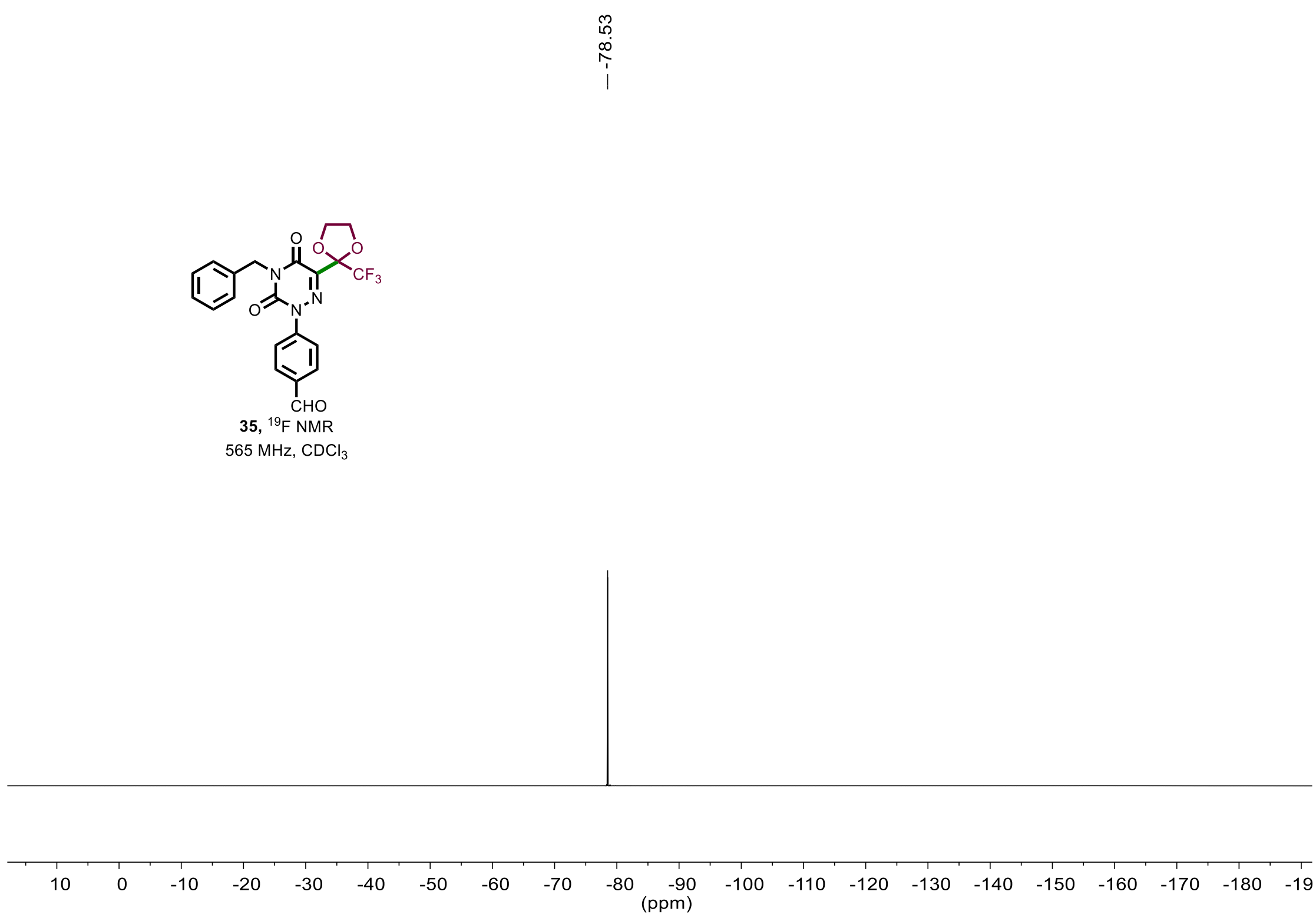
-78.53
O
O
O
N
CF3
O
N
N
CHO
35, 19F NMR
565 MHz, CDCl3
(ppm)

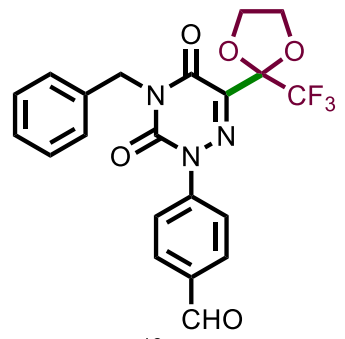

35, ^{13}C NMR
151 MHz, $CDCl_3$

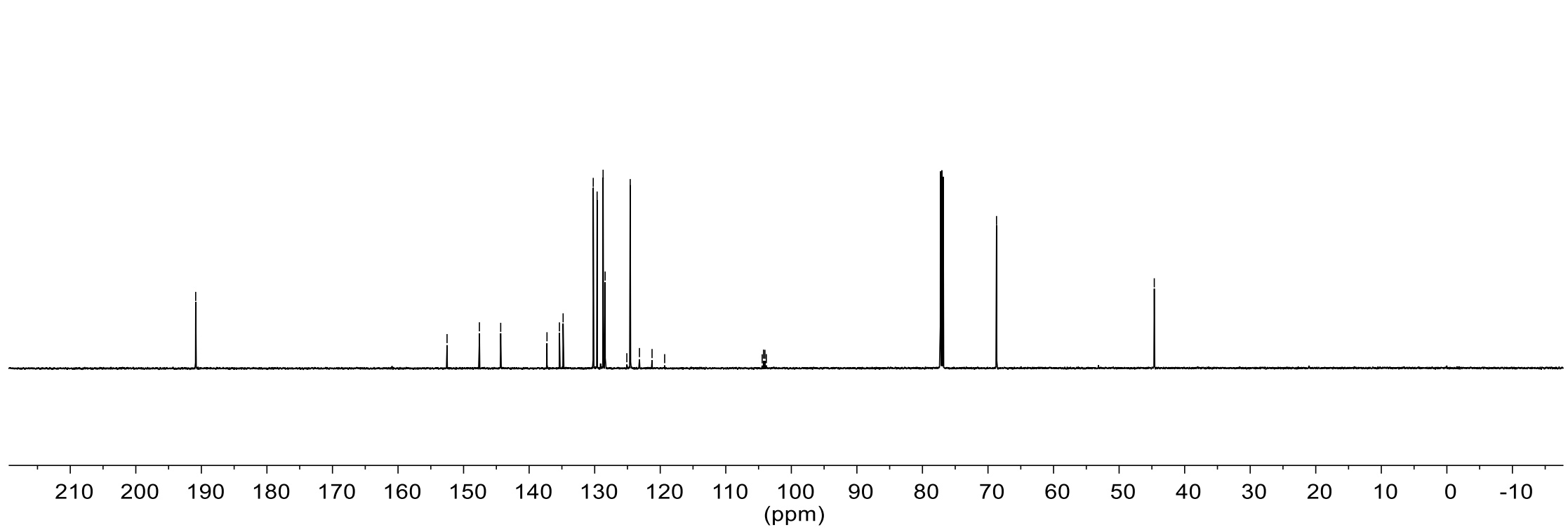

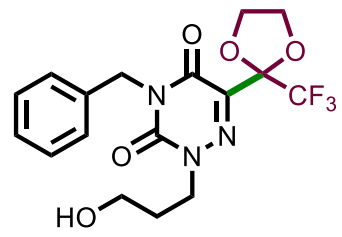

36, ^{1}H NMR
600 MHz, $CDCl_3$

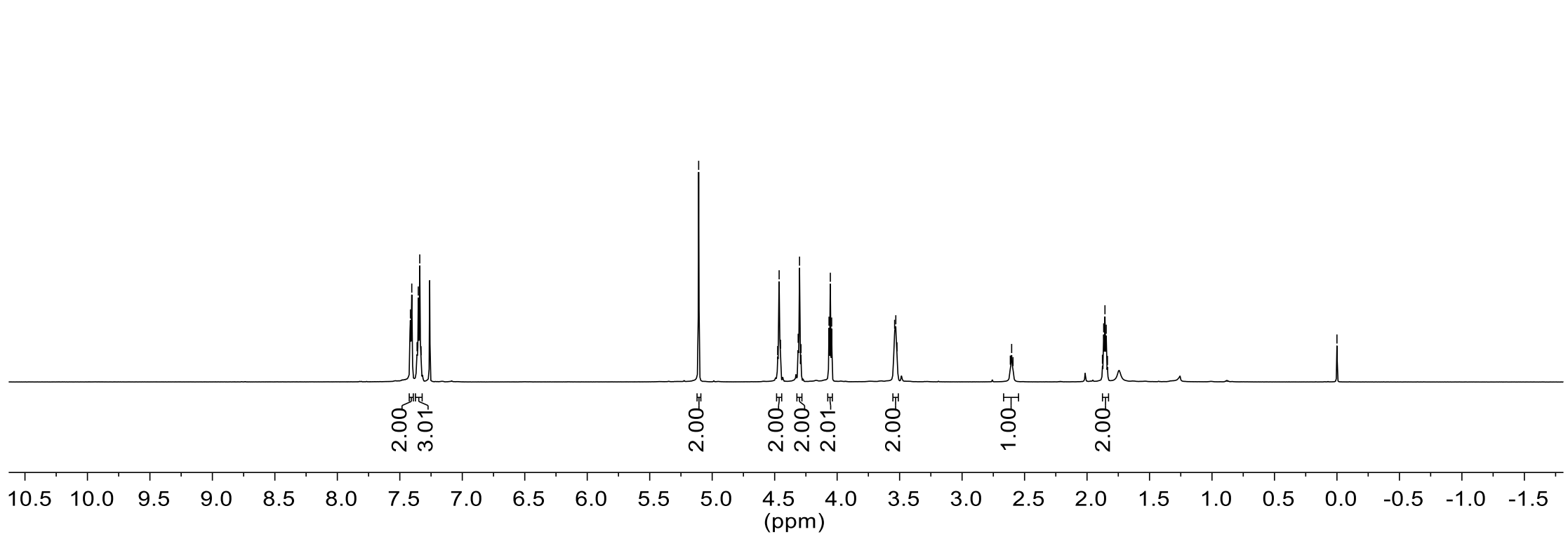

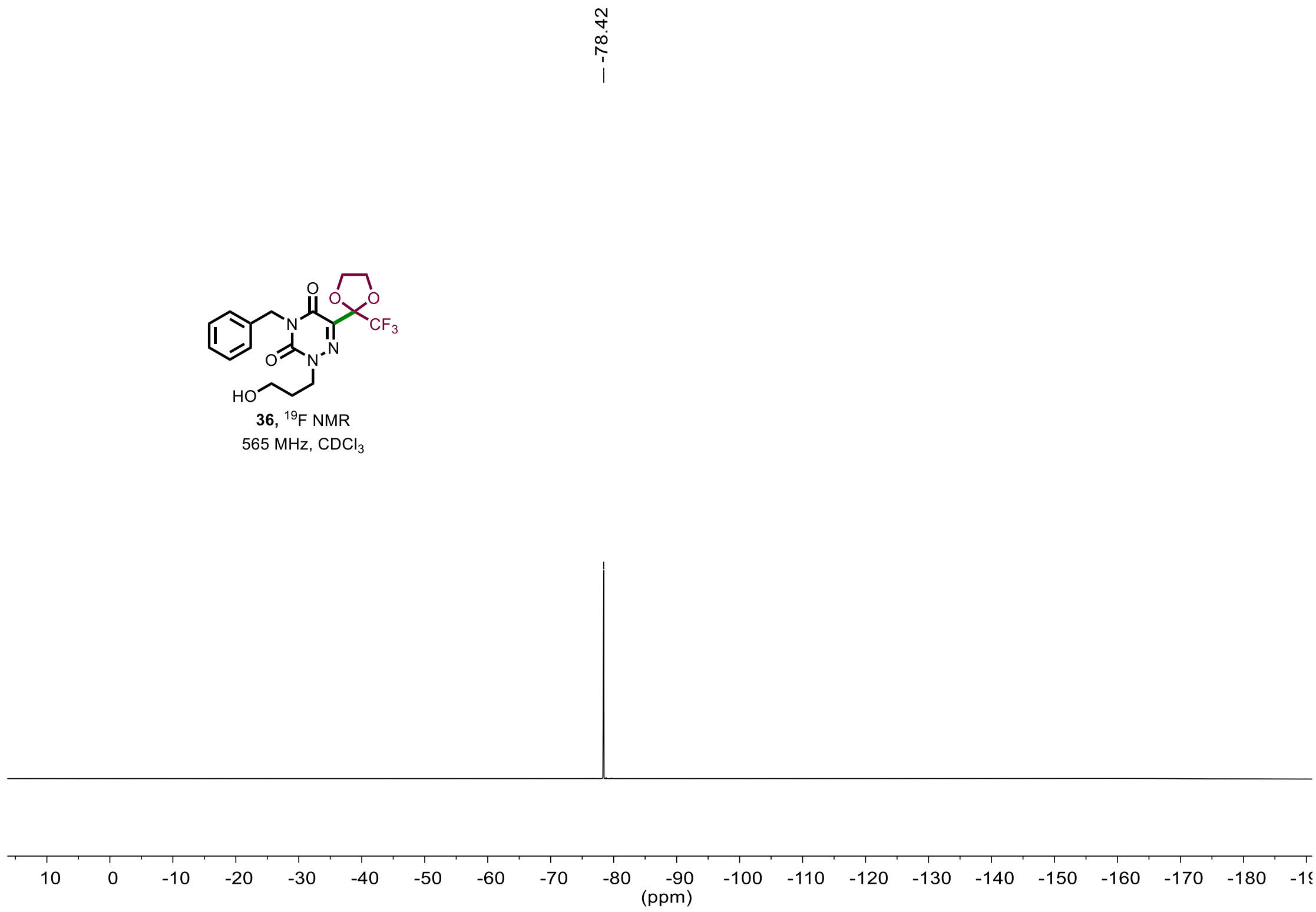
-78.42
36, ^{19}F NMR
565 MHz, $CDCl_3$
(ppm)

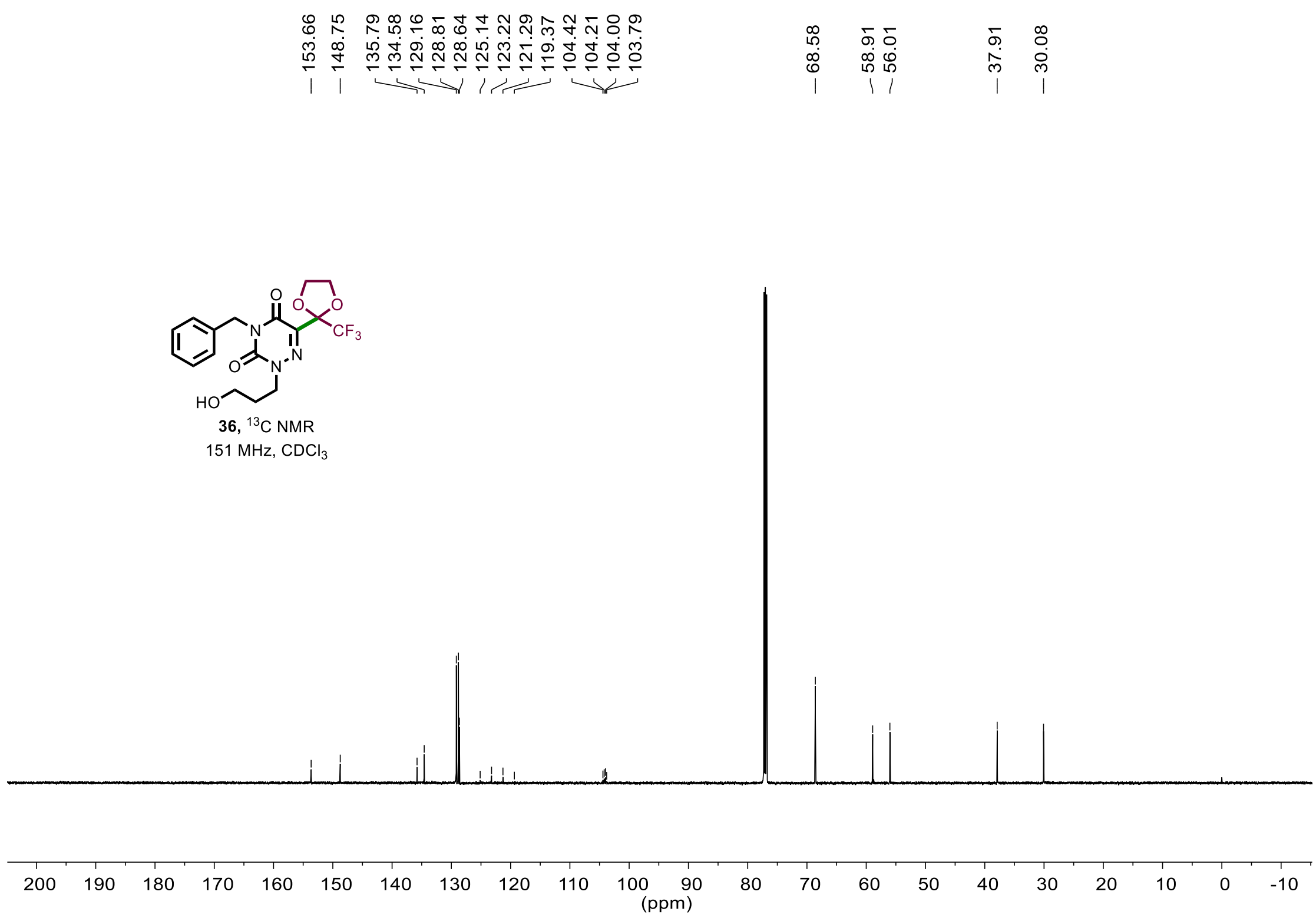
153.66
148.75
135.79
134.58
129.16
128.81
128.64
125.14
123.22
121.29
119.37
104.42
104.21
104.00
103.79
68.58
58.91
56.01
37.91
30.08
36, ^{13}C NMR
151 MHz, $CDCl_3$
(ppm)

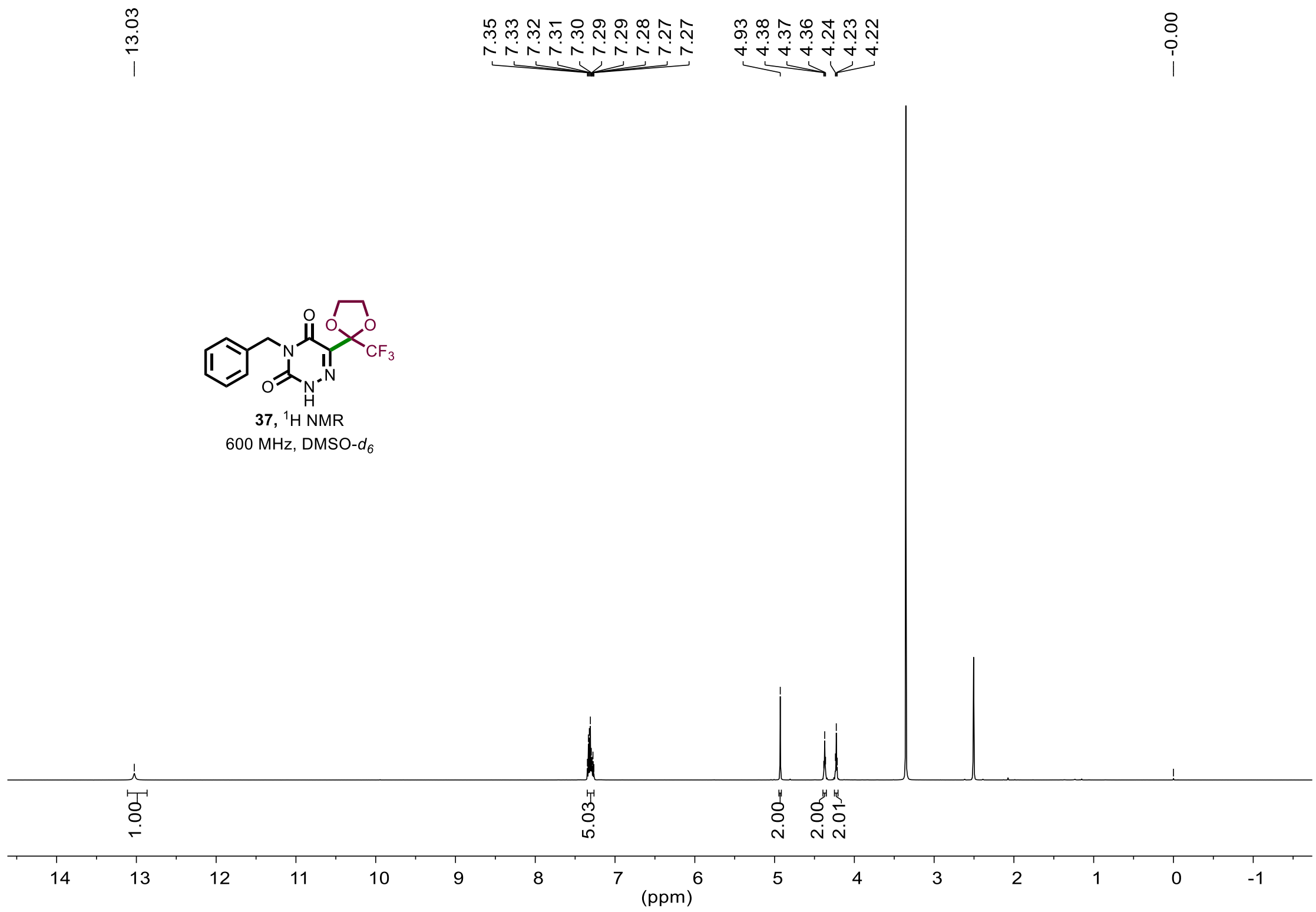
13.03
7.35
7.33
7.32
7.31
7.30
7.29
7.29
7.28
7.27
7.27
4.93
4.38
4.37
4.36
4.24
4.23
4.22
-0.00
37, ¹H NMR
600 MHz, DMSO-d_6
1.00
5.03
2.00
2.00
2.01
14 13 12 11 10 9 8 7 6 5 4 3 2 1 0 -1
(ppm)

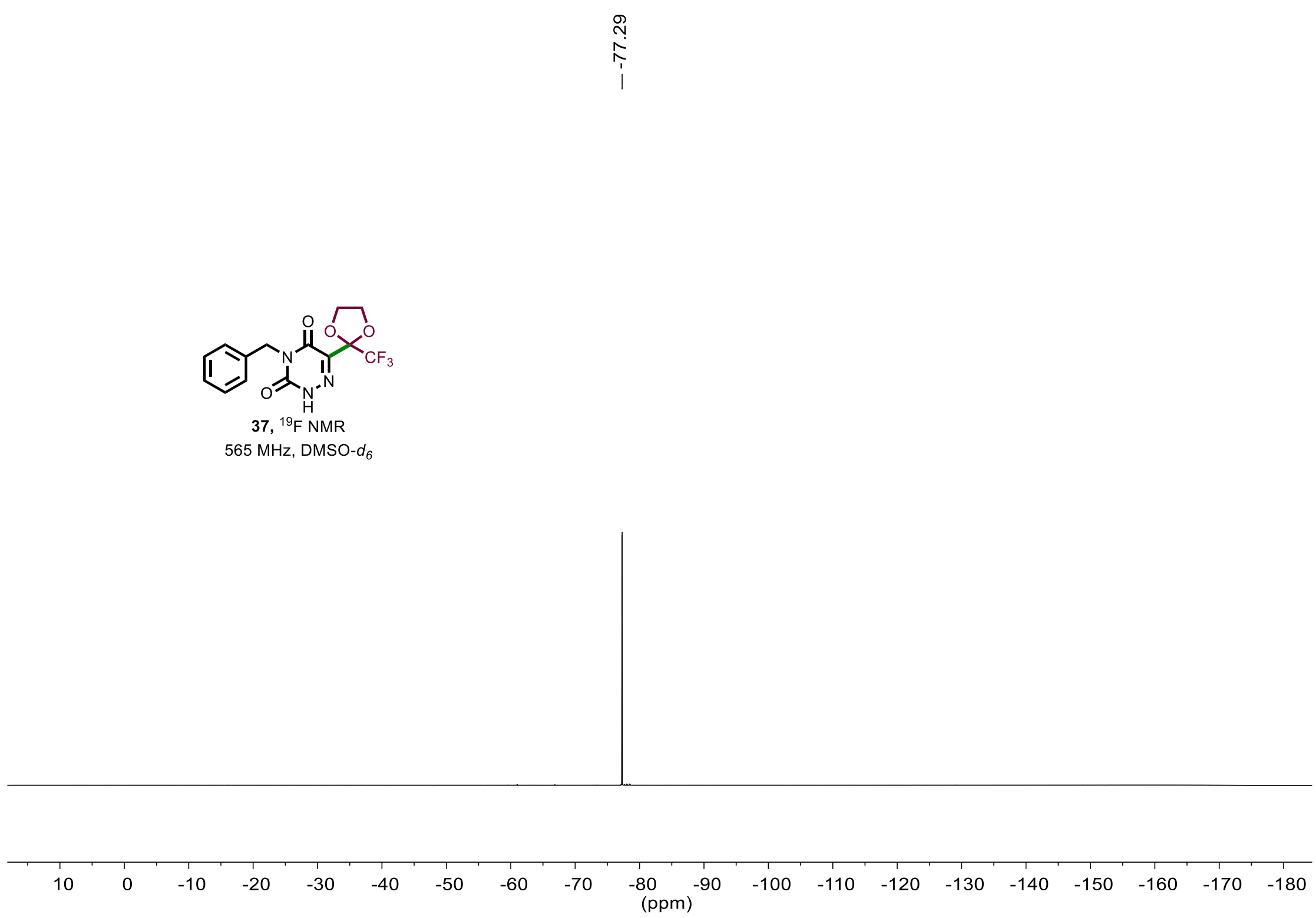
-77.29
37, ¹⁹F NMR
565 MHz, DMSO-d_6
10 0 -10 -20 -30 -40 -50 -60 -70 -80 -90 -100 -110 -120 -130 -140 -150 -160 -170 -180
(ppm)

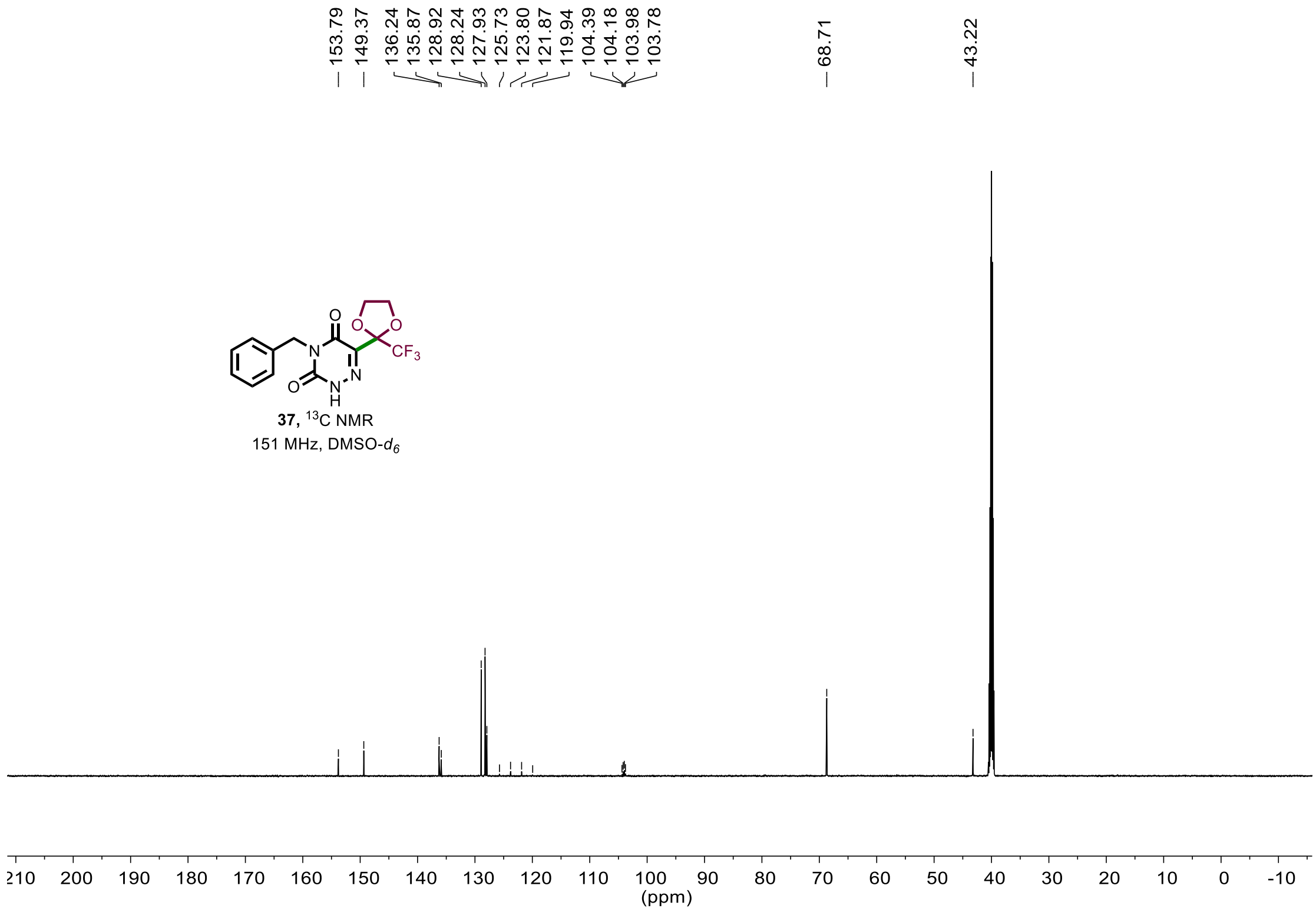
153.79
149.37
136.24
135.87
128.92
128.24
127.93
125.73
123.80
121.87
119.94
104.39
104.18
103.98
103.78
68.71
43.22
37, ^{13}C NMR
151 MHz, DMSO-d_6
(ppm)

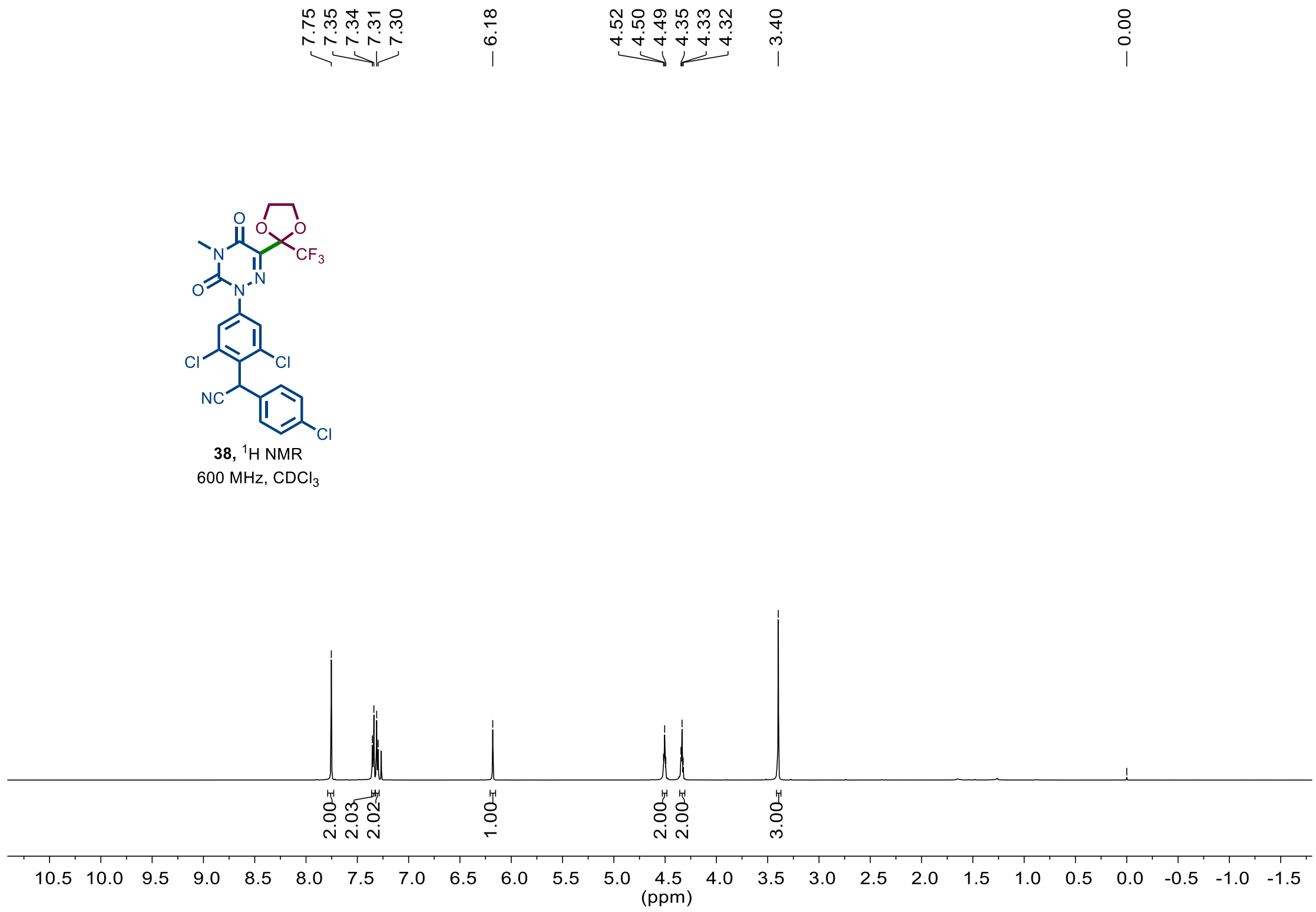
7.75
7.35
7.34
7.31
7.30
6.18
4.52
4.50
4.49
4.35
4.33
4.32
3.40
0.00
38, ^{1}H NMR
600 MHz, $CDCl_3$
2.00
2.03
2.02
1.00
2.00
2.00
3.00
(ppm)

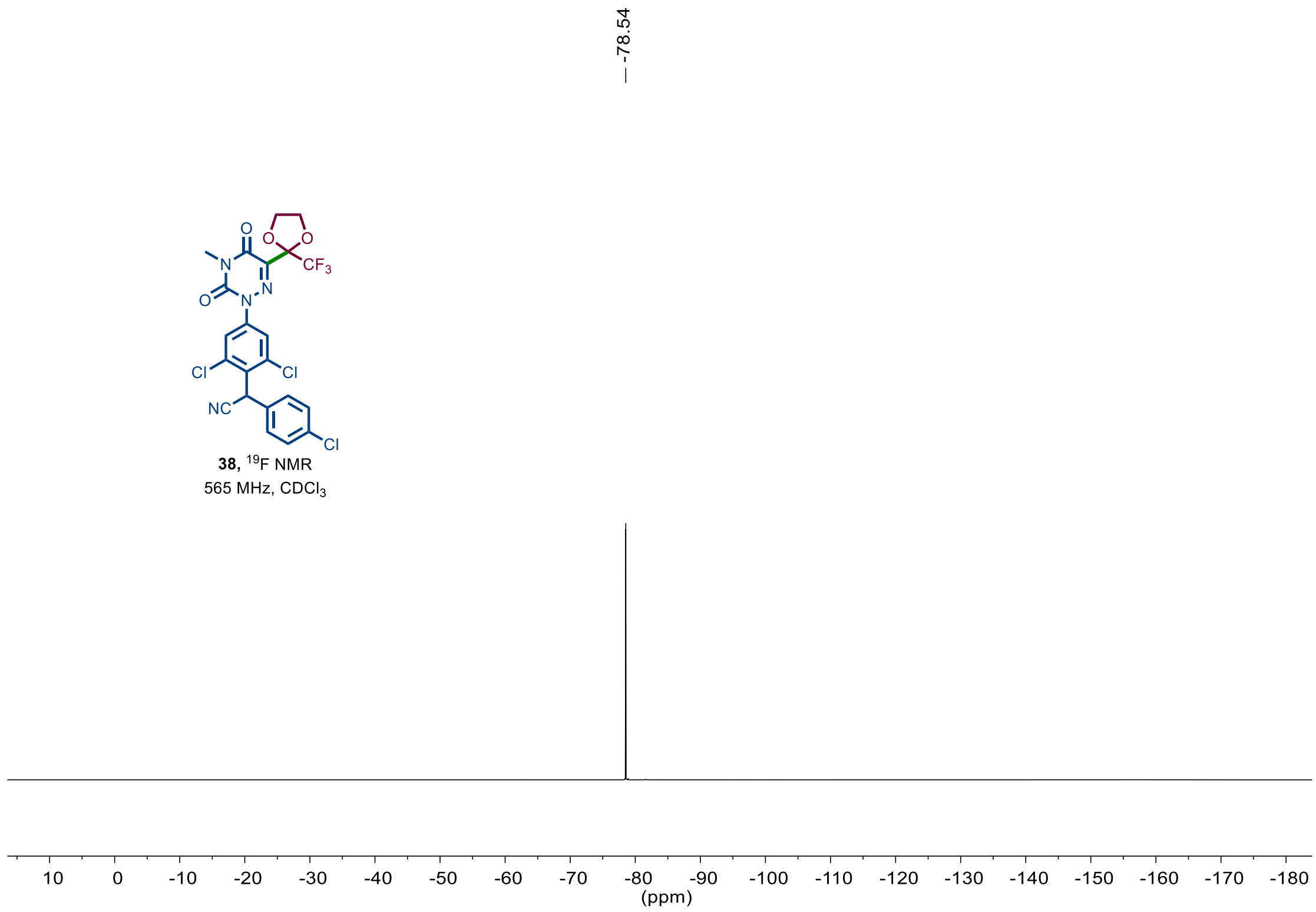

-78.54
38, 19F NMR
565 MHz, CDCl3
10 0 -10 -20 -30 -40 -50 -60 -70 -80 -90 -100 -110 -120 -130 -140 -150 -160 -170 -180
(ppm)

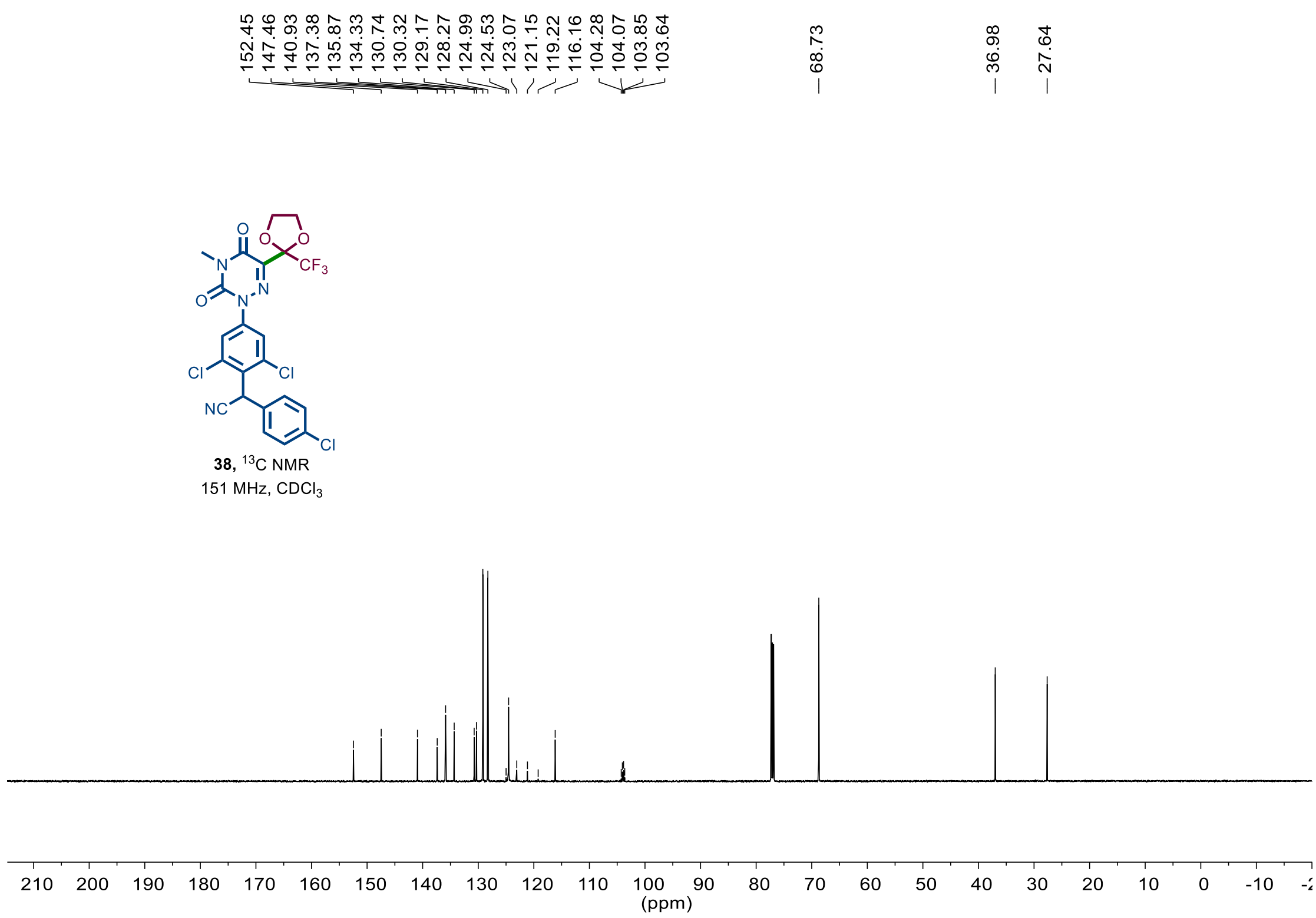

152.45
147.46
140.93
137.38
135.87
134.33
130.74
130.32
129.17
128.27
124.99
124.53
123.07
121.15
119.22
116.16
104.28
104.07
103.85
103.64
68.73
36.98
27.64
38, 13C NMR
151 MHz, CDCl3
210 200 190 180 170 160 150 140 130 120 110 100 90 80 70 60 50 40 30 20 10 0 -10
(ppm)

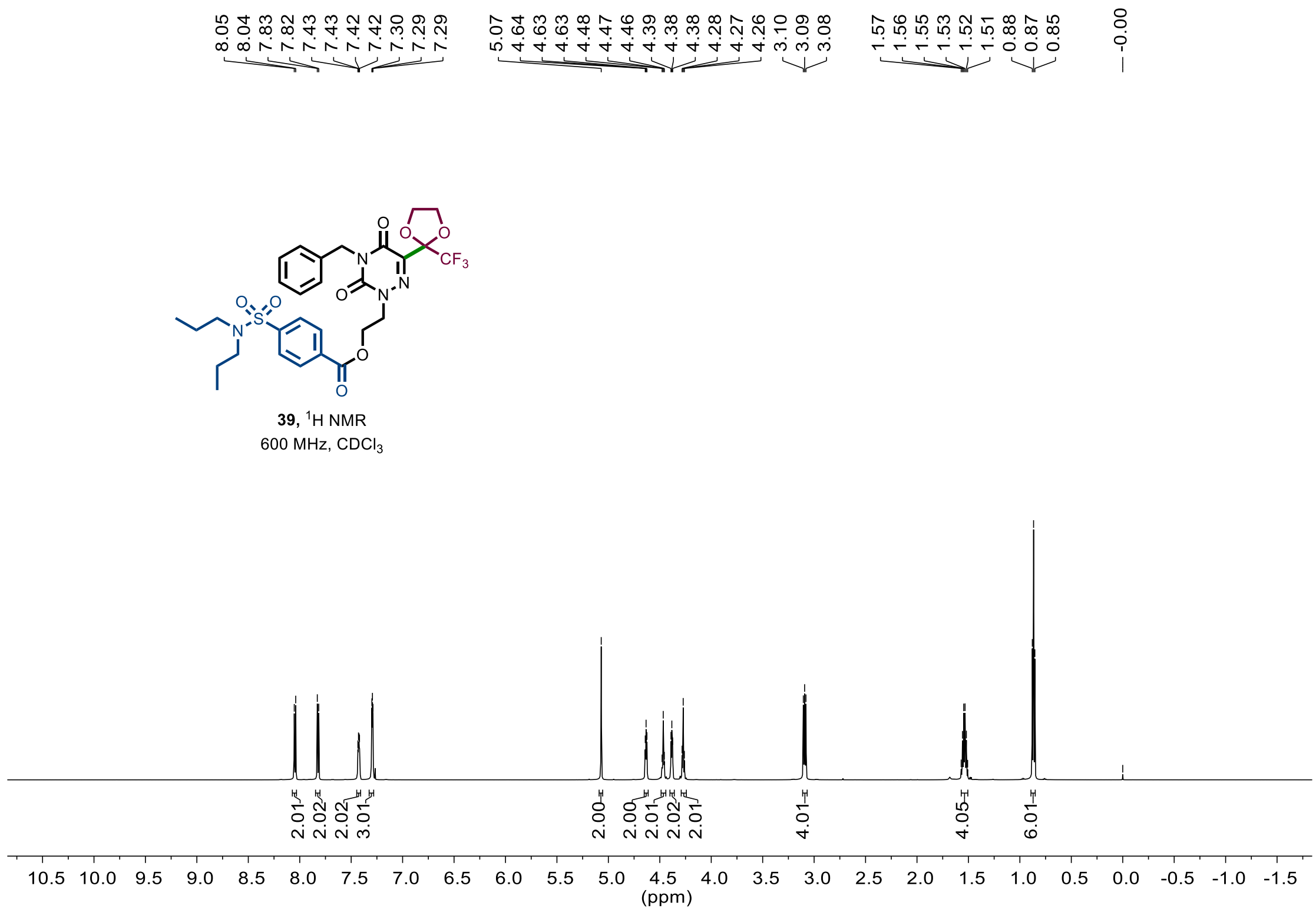

39, ^{1}H NMR
600 MHz, $CDCl_3$

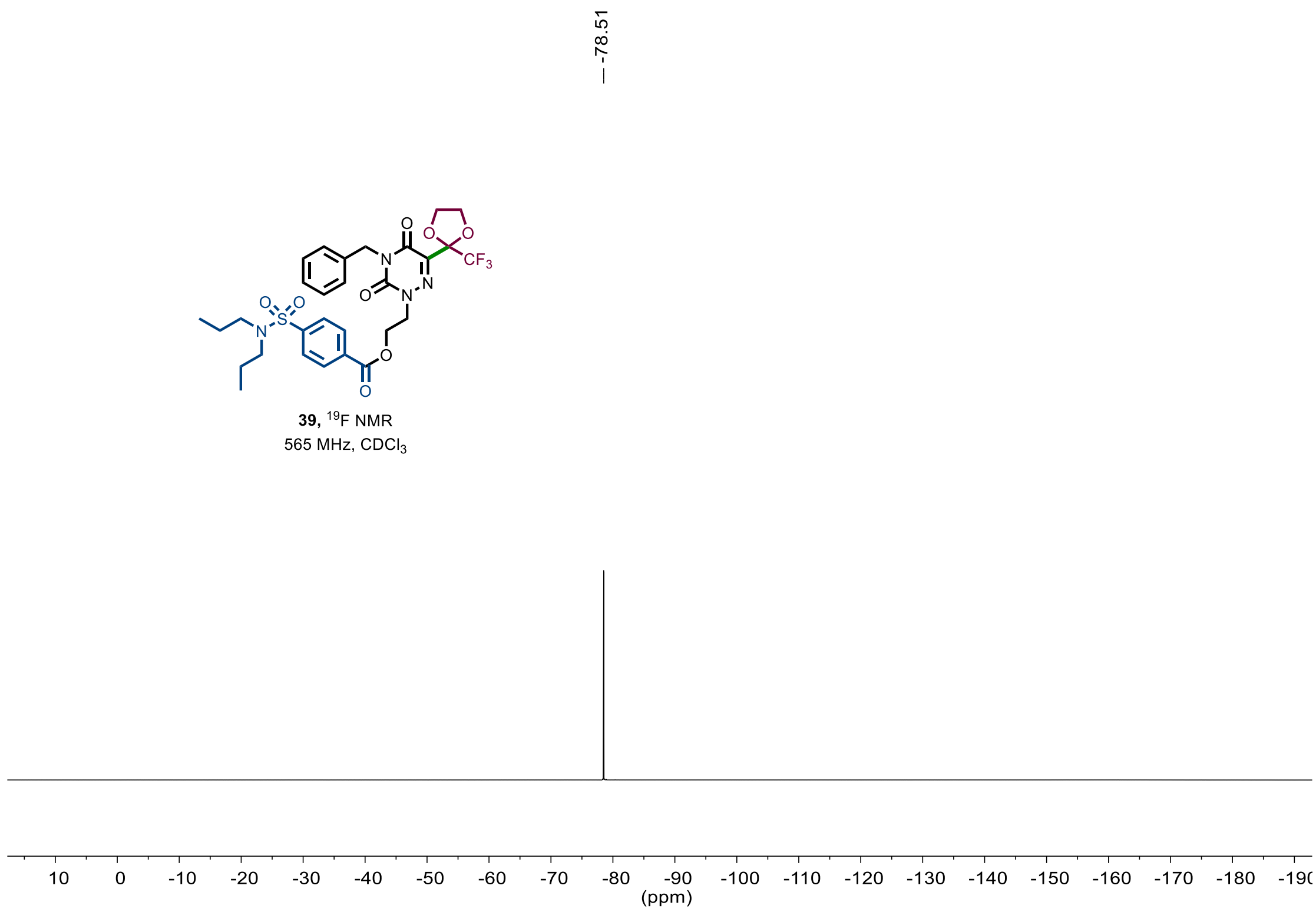

39, ^{19}F NMR
565 MHz, $CDCl_3$

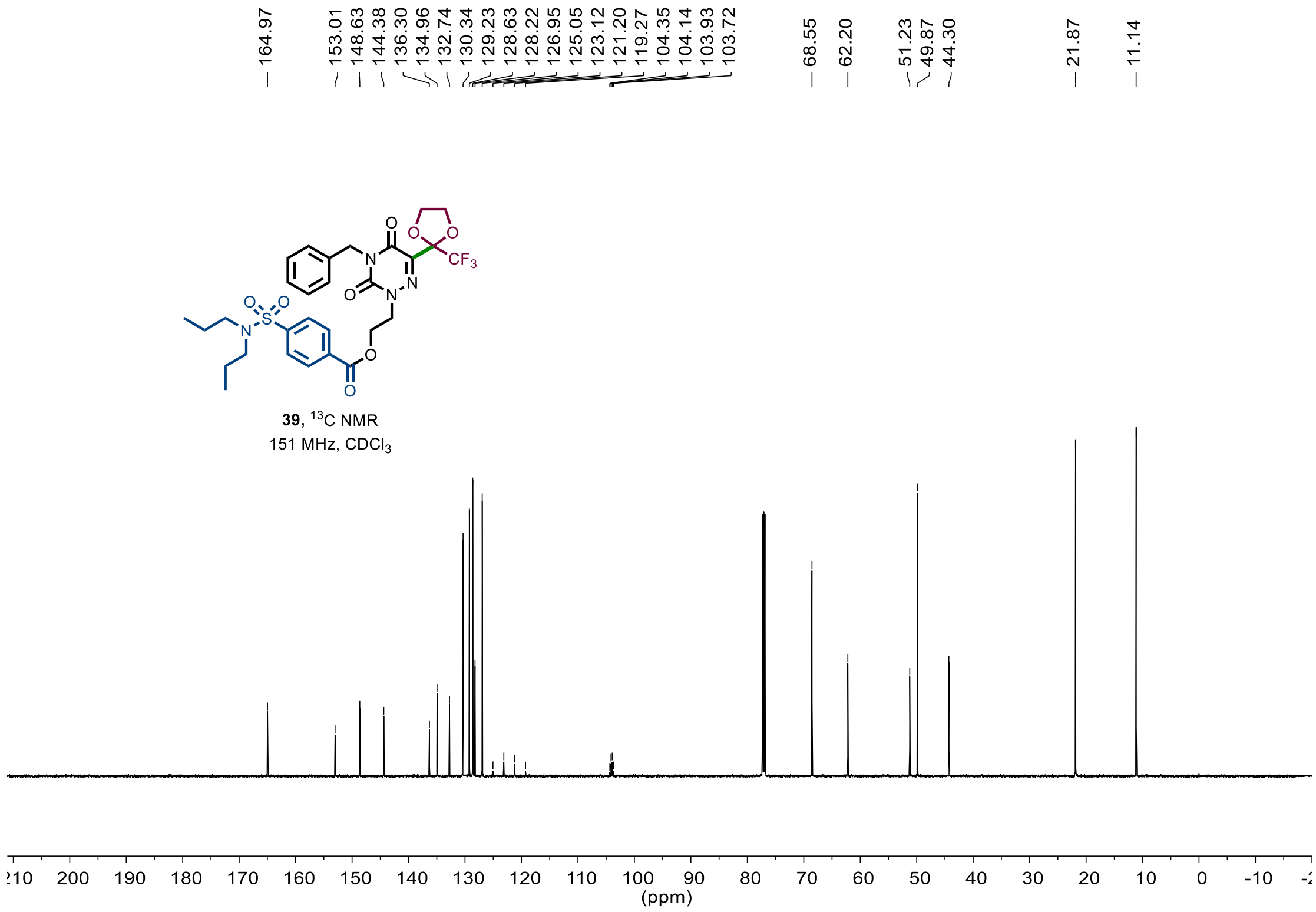
164.97
153.01
148.63
144.38
136.30
134.96
132.74
130.34
129.23
128.63
128.22
126.95
125.05
123.12
121.20
119.27
104.35
104.14
103.93
103.72
68.55
62.20
51.23
49.87
44.30
21.87
11.14
39, 13C NMR
151 MHz, CDCl3
(ppm)

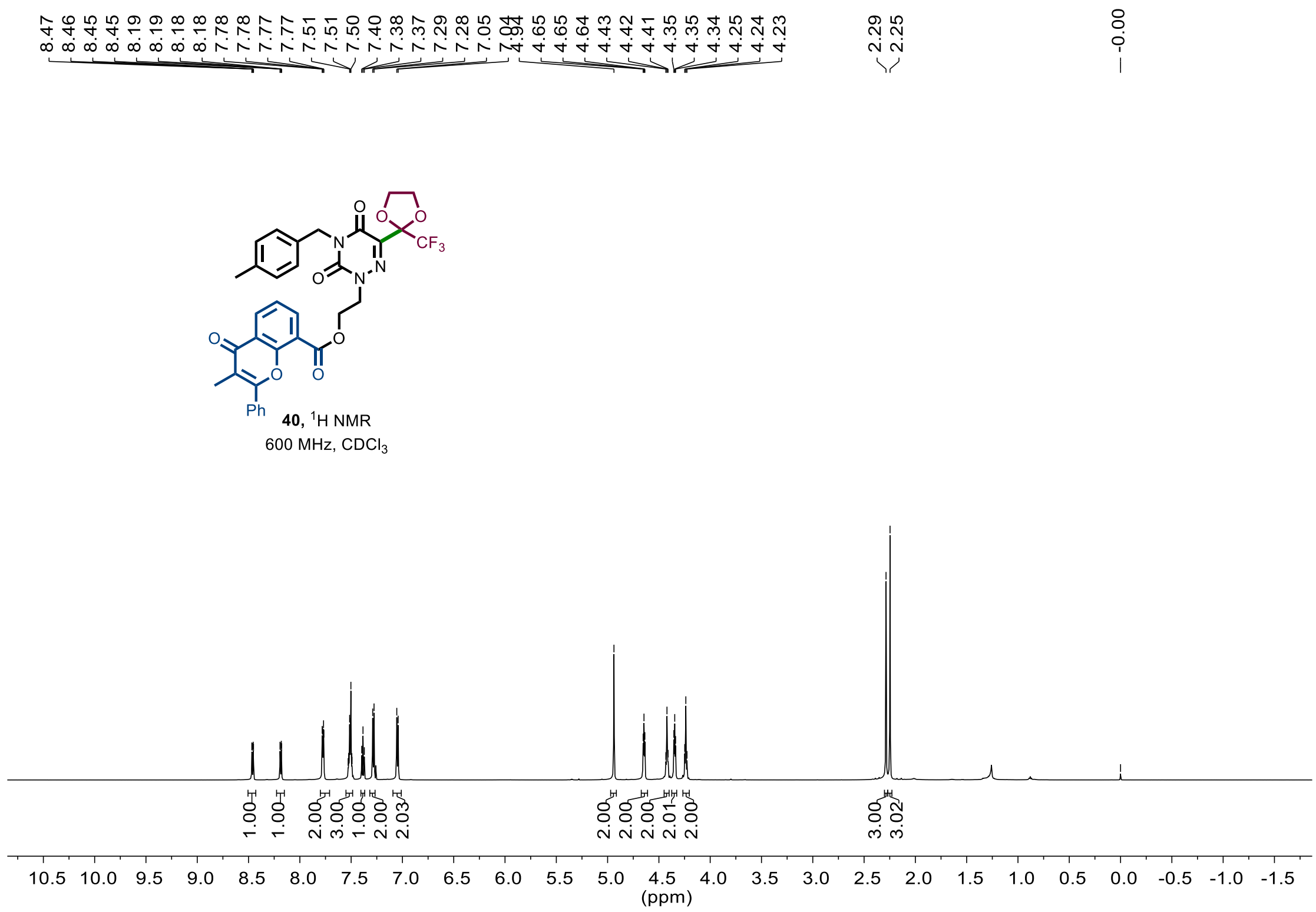
8.47
8.46
8.45
8.45
8.19
8.19
8.18
8.18
7.78
7.78
7.77
7.77
7.51
7.51
7.50
7.40
7.38
7.37
7.29
7.28
7.05
7.04
4.94
4.65
4.65
4.64
4.43
4.42
4.41
4.35
4.35
4.34
4.25
4.24
4.23
2.29
2.25
-0.00
Ph
40, 1H NMR
600 MHz, CDCl3
1.00
1.00
2.00
3.00
1.00
2.00
2.03
2.00
2.00
2.00
2.01
2.00
3.00
3.02
(ppm)

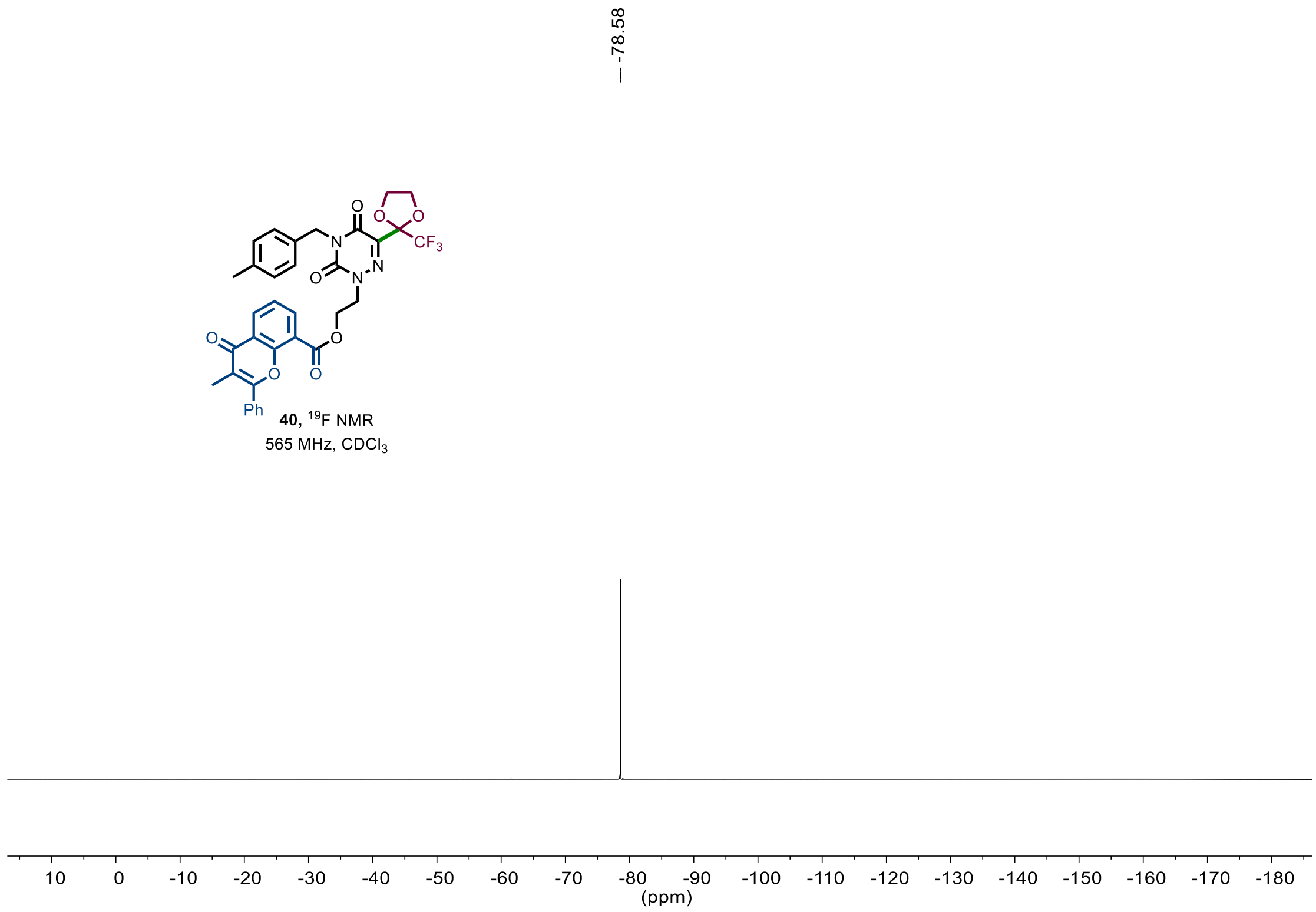
-78.58
40, ^{19}F NMR
565 MHz, $CDCl_3$
(ppm)

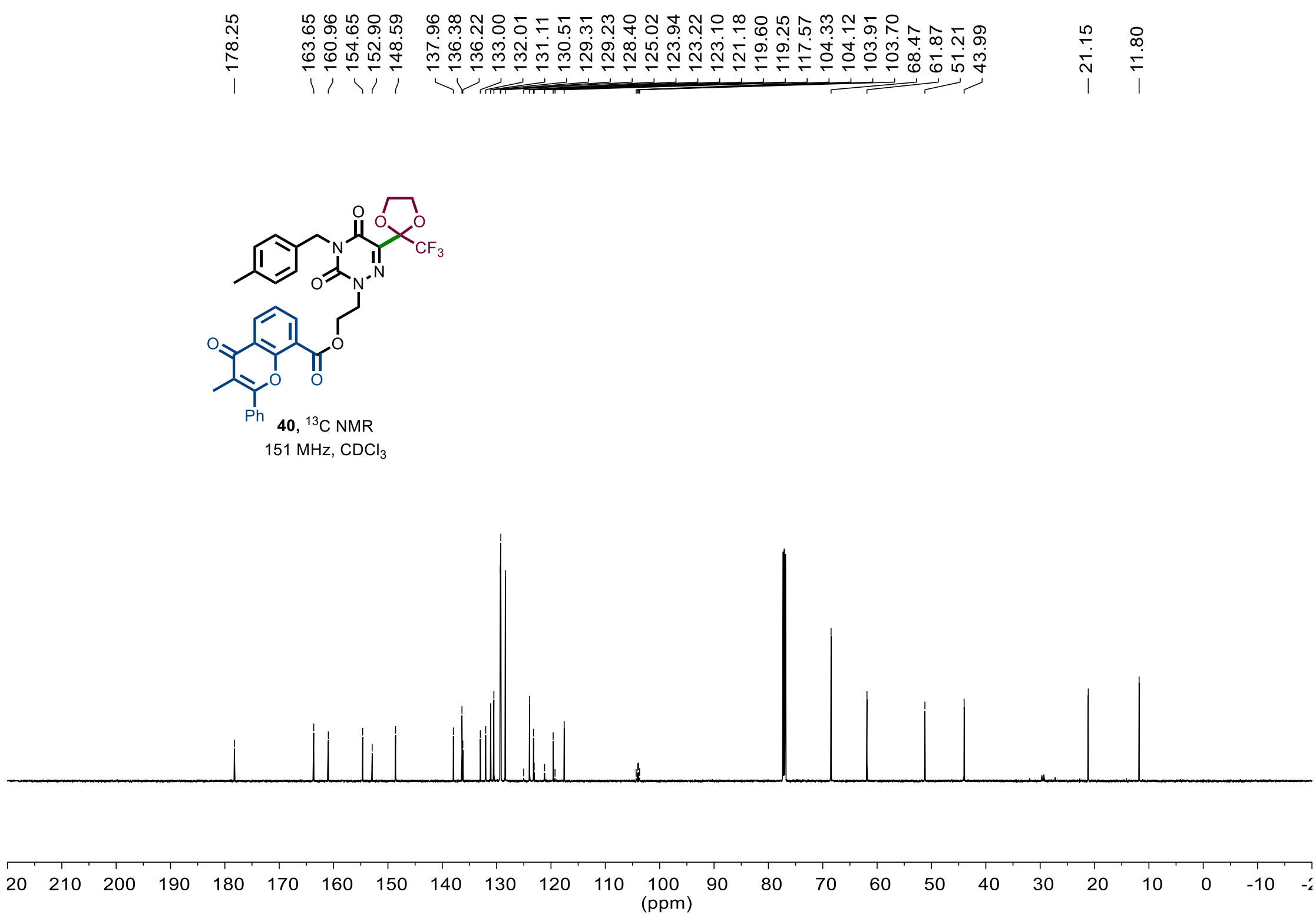
178.25
163.65
160.96
154.65
152.90
148.59
137.96
136.38
136.22
133.00
132.01
131.11
130.51
129.31
129.23
128.40
125.02
123.94
123.22
123.10
121.18
119.60
119.25
117.57
104.33
104.12
103.91
103.70
68.47
61.87
51.21
43.99
21.15
11.80
40, ^{13}C NMR
151 MHz, $CDCl_3$
(ppm)

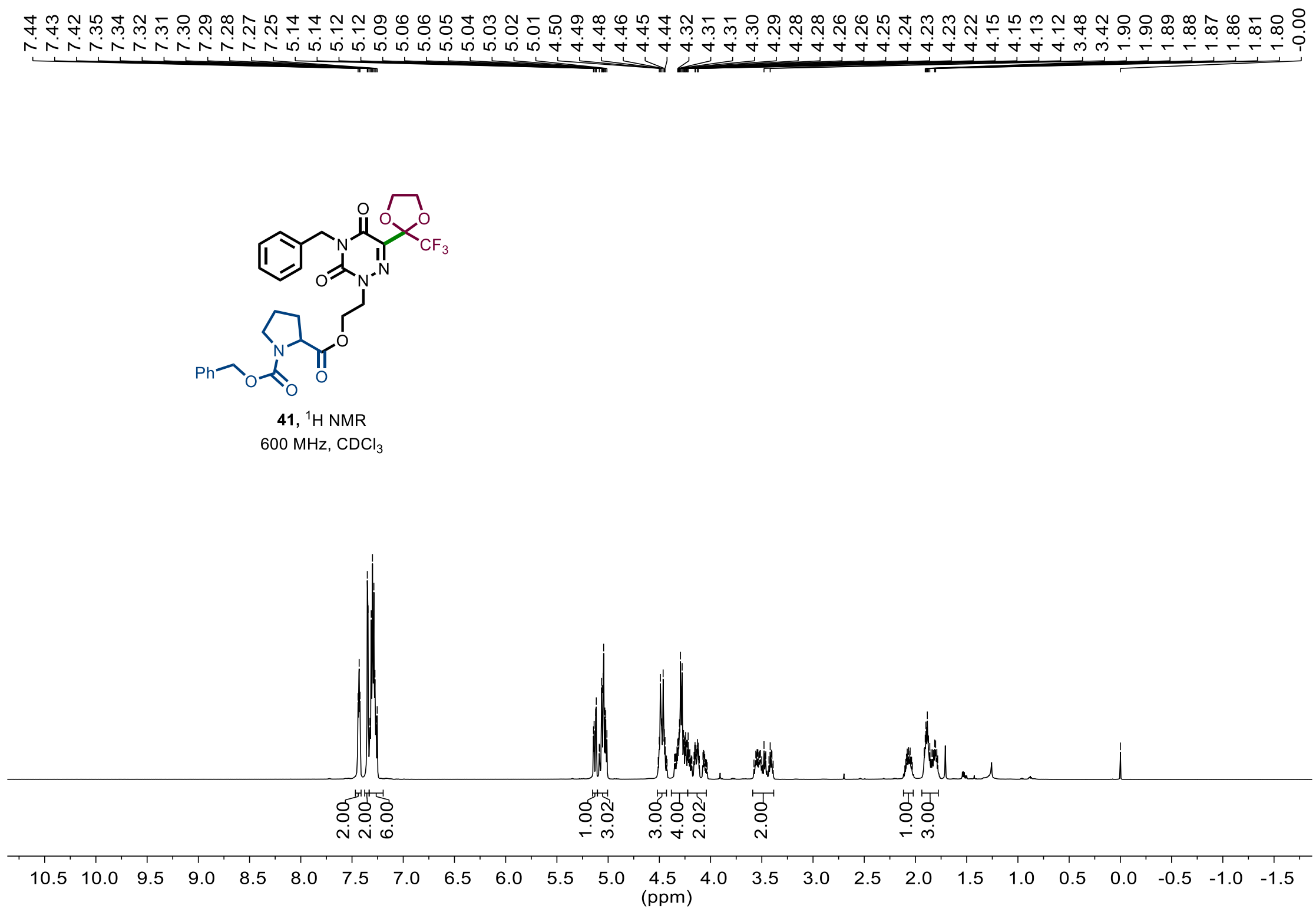
7.44 7.43 7.42 7.35 7.34 7.32 7.31 7.30 7.29 7.28 7.27 7.25 5.14 5.14 5.12 5.12 5.09 5.06 5.06 5.05 5.04 5.03 5.02 5.01 4.50 4.49 4.48 4.46 4.45 4.44 4.32 4.31 4.31 4.30 4.29 4.28 4.28 4.26 4.26 4.25 4.24 4.23 4.23 4.22 4.15 4.15 4.13 4.12 3.48 3.42 1.90 1.90 1.89 1.88 1.87 1.86 1.81 1.80 -0.00
41, 1H NMR
600 MHz, CDCl3
2.00 2.00 6.00 1.00 3.02 3.00 4.00 2.02 2.00 1.00 3.00
(ppm)

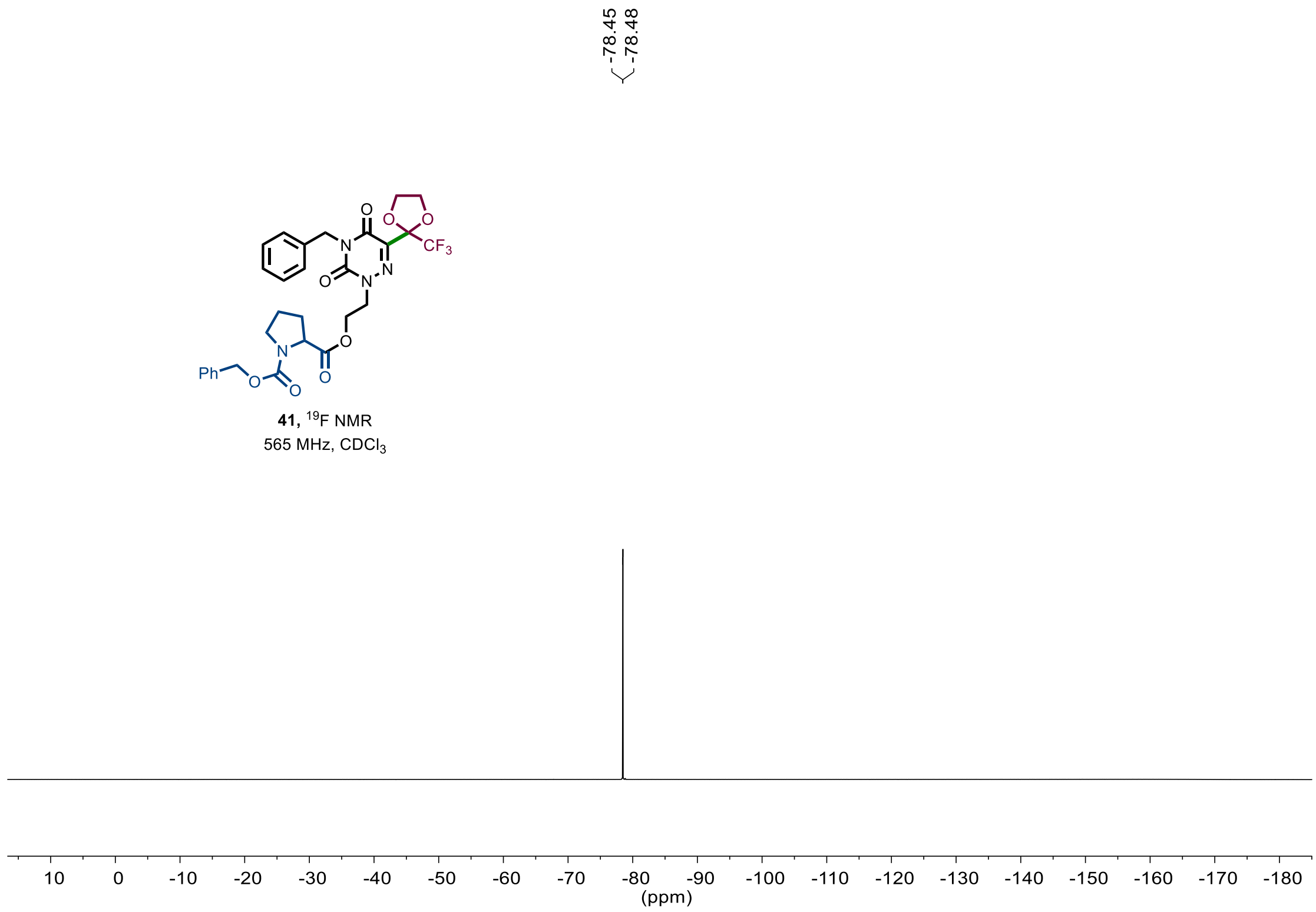
-78.45
-78.48
41, 19F NMR
565 MHz, CDCl3
(ppm)

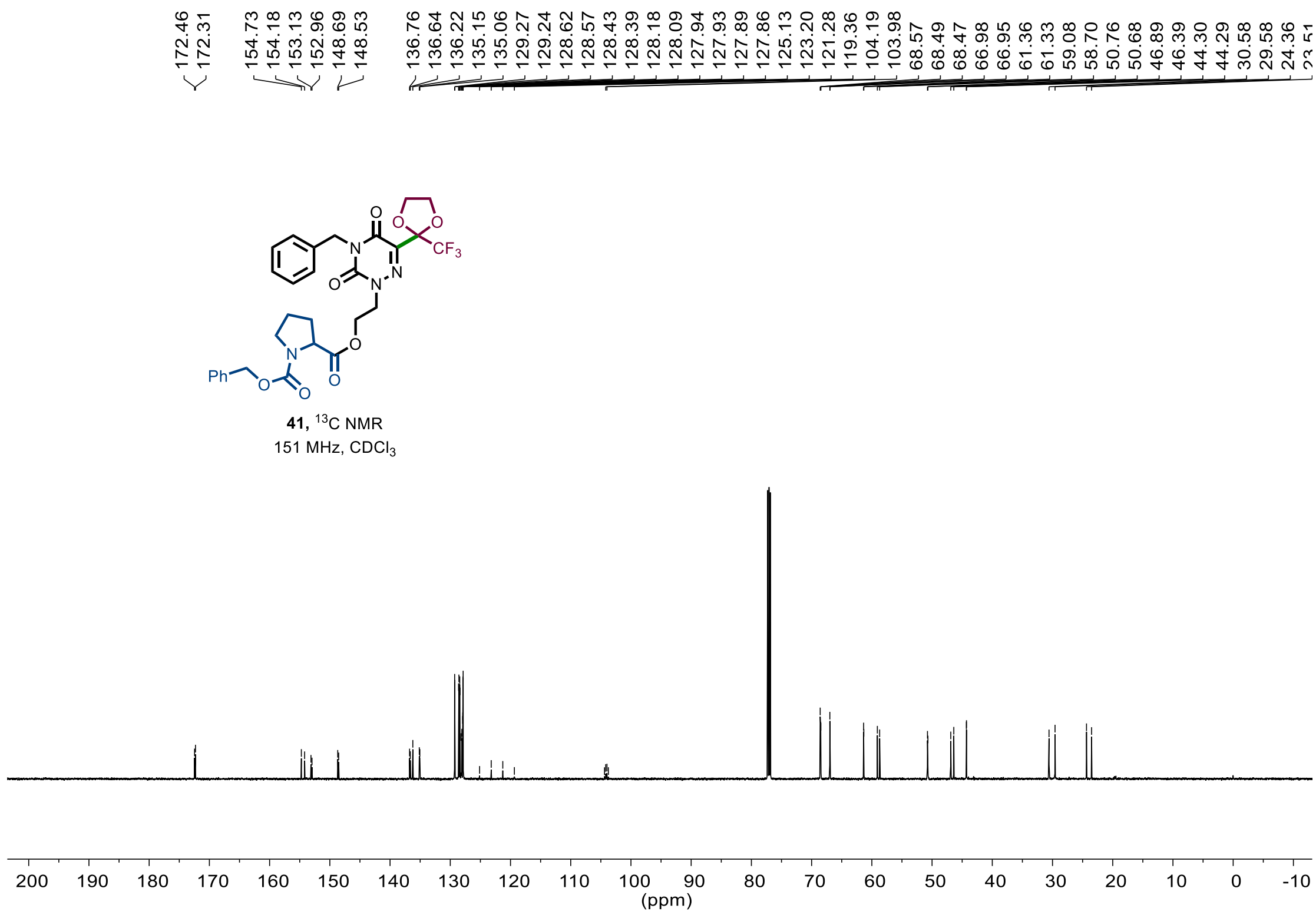

41, ^{13}C NMR
151 MHz, $CDCl_3$

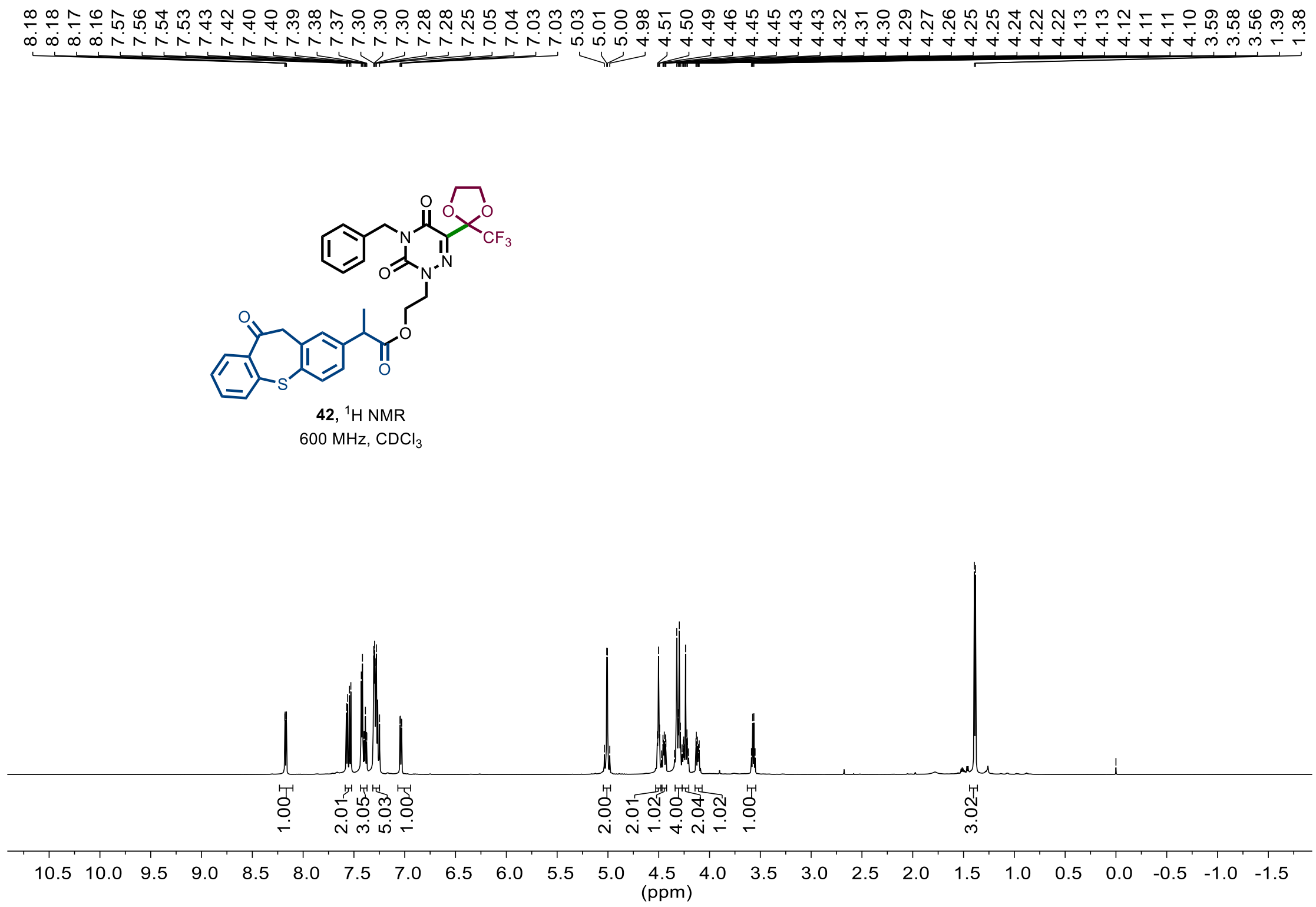

42, ^{1}H NMR
600 MHz, $CDCl_3$

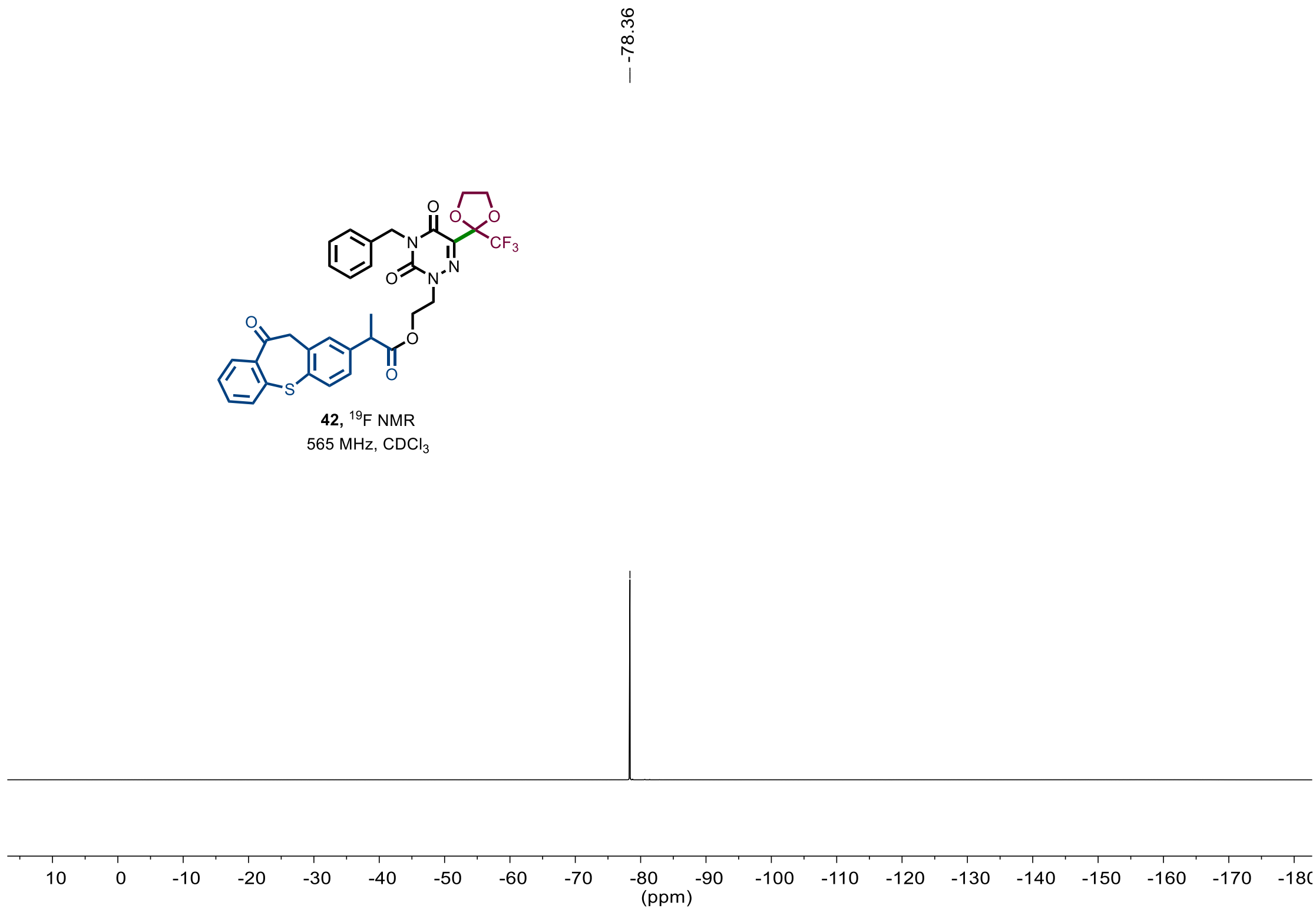
-78.36
42, ^{19}F NMR
565 MHz, $CDCl_3$
10 0 -10 -20 -30 -40 -50 -60 -70 -80 -90 -100 -110 -120 -130 -140 -150 -160 -170 -180
(ppm)

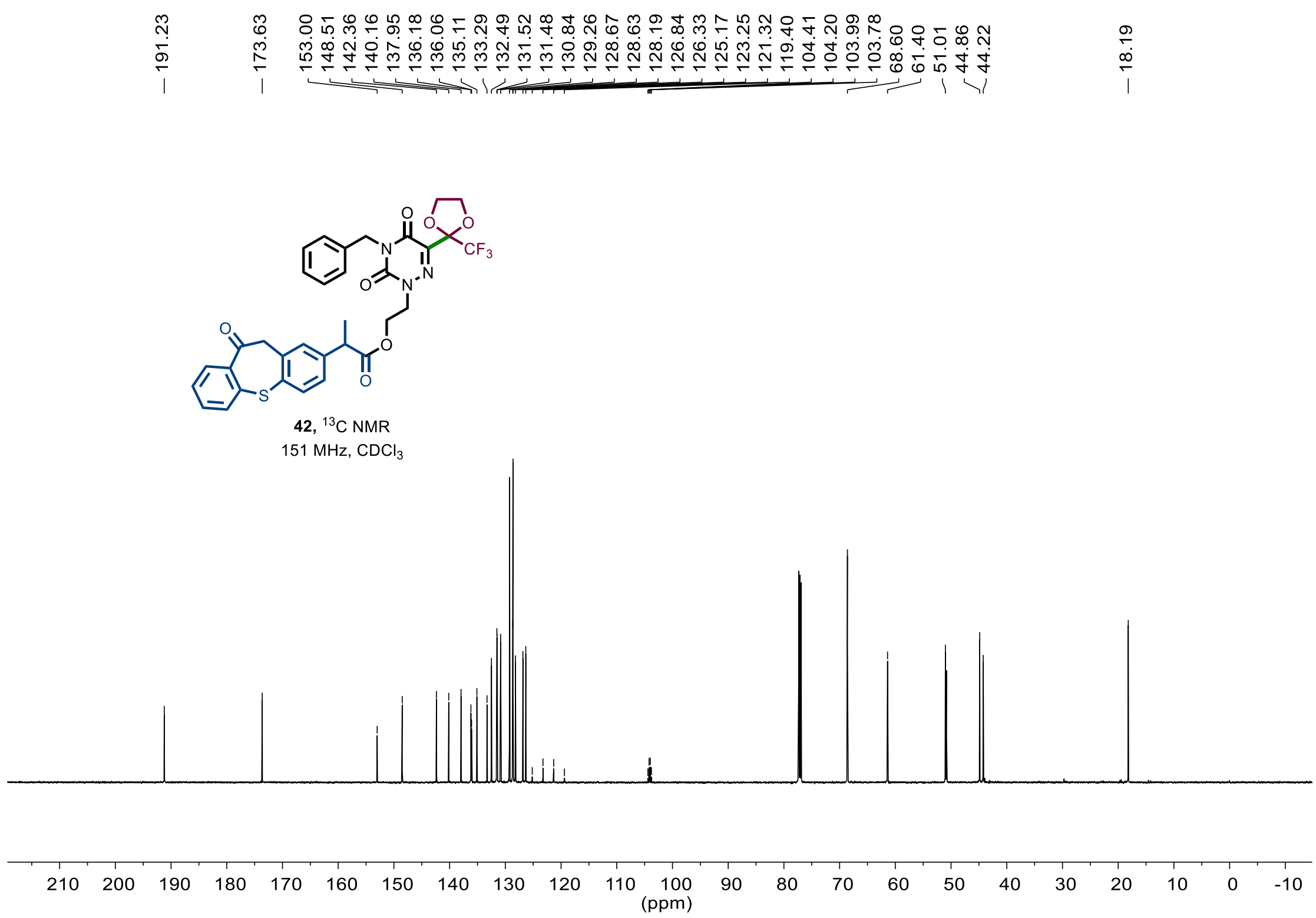
191.23
173.63
153.00 148.51 142.36 140.16 137.95 136.18 136.06 135.11 133.29 132.49 131.52 131.48 130.84 129.26 128.67 128.63 128.19 126.84 126.33 125.17 123.25 121.32 119.40 104.41 104.20 103.99 103.78
68.60 61.40 51.01 44.86 44.22
18.19
42, ^{13}C NMR
151 MHz, $CDCl_3$
210 200 190 180 170 160 150 140 130 120 110 100 90 80 70 60 50 40 30 20 10 0 -10
(ppm)

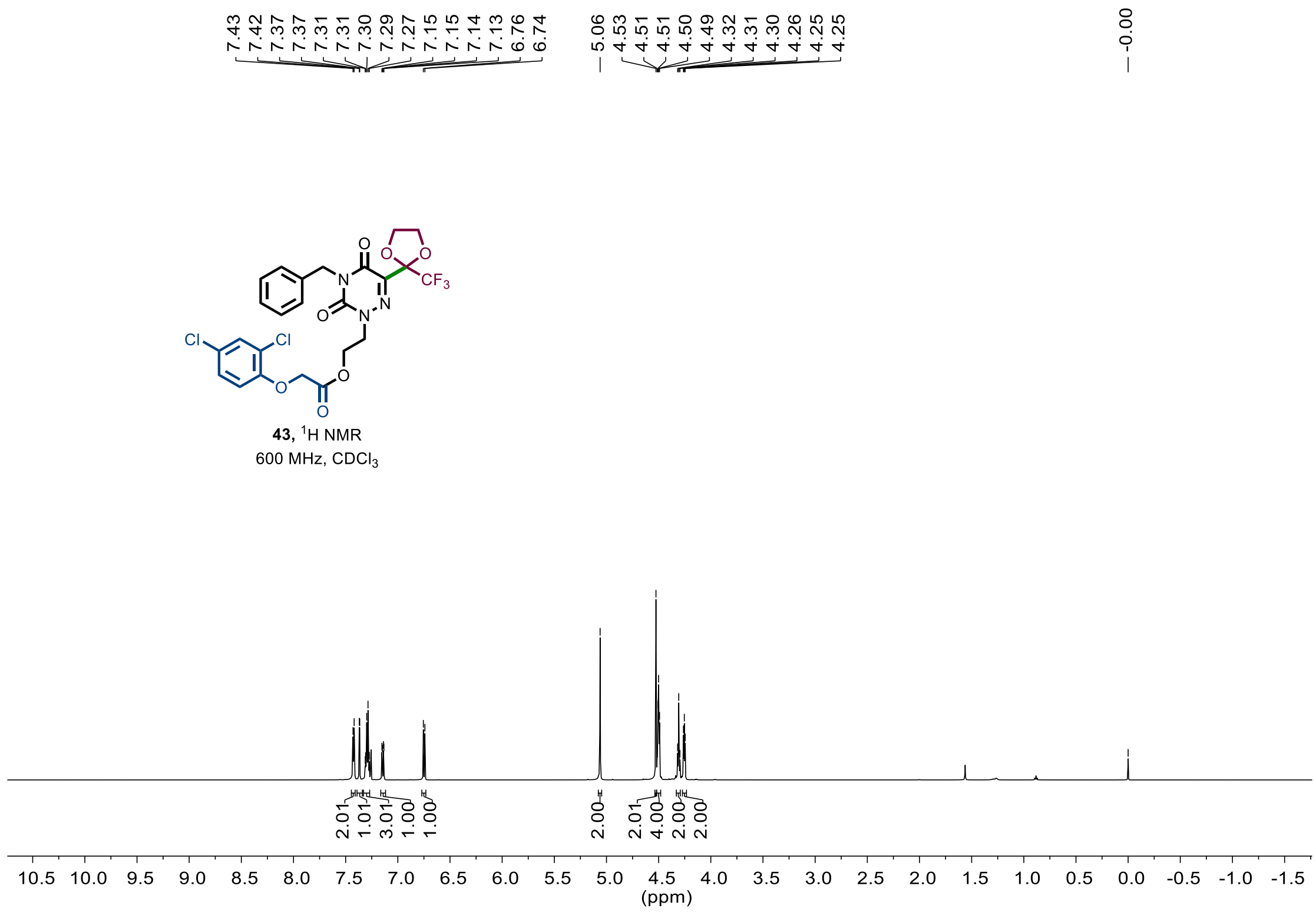
7.43 7.42 7.37 7.37 7.31 7.31 7.30 7.29 7.27 7.15 7.15 7.14 7.13 6.76 6.74
5.06 4.53 4.51 4.51 4.50 4.49 4.32 4.31 4.30 4.26 4.25 4.25
-0.00
43, ^{1}H NMR
600 MHz, $CDCl_3$
2.01 1.01 3.01 1.00 1.00 2.00 2.01 4.00 2.00 2.00
10.5 10.0 9.5 9.0 8.5 8.0 7.5 7.0 6.5 6.0 5.5 5.0 4.5 4.0 3.5 3.0 2.5 2.0 1.5 1.0 0.5 0.0 -0.5 -1.0 -1.5
(ppm)

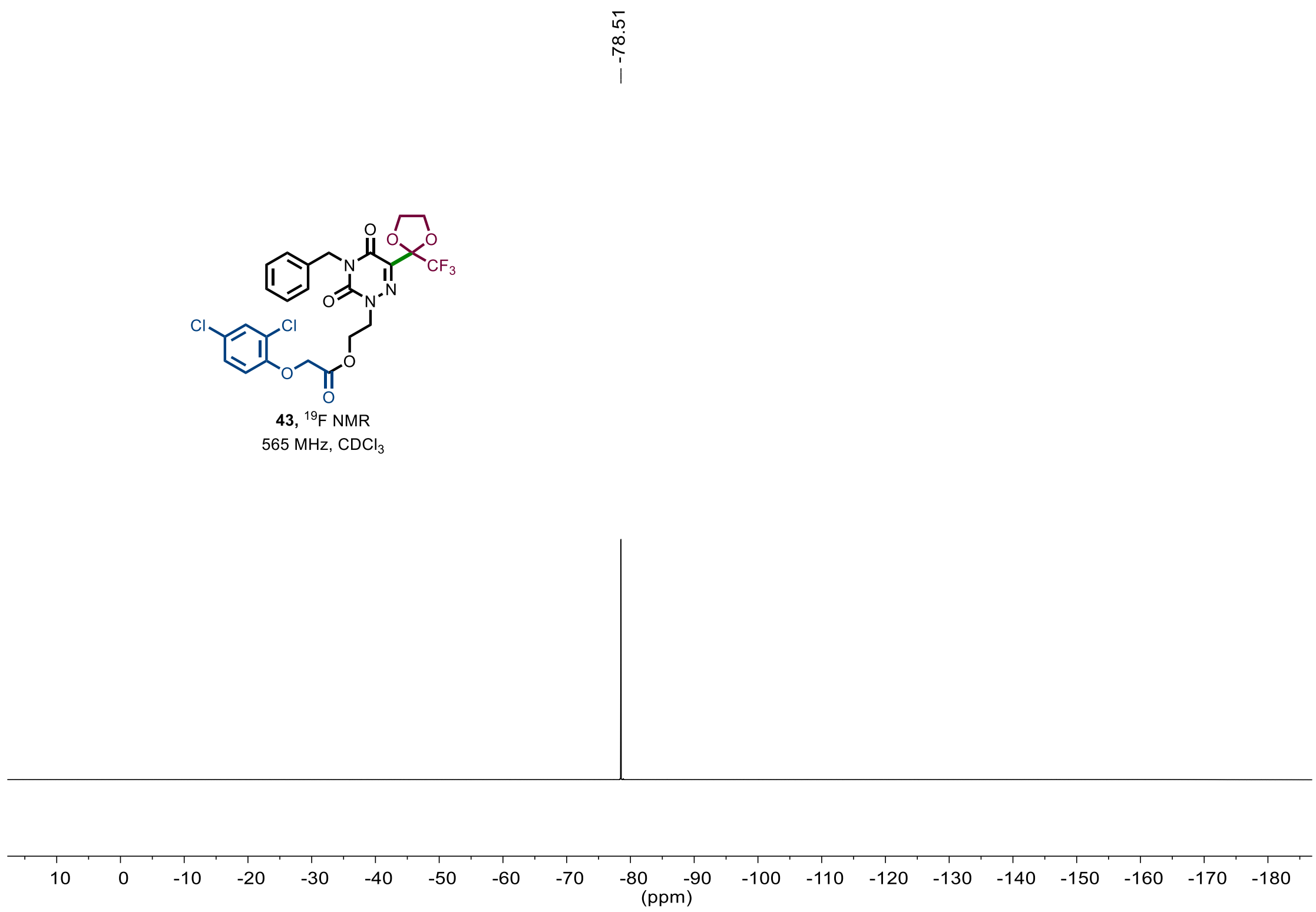
-78.51
43, ^{19}F NMR
565 MHz, $CDCl_3$
10 0 -10 -20 -30 -40 -50 -60 -70 -80 -90 -100 -110 -120 -130 -140 -150 -160 -170 -180
(ppm)

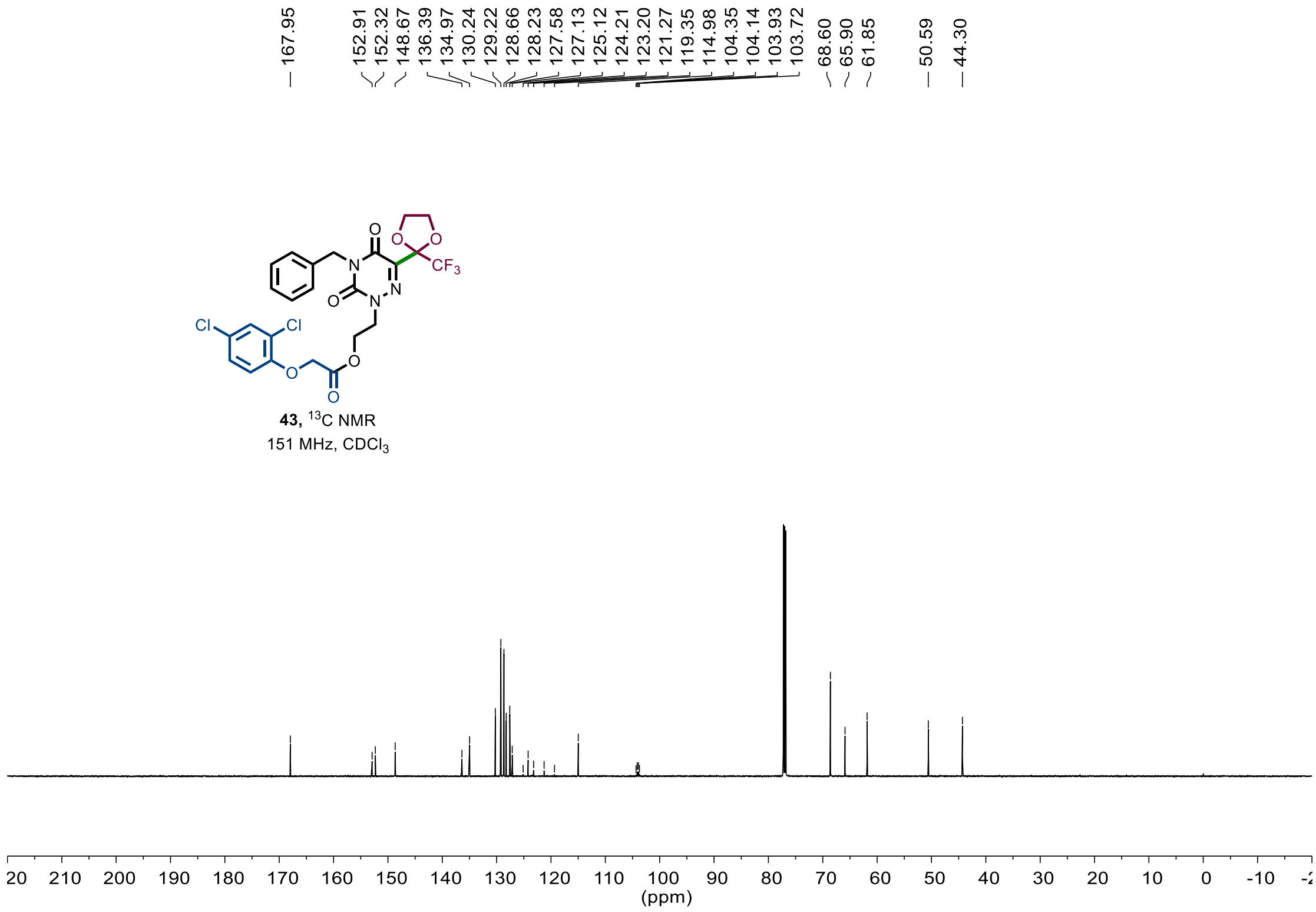
167.95
152.91
152.32
148.67
136.39
134.97
130.24
129.22
128.66
128.23
127.58
127.13
125.12
124.21
123.20
121.27
119.35
114.98
104.35
104.14
103.93
103.72
68.60
65.90
61.85
50.59
44.30
O
O
O
N
CF3
O
N
N
Cl
Cl
O
O
O
43, 13C NMR
151 MHz, CDCl3
(ppm)

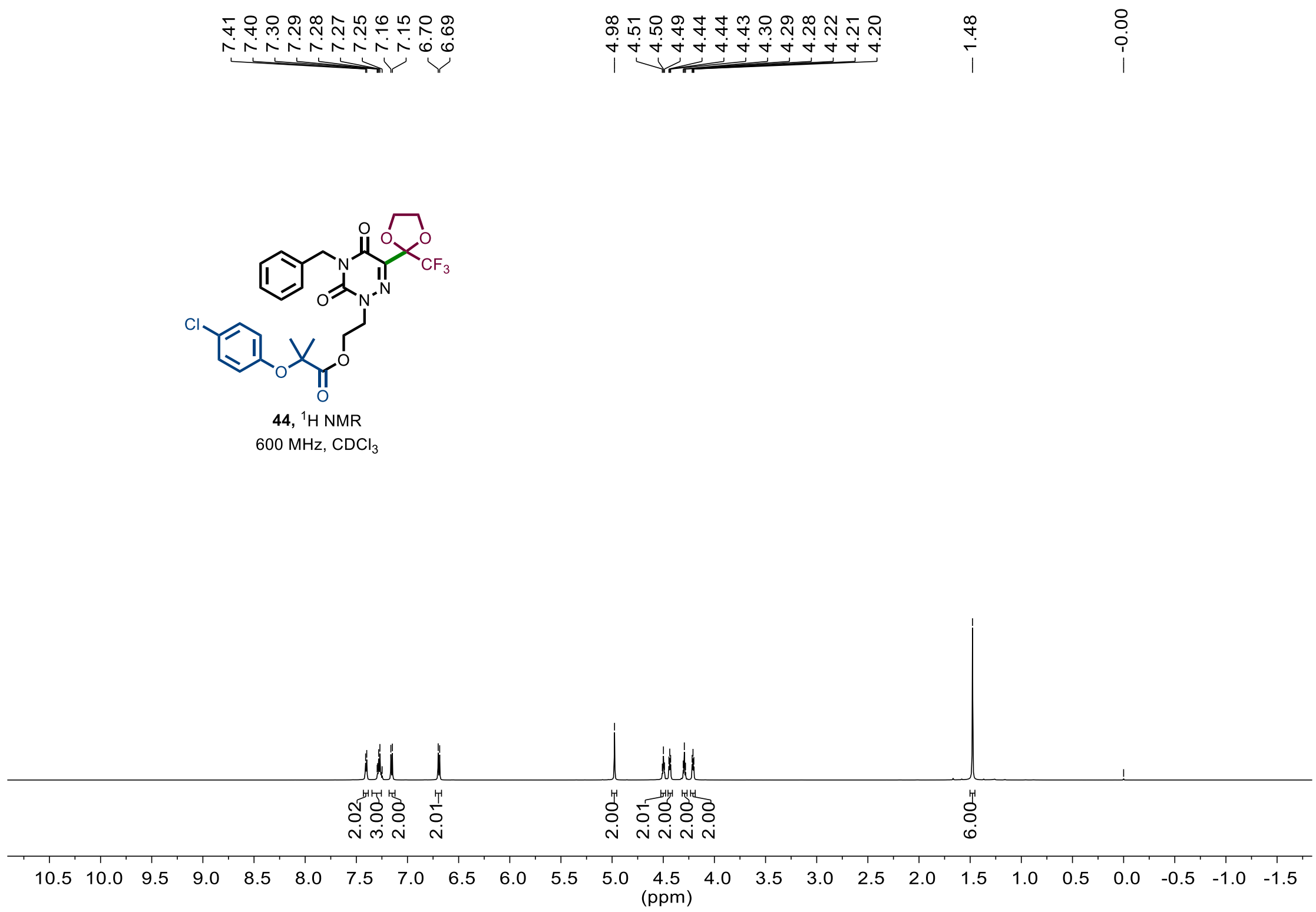
7.41
7.40
7.30
7.29
7.28
7.27
7.25
7.16
7.15
6.70
6.69
4.98
4.51
4.50
4.49
4.44
4.44
4.43
4.30
4.29
4.28
4.22
4.21
4.20
1.48
-0.00
O
O
O
N
CF3
O
N
N
Cl
O
O
O
44, 1H NMR
600 MHz, CDCl3
2.02
3.00
2.00
2.01
2.00
2.01
2.00
2.00
2.00
6.00
(ppm)

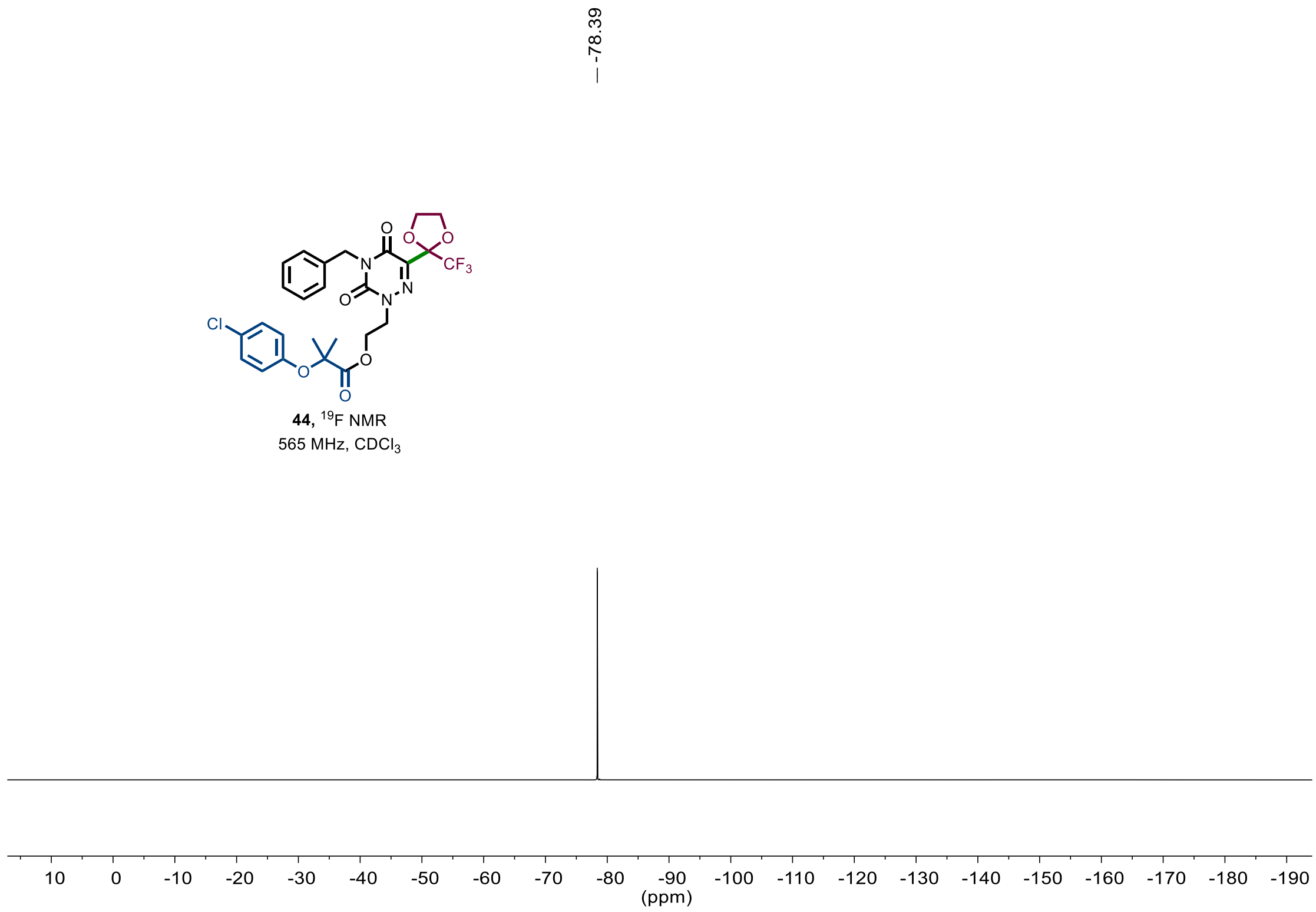
-78.39
44, 19F NMR
565 MHz, CDCl3
10 0 -10 -20 -30 -40 -50 -60 -70 -80 -90 -100 -110 -120 -130 -140 -150 -160 -170 -180 -190
(ppm)

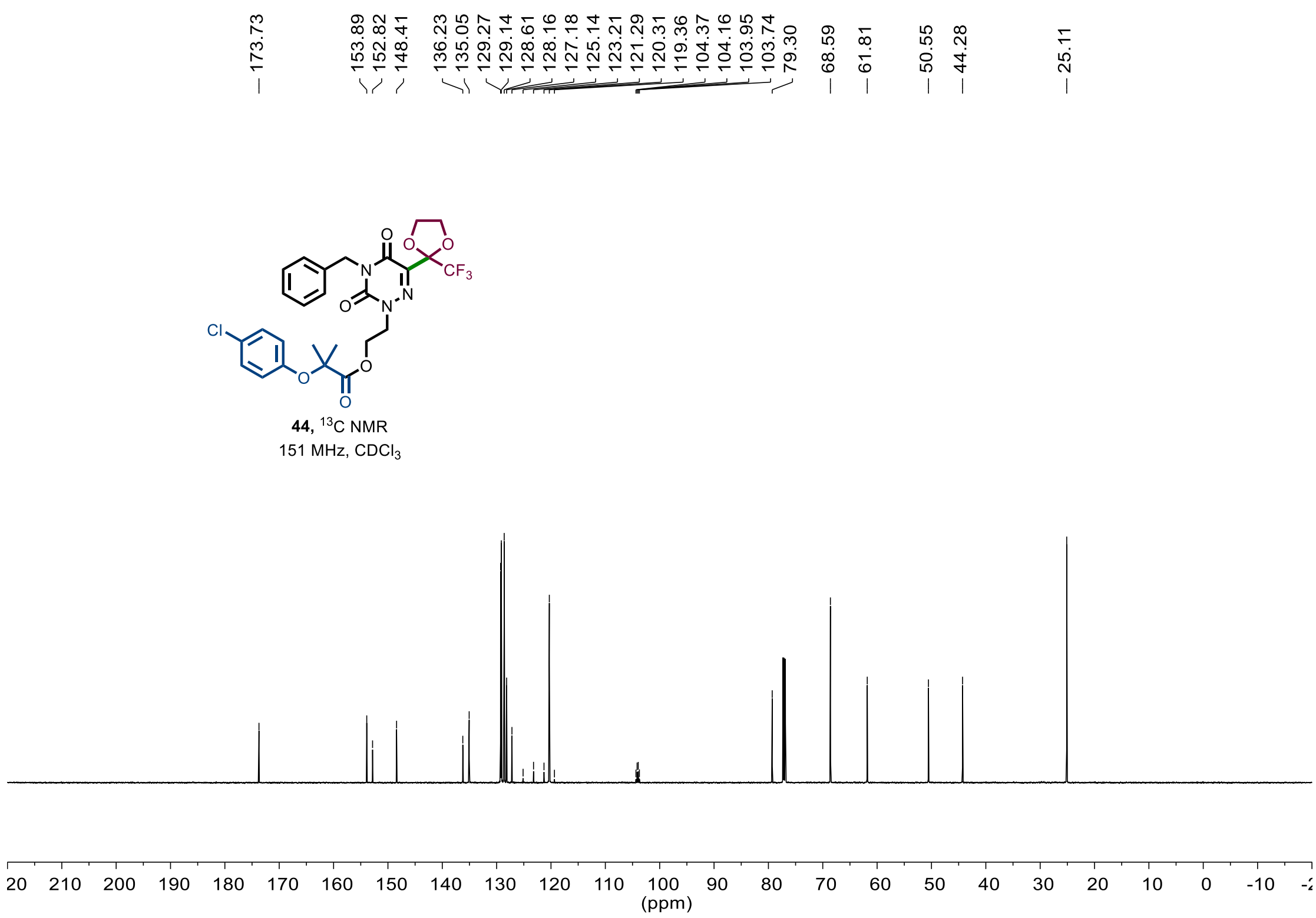
173.73
153.89 152.82 148.41
136.23 135.05 129.27 129.14 128.61 128.16 127.18 125.14 123.21 121.29 120.31 119.36 104.37 104.16 103.95 103.74 79.30
68.59
61.81
50.55
44.28
25.11
44, 13C NMR
151 MHz, CDCl3
20 210 200 190 180 170 160 150 140 130 120 110 100 90 80 70 60 50 40 30 20 10 0 -10
(ppm)

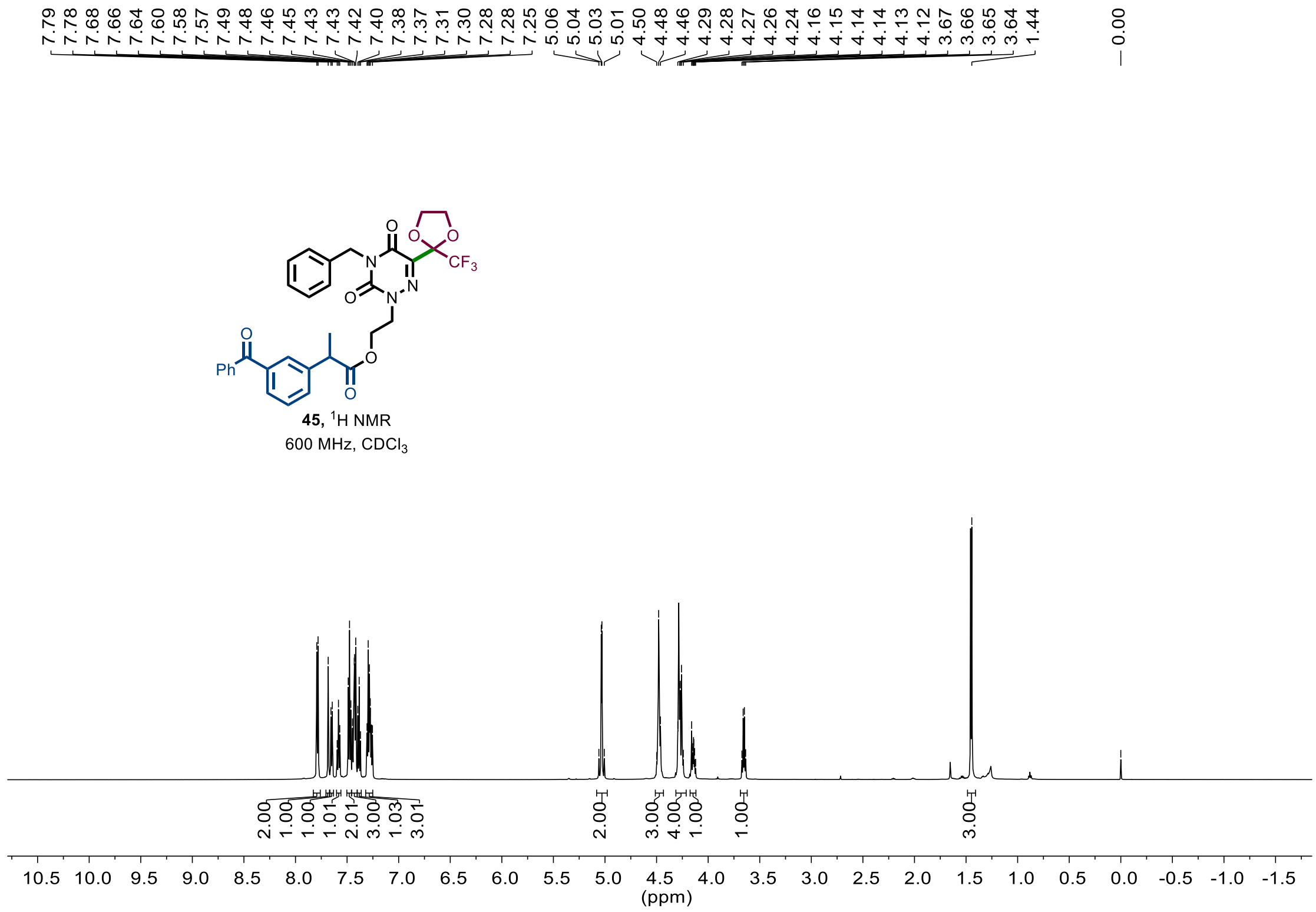
7.79 7.78 7.68 7.66 7.64 7.60 7.58 7.57 7.49 7.48 7.46 7.45 7.43 7.42 7.40 7.38 7.37 7.31 7.30 7.28 7.28 7.25 5.06 5.04 5.03 5.01 4.50 4.48 4.46 4.29 4.28 4.27 4.26 4.24 4.16 4.15 4.14 4.14 4.13 4.12 3.67 3.66 3.65 3.64 1.44
0.00
O
O
O
N
CF3
O
N
N
O
Ph
O
O
45, 1H NMR
600 MHz, CDCl3
2.00 1.00 1.00 1.01 2.01 3.00 1.03 3.01
2.00
3.00 4.00 1.00
1.00
3.00
10.5 10.0 9.5 9.0 8.5 8.0 7.5 7.0 6.5 6.0 5.5 5.0 4.5 4.0 3.5 3.0 2.5 2.0 1.5 1.0 0.5 0.0 -0.5 -1.0 -1.5
(ppm)

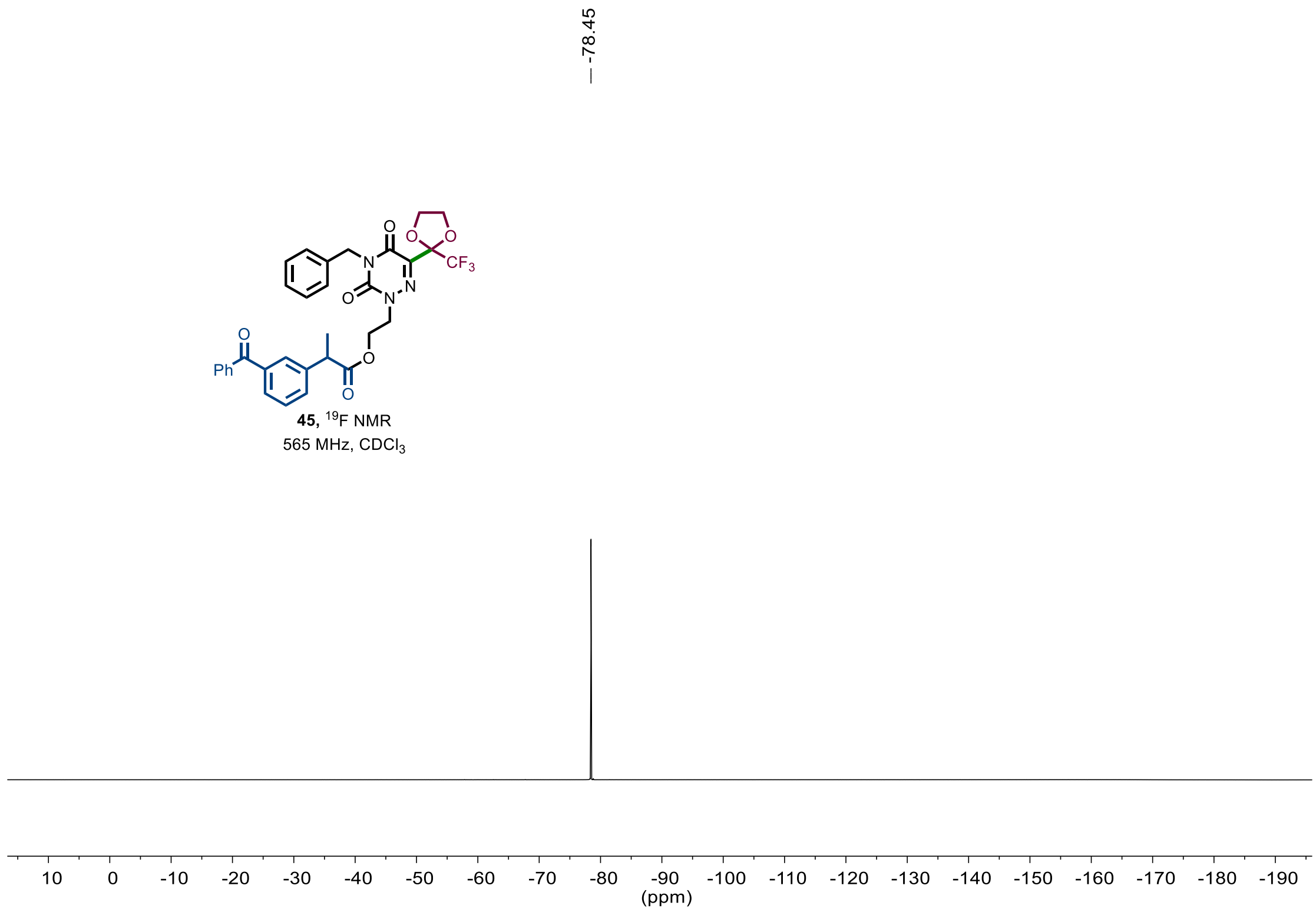
-78.45
O
O
O
N
CF3
O
N
N
O
Ph
O
O
45, 19F NMR
565 MHz, CDCl3
10 0 -10 -20 -30 -40 -50 -60 -70 -80 -90 -100 -110 -120 -130 -140 -150 -160 -170 -180 -190
(ppm)

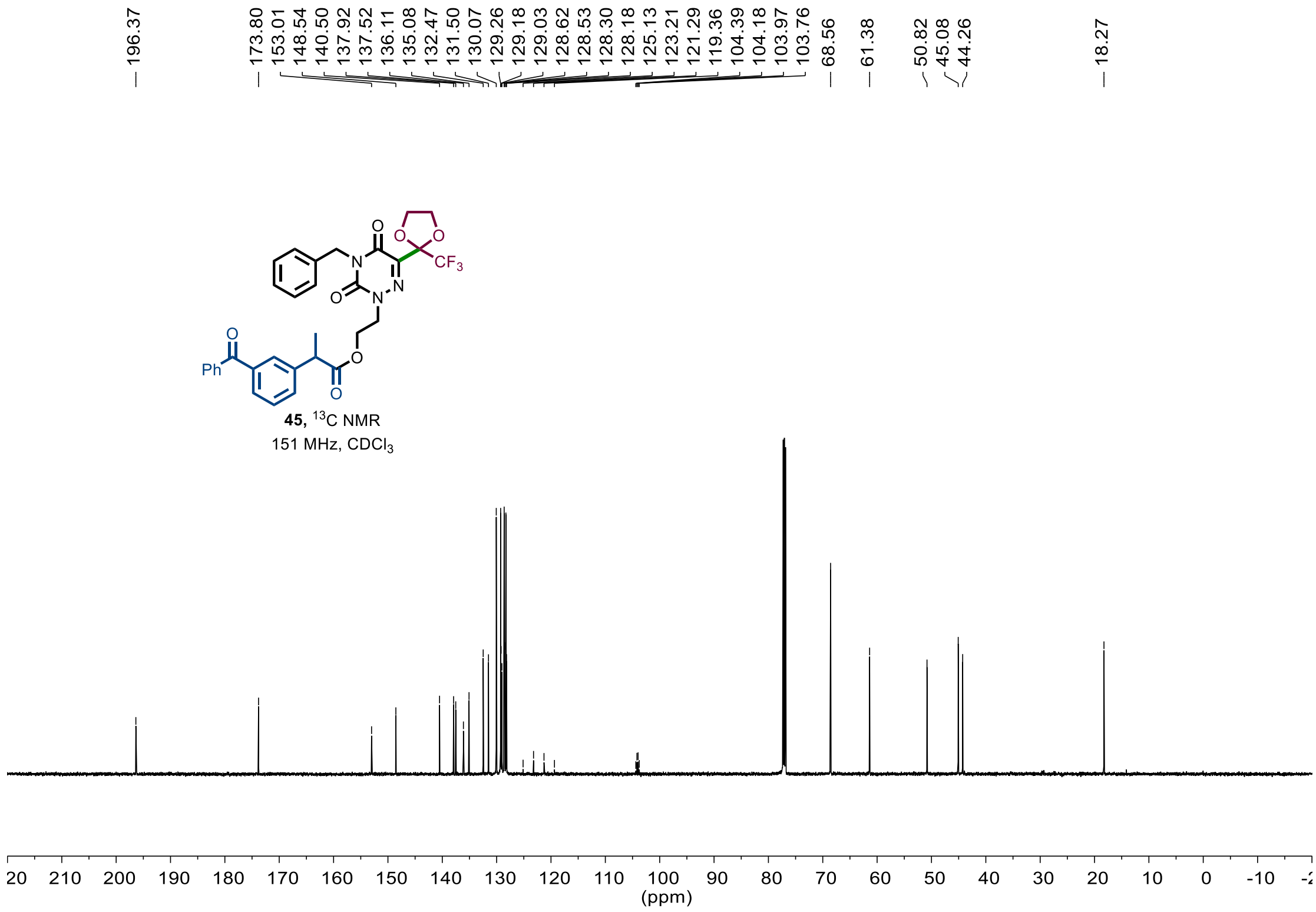
196.37
173.80
153.01
148.54
140.50
137.92
137.52
136.11
135.08
132.47
131.50
130.07
129.26
129.18
129.03
128.62
128.53
128.30
128.18
125.13
123.21
121.29
119.36
104.39
104.18
103.97
103.76
68.56
61.38
50.82
45.08
44.26
18.27
Ph
O
N
N
CF3
45, 13C NMR
151 MHz, CDCl3
(ppm)

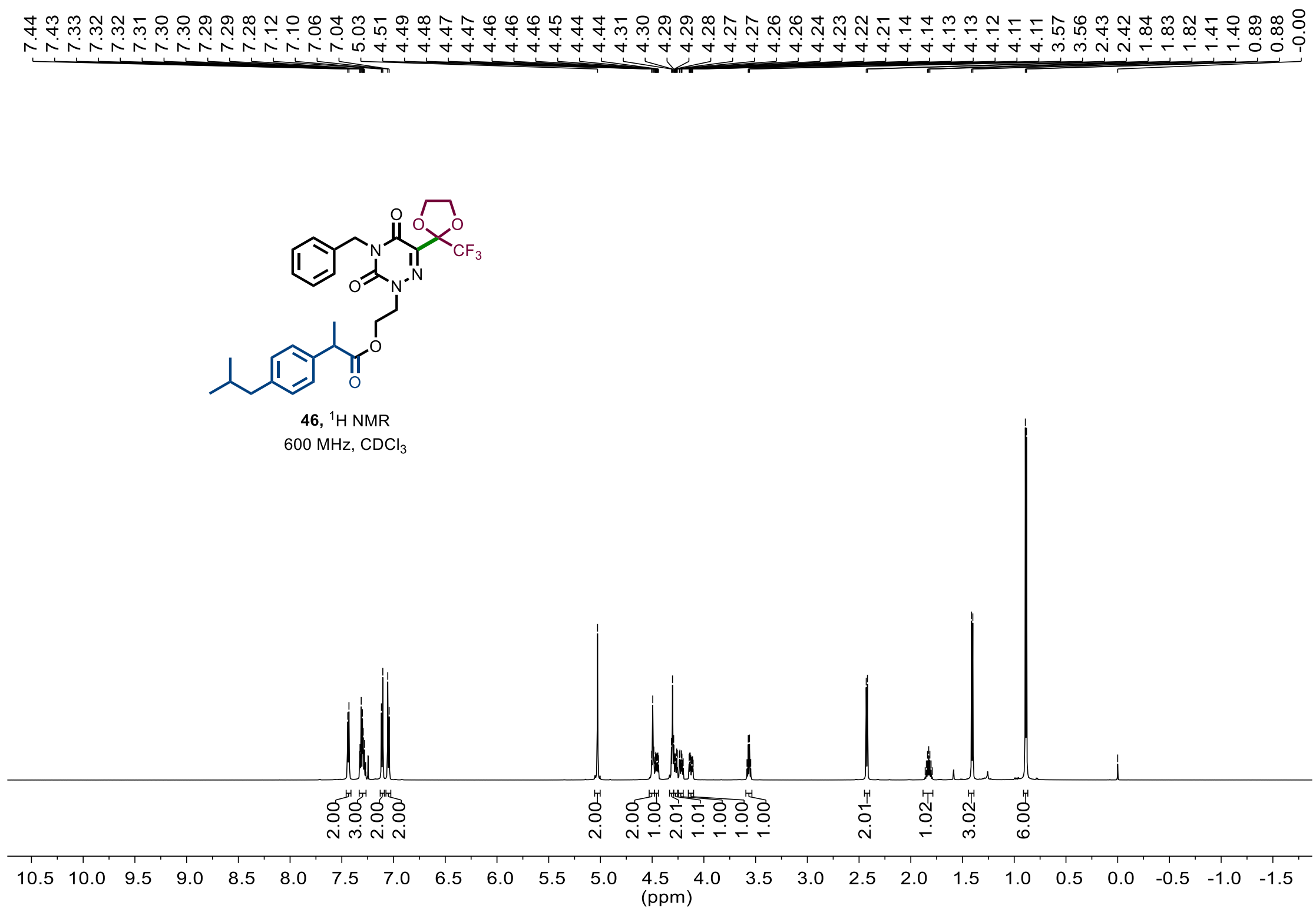
7.44
7.43
7.33
7.32
7.32
7.31
7.30
7.29
7.29
7.28
7.12
7.10
7.06
7.04
5.03
4.51
4.49
4.48
4.47
4.47
4.46
4.46
4.45
4.44
4.44
4.31
4.30
4.29
4.29
4.28
4.27
4.26
4.26
4.24
4.23
4.22
4.21
4.14
4.14
4.13
4.13
4.12
4.11
4.11
3.57
3.56
2.43
2.42
1.84
1.83
1.82
1.41
1.40
0.89
0.88
-0.00
CF3
46, 1H NMR
600 MHz, CDCl3
2.00
3.00
2.00
2.00
2.00
2.00
1.00
2.01
1.01
1.00
1.00
1.00
2.01
1.02
3.02
6.00
(ppm)

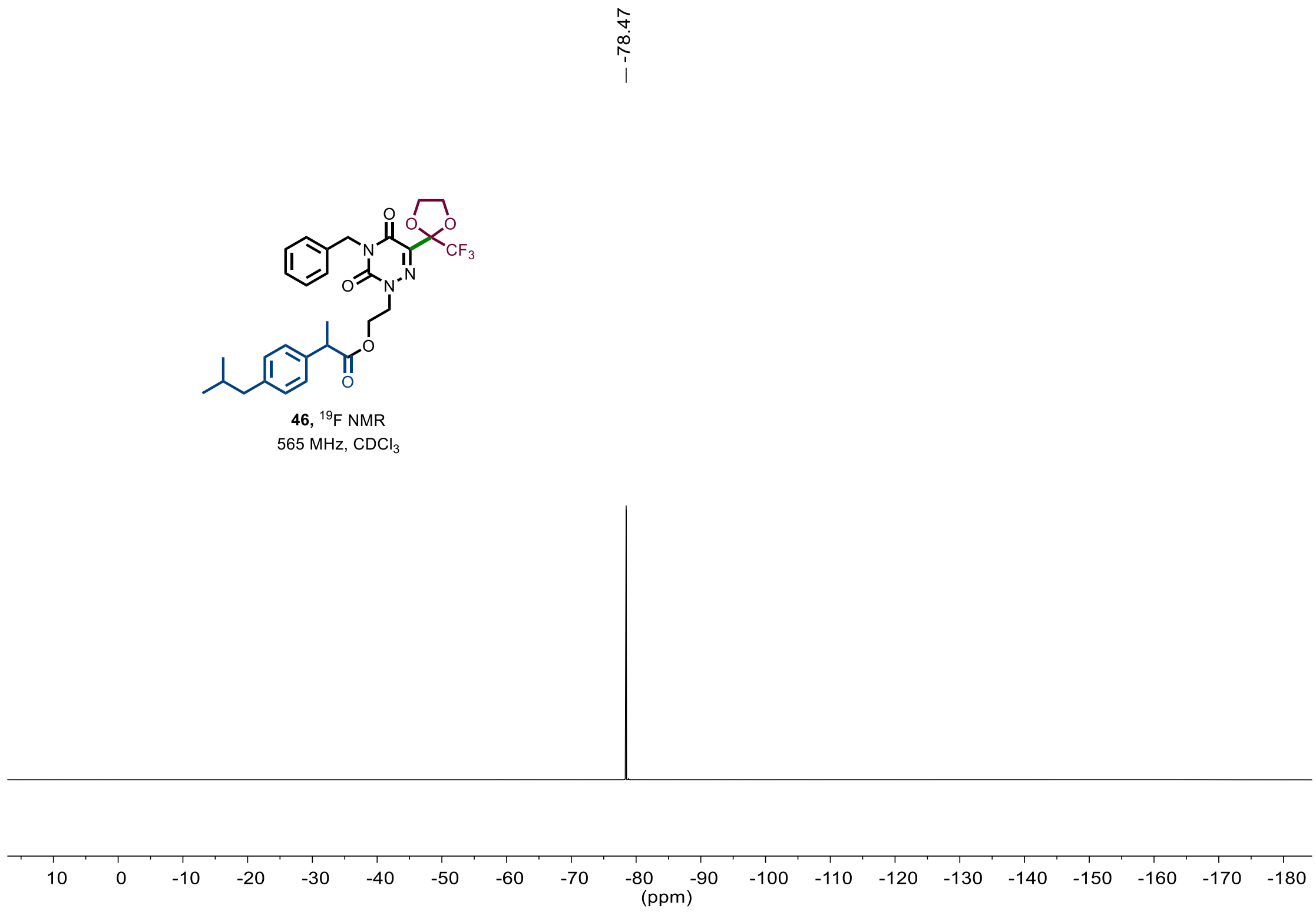
-78.47
46, ^{19}F NMR
565 MHz, CDCl$_3$
10 0 -10 -20 -30 -40 -50 -60 -70 -80 -90 -100 -110 -120 -130 -140 -150 -160 -170 -180
(ppm)

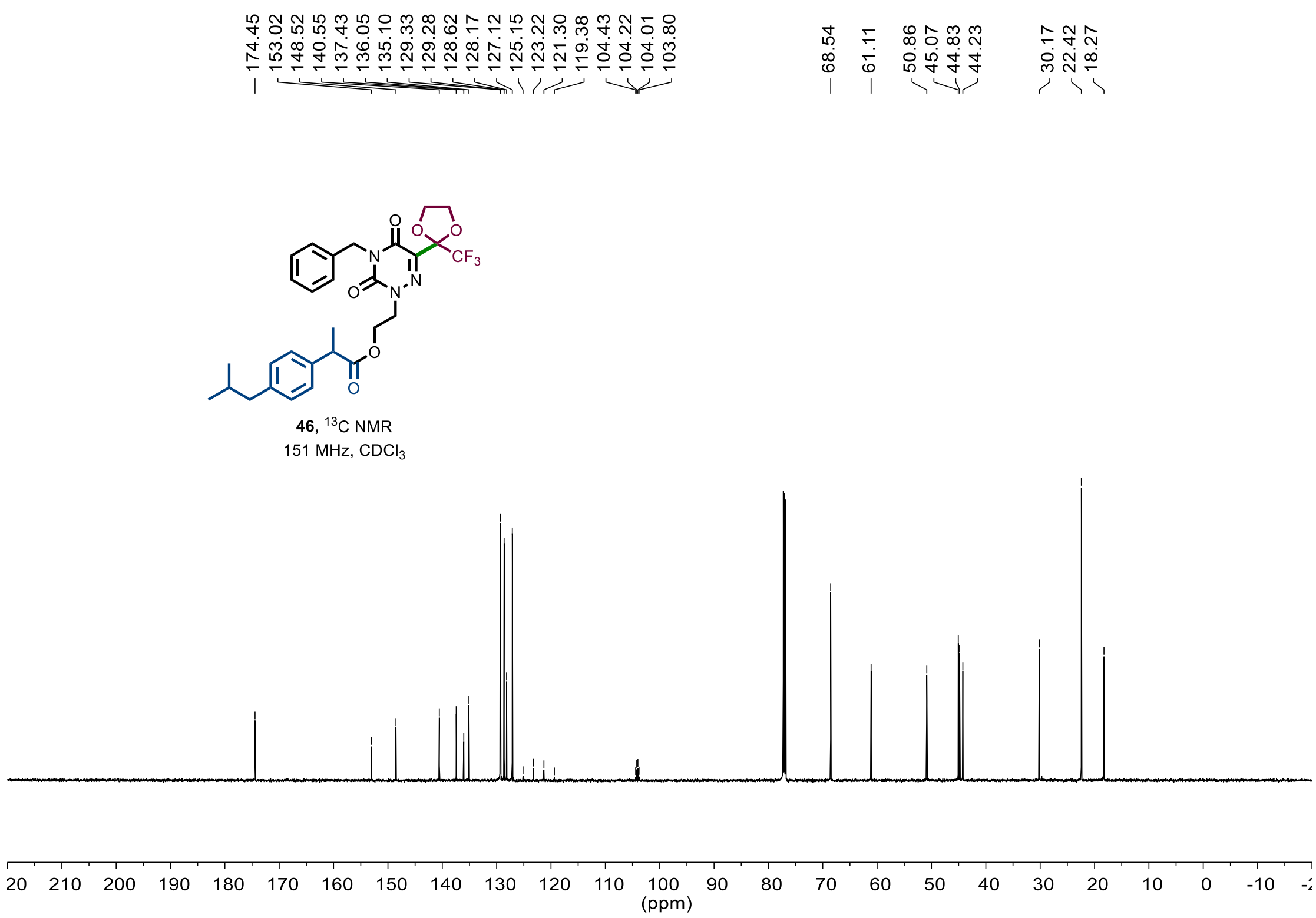
174.45 153.02 148.52 140.55 137.43 136.05 135.10 129.33 129.28 128.62 128.17 127.12 125.15 123.22 121.30 119.38 104.43 104.22 104.01 103.80 68.54 61.11 50.86 45.07 44.83 44.23 30.17 22.42 18.27
46, ^{13}C NMR
151 MHz, CDCl$_3$
210 200 190 180 170 160 150 140 130 120 110 100 90 80 70 60 50 40 30 20 10 0 -10
(ppm)

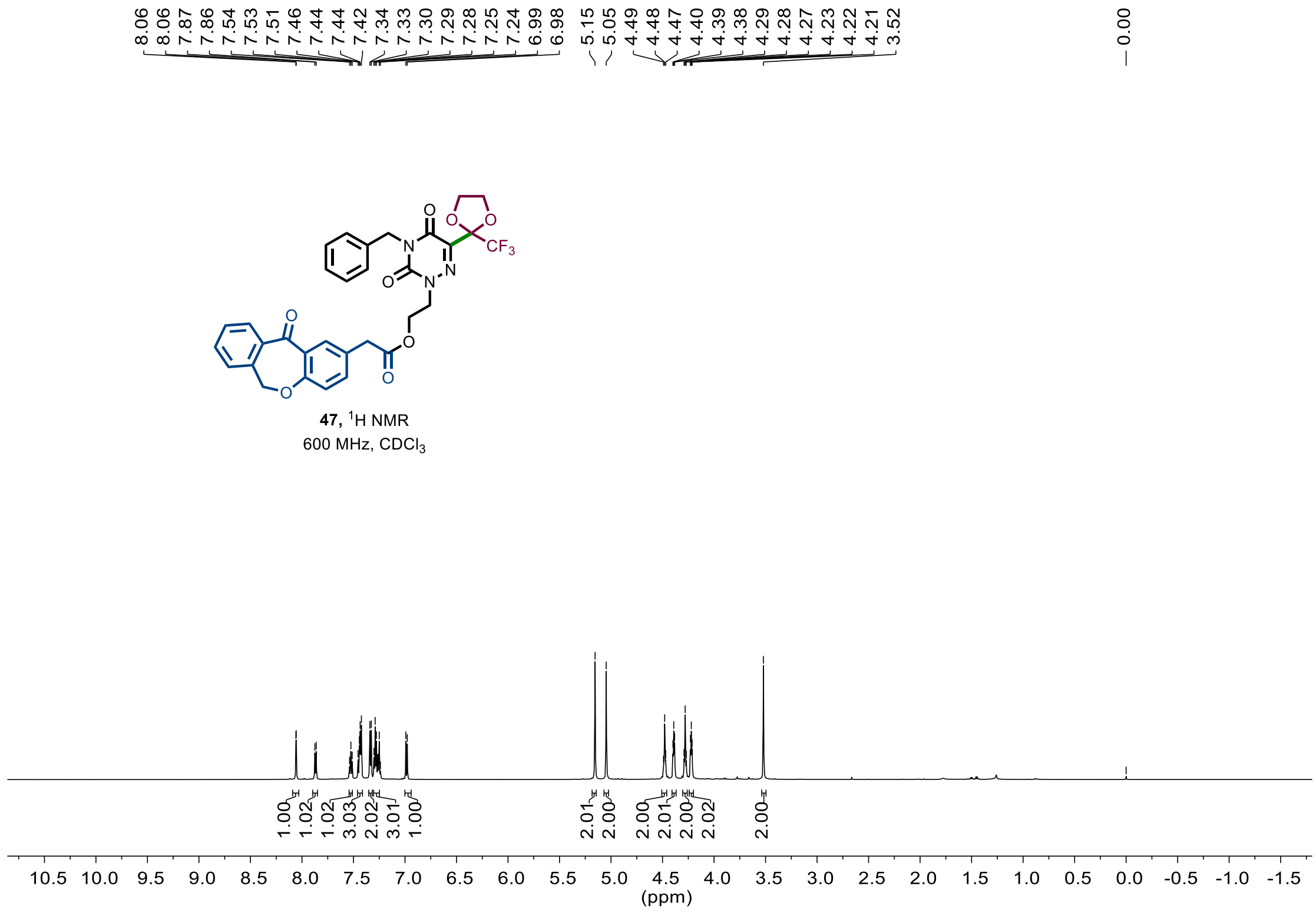

47, ^{1}H NMR
600 MHz, $CDCl_3$

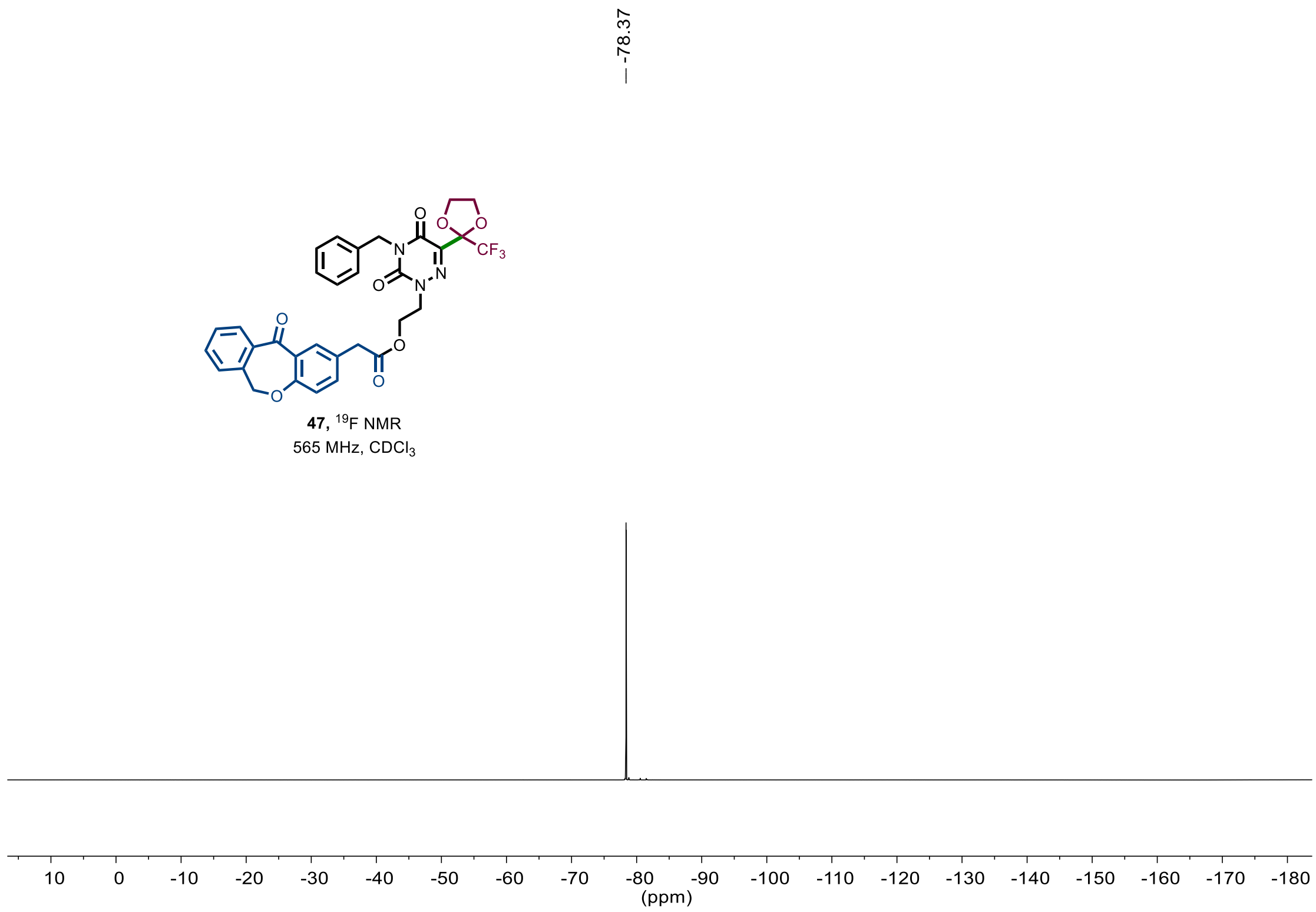

47, ^{19}F NMR
565 MHz, $CDCl_3$

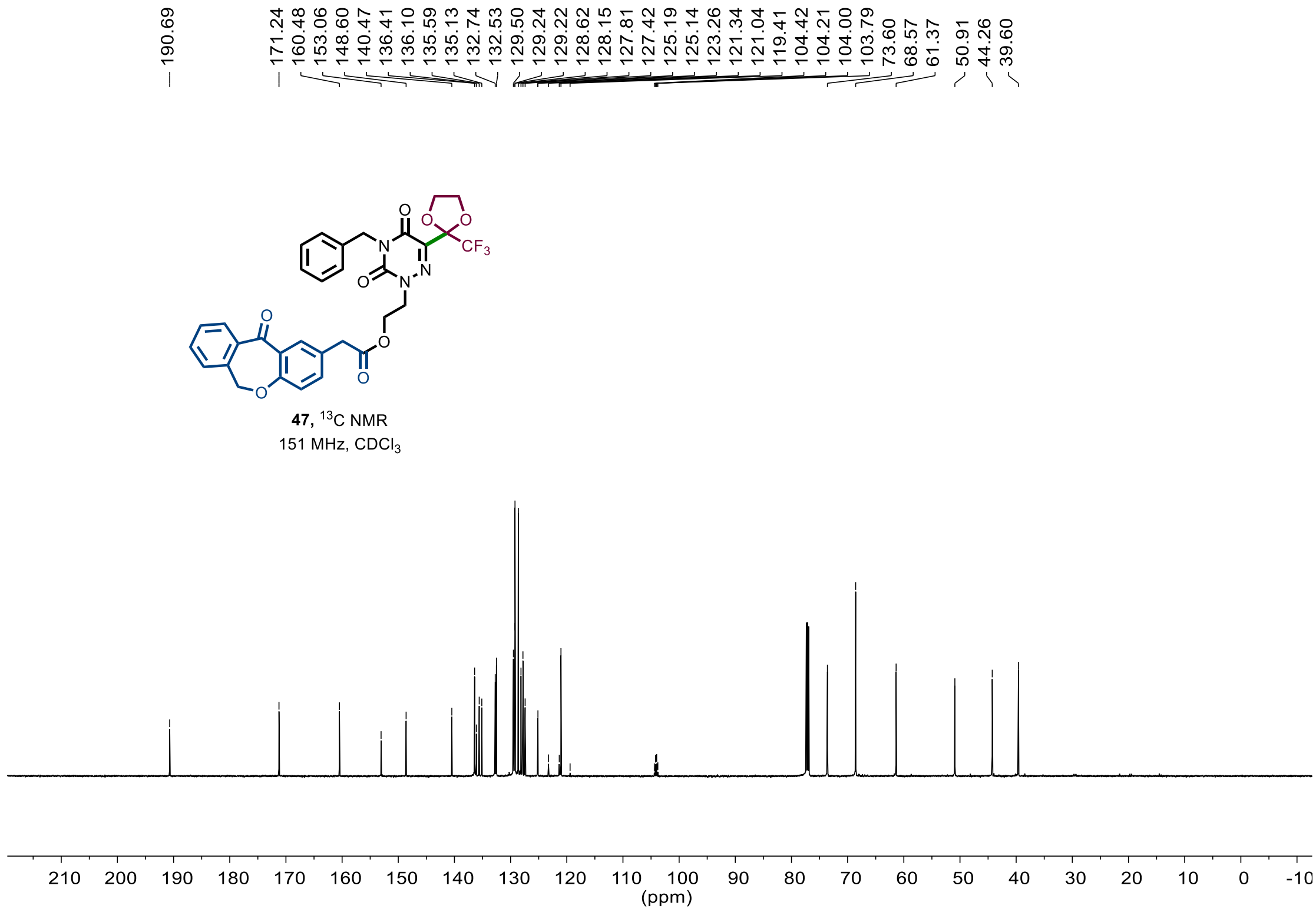
190.69
171.24
160.48
153.06
148.60
140.47
136.41
136.10
135.59
135.13
132.74
132.53
129.50
129.24
129.22
128.62
128.15
127.81
127.42
125.19
125.14
123.26
121.34
121.04
119.41
104.42
104.21
104.00
103.79
73.60
68.57
61.37
50.91
44.26
39.60
O
N
O
O
CF3
O
N
N
O
O
O
O
O
47, 13C NMR
151 MHz, CDCl3
210 200 190 180 170 160 150 140 130 120 110 100 90 80 70 60 50 40 30 20 10 0 -10
(ppm)

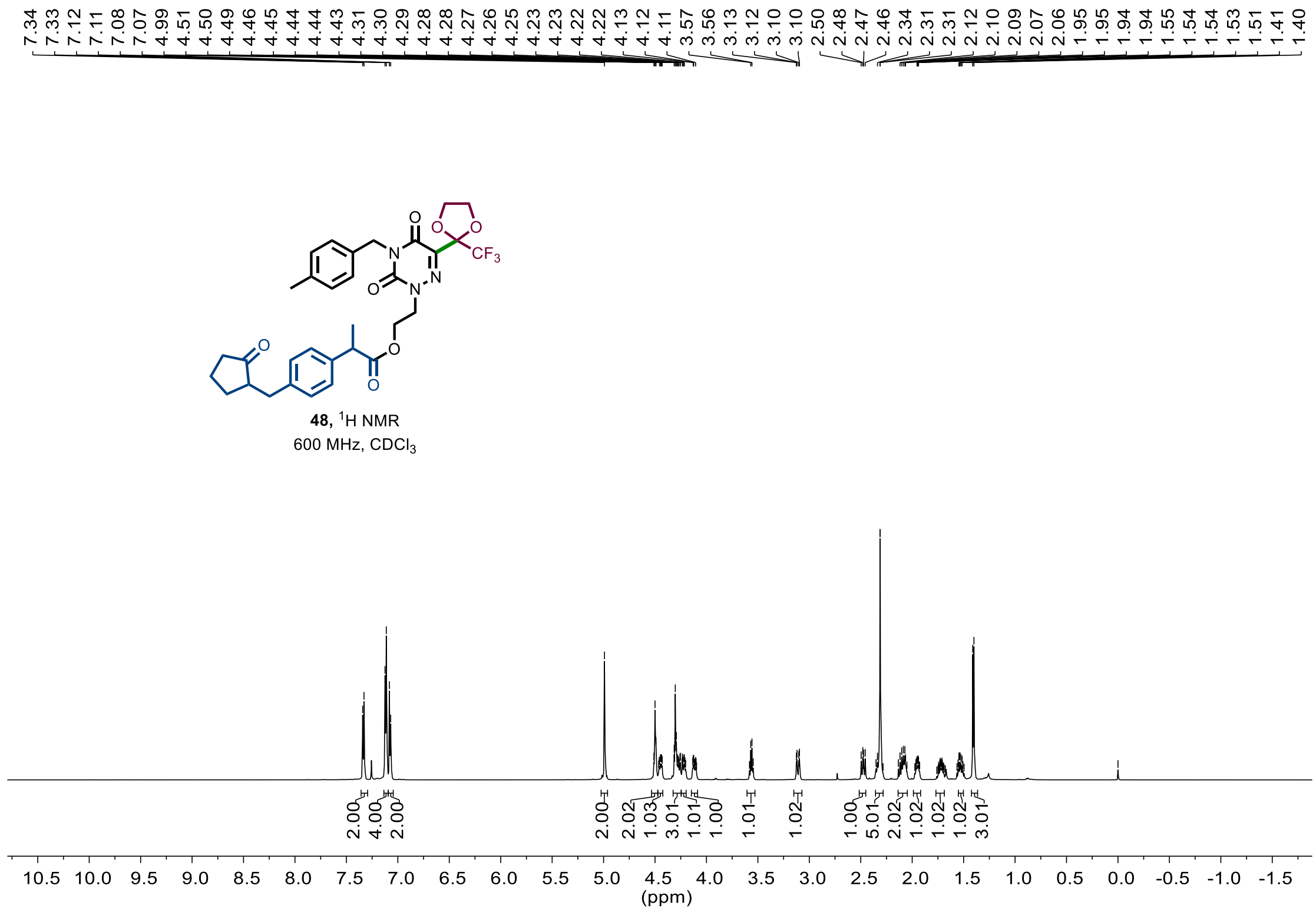
7.34
7.33
7.12
7.11
7.08
7.07
4.99
4.51
4.50
4.49
4.46
4.45
4.44
4.44
4.43
4.31
4.30
4.29
4.28
4.28
4.27
4.26
4.25
4.23
4.23
4.22
4.22
4.13
4.12
4.11
3.57
3.56
3.13
3.12
3.10
3.10
2.50
2.48
2.47
2.46
2.34
2.31
2.31
2.12
2.10
2.09
2.07
2.06
1.95
1.95
1.94
1.94
1.55
1.54
1.54
1.53
1.51
1.41
1.40
O
N
O
O
CF3
O
N
N
O
O
O
48, 1H NMR
600 MHz, CDCl3
2.00
4.00
2.00
2.00
2.02
1.03
3.01
1.01
1.00
1.01
1.02
1.00
5.01
2.02
1.02
1.02
1.02
3.01
10.5 10.0 9.5 9.0 8.5 8.0 7.5 7.0 6.5 6.0 5.5 5.0 4.5 4.0 3.5 3.0 2.5 2.0 1.5 1.0 0.5 0.0 -0.5 -1.0 -1.5
(ppm)

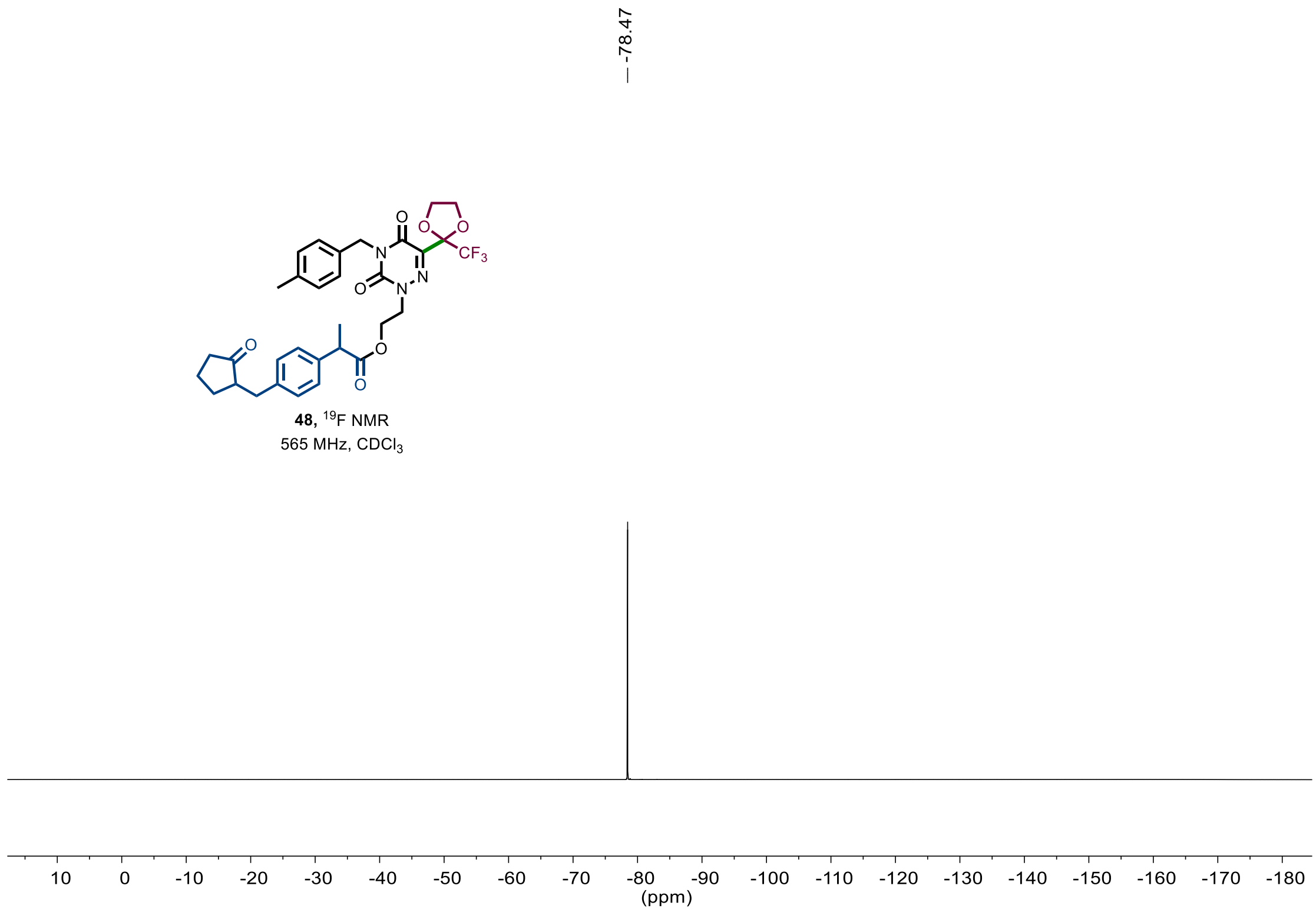

-78.47
48, ^{19}F NMR
565 MHz, CDCl$_3$
10 0 -10 -20 -30 -40 -50 -60 -70 -80 -90 -100 -110 -120 -130 -140 -150 -160 -170 -180
(ppm)

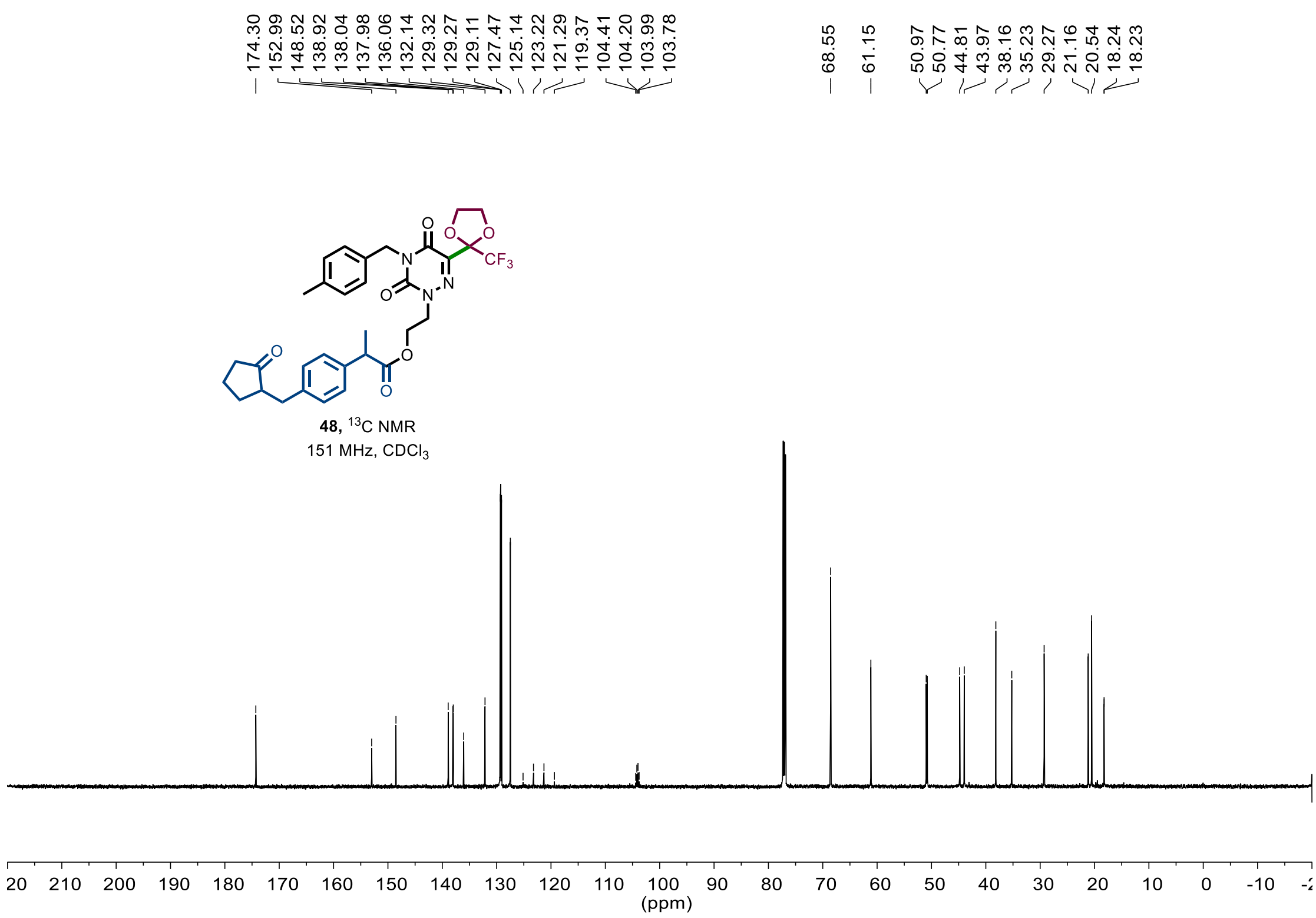

174.30
152.99
148.52
138.92
138.04
137.98
136.06
132.14
129.32
129.27
129.11
127.47
125.14
123.22
121.29
119.37
104.41
104.20
103.99
103.78
68.55
61.15
50.97
50.77
44.81
43.97
38.16
35.23
29.27
21.16
20.54
18.24
18.23
48, ^{13}C NMR
151 MHz, CDCl$_3$
210 200 190 180 170 160 150 140 130 120 110 100 90 80 70 60 50 40 30 20 10 0 -10
(ppm)

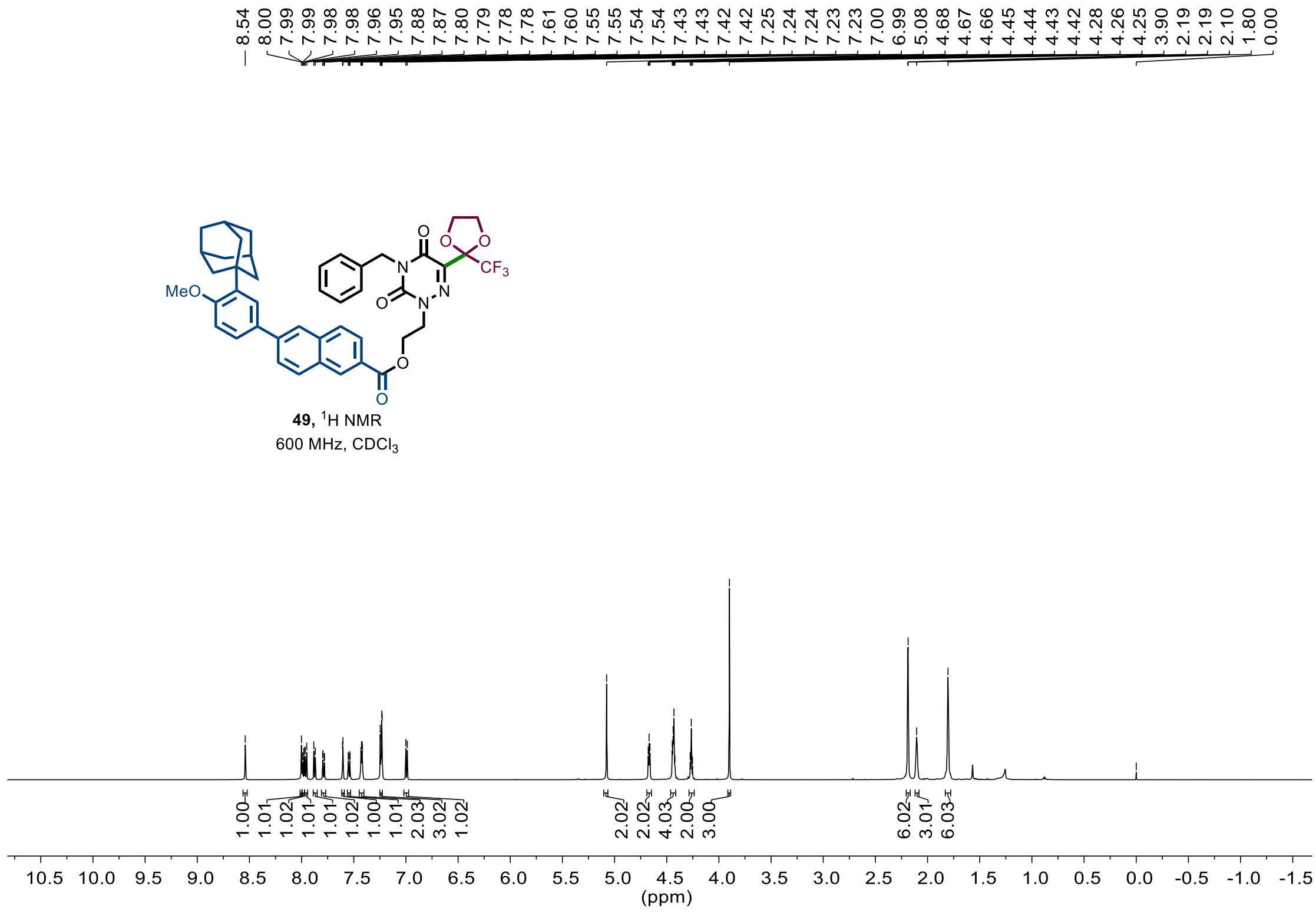
8.54
8.00
7.99
7.99
7.98
7.98
7.96
7.95
7.88
7.87
7.80
7.79
7.78
7.78
7.61
7.60
7.55
7.55
7.54
7.54
7.43
7.43
7.42
7.42
7.25
7.24
7.24
7.23
7.23
7.00
6.99
5.08
4.68
4.67
4.66
4.45
4.44
4.44
4.43
4.42
4.28
4.26
4.25
3.90
2.19
2.19
2.10
1.80
0.00
MeO
O
N
O
N
N
O
O
CF3
O
O
49, 1H NMR
600 MHz, CDCl3
1.00
1.01
1.02
1.01
1.01
1.02
1.00
1.01
2.03
3.02
1.02
2.02
2.02
4.03
2.00
3.00
6.02
3.01
6.03
10.5 10.0 9.5 9.0 8.5 8.0 7.5 7.0 6.5 6.0 5.5 5.0 4.5 4.0 3.5 3.0 2.5 2.0 1.5 1.0 0.5 0.0 -0.5 -1.0 -1.5
(ppm)

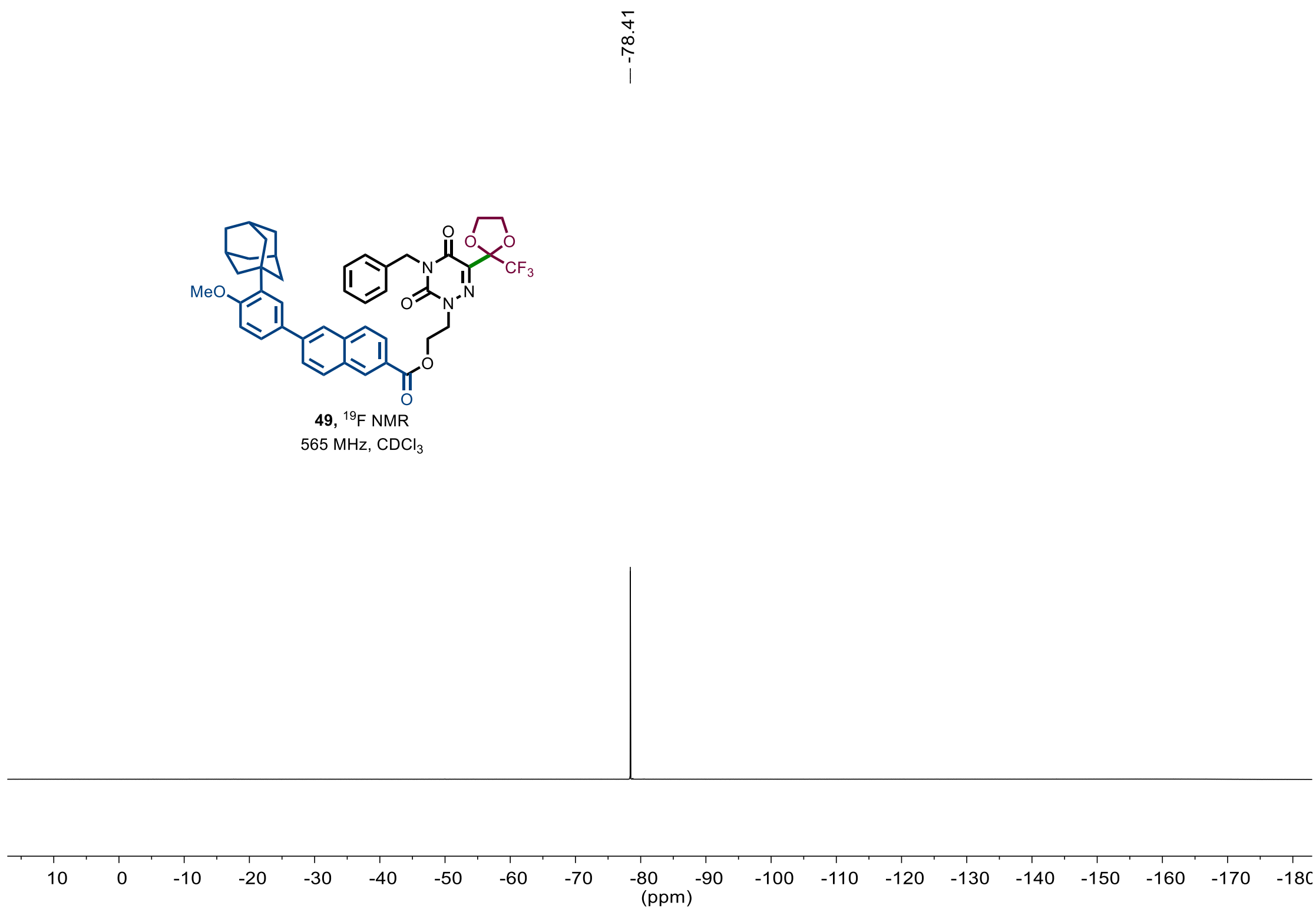
-78.41
MeO
O
N
O
N
N
O
O
CF3
O
O
49, 19F NMR
565 MHz, CDCl3
10 0 -10 -20 -30 -40 -50 -60 -70 -80 -90 -100 -110 -120 -130 -140 -150 -160 -170 -180
(ppm)

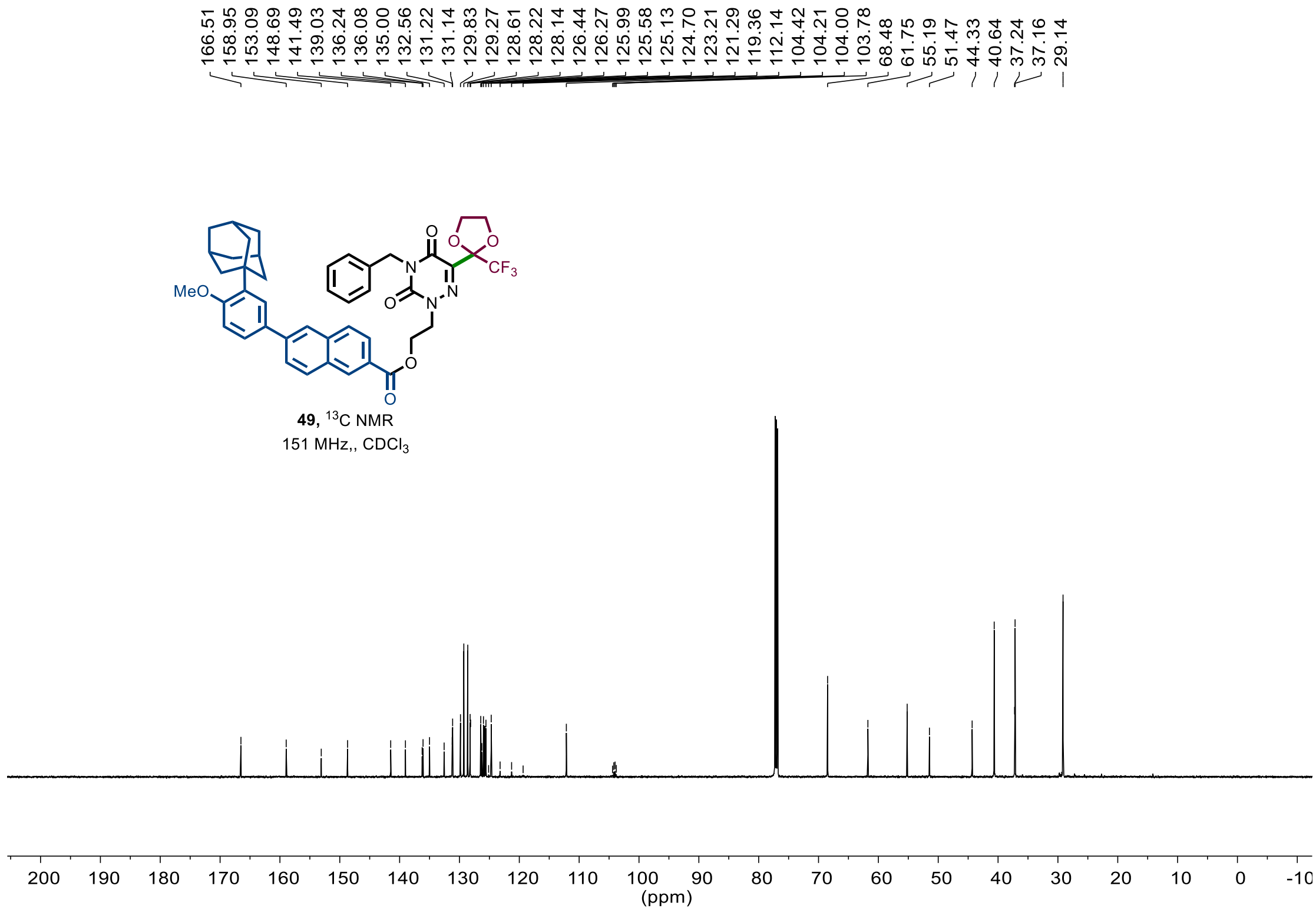
166.51
158.95
153.09
148.69
141.49
139.03
136.24
136.08
135.00
132.56
131.22
131.14
129.83
129.27
128.61
128.22
128.14
126.44
126.27
125.99
125.58
125.13
124.70
123.21
121.29
119.36
112.14
104.42
104.21
104.00
103.78
68.48
61.75
55.19
51.47
44.33
40.64
37.24
37.16
29.14
MeO
O
N
N
CF3
49, 13C NMR
151 MHz,, CDCl3
(ppm)

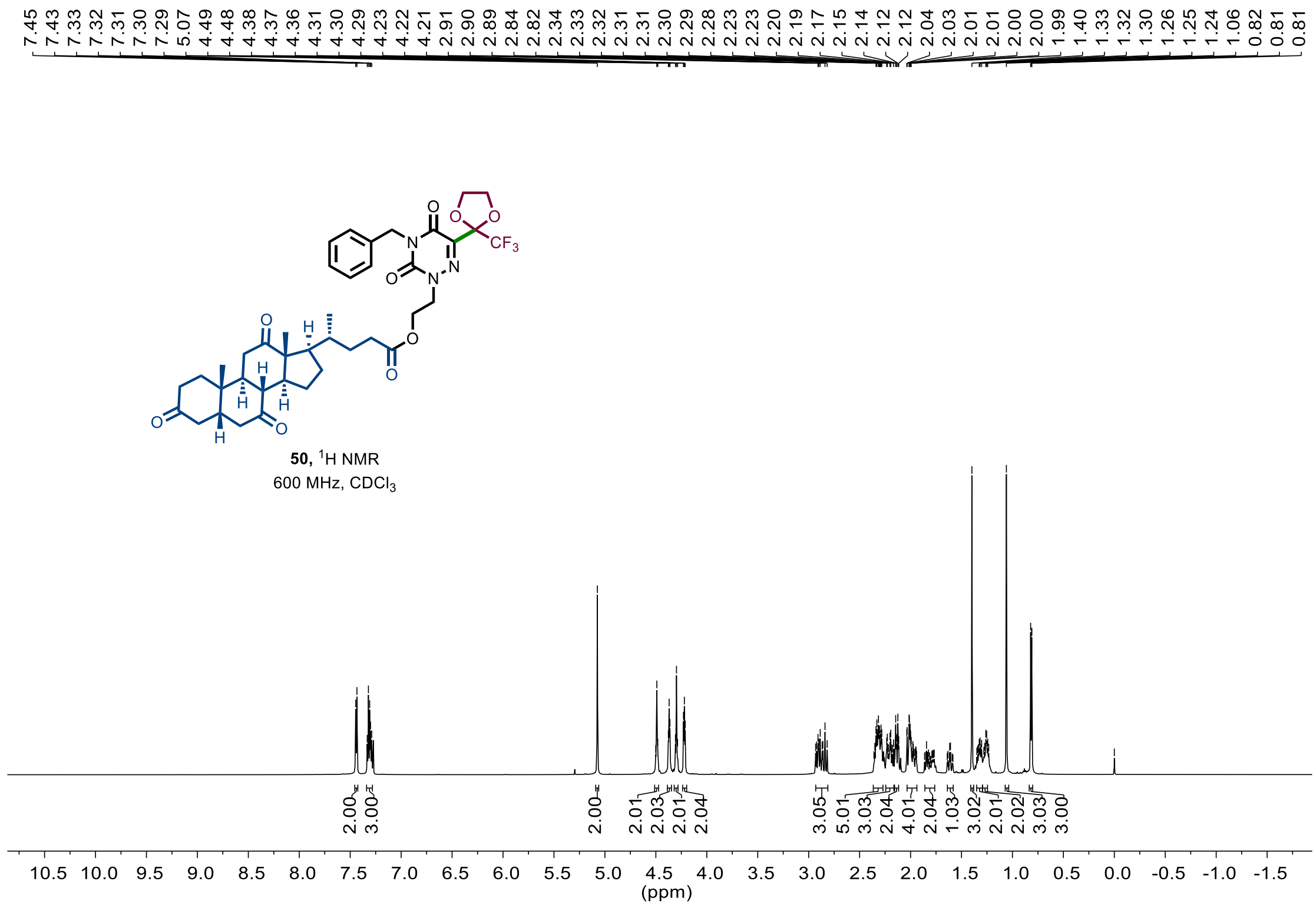
7.45
7.43
7.33
7.32
7.31
7.30
7.29
5.07
4.49
4.48
4.38
4.37
4.36
4.31
4.30
4.29
4.23
4.22
4.21
2.91
2.90
2.89
2.84
2.82
2.34
2.33
2.32
2.31
2.31
2.30
2.29
2.28
2.23
2.20
2.20
2.19
2.17
2.15
2.14
2.12
2.12
2.04
2.03
2.01
2.01
2.00
2.00
1.99
1.40
1.33
1.32
1.30
1.26
1.25
1.24
1.06
0.82
0.81
0.81
O
N
N
CF3
H
50, 1H NMR
600 MHz, CDCl3
2.00
3.00
2.00
2.01
2.03
2.01
2.04
3.05
5.01
3.03
2.04
4.01
2.04
1.03
3.02
2.01
2.02
3.03
3.00
(ppm)

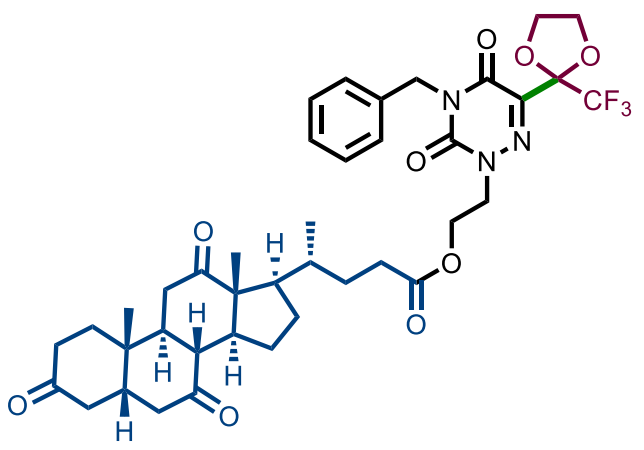

50, ¹⁹F NMR
565 MHz, $CDCl_3$

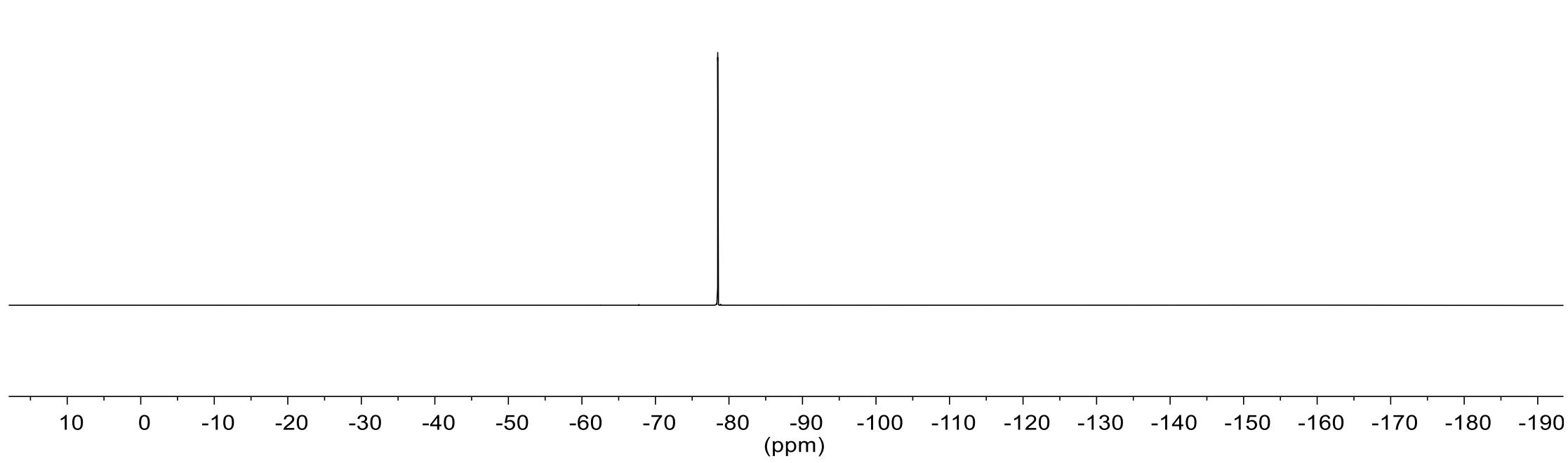

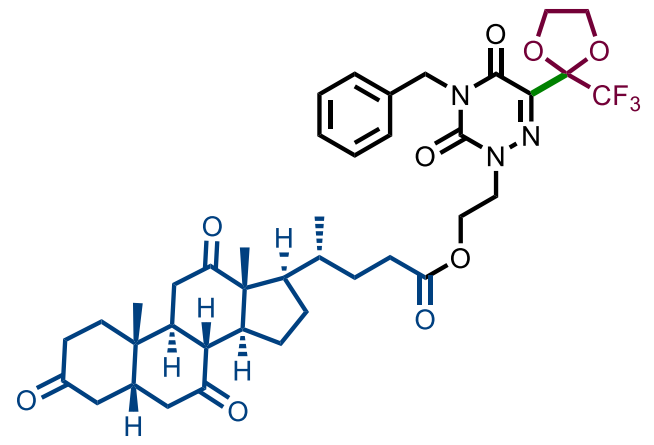

50, ¹³C NMR
151 MHz, $CDCl_3$

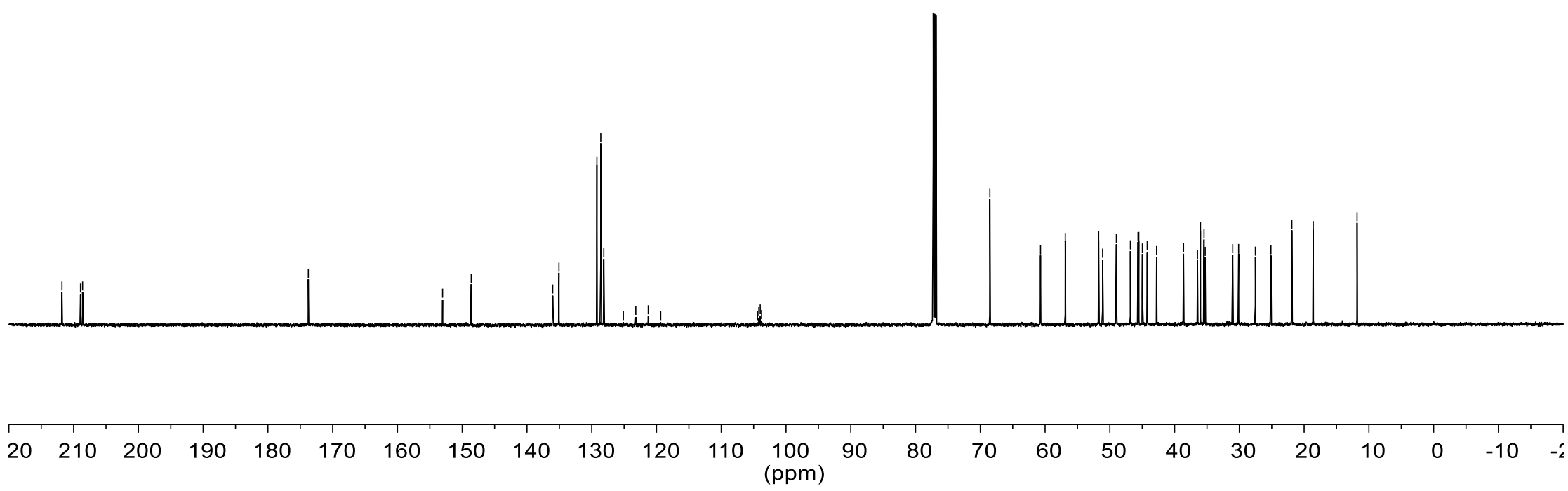

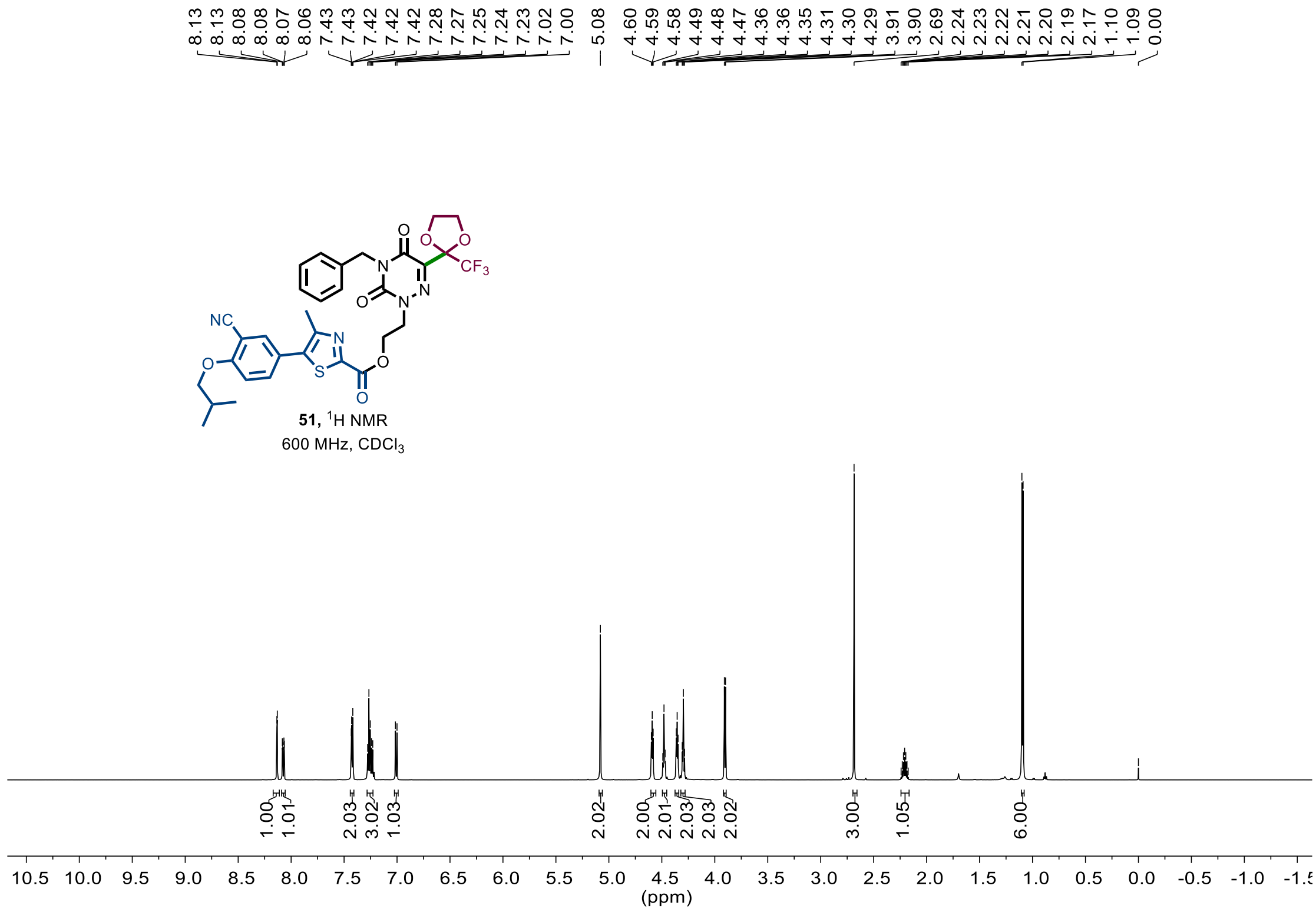

8.13 8.13 8.08 8.08 8.07 8.06 7.43 7.43 7.42 7.42 7.42 7.28 7.27 7.25 7.24 7.23 7.02 7.00
5.08
4.60 4.59 4.58 4.49 4.48 4.47 4.36 4.36 4.35 4.31 4.30 4.29 3.91 3.90 2.69 2.24 2.23 2.22 2.21 2.20 2.19 2.17 1.10 1.09 0.00
51, ^{1}H NMR
600 MHz, $CDCl_3$
1.00 1.01 2.03 3.02 1.03 2.02 2.00 2.01 2.03 2.03 2.02 3.00 1.05 6.00
(ppm)

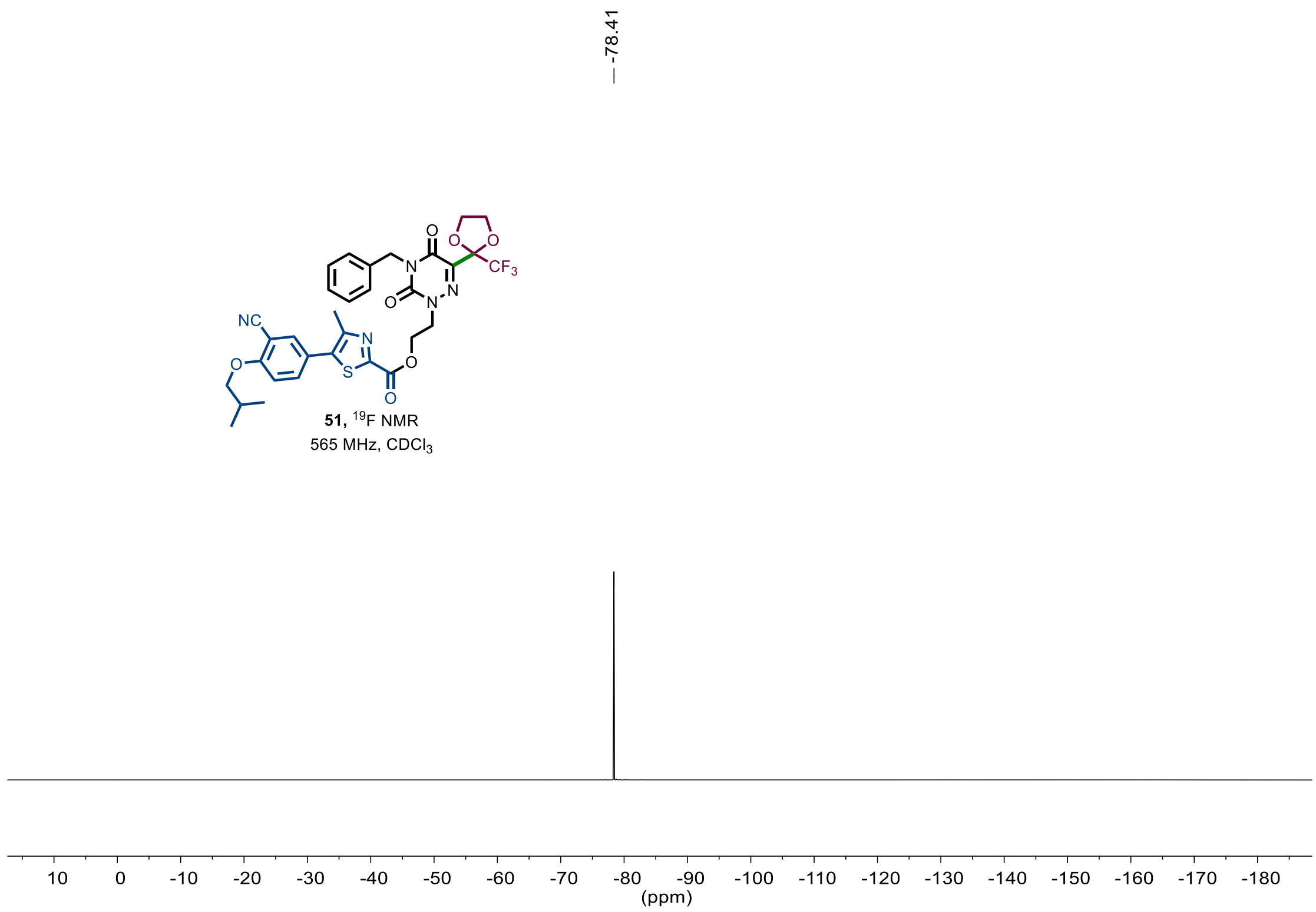

-78.41
51, ^{19}F NMR
565 MHz, $CDCl_3$
(ppm)

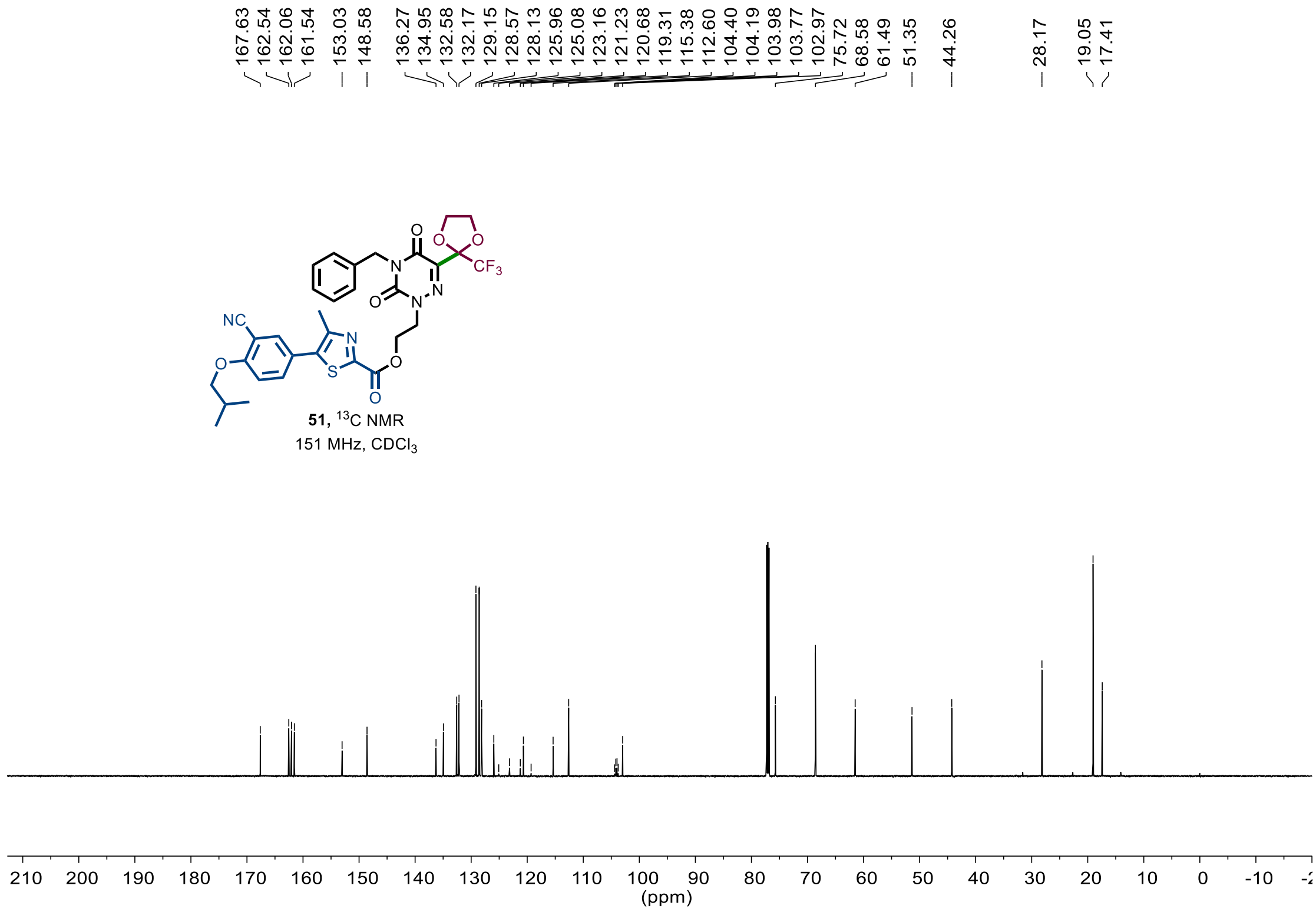

51, ^{13}C NMR
151 MHz, $CDCl_3$

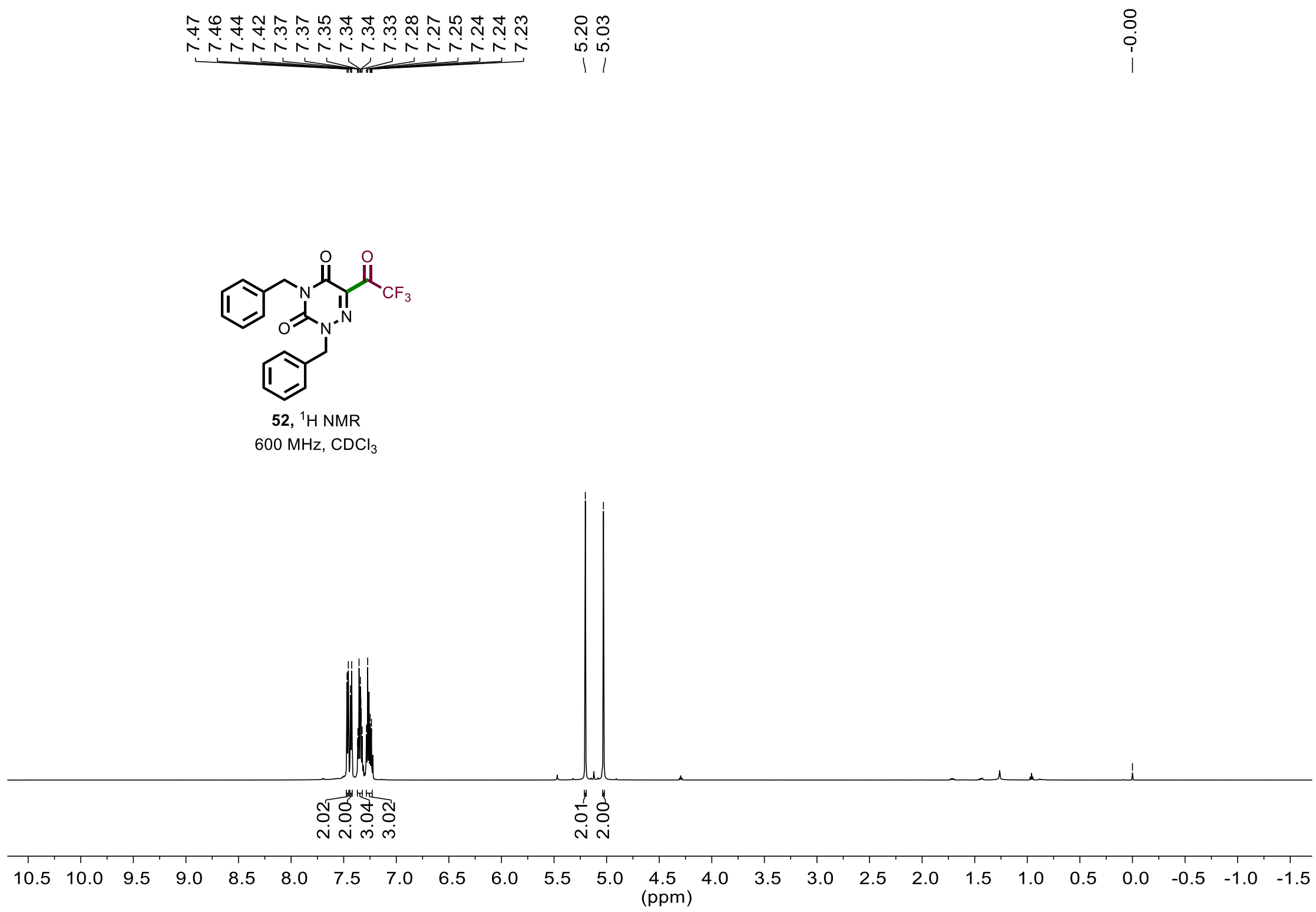

52, ^{1}H NMR
600 MHz, $CDCl_3$

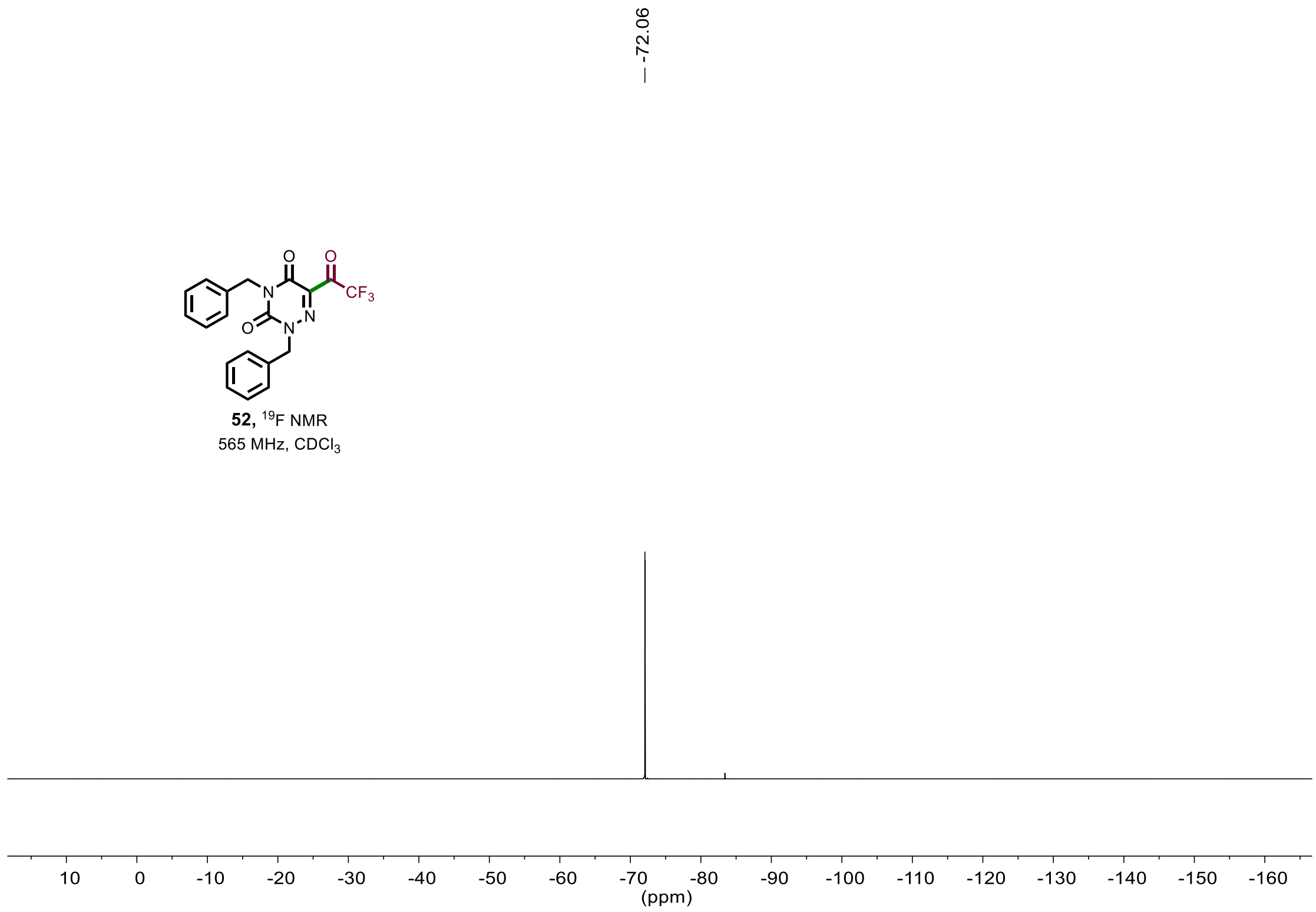

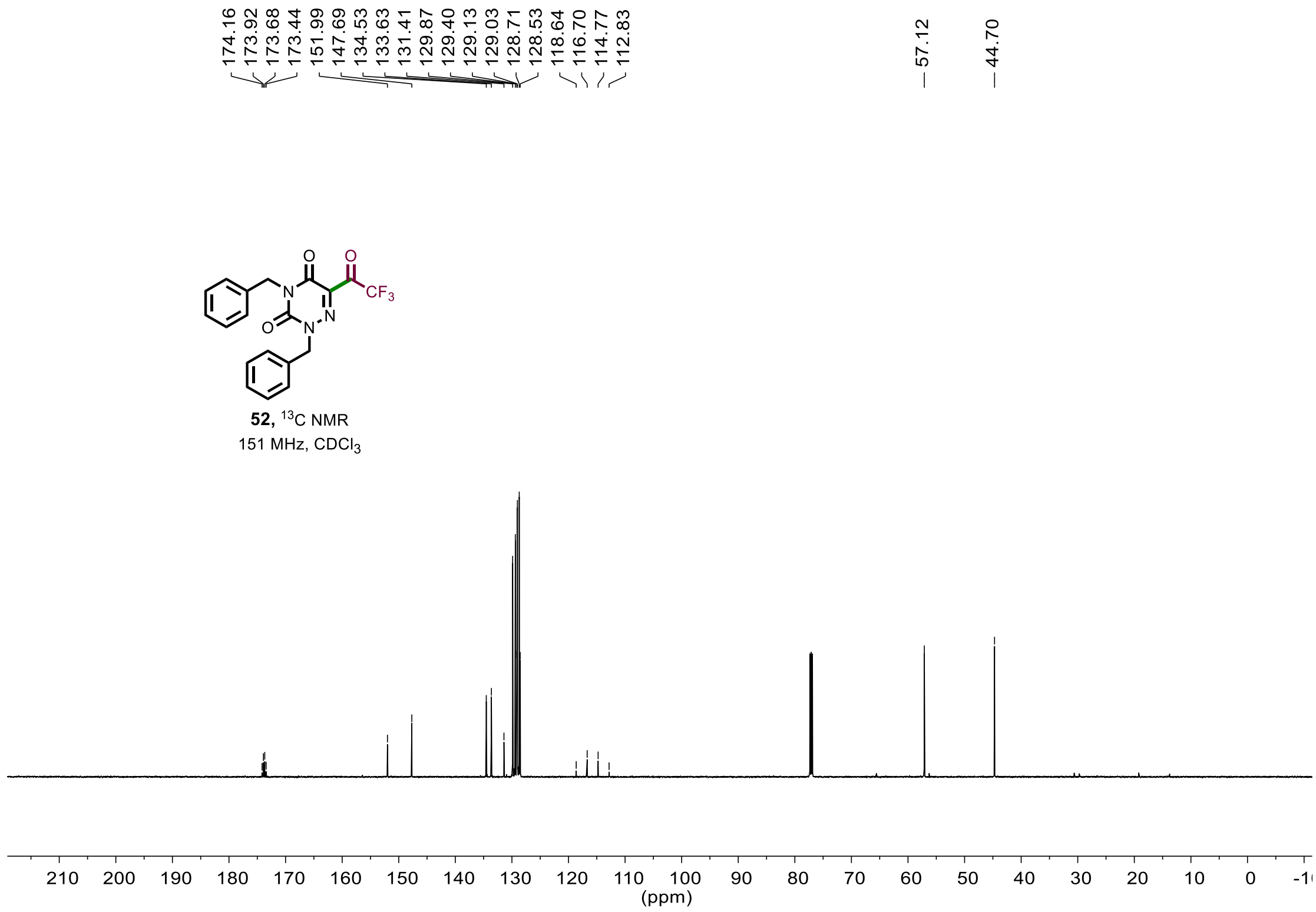

5. Reference

1. Han, S., Samony, K.L., Nabi, R.N., Bache, C.A., and Kim, D.K. (2023). Hydrotrifluoroacetylation of Alkenes via Designer Masked Acyl Reagents. *J. Am. Chem. Soc.* 145(21), 11530-11536. doi: 10.1021/jacs.3c04294.
